# Reward conditioning may not have an effect on category-specific memory

**DOI:** 10.1038/s41598-023-48874-z

**Published:** 2023-12-15

**Authors:** Priyanka Sukumaran, Nina Kazanina, Conor Houghton

**Affiliations:** 1 Faculty of Engineering, University of Bristol, Bristol, BS8 1UB UK; 2 School of Psychological Sciences, University of Bristol, Bristol, BS8 1TU UK; 3 International Laboratory of Social Neurobiology, HSE University, Moscow, Russia

**Keywords:** Human behaviour, Classical conditioning

## Abstract

Behavioural tagging facilitates the temporary storage of seemingly insignificant episodic events, which may later become salient and enhanced in memory. Human behavioural studies have demonstrated selective memory enhancement for neutral stimuli from one category when this category is subsequently paired with reward. Although this phenomenon has implications for the role of reward conditioning on emotional and adaptive memory, its generalisability is underexplored. We conducted four experiments to investigate whether pairing items from a semantic category, animals or objects, with high or low rewards resulted in preferential memory for the high-reward category. Three of these experiments also aimed to replicate the category-specific retrospective enhancement effect reported by Patil et al. and two explored the corresponding prospective memory effect. None of our experiments showed consistent evidence for an effect of reward on category-specific memory enhancement, despite employing the same reward paradigm and incidental encoding protocol as in the original study. Consequently, we found no evidence for category-specific retrospective or prospective enhancement effects. Our experiments were conducted online which is an equally relevant method for assessing behavioural phenomenon as the in-person studies conducted by Patil et al. Overall, our results question the generalisability of previously reported category-specific memory enhancement effects due to reward.

## Introduction

It is important to remember particularly emotional, rewarding or punishing events, as this information could be useful for predicting future decisions^1^. This function is facilitated by an adaptive memory system which temporarily stores memories that initially seem insignificant but later acquire salience through emotional experiences. Additionally, it might be advantageous that emotional experiences not only enhance the memory of a particularly salient event, but also other seemingly unimportant events that are conceptually, temporally or spatially related to that event. The synaptic tag-and-capture hypothesis^2^ provides an underlying neural mechanism for such adaptive memory effects, which has also gained human behavioural evidence with studies reporting ‘behavioural tagging’ effects^3^. For example, studies have shown that extrinsic reward (monetary incentives) retrospectively enhances memory for events with greater temporal^4^ and spatial^5^ closeness to rewarding events.

Interestingly, it has also been shown that conceptually related stimuli can be retrospectively enhanced in memory through reward^6^ and fear conditioning^7,8^. In the study by Patil et al.^6^ which we will refer to as the RREM (Reward Retroactively Enhances Memory) study, participants incidentally encoded neutral images from two categories, animals and tools, in a pre-conditioning phase. In a following conditioning phase, images from one category were associated with high reward and the other with low reward. A surprise 24-hour delayed recognition memory test revealed preferential memory for items from the high-reward category encoded in the conditioning as well as the pre-conditioning phase. Importantly, items from the pre-conditioning phase were never directly paired with reward. In other words, items from pre-conditioning phase were retrospectively enhanced in memory when items from the same semantic category were conditioned with high reward during the conditioning phase. While prior studies have shown general memory enhancement for neutral stimuli paired with salient events, regardless of their semantic category^9–11^, Patil et al.^6^ demonstrated that such memory effects can be highly specific and applied to conceptually related categories. However, the reliability of this effect is contested. A recent meta-analysis^12^ of 14 studies on selective retrospective memory enhancement induced by reward, fear and other salient conditioning, suggests these effects were inflated by small-study biases, resulting in Bayesian meta-analyses supporting the null hypothesis. Among the 14 studies, only the RREM study^6^ and Oyarzún et al.^13^ employed reward-conditioning. Despite using a similar monetary reward paradigm as the RREM study, with the only difference being indication of reward expectation on each trial, Oyarzún et al.^13^ found no evidence for a retrospective memory effect due to reward. They do report a strong effect of reward on category-specific memory for items directly paired with reward in the conditioning phase, and show evidence for prospective memory enhancement. The generalisability of reward-induced selective memory effects remains an open question.

We conducted four online experiments to investigate category-specific memory effects due to reward-conditioning. Experiment 1 aimed to investigate if the phenomenon found in the RREM study^6^ generalised to word and image stimuli in a more complex word-image associative learning paradigm. Experiment 1 did not show evidence for retrospective or prospective enhancement of category-specific memory due to reward. Moreover, the effects of conditioning on items that were explicitly rewarded during the conditioning phase were inconsistent across memory measures. This questions the generalisability of effects reported in the RREM study^6^. Next, Experiment 2a aimed to closely replicate the RREM study to investigate category-specific retrospective memory enhancements of images, while Experiment 2b tested the previously unexplored possibility of prospective memory enhancement using the same reward-conditioning protocol. Finally, Experiment 3 was a high-powered replication of Experiment 2a. Our experiments were conducted online, in contrast to the in-person RREM study^6^. We recognize the differences and trade-offs between online and in-person experiments: online testing allows for larger samples but may have reduced data quality, while in-person data collection is influenced by experimenter interaction, and limits the diversity and size of participant groups. However, numerous well-established psychological phenomena^14–17^, including those related to memory^18–20^ and reward processing^21–23^, yield consistent results across both settings; affirming the validity of online testing for behavioural research. It is thus informative that the two experimental conditions lead to different results, suggesting that category-specific memory effects due to reward^6^ do not reliably generalise with experimental variations.

**Figure 1:**
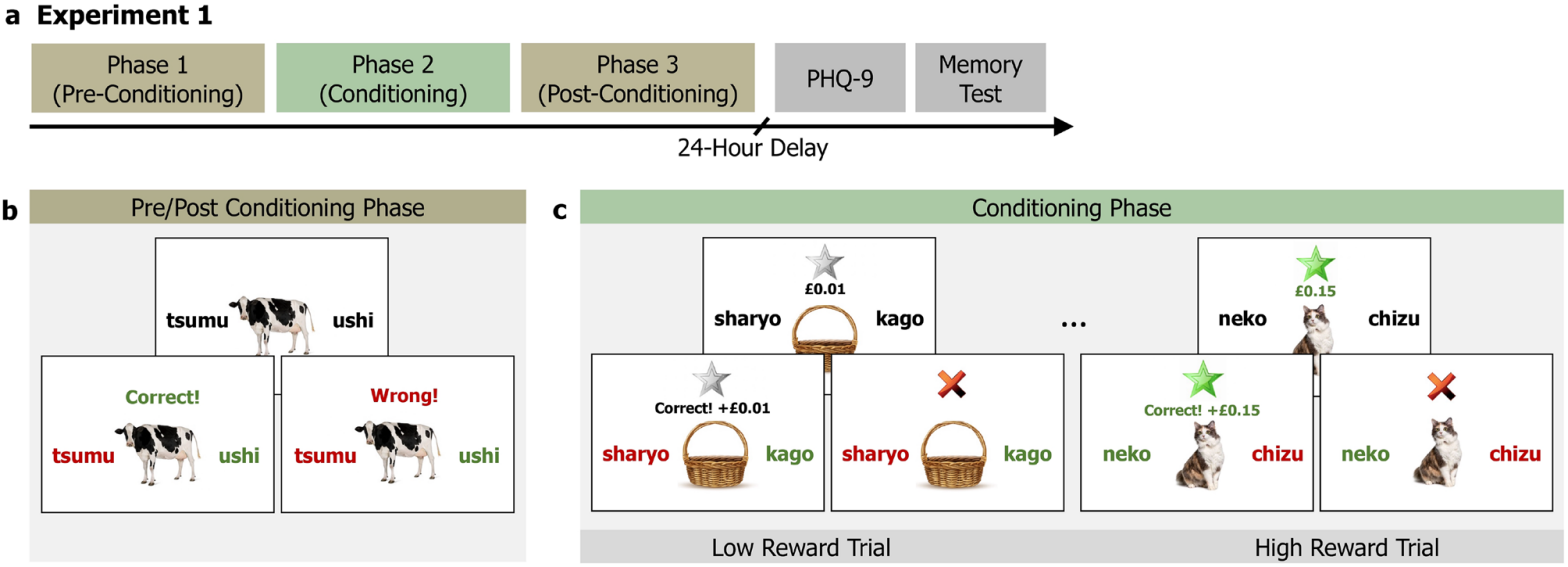
Schematic of Experiment 1 which tested retrospective and prospective effects of reward on 24-hour memory.

## Experiment 1

Experiment 1 was designed to test the generalisation of category-specific memory enhancement effects due to reward, as reported in the RREM study^6^, by employing an associative word-image encoding protocol. The word-learning protocol was adopted from a study by DeLoof et al.^24^ where participants successfully learn foreign word-to-image pairs through reward-prediction error conditioning. We use this learning paradigm along with the reward paradigm and incidental encoding design from the RREM study^6^ to investigate reward conditioning effects on category-specific memory of image and word stimuli. We test 24-hour delayed memory retrieval as this was the condition where the critical reward-conditioning effects emerged in the RREM study. In addition, we also carried out a version of Experiment 1 with immediate memory retrieval which did not show any evidence for category-specific memory effects due to reward conditioning, which is line with the RREM study, see Supplementary M1, Section F.

### Methods

#### Participants

A sample size of 120 participants, between the ages of 18–35, was targeted. As there were no previous studies with exact protocols testing selective enhancement of associative and item memory, the sample sizes were based on an *a priori* simulation of logistic regression models. We used pilot data from eight participants to estimate the necessary sample size for detecting a main effect of reward category on recognition memory. See pre-registration protocol for more details: https://osf.io/vghn4. Participants were required to have English as their first language and no literacy difficulty. 127 participants completed Experiment 1 online on prolific.co. The following were excluded: (1) participants with below-chance performance on the memory test; this was evaluated using *d*-prime scores, as described below in the “Data analysis” section below, and participants were excluded if their *d*-prime score was less than or equal to zero, (2) outliers in response times in the memory test (outside 1.5 times the interquartile range calculated across participants). The final group of 120 participants consisted of 86 females and 34 males, aged $M=26.17$, $SEM=0.46$. All participants in Experiments 1, 2 and 3 provided informed consent prior to the experiment. All experiments were approved by the School of Psychological Sciences Research Ethics Committee of University of Bristol and were performed in accordance with relevant guidelines and regulations.

#### Materials

The stimulus consisted of 192 images, 96 animals and 96 objects on white backgrounds, and 384 words which were Japanese nouns of two or three syllables. For each participant, image-word pairs from one semantic category, either animal or object, were associated with high reward and the other category with low reward. This association was learnt in the conditioning phase during encoding (see “Procedure” section below). Half the images and words were used during encoding and the other half as foils for a recognition memory test after encoding phases.

#### Procedure

The experiment consisted of three encoding phases: pre-conditioning, conditioning, and post-conditioning. Each phase had 32 trials, including 16 animal trials and 16 object trials. Allocation of stimuli to the three phases were randomised and additionally, stimulus order was pseudo-randomised such that no more than three trials from the same semantic category occurred consequently. On each trial of the pre-conditioning phase, participants were presented with an image of either an animal or an object and two Japanese words, one of which was the correct Japanese word for the image. The set of two words presented with a particular image remained the same for all participants, but were randomly positioned on the left or right of the image. Participants were given two seconds to guess the correct word, and feedback was provided to ensure they learned the correct word-image pairing: the correct word turned green, while the wrong word turned red.

During the conditioning phase, participants were told that guessing the correct Japanese word would result in earning £0.01 on grey star trials and $\pounds$0.15 on green star trials, and trial type would be indicated along with the image cue, see Fig. 1. Grey star, low-reward and green star, high-reward trials were each associated with one of the two image categories: animals or objects. However, participants were not informed of this association and had to learn it through the conditioning trials. Allocation of animal or object image to high- or low-reward category was randomised and counterbalanced across participants. After any exclusions, further participants were allocated categories while maintaining perfect counterbalancing. During each trial, as in the pre-conditioning phase, participants were presented with an image and two Japanese words, accompanied by a grey or green star, as well as the potential reward ($\pounds$0.01 or $\pounds$0.15). After selecting a word, any earned reward was displayed, together with feedback on whether the choice was correct or not. The post-conditioning phase was identical to the pre-conditioning phase, with no rewards or information about trial type provided.

Participants then completed a surprise recognition memory test 24 hours after the post-conditioning phase. To avoid any biases due to test expectancy, no prior indication had been given to participants that they would be asked to do a memory test beyond the learning task itself^25^. In the memory test, participants had to decide whether they had previously seen a given item (word or image) in the encoding phases. 192 images and 384 words, half of which were foils, were intermixed and randomly presented one after the other. For each trial, participants chose the most applicable response from: ‘definitely old’, ‘likely old’, ‘maybe old’, ‘maybe new’, ‘likely new’, ‘definitely new’. Participants also completed a mental health questionnaire and another association memory task, the results of which are not explored in this paper.

#### Data analysis

We follow a similar analysis approach as in the RREM study^6^, which is also detailed in our pre-registration protocol (https://osf.io/vghn4/). Recognition memory was quantified using corrected recognition scores: $R = H - F$, where *H* is the hit rate and *F* is the false alarm rate. In addition, we report a version of the analysis using the signal-detection theoretic measure of sensitivity *d*-prime ($d'$) as a measure of memory^26,27^: $d' = z(H) - z(F)$, where *z*(*H*) is *z*-scored hit rate and *z*(*F*) is *z*-scored false alarm rate. In order to compute *z*-scores, *H* and *F* were corrected to the range of 1% : 99%, as per standard practice followed in previous studies using *d*-primes^28^. The use of *d*-primes has been suggested to have advantages over other measures including corrected recognition, which are based on threshold models of recognition memory^28–31^.

For the main analysis, similar to RREM^6^ and Oyarzún et al.^13^, we conducted a 3 × 2 repeated measures analysis of variance (ANOVA) on memory measures (*d*-prime and corrected recognition) with encoding phase (pre-conditioning, conditioning, post-conditioning) and reward category (high, low) as within-subject factors. The ANOVA will indicate memory differences across phases and reward categories. To dissect specific effects within each phase, the effect of reward category on memory of items was further quantified using two-tailed paired *t*-tests with alpha=0.05. Cohen’s d(average) was used to estimate effect sizes for paired *t*-tests, referred to as $d_{av}$ in the text and tables^32^. We additionally used Bayesian counterparts for *t*-tests to quantify evidence in support of the null hypothesis. A Bayes factor:

1 $$\begin{aligned} BF_{10}=\frac{P(D|H_1)}{P(D|H_0)} \end{aligned}$$

was calculated, where *D* represents the data, $P(D|H_a)$ the probability of the data conditional on a hypothesis $H_a$. $H_0$ and $H_1$ are the two competing hypotheses, in this case $H_0$ is the null hypothesis that there is no effect of reward category on memory and $H_1$ is the alternative hypothesis that there is an effect of reward category on memory (i.e. items specifically drawn from high-reward category are enhanced in memory). We implemented this using the ttestBF function in R, with a Cauchy prior distribution and a default scale parameter of $r=0.707$. Bayes factors less than 0.33 signifies substantial evidence for $H_0$, whereas Bayes factors greater than three signifies substantial evidence for $H_1$. Anecdotal evidence for $H_0$ and $H_1$ corresponds to $0.33< BF_{10} < 1$ and $1< BF_{10} < 3$ respectively^33,34^.

In addition to the ANOVA and *t*-tests on memory measures, we also estimated generalised linear mixed-effects models (GLMM) on categorical responses from the memory test as described in the pre-registration protocol. We ran GLMM models with a logit-link function using the lme4 package in R^35^. The dependent variable was the binarised response in the memory test collapsed across certainty levels: responding ‘old’ or responding ‘new’. The GLMM included main effects of reward category and encoding phase, and the interaction between them. Random intercepts were included for each participant and stimuli items. All analysis was conducted in R 4.2.3.

**Figure 2:**
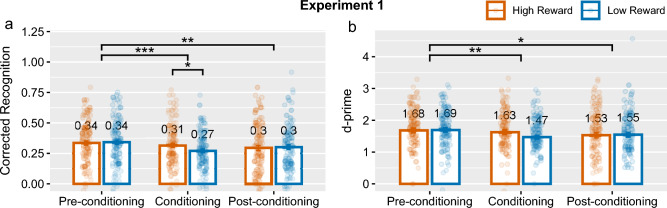
Memory performance measured by corrected recognition (left) and *d*-primes (right) in Experiment 1 by phase and reward category. Group means are displayed with error bars showing $\pm 1$ *SEM*. *$p < 0.05$, **$p < 0.01$, ***$p < 0.001$.

Furthermore, as in the RREM study, we repeated all the analysis steps using a subset of trials with only higher certainty responses from the memory test. This was done by including trials with responses ‘definitely old’, ‘likely old’, ‘likely new’, ‘definitely new’ and excluding trials in which participants chose ‘maybe old’ or ‘maybe new’. Experiment code, analysis scripts and raw data are available at: https://github.com/prisukumaran23/adaptiveMemoryReplication.

**Table 1:** Summary of *t*-tests in Experiment 1.

		Frequentist *t*-test			Bayesian *t*-test	
		t(119)	*p*	${d_{av}}$	${BF_{10}}$	Interpretation
Pre-conditioning	CR	− 0.38	0.71	− 0.03	0.08	Substantial evidence for $H_0$
	DP	− 0.21	0.83	− 0.02	0.08	Substantial evidence for $H_0$
Conditioning	CR	2.33	0.02*	0.25	2.70	Anecdotal evidence for $H_1$
	DP	1.97	0.05$\dagger$	0.22	1.28	Anecdotal evidence for $H_1$
Post-conditioning	CR	− 0.28	0.78	− 0.03	0.08	Substantial evidence for $H_0$
	DP	− 0.21	0.83	− 0.02	0.09	Substantial evidence for $H_0$

Frequentist paired *t*-test for effect of reward category (high vs. low) on memory annotated with: $\dagger$ $p< 0.1$, *$p< 0.05$, **$p< 0.01$, ***$p< 0.001$. Bayesian *t*-test measures the evidence for $H_1$: one sided hypothesis that effect of reward is greater than zero, i.e. better memory for items in the high-reward category versus $H_0$: no effect of reward category. Bayes factors $\mathbf {BF_{10}}$ are interpreted as providing substantial or anecdotal evidence in favor of $H_1$ or $H_0$. CR, corrected recognition; DP, *d*-primes.

### Results

#### Overall performance

Guessing accuracy did not significantly differ between image category (animal vs. object) in any of the three phases, $p >.15$, or between high- and low-reward trials in the conditioning phase, $t_{(119)}=-1.53$, $p=0.13$, $d_{av}=-.19$. The average hit rate was $M=0.51$, $SEM=0.15$, and the average false alarm rate was $M=0.19$, $SEM=0.12$. See Supplementary M1, Table S2 for breakdown of memory test responses by certainty.

#### Recognition memory by phase and reward category

A repeated measures ANOVA revealed an effect of phase, $F(1, 119)=7.29$, $p=0.001$, $\eta ^{2}=0.005$, on corrected recognition. There was a significant interaction effect between encoding phase and reward category, $F(1, 119)=3.46$, $p=0.03$, $\eta ^{2}=0.004$, suggesting that the effect of reward category varied with phase. The *d*-prime analysis revealed an effect of phase, $F(1, 119)=4.48$, $p=0.02$, $\eta ^{2}=0.008$, but no interaction effect between encoding phase and reward category, $F(1, 119)=2.45$, $p=0.09$, $\eta ^{2}=0.003$, unlike the corrected recognition analysis.

For items encoded in the conditioning phase, *t*-tests revealed significant evidence for an effect of reward category on corrected recognition, $t(119)=2.33$, $p=0.02$, $d_{av}=0.25$, but this was diminished with *d*-primes, $t(119)=1.97$, $p=0.05$, $d_{av} =0.22$, see Fig. 2. This suggests that reward conditioning was successful, albeit weak, and emerged after a 24-hour post-consolidation period as found in the RREM study^6^. However, there was no significant evidence for an effect of reward-category on corrected recognition nor on *d*-primes for items encoded in the pre- and post-conditioning phases, see Table 1 for *t*-tests.

#### Bayesian analysis

The Bayesian hypothesis test on corrected recognition of items from the conditioning phase revealed anecdotal evidence for the one-sided alternative hypothesis $H_1$ that the reward category effect is greater than zero, $BF_{10}$ $=2.70$. The equivalent analysis with *d*-primes revealed weaker evidence in favor of $H_1$, $BF_{10}$ $=1.28$, which is consistent with the *t*-test analysis above. For items in the pre- and post-conditioning phases, there was substantial evidence for the null hypothesis, with all Bayes factors less than 0.33. Although there was some anecdotal evidence for $H_1$ when analysing all memory trials, analysis focusing on higher certainty responses did not support this and showed evidence in favor of the null hypothesis, see Supplementary M1, Table S6. Additionally, linear mixed-effects modelling on categorical response data, presented in Supplementary M1, Table S11–S12, also showed that there were no significant interaction effects between reward category and the three phases.

### Discussion

In Experiment 1, a significant effect of reward category on items in the conditioning phase was observed when measured by corrected recognition, but not by *d*-primes or when analyzing only higher certainty memory responses. This questions the generalisability of memory enhancement effects found using this reward conditioning paradigm, and suggests that the distinction between high- and low-reward category was not salient enough in our experiment. Given that the overall design was close to the original RREM study^6^, it is unclear why we did not find consistent evidence for category-specific memory enhancement effects and why any effects found were not significant when evaluated using *d*-prime measures and higher certainty responses.

However, our experimental design deviates from the RREM study in a few ways. Firstly, our study only had 32 images per phase whereas the RREM study had 60. Secondly, the RREM study used only image cues and a delayed match-to-sample task, whereas our study had a more complicated learning paradigm with word-image associations and a guessing task. In our study, recognition memory could have been affected by salient effects related to the guessing task, for example wrong guesses could lead to diminished memory. In order to characterise this effect, we estimated GLMMs on categorical responses from the memory test with phase and guess outcome as a predictor, see Supplementary M2, section 1.B.2.1, pg. 97–98, which revealed that guess-outcome (correct/wrong feedback) did not significantly affect memory for items in the pre-conditioning or conditioning phases, however, there was a significant but weak effect for items in the post-conditioning phase. Since guess-outcome did not differ significantly across reward category (high/low) and semantic category (animal/object), any effects would have been equal across categories, leading to no differences in memory.

Thirdly, in the RREM study, the rewards were motivated by an overall bonus of $20 or $1 for high matching performance for items in each category. In our study, the potential monetary reward was shown on each trial as $$\pounds$$0.15 or $$\pounds$$0.01, which could have induced an interfering reward-anticipatory response. Interestingly, in the study by Oyarzún et al.^13^ which also failed to find evidence for reward-induced category-specific retrospective memory effects, participants answered a (yes/no) reward anticipation question on each trial. This indicates that reward conditioning may be influenced by minor changes to factors such as anticipation, overall motivation and reward outcome in the conditioning paradigms. Relatedly, reward prediction error (RPE) conditioning, as opposed to explicit reward conditioning as used in our experiments, was successful in increasing recognition memory of images in the same foreign-word and image learning protocol^24^ that we adopted for our experiment. Since RPE accounts for the effects of reward anticipation and outcome, it may be a better measure of reward salience than explicit reward^23^. Further work is needed to explore the role of explicit reward versus RPE conditioning in inducing adaptive memory effects.

The neurobiological literature on memory suggests that associative memory retrieval is more reliant on the hippocampal activity than item recognition memory^36,37^. Consequently, associative memory tasks may, in theory, offer an alternative memory assay to probe the dopaminergic modulation of the hippocampus by rewards, which is the proposed neural mechanism underlying behavioural tagging with rewards^1–3^. Our finding that selective memory effects due to reward, reported by Patil et al.^6^, do not generalise to a previously unexplored word-image associative memory task, calls for further research to characterise associative memory in behavioural tagging experiments, as well as in the broader context of reward-memory studies.

**Figure 3:**
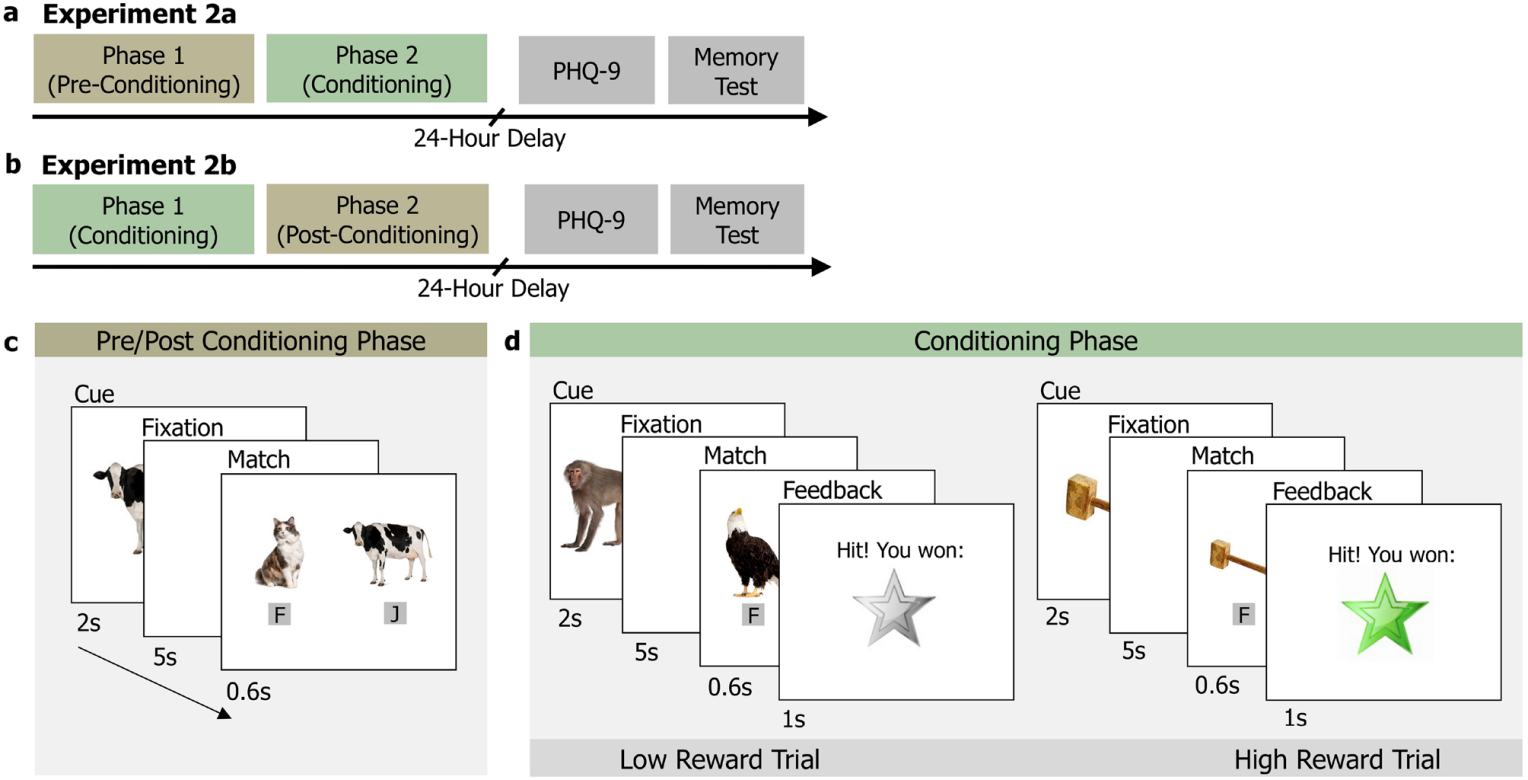
Schematic illustration of Experiment 2. Experiment 2a tested for retrospective memory enhancement with a pre-conditioning phase followed by a conditioning phase. Experiment 2b tested for prospective memory enhancement and participants completed the conditioning phase followed by post-conditioning phase. Participants completed a surprise recognition memory test 24 hours after encoding phases.

## Experiment 2

In Experiment 2, we follow the same protocols for incidental learning and reward conditioning as in the RREM study^6^, and reduce differences that could have resulted in weaker reward-conditioning and category-specific memory enhancement. Experiment 2a replicates the design of Experiment 1 from the RREM study^6^ to test retrospective memory enhancement 24-hours after encoding. In Experiment 2b, we incorporated a post-conditioning phase to examine prospective memory enhancement effects, building on prior reports of this effect induced by reward-conditioning^13^ and fear-conditioning^7^.

### Methods

#### Participants

We targeted a sample size of 60 participants for each experiment (age range=18–35 years) based on an *a priori* sample size estimation on G*Power 3.1 detailed in our pre-registration: https://osf.io/mbj62. Based on the original effects reported in the RREM study^6^, we aimed for an effect size of $d=0.61$, where *d* indicates Cohen’s $d_z$^32^. A minimum sample size of $n=44$ was required to see an effect of $d=0.61$ on a two-tailed paired t-test with $\alpha =0.01$ and $power=0.90$; this was increased to $n=60$, as at least a 20% increase is commonly recommended for online testing^17^. A total of 62 participants in Experiment 2a and 66 participants in Experiment 2b completed the two-day study online on prolific.co. Participants scoring below a 60% accuracy threshold on the match-to-sample task were excluded. This was used as an attention gauge during the experiment to remove participants who were choosing random options during encoding. This criterion was added after pre-registration to ensure high quality results. The following exclusions were also carried out: (1) participants with *d*-prime values less than or equal to zero, (2) outliers with mean response times in the memory test outside 1.5 times the inter-quartile range across participants. After exclusions, we allocated animal or object as the high-reward category to all further participants to maintain counterbalancing. In Experiment 2a, the final 60 participants consisted of 40 females, 20 males, aged $M=27.80$, $SEM=0.61$. In Experiment **2b** the final 60 participants included 34 females, 26 males, aged $M=28.15$, $SEM=0.62$.

#### Materials

The stimulus set was obtained from the authors of the RREM study^6^, and consisted of 240 images with 120 animal images and 120 object images isolated on white backgrounds.

#### Procedure

Participants completed two phases of incidental encoding in each experiment: Experiment 2a had a pre-conditioning phase followed by a conditioning phase, whereas in Experiment 2b, participants underwent the conditioning phase first, followed by a post-conditioning phase, see Fig. 3. There were 60 trials in each encoding phase (30 animal and 30 object). Stimuli were pseudo-randomised for each participant such that no more than three images from the same category occurred consequently. On each trial, participants were presented with an image for two seconds, followed by a fixation cross for five seconds, and then a match-to-sample task. The delayed match-to-sample task presented the original image and a new image, and the participant had to select the old image by pressing the ‘F’ key for left and ‘J’ key for right (Fig. 3c).

During the conditioning phase, low-reward trials were associated with a grey star and high-reward trials with a green star. The grey star, low-reward, and green star, high-reward trials were each associated with one of the two image categories, animal or object. As in the RREM study, participants were instructed that correct matching performance of 90% or more on ‘green star’ trials would result in a $\pounds$5 bonus, whereas a performance of 90% or more on ‘grey star’ trials would result in a $\pounds$0.25 bonus. They were not explicitly told that high- and low-reward categories were associated with a particular image category. After each match-to-sample trial, a feedback screen indicated correct or incorrect match along with a green or grey star indicating the high- or low-reward category of the trial (Fig. 3d).

After a 24-hour delay, participants completed a surprise recognition memory test where they had to determine if they had seen a given image during the encoding phases. A total of 240 images, with half being foils, were presented sequentially and participants selected the most appropriate response from the following options: ‘definitely old’, ‘likely old’, ‘maybe old’, ‘maybe new’, ‘likely new’, ‘definitely new’. Following this, participants also answered survey questions about how surprised they were about the memory test (see Supplementary M1, Section E). They also completed a mental health questionnaire, the results of which are not explored in this study.

**Figure 4:**
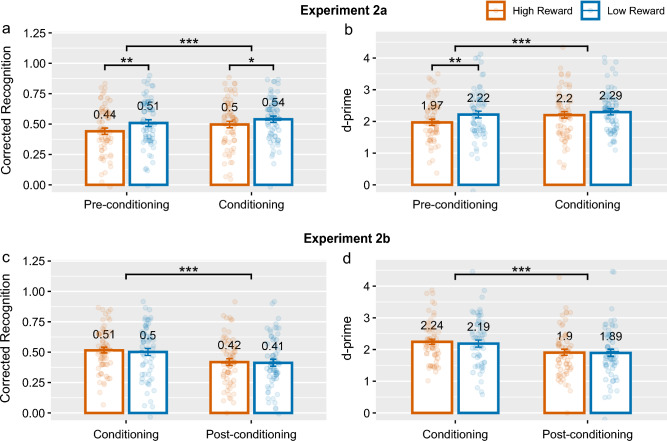
Memory performance measured by corrected recognition (left) and *d*-primes (right) in Experiment 2 by phase and reward category. Group means are displayed with error bars showing $\pm 1$ *SEM*. *$p < 0.05$, **$p < 0.01$, ***$p < 0.001$.

#### Data analysis

Data analysis was identical to Experiment 1. In addition, we also repeated all the analysis steps after excluding participants who were not surprised by the memory test, as done in the RREM study, to ensure that this did not significantly change our main results (see Supplementary M2, Sections 2.A.4.2 and 2.B.2).

**Table 2:** Summary of *t*-tests in Experiment 2a and 2b.

		Frequentist *t*-test			Bayesian *t*-test	
		t(59)	*p*	${d_{av}}$	${BF_{10}}$	Interpretation
Experiment 2a						
Pre-conditioning	CR	− 3.42	0.001**	− 0.33	47.97	Substantial evidence for $H_1$
	DP	− 3.24	0.002**	− 0.31	29.20	Substantial evidence for $H_1$
Conditioning	CR	− 2.30	0.03*	− 0.21	3.16	Substantial evidence for $H_1$
	DP	− 1.31	0.19	− 0.12	0.57	Anecdotal evidence for $H_0$
Experiment 2b						
Conditioning	CR	0.62	0.53	0.06	0.25	Substantial evidence for $H_0$
	DP	0.67	0.51	0.07	0.26	Substantial evidence for $H_0$
Post-conditioning	CR	0.30	0.76	0.03	0.18	Substantial evidence for $H_0$
	DP	0.12	0.90	0.01	0.16	Substantial evidence for $H_0$

Frequentist paired *t*-test for effect of reward category (high vs. low) on memory annotated with: $\dagger$ $p< 0.1$, *$p< 0.05$, ** $p< 0.01$, ***$p< 0.001$. Bayesian *t*-test $H_1$: one sided hypothesis that effect of reward is greater than zero, i.e. better memory for items in the high-reward category, $H_0$: no effect of reward category. Bayes factors $\mathbf {BF_{10}}$ are interpreted as providing substantial or anecdotal evidence in favor of $H_1$ or $H_0$. CR: corrected recognition, DP: *d*-primes.

### Results

### Experiment 2a

#### Overall performance

Matching accuracy was calculated to analyse the performance on the match-to-sample task during encoding phases. Matching accuracy was significantly above chance (50%) in both phases, all $p <.001$, and did not significantly differ between image category (animal vs. object) in neither phases, $p > 0.09$. For the conditioning phase, matching accuracy did not differ between high- and low-reward trials, $t_{(59)}=-0.77$, $p=0.44$, $d_{av}=0.08$. Participants achieved an average hit rate of $M=0.70$, $SEM=0.14$ and false alarm rate of $M=0.20$, $SEM=0.11$ on the memory test.

#### Recognition memory by phase and reward category

Recognition memory performance by phase and reward category is shown in Fig. 4. First, we conducted an ANOVA on memory performance (both corrected recognition and *d*-prime) with phase (pre-conditioning, conditioning) and reward category (high reward, low reward) as independent variables. The ANOVA revealed a strong effect of phase, $F(1,59)=12.76$, $p <0.001$, $\eta ^{2}=0.01$, and reward category, $F(1,59)=11.95$, $p=0.001$, $\eta ^{2}=0.02$ on corrected recognition, but no evidence for a significant interaction effect, $F(1,59)=12.76$, $p=0.24$, $\eta ^{2}=0.001$. In line with the corrected recognition analysis, the ANOVA on *d*-primes revealed a strong effect of phase, $F(1,59)=10.97$, $p=0.002$, $\eta ^{2}=0.01$, and reward category $F(1,59)=7.99$, $p=0.006$, $\eta ^{2}=0.01$, but no intersction thereof $F(1,59)=3.14$, $p=0.082$, $\eta ^{2}=0.002$.

Further *t*-tests were conducted to characterise these results. For the items encoded in the conditioning phase, there was a significant effect of reward category on corrected recognition $t(59)=-2.30$, $p=0.025$, $d_{av}=-.21$, although, surprisingly it was in favor of the low-reward category. In other words, items in the conditioning phase which belonged to the low-reward category resulted in enhanced memory, which is in the opposite direction compared to that reported in the RREM study. However, this effect was not seen with the *d*-prime scores, $t(59)=-1.31$, $p=0.19$, $d_{av}=-.12$.

Consistent with the reward category effect favoring the low-reward category during the conditioning phase, we also found evidence for category-specific retrospective memory enhancement for items encoded in the pre-conditioning phase. The evidence was strong with corrected recognition as well as *d*-primes with $p <0.002$ in each case, see Table 2. Importantly, the observed effect of reward category on recognition memory in the pre-conditioning and conditioning phase was no longer significant in both corrected recognition and *d*-prime analyses when focusing on higher certainty responses, see Supplementary M1, Table S7 and Figure S2.

#### Bayesian analysis

Bayesian *t*-tests yielded results that were consistent with the frequentist analysis. We tested for the one-sided alternative hypothesis, $H_1$, that the effects is less than zero. This is a different $H_1$ to that tested in Experiments 1 and 2b, and was chosen based on findings from frequentist analysis which show better memory performance for items belonging to the low-reward category in both phases. For results for the other directional hypothesis, see Supplementary M1 Section C.

For items from the conditioning phase, there was substantial evidence for $H_1$: $BF_{10}$ $=3.16$ with corrected recognition but not true for *d*-prime scores, $BF_{10}$ $=0.57$, which showed anecdotal evidence for the null hypothesis $H_0$. For items encoded in the pre-conditioning phase, there was substantial evidence for $H_1$ showing a retrospective effect of reward category on memory enhancement, in favor of the low-reward category. When considering higher certainty memory responses, however, for items from the conditioning phase, there was substantial evidence for the null hypothesis, Bayes factors less than 0.33 for *d*-prime and corrected recognition, see Supplementary M1, Section B. For items from the pre-conditioning phase, Bayes factors again favored the null hypothesis although the evidence was only anecdotal. Overall, Bayesian results support the frequentist analysis and suggest that reward conditioning was not consistent in the analysis of between corrected recognition, *d*-prime, and when analysing only higher certainty responses, thus questioning the validity of the significant effects found.

### Experiment 2b

#### Overall performance

Matching accuracy was significantly above chance (50%), *p*-values$<0.001$, and did not differ between image category (animal vs. object), $p>0.09$, in both phases. We also confirmed that matching accuracy during the conditioning phase did not differ between high- and low-reward trials, $t_{(59)}=-.26$, $p=0.79$. On the memory test, participants scored an average hit rate of $M=0.75$, $SEM=0.15$, and average false alarm rate of $M=0.17$, $SEM=0.12$, see Supplementary M1, Table S4 for breakdown of responses by certainty.

#### Recognition memory by phase and reward category

The repeated-measures ANOVA showed evidence for an effect of phase on corrected recognition, $F(1,59)=43.63$, $p<0.001$, $\eta ^{2}=0.05$, and on *d*-prime scores, $F(1,59)=42.19$, $p <0.001$, $\eta ^{2}=0.04$, but not reward category. Critically, there was no interaction between reward category and phase on corrected recognition $F(1,59)=0.12$, $p=0.74$, $\eta ^2 <0.001$, nor on *d*-prime scores $F(1,59)=0.34$, $p=0.56$, $\eta ^2<0.001$. The *t*-tests revealed no significant effects of reward category on memory for items encoded in the conditioning phase nor in the post-conditioning phase, see Table 2. The analysis focusing on only higher certainty responses from the memory test was consistent with analysis using all memory trials.

#### Bayesian analysis

 Bayesian analysis with all memory trials as well as only higher certainty responses substantially favored the null hypothesis for items from both phases, with all Bayes factors less than 0.33, see Supplementary M1, Section B. For completeness and in light of the reward category effect appearing in the direction favoring low-reward category for items in the conditioning phase in Experiment 2a, we also report null effects when testing with $H_1$ describing a negative effect of reward category, see Supplementary M1, Section C. Results of generalised linear mixed models reported in Supplementary M1, Section D, also showed no evidence for an effect of reward category on recognition memory for items encoded in the conditioning or post-conditioning phases.

### Discussion

We did not find a consistent memory advantage for items belonging to the high-reward category in Experiment 2a and Experiment 2b. In contrast to the RREM study, Experiment 2a showed preferential memory for items from the low-reward category in the conditioning phase. However this effect was only present when measured with corrected recognition, and was not observed in *d*-prime scores nor in the analysis focusing on higher certainty responses. Experiment 2b showed no difference in recognition memory for items from high- versus low-reward categories encoded during conditioning. In terms of retrospective and prospective memory effects, we did find some evidence in Experiment 2a for category-specific retrospective memory enhancement, again in favour of low-reward category, but the evidence was absent when restricting the analysis to higher certainty responses only. Experiment 2b provided no evidence for prospective memory effects.

Overall, Experiment 2 challenges the robustness of the reward-induced memory enhancement effects reported in the RREM study, which we aimed to closely replicate, when subjected to experimental variations. Furthermore, our results also challenge the generalisability of the reward-induced retrospective memory enhancement effect found in the original study by Patil et al.^6^ that relies on the reward conditioning paradigm. In order to corroborate the effects found in Experiment 2a which were inconsistent across memory measures, and as a final replication attempt of the original RREM study, we conduct Experiment 3 as a high-powered replication of Experiment 2a.

## Experiment 3

### Methods

Procedures and data analysis were identical to Experiment 2a and preregistered at https://osf.io/v564c. Since Experiment 2a did not replicate the expected effect^6^, we adjusted our power calculations for Experiment 3. As per common practice for replications^38^, we halved our effect size from the original $d=0.61$^6^ to $d=0.3$, also reflecting the benchmark for significant memory effects, as per meta-analysis by Klein at al.^39^ We conducted an *a priori* power analysis on G*Power 3.1: a two-tailed paired t-test with $\alpha =0.01$ and $power=0.90$ resulted in $n=169$ participants as a minimum sample size to see an effect of $d=0.3$. We recruited $n=170$ participants for counterbalancing the reward-category assignment, with 83 females and 87 males, aged $M=28.52$, $SEM=0.41$.

### Results

#### Overall performance

Matching accuracy was significantly above chance (50%), *p*-values$<0.001$, and did not differ between image category (animal vs. object), $p>0.05$, in both phases. We also confirmed that matching accuracy during the conditioning phase did not differ between high- and low-reward trials, $t_{(169)}=0.24$, $p=0.81$. On the memory test, participants scored an average hit rate of $M=0.67$, $SEM=0.02$, and average false alarm rate of $M=0.22$, $SEM=0.01$, see Supplementary M1, Table S5 for breakdown of responses by certainty.

#### Recognition memory by phase and reward category

The repeated measures ANOVA revealed no effect of reward category on recognition memory, but there was a strong effect of phase on corrected recognition, $F(1,169)=30.49$, $p <0.001$, $\eta ^{2}=0.02$, and *d*-prime scores $F(1,169)=33.26$, $p <0.001$, $\eta ^{2}=0.015$. There was no significant interaction between encoding phase and reward category associated with the item: $F(1,169)=3,38$, $p=0.07$, $\eta ^{2}=0.001$ for corrected recognition and $F(1,169)=2.23$, $p=0.14$, $\eta ^{2}=0.001$ for *d*-prime scores.

**Figure 5:**
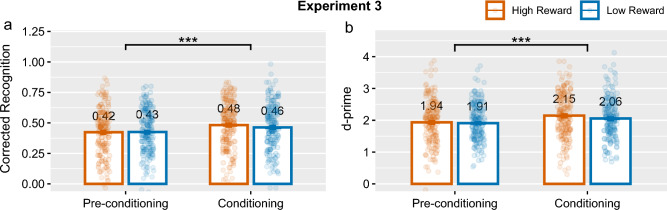
Memory performance measured by corrected recognition (left) and *d*-primes (right) in Experiment 3 by phase and reward category. Group means are displayed with error bars showing $\pm 1$ *SEM*. *$p < 0.05$, **$p < 0.01$, ***$p < 0.001$.

For items in the conditioning phase, we observed a trend showing enhanced memory for items in the high-reward category as found in the RREM study, however paired *t*-tests revealed that the effect was only at trend level and not significant: $t(169)=1.61$, $p=0.11$, $d_{av}=0.10$ for corrected recognition, and $t(169)=1.79$, $p=0.08$, $d_{av}=0.12$ for *d*-prime, see Fig. 5. This effect was not seen in the analysis focusing on higher certainty responses, see Supplementary M1, Section B. In the pre-conditioning phase, the effect of reward category was not significant, *p*-values$>.0.55$.

#### Bayesian analysis

Bayesian *t*-tests yielded results that were consistent with the frequentist analysis suggesting that any effects seen were only at trend level and insignificant. In the conditioning phase, there was anecdotal evidence for the null hypothesis $H_0$. While in the pre-conditioning phase, the evidence for the null hypothesis was substantial, see Table 3. Results of generalised linear mixed models reported in Supplementary M1, Section D, also showed no significant evidence for an effect of reward category on recognition memory for items encoded in the conditioning or post-conditioning phases.

**Table 3:** Summary of *t*-tests in Experiment 3.

		Frequentist *t*-test			Bayesian *t*-test	
		t(169)	*p*	${d_{av}}$	${BF_{10}}$	Interpretation
Pre-conditioning	CR	− 0.15	0.88	− 0.01	0.08	Substantial evidence for $H_0$
	DP	0.60	0.55	0.04	0.15	Substantial evidence for $H_0$
Conditioning	CR	1.61	0.11	0.10	0.57	Anecdotal evidence for $H_0$
	DP	1.79	0.08$\dagger$	0.12	0.78	Anecdotal evidence for $H_0$

Frequentists paired *t*-tests for effect of reward category (high vs. low) on memory annotated with: $\dagger$ $p< 0.1$, *$p< 0.05$, **$p< 0.01$, ***$p< 0.001$. Bayesian *t*-test $H_1$: one sided hypothesis that effect of reward is greater than zero, i.e. better memory for items from the high-reward category, $H_0$: no effect of reward category. Bayes factors $\mathbf {BF_{10}}$ are interpreted as providing substantial or anecdotal evidence in favor of $H_1$ or $H_0$. CR, corrected recognition; DP, *d*-primes.

### Discussion

We did not find a significant memory advantage for items belonging to the high-reward semantic category in Experiment 3 despite the higher sample size used in this experiment. While there was a trend of increased memory for items the high-reward category, this effect was not significant and not seen in the analysis of higher certainty responses.

## General discussion

Across four experiments, we tested whether pairing items from a specific semantic category with high- versus low-reward value results in preferential memory for all items from the semantic category paired with high-reward. We did not find conclusive evidence for this effect. Experiment 1 indicated some evidence of an effect of reward category in the conditioning phase in favor of the high-reward category. Experiment 2a showed an effect of reward in favor of the low-reward category, while a trend level effect in favor of the high-reward category was seen in 3. Experiment 2b showed no significant effects. Any effects of reward category on category-specific memory were either inconsistent across *d*-prime scores and corrected recognition, or diminished with higher-certainty analysis.

Only Experiment 2 showed some evidence for the proposed category-specific retrospective memory enhancement effect reported in Patil et al.^6^, albeit in the direction favoring low reward. However, this effect became insignificant when evaluated with *d*-primes and in the analysis focusing on higher certainty responses. Overall, the variable effects of reward category observed in the conditioning phases of our experiments precludes any conclusions about the absence of selective retrospective or prospective memory effects. Critically, our results demonstrate the instability of the particular reward-conditioning protocol in inducing category-specific memory improvements, even during the direct conditioning phase. Therefore, while our results do not negate the original experimental result reported by Patil et al.^6^, they do call into question their generalisability. Despite some limitations and differences from the original study listed below, our study is an important first step towards characterising the generalisability of this effect; and provides several considerations for future research aiming to investigate or use a similar conditioning paradigm to probe adaptive memory.

Although our experiments resulted in ambiguous evidence for memory enhancement of items from high- versus low-reward categories, we did find a strong and significant memory advantage for all items (i.e. from both high- and low-reward categories) in the reward-conditioning phase compared to the non-conditioning phases in Experiments 2a, 2b and 3. This suggests that incentivising with monetary rewards does have preferential effects on memory but there was no marked memory difference between items paired with high- and low-reward values. Our study highlights the variability in memory advantages resulting from reward-value conditioning and contributes to the reward-memory literature that have previously reported similar inconsistencies in the effect^40–43^. Factors such as the difference between reward anticipation and outcome, reward value, surprisal and uncertainty and how these are associated with the stimuli, are all shown to be mediators in the overall effects on recognition memory. While association with higher reward magnitude regardless of positive or negative valence^43^ and a positive difference between reward outcome and expectation has been found to enhance memory^23^, the effects of general surprisal and uncertainty^44^ are less clear.

It is also important to differentiate intentional and incidental learning studies. In intentional learning studies, participants are explicitly told that rewards are dependent on their memory performance and generally report a stronger reward-memory relationship^24,45^. The RREM study utilised incidental learning and still reported a strong effect of reward on recognition memory. Other incidental learning studies, such as our current study, exploring reward based memory effects without the explicit motivation have not only failed to quantify the effect, but have also found contradicting evidence showing memory enhancements for lower (as opposed to higher) reward outcomes^42,46^.

It has been argued that in studies exploring the reward-memory relationship, high-reward outcomes can have an undesired negative effect due to heightened performance anxiety that results in detriment of attention and cognitive control. Such interfering effects due to performance anxiety while completing a study could also lead to expending excessive attention to details of a task and thus reducing the attention directed toward the intended task^47–49^. This factor is especially a concern for Experiment 1 which involves a more complex incidental encoding task compared to previous studies. Yet, if there were considerable effects of attention and anxiety influencing encoding and adaptive memory effects in our experiments, it remains unclear as to why this was not a confounding factor in the RREM study.

Another factor that could have influenced memory that we did not control for was stimulus typicality. Studies using the same stimulus set as the RREM study^6^, originally used by Dunsmoor et al.^7^ have found that there was a general higher rating of typicality for animal images^28^. Although this was not controlled in the original RREM study nor in our study, for Experiments 2 and 3, it is unlikely to be a problem because stimuli were randomly allocated to encoding phases and high reward categories were counterbalanced, future studies should control for this factor to avoid any possibility of spurious memory effects. Future studies should also attempt to replicate these effects with image stimuli from other semantic categories, for example outdoor versus indoor scenes, to avoid potential biases specific to stimuli.

Most of the observed effects in our experiments had discrepancies between analyses based on *d*-prime and corrected recognition. While the RREM study only reported corrected recognition, we additionally report *d*-prime scores. We find that across Experiments 1 and 2a, any effects of reward-category on recognition memory were seen in corrected recognition but not in the purportedly more stringent *d*-prime measure. Similar discrepancies were also found in a replication^28^ of a fear-conditioning study^7^ reporting similar category-specific retrospective memory enhancement effects. The two memory measures rely on different models of recognition memory: corrected recognition adopts a threshold model^50,51^ and d-prime is based on signal detection theory (SDT) which assumes a curved receiver operating curve (ROC). While a discussion of the specific theory behind these models is beyond the scope of this paper^26^, most empirical studies, especially in memory and perception research, argue that SDT is the preferred method of accounting for the independent contributions of memory sensitivity and response bias to recognition memory^29,31^. While we cannot fully explain the discrepancies between measures of memory, the differences may point to other factors, such as response biases, that influence memory and require further study; we urge future work exploring adaptive memory and reward-memory effects to report both corrected recognition and d-primes.

Our experiments had some differences in protocol compared to the RREM study. While Experiment 1 has a more complex incidental learning paradigm, and may not by directly comparable to the RREM study, our Experiments 2a, 2b and 3 are very close replications. However, our experiments deviate from the RREM study in that we used smaller monetary incentives with the same ratio as the RREM study: in Experiment 2, we used $$\pounds$$5 as opposed to $20 for high reward, and $$\pounds$$0.25 as opposed to $1 for low reward. There is evidence to suggest reward value has a non-linear effect on memory^41,44^, and it could be that the dopaminergic neuromodulation required to induce a reward effect was not differential enough for the values $$\pounds$$5 and $$\pounds$$0.25. Another difference between our study and the RREM study was that we conducted our experiments online on prolific.co. However, if using online studies and reducing the monetary reward value renders the reward conditioning effects on recognition memory void, this challenges the robustness of this particular reward conditioning paradigm and the generalisability of the reward-induced category-specific and retrospective memory effects^6^.

## Conclusion

The hypothesis that rewards can selectively and retrospectively improve memory neatly ties the neurobiological underpinnings of dopaminergic modulation^52^ within the hippocampus^53^, to the behavioral advantages of prioritising significant memories. However, robust evidence for this effect is limited^6^ and variable across studies including our own^12,13, 54^. We consider several theoretical reasons for this variability. It is well-established that dopaminergic modulation by rewards can enhance hippocampal memory formation for items presented in similar contexts^55^. Memory improvements for items from the high-reward category^6^ may be explained by strengthened interaction between the hippocampus and the rewarded category-selective visual cortex^3,56, 57^. One explanation for null category-specific memory effects in our study could be that the semantic category was not strongly associated to high or low reward, instead it was indicated by a peripheral feature of the stimuli, namely green/grey color of stars, leading to unsuccessful reward and memory modulation. In fact, Wittman et al.^41^ show that explicit semantic categorisation of stimuli, and link to rewards, is required to modulate memory. The additional task of explicitly identifying the item category (animal/object) during encoding^7,13^, might be crucial to strengthen this association. Successful reward modulation is further complicated by the variable effect of reward value on memory; neuroimaging studies have shown a lack of dopamine activity during passive monetary reward tasks^58^. Future studies should consider strengthening the reward conditioning paradigm and incorporating an active task that associates items with their semantic and reward categories.

The absence of selective memory effects in studies by Oyarzún et al.^13^ and our own could also be due to the reliance on an item recognition task that does not heavily engage the hippocampus, as recognition does not require retrieving contextually rich memories^36,37, 59–61^. Conversely, a rewarded spatial exploration task which directly probes hippocampus-dependent spatial memory did show a strong retrospective reward effect^5^. However, it remains unclear why the item recognition task design in the RREM study was sufficient to observe the effect. Future research should investigate how selective memory effects vary with tasks assessing different memory systems.

Overall, although there is substantial evidence for the neural basis of adaptive memory as per the synaptic tag-and-capture hypothesis, consistent behavioural evidence for this phenomenon only shows general memory enhancement due to temporal and spatial closeness to salient events^4,5^. The highly specific memory enhancement effect for conceptually related stimuli^6^ was not replicated in our experiments using the same reward-conditioning paradigm. The inconsistent results for selective memory effects due to semantic categorisation suggest its minor role in human behaviour, while the stronger effects linked to temporal^4^ and spatial^5^ proximity to rewards indicate a more substantial role in informing future behaviour.

Our study also highlights the variability in the influence of reward value on recognition memory. Although fear and reward processing rely on different processes, the same highly specific retrospective enhancement effect has suffered non-replication in the fear-conditioning domain as well^12,28^. It is thus important that studies explore the underlying phenomenon of adaptive memory with a wider range and possibly stronger methods of conditioning which also influence hippocampal-dependent memory consolidation processes such as reward prediction error, novelty, emotion or stress.

### Supplementary Information

Supplementary Information.

## Data Availability

The code for the experiments, stimuli and raw data are publicly available at https://github.com/prisukumaran23/adaptiveMemoryReplication.
