# Supplementary material for: Reward conditioning may not have an effect on category-specific memory
Source: Sci Rep. 2023 Dec 15;13:22297. doi: 10.1038/s41598-023-48874-z (PMC10724210; doi:10.1038/s41598-023-48874-z)
Supplement: Supplementary file 1 — Supplementary Information. [file 41598_2023_48874_MOESM1_ESM.pdf]

# Supplementary Material 1 (M1): Reward conditioning may not have an effect on category-specific memory

**Priyanka Sukumaran[1,2,*], Nina Kazanina[2,3,+], and Conor Houghton[1,+]**

[1]Faculty of Engineering, University of Bristol, Bristol, BS8 1UB, United Kingdom
[2]School of Psychological Sciences, University of Bristol, Bristol, BS8 1TU, United Kingdom
[3]International Laboratory of Social Neurobiology, HSE University, Moscow, Russia
[*]corresponding author: p.sukumaran@bristol.ac.uk
[+]These authors contributed equally

## A. Performance Summary

**Table S1.** Summary of Performance on Memory Tests

| Experiment | CR | d' |
|---|---|---|
| All Memory | | |
| 1 | 0.466 ± 0.010 | 2.224 ± 0.039 |
| 2a | 0.496 ± 0.024 | 2.128 ± 0.083 |
| 2b | 0.455 ± 0.024 | 1.980 ± 0.084 |
| 3 | 0.449 ± 0.007 | 2.012 ± 0.028 |
| High Certainty | | |
| 1 | 0.527 0.010 ± | 2.592 ± 0.042 |
| 2a | 0.580 ± 0.025 | 2.550 ± 0.108 |
| 2b | 0.579 ± 0.024 | 2.513 ± 0.101 |
| 3 | 0.571 ± 0.010 | 2.632 ± 0.039 |

*Note:* Mean and standard errors of CR = corrected recognition; d' = d-prime.

**Table S2.** Experiment **1**, Mean Proportion of Memory Responses by Certainty

| | High Reward | | | | | |
|---|---|---|---|---|---|---|
| Measure | DO | LO | MO | MN | LN | DN |
| Pre-conditioning | 0.290 | 0.182 | 0.158 | 0.170 | 0.183 | 0.259 |
| Conditioning | 0.272 | 0.181 | 0.152 | 0.187 | 0.191 | 0.276 |
| Post-conditioning | 0.272 | 0.160 | 0.153 | 0.180 | 0.206 | 0.279 |
| New | 0.077 | 0.083 | 0.094 | 0.171 | 0.256 | 0.424 |
| | Low Reward | | | | | |
| Measure | DO | LO | MO | MN | LN | DN |
| Pre-conditioning | 0.297 | 0.170 | 0.172 | 0.160 | 0.191 | 0.256 |
| Conditioning | 0.251 | 0.139 | 0.151 | 0.179 | 0.223 | 0.289 |
| Post-conditioning | 0.275 | 0.159 | 0.156 | 0.190 | 0.208 | 0.256 |
| New | 0.068 | 0.077 | 0.096 | 0.167 | 0.252 | 0.434 |

*Note:* DO = definitely old; LO = likely old; MO = maybe old; MN = maybe new; LN = likely new, DN = definitely new.

**Table S3.** Experiment **2a**, Mean Proportion of Memory Responses by Certainty

| | High Reward | | | | | |
|---|---|---|---|---|---|---|
| Measure | DO | LO | MO | MN | LN | DN |
| Pre-conditioning | 0.407 | 0.145 | 0.136 | 0.161 | 0.144 | 0.117 |
| Conditioning | 0.452 | 0.141 | 0.157 | 0.146 | 0.119 | 0.114 |
| New | 0.058 | 0.074 | 0.107 | 0.235 | 0.246 | 0.322 |
| | Low Reward | | | | | |
| Measure | DO | LO | MO | MN | LN | DN |
| Pre-conditioning | 0.404 | 0.180 | 0.141 | 0.141 | 0.138 | 0.107 |
| Conditioning | 0.451 | 0.171 | 0.149 | 0.128 | 0.121 | 0.089 |
| New | 0.054 | 0.064 | 0.107 | 0.235 | 0.273 | 0.323 |

*Note:* DO = definitely old; LO = likely old; MO = maybe old; MN = maybe new; LN = likely new, DN = definitely new.

**Table S4.** Experiment **2b**, Mean Proportion of Memory Responses by Certainty

| | High Reward | | | | | |
|---|---|---|---|---|---|---|
| Measure | DO | LO | MO | MN | LN | DN |
| Conditioning | 0.418 | 0.187 | 0.155 | 0.131 | 0.117 | 0.104 |
| Post-conditioning | 0.332 | 0.172 | 0.171 | 0.171 | 0.139 | 0.130 |
| New | 0.050 | 0.075 | 0.139 | 0.245 | 0.300 | 0.264 |
| | Low Reward | | | | | |
| Measure | DO | LO | MO | MN | LN | DN |
| Conditioning | 0.423 | 0.200 | 0.129 | 0.158 | 0.112 | 0.109 |
| Post-conditioning | 0.288 | 0.202 | 0.163 | 0.182 | 0.140 | 0.136 |
| New | 0.048 | 0.084 | 0.109 | 0.255 | 0.291 | 0.271 |

*Note:* DO = definitely old; LO = likely old; MO = maybe old; MN = maybe new; LN = likely new, DN = definitely new.

**Table S5.** Experiment **3**, Mean Proportion of Memory Responses by Certainty

| | High Reward | | | | | |
|---|---|---|---|---|---|---|
| Measure | DO | LO | MO | MN | LN | DN |
| Pre-conditioning | 0.319 | 0.183 | 0.163 | 0.195 | 0.128 | 0.105 |
| Conditioning | 0.372 | 0.190 | 0.159 | 0.165 | 0.127 | 0.080 |
| New | 0.058 | 0.081 | 0.122 | 0.279 | 0.301 | 0.219 |
| | Low Reward | | | | | |
| Measure | DO | LO | MO | MN | LN | DN |
| Pre-conditioning | 0.325 | 0.186 | 0.160 | 0.192 | 0.127 | 0.109 |
| Conditioning | 0.360 | 0.181 | 0.159 | 0.172 | 0.126 | 0.100 |
| New | 0.060 | 0.080 | 0.120 | 0.281 | 0.292 | 0.240 |

*Note:* DO = definitely old; LO = likely old; MO = maybe old; MN = maybe new; LN = likely new, DN = definitely new.

## B. Analysis of Higher Certainty Responses

| | | Frequentist $t$-test | | | Bayesian $t$-test | |
|---|---|---|---|---|---|---|
| | | **t(119)** | **p** | **dav** | **B10** | **Note** |
| **Pre** | **CR** | -0.56 | 0.58 | -0.05 | 0.07 | Sub $H_0$ |
| | **DP** | -0.67 | 0.51 | -0.06 | 0.06 | Sub $H_0$ |
| **Cond** | **CR** | 1.65 | 0.10 | 0.20 | 0.72 | Anec $H_0$ |
| | **DP** | 1.14 | 0.26 | 0.13 | 0.32 | Sub $H_0$ |
| **Post** | **CR** | -0.72 | 0.48 | -0.07 | 0.06 | Sub $H_0$ |
| | **DP** | -0.95 | 0.35 | -0.10 | 0.06 | Sub $H_0$ |

**Table S6.** Summary of $t$-tests of higher certainty responses in Experiment **1**. Frequentist paired $t$-test for effect of reward category (high vs. low) on memory annotated with: † $p < 0.1$, * $p < 0.05$, ** $p < 0.01$, *** $p < 0.001$. Bayesian $t$-test $H_1$: one sided hypothesis that effect of reward is greater than zero, i.e. better memory for items in the high-reward category, $H_0$: no effect of reward category. Bayes factors are interpreted as Sub: substantial evidence and Anec: anecdotal evidence in favor of $H_1$ or $H_0$.

| | | Frequentist $t$-test | | | Bayesian $t$-test | |
|---|---|---|---|---|---|---|
| | | **t(59)** | **p** | **dav** | **B10** | **Note** |
| **Exp 2a** | | | | | | |
| **Pre** | **CR** | -1.62 | 0.11 | -0.19 | 0.91 | Anec $H_0$ |
| | **DP** | -1.32 | 0.19 | -0.13 | 0.58 | Anec $H_0$ |
| **Cond** | **CR** | -0.64 | 0.52 | -0.07 | 0.25 | Sub $H_0$ |
| | **DP** | 0.44 | 0.66 | 0.04 | 0.10 | Sub $H_0$ |
| **Exp 2b** | | | | | | |
| **Cond** | **CR** | 0.13 | 0.90 | 0.01 | 0.16 | Sub $H_0$ |
| | **DP** | 0.13 | 0.90 | 0.01 | 0.16 | Sub $H_0$ |
| **Post** | **CR** | 0.80 | 0.43 | 0.08 | 0.30 | Sub $H_0$ |
| | **DP** | 0.31 | 0.75 | 0.03 | 0.18 | Sub $H_0$ |

**Table S7.** Summary of $t$-tests of higher certainty responses in Experiment **2a** and **2b**. Frequentist paired $t$-test for effect of reward category (high vs. low) on memory annotated with: † $p < 0.1$, * $p < 0.05$, ** $p < 0.01$, *** $p < 0.001$. Bayesian $t$-test $H_1$: one sided hypothesis that effect of reward is greater than zero, i.e. better memory for items in the high-reward category, $H_0$: no effect of reward category. Bayes factors are interpreted as Sub: substantial evidence and Anec: anecdotal evidence in favor of $H_1$ or $H_0$.

| | | Frequentist $t$-test | | | Bayesian $t$-test | |
|---|---|---|---|---|---|---|
| | | **t(169)** | **p** | **dav** | **B10** | **Note** |
| **Pre** | **CR** | 0.16 | 0.87 | 0.01 | 0.10 | Sub $H_0$ |
| | **DP** | 0.32 | 0.75 | 0.02 | 0.11 | Sub $H_0$ |
| **Cond** | **CR** | 0.96 | 0.34 | 0.06 | 0.22 | Sub $H_0$ |
| | **DP** | 0.38 | 0.71 | 0.02 | 0.12 | Sub $H_0$ |

**Table S8.** Summary of $t$-tests of higher certainty responses in Experiment **3**. Frequentist paired $t$-test for effect of reward category (high vs. low) on memory annotated with: † $p < 0.1$, * $p < 0.05$, ** $p < 0.01$, *** $p < 0.001$. Bayesian $t$-test $H_1$: one sided hypothesis that effect of reward is greater than zero, i.e. better memory for items in the high-reward category, $H_0$: no effect of reward category. Bayes factors are interpreted as Sub: substantial evidence and Anec: anecdotal evidence in favor of $H_1$ or $H_0$.

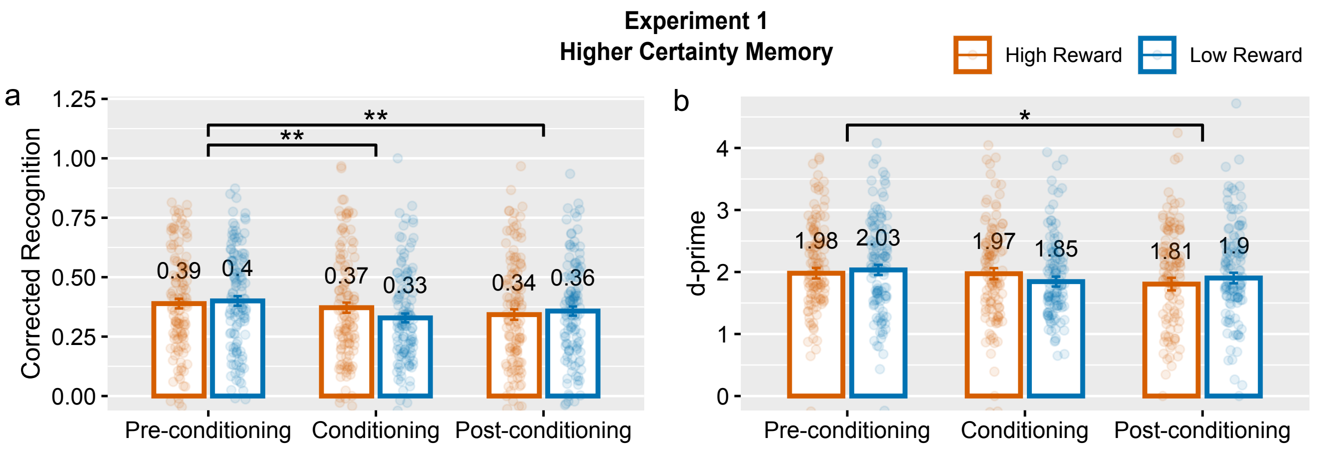


**Figure S1.** *Performance filtered by high certainty memory trials on memory task in Experiment **1** by phase and reward category.* Corrected recognition (left) and *d*-primes (right) by phase and reward category. Group means are labelled and error bars represent ±1 *SEM*. *** $p < 0.001$, ** $p < 0.01$, * $p < 0.05$.

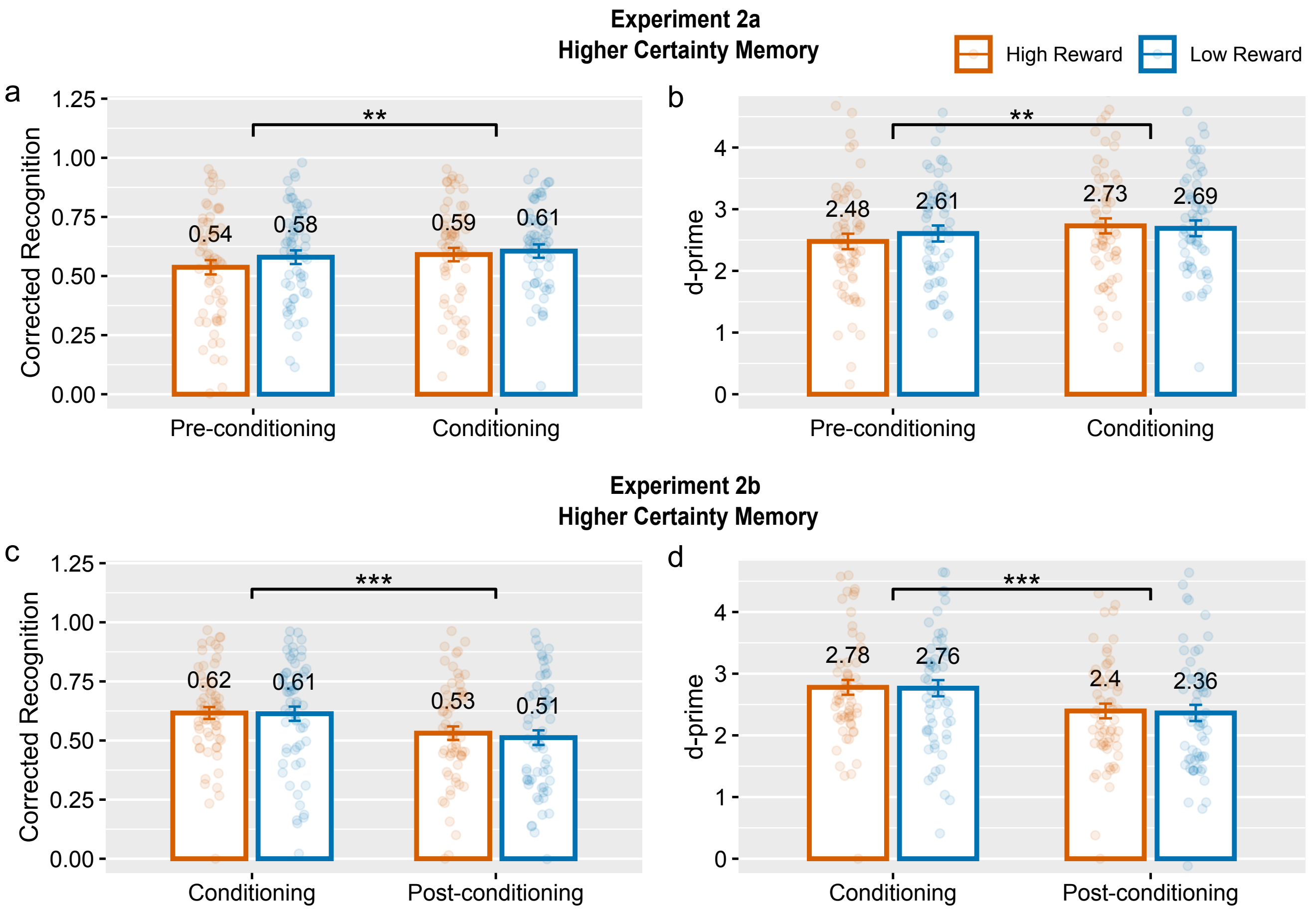


**Figure S2.** *Performance filtered by high certainty memory trials on memory task in Experiment **2a** and **2b** by phase and reward category.* Corrected recognition (left) and *d*-primes (right) by phase and reward category. Group means are labelled and error bars represent ±1 *SEM*. *** $p < 0.001$, ** $p < 0.01$, * $p < 0.05$.

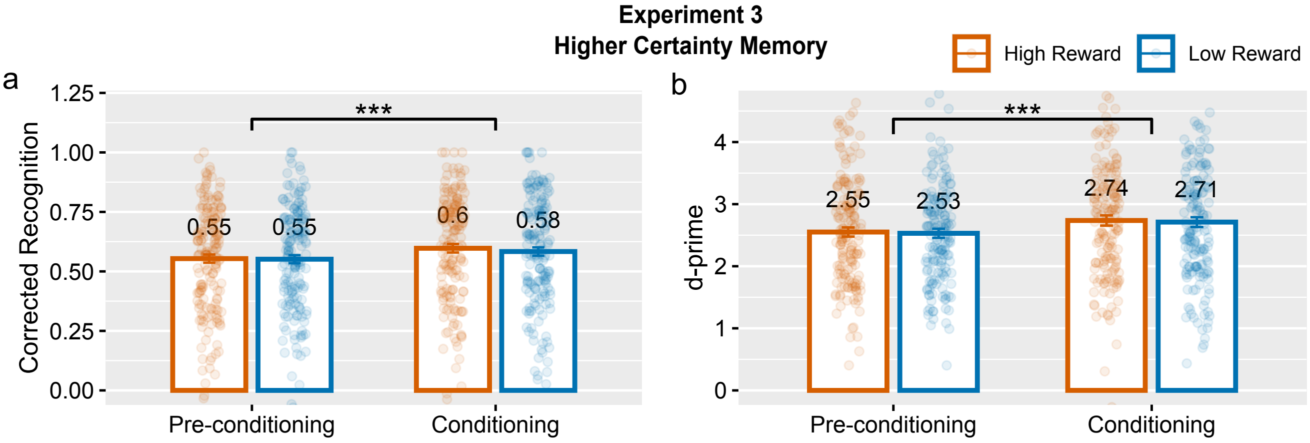


**Figure S3.** *Performance filtered by high certainty memory trials on memory task in Experiment **3** by phase and reward category.* Corrected recognition (left) and *d*-primes (right) by phase and reward category. Group means are labelled and error bars represent ±1 *SEM*. *** $p < 0.001$, ** $p < 0.01$, * $p < 0.05$.

# C. Additional Bayesian t-tests

**Table S9.** Summary of additional Bayesian t-tests in Experiment **2a**

| | | Bayesian t-test B1+0 | Note | | | Bayesian t-test B1+0 | Note |
|---|---|---|---|---|---|---|---|
| **Exp 2a All Memory** | | | | **Exp 2a High Certainty** | | | |
| **Pre** | **CR** | 0.03 | Sub H0 | **Pre** | **CR** | 0.06 | Sub H0 |
| | **DP** | 0.04 | Sub H0 | | **DP** | 0.07 | Sub H0 |
| **Cond** | **CR** | 0.05 | Sub H0 | **Cond** | **CR** | 0.09 | Sub H0 |
| | **DP** | 0.07 | Sub H0 | | **DP** | 0.21 | Sub H0 |

*Note:* H1+: One sided hypothesis that effect of reward is greater than zero, i.e. items in high reward category are enhanced in memory, H0: No effect of reward category. The result is described in the note section as Sub: substantial evidence and Anec: anecdotal evidence in favor of H1 or H0.

**Table S10.** Summary of additional Bayesian t-tests in Experiment **2b**

| | | Bayesian t-test B1-0 | Note | | | Bayesian t-test B1-0 | Note |
|---|---|---|---|---|---|---|---|
| **Exp 2b All Memory** | | | | **Exp 2b High Certainty** | | | |
| **Cond** | **CR** | 0.09 | Sub H0 | **Cond** | **CR** | 0.16 | Sub H0 |
| | **DP** | 0.09 | Sub H0 | | **DP** | 0.16 | Sub H0 |
| **Post** | **CR** | 0.11 | Sub H0 | **Post** | **CR** | 0.29 | Sub H0 |
| | **DP** | 0.13 | Sub H0 | | **DP** | 0.18 | Sub H0 |

*Note:* H1-: One sided hypothesis that effect of reward is less than zero, i.e. items in low reward category are enhanced in memory, H0: No effect of reward category. The result is described in the note section as Sub: substantial evidence and Anec: anecdotal evidence in favor of H1 or H0.

## D. Linear Mixed-Effects Models

In the supplemental materials, we present results of applying a generalized linear mixed-effect model to the categorical response data from the memory test.

**Table S11.** Experiment **1** (All Memory)

| Fixed Effects | Estimate (Log Odds) | SE | 95% CI Lower | 95% CI Upper | p |
|---|---|---|---|---|---|
| Intercept | -1.55 | 0.47 | -2.49 | -0.45 | < 0.001 |
| Phase 1 | 1.70 | 0.47 | 0.56 | 2.66 | < 0.001 |
| Phase 2 | 1.38 | 0.47 | 0.25 | 2.27 | < 0.001 |
| Phase 3 | 1.52 | 0.47 | 0.34 | 2.39 | < 0.001 |
| Reward | 0.04 | 0.05 | -0.06 | 0.15 | 0.34 |
| Phase 1 : Reward | -0.04 | 0.08 | -0.25 | 0.13 | 1.28 |
| Phase 2 : Reward | 0.17 | 0.08 | -0.03 | 0.33 | 0.08 |
| Phase 3 : Reward | -0.04 | 0.08 | -0.21 | 0.15 | 0.66 |
| | Variance | SD | | | |
| Stim (Intercept) | 0.38 | 0.62 | | | |
| UserID (Intercept) | 0.22 | 0.47 | | | |

**Table S12.** Experiment **1** (High Certainty Memory)

| Fixed Effects | Estimate (Log Odds) | SE | 95% CI Lower | 95% CI Upper | p |
|---|---|---|---|---|---|
| Intercept | -1.96 | 0.58 | -2.86 | -0.52 | < 0.001 |
| Phase 1 | 2.18 | 0.58 | 0.79 | 3.17 | < 0.001 |
| Phase 2 | 1.81 | 0.58 | 0.40 | 2.73 | < 0.001 |
| Phase 3 | 1.99 | 0.58 | 0.64 | 3.03 | < 0.001 |
| Reward | 0.17 | 0.06 | 0.05 | 0.31 | < 0.001 |
| Phase 1 : Reward | -0.11 | 0.10 | -0.27 | 0.08 | 0.18 |
| Phase 2 : Reward | 0.17 | 0.10 | -0.001 | 0.43 | 0.12 |
| Phase 3 : Reward | -0.12 | 0.10 | -0.32 | 0.07 | 0.16 |
| | Variance | SD | | | |
| Stim (Intercept) | 0.62 | 0.79 | | | |
| UserID (Intercept) | 0.34 | 0.58 | | | |

**Table S13.** Experiment **2a** (All Memory)

| Fixed Effects | Estimate (Log Odds) | SE | 95% CI Lower | 95% CI Upper | p |
|---|---|---|---|---|---|
| Intercept | -1.63 | 0.10 | -1.85 | -1.38 | < 0.001 |
| Phase 1 | 2.60 | 0.11 | 2.39 | 2.86 | < 0.001 |
| Phase 2 | 2.77 | 0.11 | 2.51 | 2.99 | < 0.001 |
| Reward | 0.12 | 0.06 | 0.01 | 0.24 | < 0.001 |
| Phase 1 : Reward | -0.36 | 0.10 | -0.59 | -0.16 | < 0.001 |
| Phase 2 : Reward | -0.27 | 0.10 | -0.48 | -0.08 | 0.02 |
| **Random Effects** | **Variance** | **SD** | | | |
| Stim (Intercept) | 0.39 | 0.62 | | | |
| UserID (Intercept) | 0.28 | 0.53 | | | |

**Table S14.** Experiment **2a** (High Certainty Memory)

| Fixed Effects | Estimate (Log Odds) | SE | 95% CI Lower | 95% CI Upper | p |
|---|---|---|---|---|---|
| Intercept | -2.08 | 0.15 | -2.37 | -1.82 | < 0.001 |
| Phase 1 | 3.44 | 0.15 | 3.23 | 3.75 | < 0.001 |
| Phase 2 | 3.63 | 0.15 | 3.34 | 3.92 | < 0.001 |
| Reward | 0.15 | 0.09 | 0.031 | 0.32 | 0.04 |
| Phase 1 : Reward | -0.32 | 0.13 | -0.58 | -0.13 | 0.02 |
| Phase 2 : Reward | -0.22 | 0.13 | -0.46 | 0.07 | 0.06 |
| **Random Effects** | **Variance** | **SD** | | | |
| Stim (Intercept) | 0.71 | 0.84 | | | |
| UserID (Intercept) | 0.66 | 0.81 | | | |

**Table S15.** Experiment **2b** (All Memory)

| Fixed Effects | Estimate (Log Odds) | SE | 95% CI Lower | 95% CI Upper | p |
|---|---|---|---|---|---|
| Intercept | -1.49 | 0.10 | -1.71 | -1.32 | < 0.001 |
| Phase 1 | 2.53 | 0.11 | 2.33 | 2.74 | < 0.001 |
| Phase 2 | 2.07 | 0.11 | 1.88 | 2.27 | < 0.001 |
| Reward | 0.06 | 0.06 | -0.06 | 0.16 | 0.34 |
| Phase 1 : Reward | 0.06 | 0.10 | -0.14 | 0.24 | 0.58 |
| Phase 2 : Reward | 0.003 | 0.10 | -0.18 | 0.22 | 0.94 |
| **Random Effects** | **Variance** | **SD** | | | |
| Stim (Intercept) | 0.43 | 0.65 | | | |
| UserID (Intercept) | 0.21 | 0.46 | | | |

**Table S16.** Experiment **2b** (High Certainty Memory)

| Fixed Effects | Estimate (Log Odds) | SE | 95% CI Lower | 95% CI Upper | p |
|---|---|---|---|---|---|
| Intercept | -1.77 | 0.13 | -2.07 | -1.55 | < 0.001 |
| Phase 1 | 3.42 | 0.15 | 3.17 | 3.75 | < 0.001 |
| Phase 2 | 2.78 | 0.14 | 2.52 | 3.06 | < 0.001 |
| Reward | -0.16 | 0.09 | -0.30 | -0.002 | 0.04 |
| Phase 1 : Reward | 0.07 | 0.14 | -0.21 | 0.37 | 0.44 |
| Phase 2 : Reward | 0.17 | 0.13 | -0.09 | 0.41 | 0.18 |

| Random Effects | Variance | SD |
|---|---|---|
| Stim (Intercept) | 0.67 | 0.82 |
| UserID (Intercept) | 0.42 | 0.65 |

**Table S17.** Experiment **3** (All Memory)

| Fixed Effects | Estimate (Log Odds) | SE | 95% CI Lower | 95% CI Upper | p |
|---|---|---|---|---|---|
| Intercept | -1.48 | 0.08 | -1.63 | -1.33 | < 0.001 |
| Phase 1 | 2.16 | 0.11 | 1.97 | 2.33 | < 0.001 |
| Phase 2 | 2.37 | 0.11 | 2.17 | 2.52 | < 0.001 |
| Reward | 0.02 | 0.06 | -0.05 | 0.10 | 0.58 |
| Phase 1 : Reward | -0.02 | 0.10 | -0.12 | 0.11 | 0.74 |
| Phase 2 : Reward | 0.01 | 0.10 | -0.01 | 0.22 | 0.09 |

| Random Effects | Variance | SD |
|---|---|---|
| Stim (Intercept) | 0.42 | 0.65 |
| UserID (Intercept) | 0.31 | 0.56 |

**Table S18.** Experiment **3** (High Certainty Memory)

| Fixed Effects | Estimate (Log Odds) | SE | 95% CI Lower | 95% CI Upper | p |
|---|---|---|---|---|---|
| Intercept | -1.83 | 0.12 | -2.05 | -1.60 | < 0.001 |
| Phase 1 | 3.06 | 0.11 | 2.80 | 3.37 | < 0.001 |
| Phase 2 | 3.31 | 0.11 | 3.03 | 3.64 | < 0.001 |
| Reward | 0.05 | 0.06 | -0.07 | 0.15 | 0.40 |
| Phase 1 : Reward | 0.001 | -0.08 | -0.15 | 0.16 | 1.00 |
| Phase 2 : Reward | 0.08 | -0.09 | -0.09 | 0.24 | 0.34 |

| Random Effects | Variance | SD |
|---|---|---|
| Stim (Intercept) | 0.73 | 0.86 |
| UserID (Intercept) | 0.68 | 0.83 |

# E. Survey Questions

### Experiment 1 Survey Questions

- Did you have any knowledge of Japanese beforehand?
- What strategy did you use for the first memory test?
- What strategy did you use for the second memory test?
- Please rate your sleep quality last night from 1 (very bad) to 5 (very good)
- Did you notice that there were two categories of images (animals and objects)?
- Did you notice which category was rewarded with a higher bonus in phase 2?

### Experiment 2 Survey Questions

- How surprised were you by the memory test?
- What strategy did you use for the memory test?
- Please rate your sleep quality last night from 1 (very bad) to 5 (very good)
- Did you notice that there were two categories of images (animals and objects)?
- Did you notice which category was rewarded with a higher bonus from day 1?

# F. Results of Experiment 1 - Immediate Memory Test

In addition to Experiment 1 reported in the main article, which had a 24-hour delayed memory test, we also performed a version of Experiment 1 testing immediate memory retrieval. The encoding protocol was identical to Experiment 1 with three phases: pre-conditioning, conditioning and post-conditioning. We then probed immediate memory retrieval by administering a surprise recognition memory test approximately three minutes after the encoding phases, analogous to Experiment 2 in the Patil et al. study[1]. We recruited 123 participants for the experiment, and the final 120 participants after exclusions included 79 females, 40 males, one with undisclosed gender, with an average age of $M = 26.80$, $SEM = 0.48$.

**Table S19.** Experiment **1-Immediate Memory**, Mean Proportion of Memory Responses by Certainty

| | High Reward | | | | | |
|---|---|---|---|---|---|---|
| Measure | DO | LO | MO | MN | LN | DN |
| Pre-conditioning | 0.438 | 0.153 | 0.174 | 0.150 | 0.155 | 0.272 |
| Conditioning | 0.428 | 0.164 | 0.153 | 0.181 | 0.161 | 0.258 |
| Post-conditioning | 0.450 | 0.153 | 0.146 | 0.178 | 0.160 | 0.242 |
| New | 0.051 | 0.065 | 0.082 | 0.173 | 0.221 | 0.563 |
| | Low Reward | | | | | |
| Measure | DO | LO | MO | MN | LN | DN |
| Pre-conditioning | 0.428 | 0.146 | 0.136 | 0.159 | 0.167 | 0.258 |
| Conditioning | 0.400 | 0.139 | 0.151 | 0.179 | 0.223 | 0.289 |
| Post-conditioning | 0.419 | 0.145 | 0.137 | 0.177 | 0.177 | 0.267 |
| New | 0.053 | 0.068 | 0.094 | 0.151 | 0.206 | 0.555 |

*Note:* DO = definitely old; LO = likely old; MO = maybe old; MN = maybe new; LN = likely new, DN = definitely new.

**Table S20.** Summary of t-tests in Experiment **1-Immediate Memory**

| | | **Frequentist t-test** | | | **Bayesian t-test** | |
|---|---|---|---|---|---|---|
| | | **t(119)** | **p** | **dav** | **B10** | **Note** |
| **Exp 1a All Memory** | | | | | | |
| **Pre** | **CR** | 1.85 | 0.07 | 0.14 | 1.02 | Anec H1 |
| | **DP** | 1.25 | 0.22 | 0.10 | 0.38 | Anec H0 |
| **Cond** | **CR** | 1.12 | 0.26 | 0.08 | 0.32 | Sub H0 |
| | **DP** | 0.90 | 0.37 | 0.07 | 0.24 | Sub H0 |
| **Post** | **CR** | 2.30 | 0.02* | 0.18 | 2.54 | Anec H1 |
| | **DP** | 1.61 | 0.11 | 0.13 | 0.66 | Anec H0 |
| **Exp 1a High Certainty** | | | | | | |
| **Pre** | **CR** | 0.97 | 0.33 | 0.07 | 0.27 | Sub H0 |
| | **DP** | 0.91 | 0.36 | 0.07 | 0.25 | Sub H0 |
| **Cond** | **CR** | 1.40 | 0.16 | 0.12 | 0.26 | Sub H0 |
| | **DP** | 1.05 | 0.30 | 0.10 | 0.29 | Sub H0 |
| **Post** | **CR** | 1.74 | 0.08 | 0.14 | 0.84 | Anec H0 |
| | **DP** | 1.32 | 0.19 | 0.09 | 0.42 | Anec H0 |

*Note:* Frequentist paired t-test for effect of reward category (high vs. low) on memory: $p < 0.05$ are underlined and starred, non-significant trends, $p < 0.10$, are underlined. Bayesian t-test H1: One sided hypothesis that effect of reward is greater than zero, i.e. better memory for items in high reward category, H0: No effect of reward category. Interpretation of Bayes Factors described as Sub: substantial evidence and Anec: anecdotal evidence in favor of H1 or H0.

**Table S21.** Linear Mixed-Effects Model Experiment **1-Immediate Memory** (All Memory)

| Fixed Effects | Estimate (Log Odds) | SE | 95% CI Lower | 95% CI Upper | p |
|---|---|---|---|---|---|
| Intercept | -1.85 | 0.37 | -2.53 | 1.13 | < 0.001 |
| Phase 1 | 2.36 | 0.37 | 1.74 | 3.07 | < 0.001 |
| Phase 2 | 2.32 | 0.37 | 1.65 | 2.99 | < 0.001 |
| Phase 3 | 2.22 | 0.37 | 1.56 | 2.90 | < 0.001 |
| Reward | -0.11 | 0.05 | -0.23 | 0.02 | 0.08 |
| Phase 1 : Reward | 0.20 | 0.09 | 0.05 | 0.37 | 0.06 |
| Phase 2 : Reward | 0.13 | 0.09 | -0.05 | 0.33 | 0.08 |
| Phase 3 : Reward | 0.26 | 0.09 | 0.13 | 0.47 | < 0.001 |

| Random Effects | Variance | SD |
|---|---|---|
| Stim (Intercept) | 0.34 | 0.58 |
| UserID (Intercept) | 0.14 | 0.38 |

**Table S22.** Linear Mixed-Effects Model Experiment **1-Immediate Memory** (High Certainty Memory)

| Fixed Effects | Estimate (Log Odds) | SE | 95% CI Lower | 95% CI Upper | p |
|---|---|---|---|---|---|
| Intercept | -2.38 | 0.44 | -3.29 | -1.48 | $< 0.001$ |
| Phase 1 | 3.05 | 0.44 | 2.01 | 3.94 | $< 0.001$ |
| Phase 2 | 3.02 | 0.44 | 2.05 | 3.95 | $< 0.001$ |
| Phase 3 | 2.94 | 0.44 | 1.95 | 3.83 | $< 0.001$ |
| Reward | -0.07 | 0.07 | -0.23 | 0.07 | 0.22 |
| Phase 1 : Reward | 0.17 | 0.11 | -0.06 | 0.44 | 0.08 |
| Phase 2 : Reward | 0.16 | 0.11 | -0.07 | 0.40 | 0.16 |
| Phase 3 : Reward | 0.25 | 0.11 | 0.05 | 0.48 | $< 0.001$ |
| Random Effects | | | | | |
| | Variance | SD | | | |
| Stim (Intercept) | 0.19 | 0.44 | | | |
| UserID (Intercept) | 0.64 | 0.80 | | | |

**Table S23.** Response Bias in Experiment **1-Immediate Memory**

| | All Memory | | |
|---|---|---|---|
| | Pre | Cond | Post |
| M (SD) (High Reward) | 0.413 (0.492) | 0.449 (0.486) | 0.445 (0.451) |
| M (SD) (Low Reward) | 0.435 (0.466) | 0.454 (0.461) | 0.471 (0.519) |
| p | 0.644 | 0.907 | 0.585 |
| t(119) | -0.463 | -0.117 | -0.548 |
| dav | -0.056 | -0.011 | -0.054 |
| | High Certainty Memory | | |
| | Pre | Cond | Post |
| M (SD) (High Reward) | 0.459 (0.587) | 0.466 (0.612) | 0.506 (0.567) |
| M (SD) (Low Reward) | 0.477 (0.577) | 0.492 (0.560) | 0.534 (0.611) |
| p | 0.727 | 0.587 | 0.602 |
| t(119) | -0.350 | -0.544 | -0.523 |
| dav | -0.032 | -0.045 | -0.049 |

## G. Performance on Match-to-Sample Task

**Table S24.** Summary of Performance on Match-to-Sample Task

| Phase | Accuracy | Reaction Time (ms) |
|---|---|---|
| **Exp 2a** | | |
| Pre-conditioning | 86.8 ± 0.01 | 441 ± 1.2 |
| Conditioning | 91.0 ± 0.009 | 419 ± 0.8 |
| **Exp 2b** | | |
| Conditioning | 91.0 ± 0.005 | 419 ± 1.5 |
| Post-conditioning | 86.3 ± 0.009 | 431 ± 0.2 |
| **Exp 3** | | |
| Pre-conditioning | 87.1 ± 0.01 | 439 ± 1.3 |
| Conditioning | 93.5 ± 0.004 | 417 ± 1.1 |

*Note:* Mean and standard errors of accuracies and reaction times on match-to-sample task.

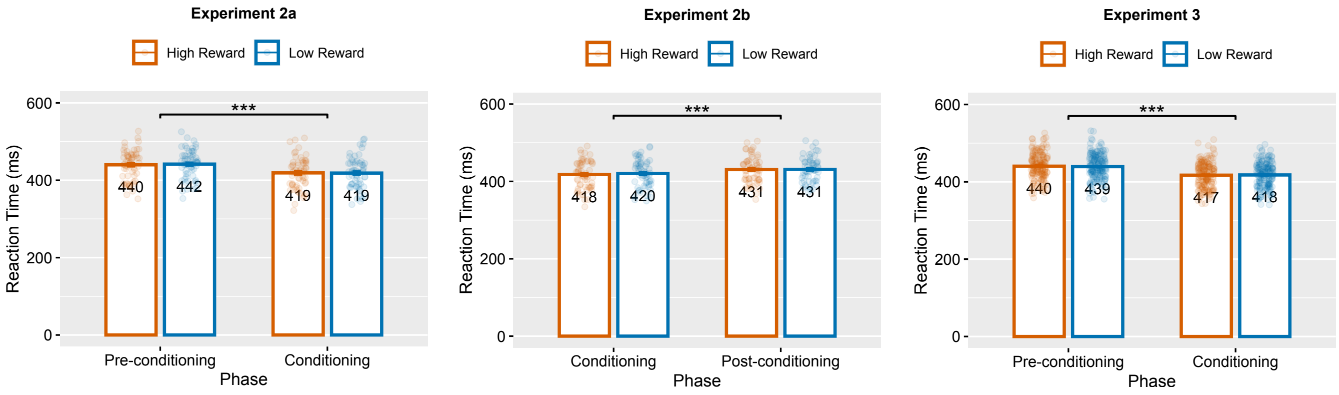


**Figure S4.** *Reaction times during match-to-sample trials by phase and reward category for Experiments 2a, 2b and 3. Group means are labelled and error bars represent* $\pm 1$ *SEM*. *** $p < 0.001$, ** $p < 0.01$, * $p < 0.05$.

## References


**1.** Patil, A., Murty, V. P., Dunsmoor, J. E., Phelps, E. A. & Davachi, L. Reward retroactively enhances memory consolidation for related items. *Learn. Mem.* **24**, 65–69 (2017).

# Supplementary Material 2 (M2)

## Experiment 1 - Main Analysis

```
# Load necessary packages
library(dplyr)
library(tidyverse)
library(rstatix)
library(ggplot2)
library(ggpubr)
library(ggprism)
library(svglite)
library(rlang)
library(plotrix)
library(gridExtra)
library(BayesFactor)
library(tinytex)
library(formatR)
library(knitr)
source("x1_funcs.R")
```

**This section contains the analysis and results associated with Experiment 1 reported in the article 'Reward conditioning may not have an effect on category-specific memory'. The main article only discusses the results of Experiment 1b which tests 24-hour delayed memory, and is referred to as Experiment 1 in the main text. In addition to this, Experiment 1a tested immediate memory retrieval. Analysis and results for both experiments are presented below.**

```
# Load Experiment 1a data (Immediate recognition memory)
data.x1a <- read.csv("Exp1a_CleanData/Main/x1a_Anova.csv")  # all memory data
data.x1a.high <- read.csv("Exp1a_CleanData/Main/x1a_High_Anova.csv")  # only high certainty data
# Change phase labels for readability
data.x1a$Phase[data.x1a$Phase == "Ph1"] <- "Pre-conditioning"
data.x1a$Phase[data.x1a$Phase == "Ph2"] <- "Conditioning"
data.x1a$Phase[data.x1a$Phase == "Ph3"] <- "Post-conditioning"
data.x1a.high$Phase[data.x1a.high$Phase == "Ph1"] <- "Pre-conditioning"
data.x1a.high$Phase[data.x1a.high$Phase == "Ph2"] <- "Conditioning"
data.x1a.high$Phase[data.x1a.high$Phase == "Ph3"] <- "Post-conditioning"
# Reorder variables for graphs
data.x1a$Reward_Category <- factor(data.x1a$Reward_Category,
    levels = c("High Reward", "Low Reward"))
data.x1a$Phase <- factor(data.x1a$Phase, levels = c("Pre-conditioning",
    "Conditioning", "Post-conditioning"))
data.x1a.high$Reward_Category <- factor(data.x1a.high$Reward_Category,
    levels = c("High Reward", "Low Reward"))
data.x1a.high$Phase <- factor(data.x1a.high$Phase, levels = c("Pre-conditioning",
    "Conditioning", "Post-conditioning"))

# Load Experiment 1b data (24 hour recognition memory)
data.x1b <- read.csv("Exp1b_CleanData/Main/x1b_Anova.csv")  # all memory data
```

```r
data.x1b.high <- read.csv("Exp1b_CleanData/Main/x1b_High_Anova.csv")  # only high certainty data
# Change phase labels for readability
data.x1b$Phase[data.x1b$Phase == "Ph1"] <- "Pre-conditioning"
data.x1b$Phase[data.x1b$Phase == "Ph2"] <- "Conditioning"
data.x1b$Phase[data.x1b$Phase == "Ph3"] <- "Post-conditioning"
data.x1b.high$Phase[data.x1b.high$Phase == "Ph1"] <- "Pre-conditioning"
data.x1b.high$Phase[data.x1b.high$Phase == "Ph2"] <- "Conditioning"
data.x1b.high$Phase[data.x1b.high$Phase == "Ph3"] <- "Post-conditioning"
# Reorder variables for graphs
data.x1b$Reward_Category <- factor(data.x1b$Reward_Category,
    levels = c("High Reward", "Low Reward"))
data.x1b$Phase <- factor(data.x1b$Phase, levels = c("Pre-conditioning",
    "Conditioning", "Post-conditioning"))
data.x1b.high$Reward_Category <- factor(data.x1b.high$Reward_Category,
    levels = c("High Reward", "Low Reward"))
data.x1b.high$Phase <- factor(data.x1b.high$Phase, levels = c("Pre-conditioning",
    "Conditioning", "Post-conditioning"))
```

# Data format

**Datasets:**

By participant summary of performance on the matching and memory tasks. There are two summary datasets for each experiment:

1. data.x1a & data.x1b summarises all memory trials
2. data.x1a.high & data.x1b.high summarises memory trials in which participants responded with higher certainty (confidence rating). This includes trials with 'Definitely Old/New' and 'Likely Old/New' responses, and excludes 'Maybe Old/New' responses.

**Data variables:**

1. UserID: unique user identification
2. Category: stimuli category ("Animal", "Object")
3. Reward_Category: stimuli reward category (High Reward",”Low Reward")
4. Phase: phase in which stimuli was encoded ("Pre-conditioning", "Conditioning", "Post-conditioning")
5. CR: corrected recognition scores from memory task
6. DP: d-prime memory sensitivity in memory task (as per signal detection theory)
7. MA: guessing accuracy in word-image matching task during encoding
8. RT: reaction time (ms) in word-image matching task during encoding
9. RB: response bias in memory task (as per signal detection theory)

Further unused variables: 10. Rew_Subgroup: allocation of situmuli category to high reward ("Reward_Animals", "Reward_Objects") 11. Age 12. Sex 13. HR: hit rate in memory task 14. FA: false alarm rate in memory task

# 1. Main Analysis (Frequentists statistics)

Recognition memory performance was calculated using two measures: corrected recognition (hit rate - false alarm rate) and (d-prime) memory sensitivity as per signal detection theory. Parametric tests were used since the sample size (n = 120) was large enough (n > 30) to assume that data follows normality requirements.

Firstly, a 2x3 factor repeated measures Anova was done to characterise the effects of phase and reward category on the memory of items. This analysis was performed on both measures of memory. Mauchly's test of sphericity was applied to check if variances of group differences are equal. If the sphericity assumptions were violated then the Greenhouse-Geisser corrected results were reported. Following this, the effect of reward category (high vs. low reward) on the memory of items from each phase was quantified using two-tailed paired t-tests with alpha = .05.

For each experiment, we then repeated the analysis taking into account only high-certainty memory responses.

## 1.1 Experiment 1a (All Memory)

**Corrected recognition (CR) by phase and reward category**

```
# Summary table and graph
aggregate(CR ~ Reward_Category + Phase, data.x1a, FUN = function(CR) c(mean = mean(CR),
    se = std.error(CR)))
```

```
##   Reward_Category             Phase    CR.mean      CR.se
## 1     High Reward  Pre-conditioning 0.49654034 0.02111878
## 2      Low Reward  Pre-conditioning 0.46432920 0.02122155
## 3     High Reward      Conditioning 0.47490839 0.02155314
## 4      Low Reward      Conditioning 0.45384309 0.02171248
## 5     High Reward Post-conditioning 0.47729431 0.02225552
## 6      Low Reward Post-conditioning 0.43107523 0.02538487
```

```
x1a.CR = plot_by_group(data = data.x1a, yvar = "CR", ylim = c(0,
    1.2), ylab = "Corrected Recognition", subtitle = "Experiment 1a (All Memory)",
    tag = "1.1 A")
x1a.CR
```

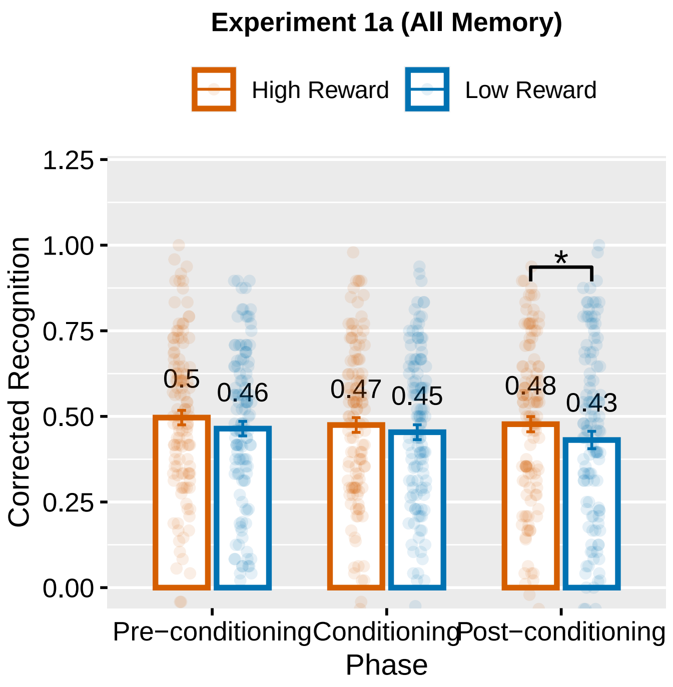

```r
# Repeated measures two-factor ANOVA on corrected
# recognition
anova_test(CR ~ Phase * Reward_Category + Error(UserID/(Phase *
    Reward_Category)), data = data.x1a)
```

```
## ANOVA Table (type III tests)
##
## $ANOVA
##                    Effect DFn DFd     F     p p<.05      ges
## 1                   Phase   2 238 1.848 0.160       0.002000
## 2         Reward_Category   1 119 5.432 0.021     * 0.005000
## 3 Phase:Reward_Category   2 238 0.705 0.495       0.000449
##
## $`Mauchly's Test for Sphericity`
##                    Effect     W     p p<.05
## 1                   Phase 0.916 0.006     *
## 2 Phase:Reward_Category 0.995 0.748
##
## $`Sphericity Corrections`
##                    Effect   GGe        DF[GG] p[GG] p[GG]<.05   HFe        DF[HF]
## 1                   Phase 0.922 1.84, 219.53 0.163           0.936 1.87, 222.83
## 2 Phase:Reward_Category 0.995 1.99, 236.84 0.495           1.012 2.02, 240.85
##   p[HF] p[HF]<.05
## 1 0.163
## 2 0.495
```

The sphericity assumption was not met for the phase factor, W = .92, p = .006, and so Greenhouse-Geisser corrections were applied to the results. The repeated measures ANOVA revealed an effect of reward category, F(1,119) = 5.43, p = .021, $\eta^2$ = .005, on corrected recognition. However there was no significant effect of phase or an interaction between encoding phase and the reward category associated with the item, F(1,236.84) = 0.705, p = .50, $\eta^2 < .001$. We next repeat the same ANOVA analysis for d-prime measures.

**d-prime (DP) by phase and reward category**

```r
# Summary table and graph
aggregate(DP ~ Reward_Category + Phase, data.x1a, FUN = function(DP) c(mean = mean(DP),
    se = std.error(DP)))
```

```
##   Reward_Category              Phase    DP.mean      DP.se
## 1     High Reward  Pre-conditioning 2.33013918 0.09449937
## 2      Low Reward  Pre-conditioning 2.23015253 0.08858173
## 3     High Reward      Conditioning 2.24917812 0.09307704
## 4      Low Reward      Conditioning 2.17478956 0.09342245
## 5     High Reward Post-conditioning 2.25417101 0.09257070
## 6      Low Reward Post-conditioning 2.10489669 0.11533933
```

```r
x1a.DP = plot_by_group(data = data.x1a, yvar = "DP", ylim = c(0,
    4.6), ylab = "d-prime", subtitle = "Experiment 1a (All Memory)",
    tag = "1.1 B")
x1a.DP
```

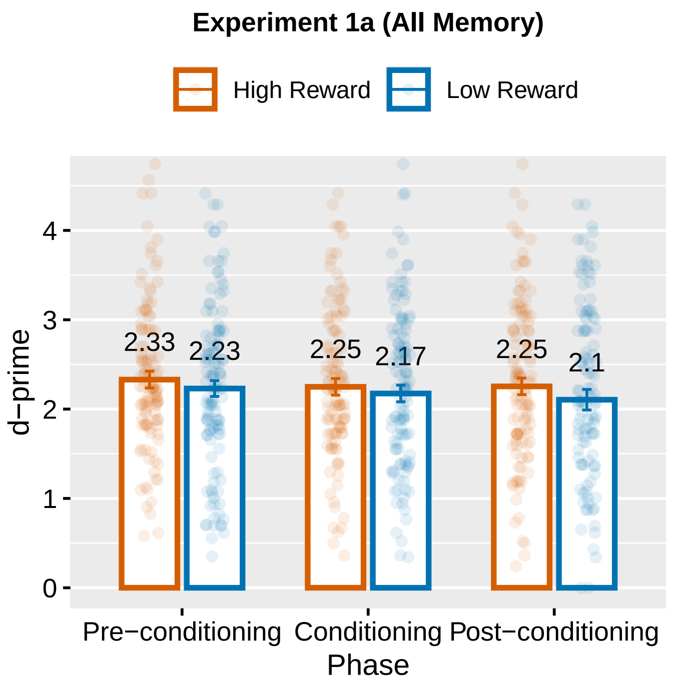


```
# Repeated measures two-factor ANOVA on d-prime scores
anova_test(DP ~ Phase * Reward_Category + Error(UserID/(Phase *
    Reward_Category)), data = data.x1a)
```

```
## ANOVA Table (type III tests)
##
## $ANOVA
##                  Effect DFn DFd     F     p p<.05      ges
## 1                 Phase   2 238 1.652 0.194       0.002000
## 2       Reward_Category   1 119 2.614 0.109       0.003000
## 3 Phase:Reward_Category   2 238 0.340 0.712       0.000217
##
## $`Mauchly's Test for Sphericity`
##                  Effect     W     p p<.05
## 1                 Phase 0.934 0.018     *
## 2 Phase:Reward_Category 0.988 0.483
##
## $`Sphericity Corrections`
##                  Effect   GGe       DF[GG] p[GG] p[GG]<.05   HFe       DF[HF]
## 1                 Phase 0.938 1.88, 223.29 0.196           0.953 1.91, 226.74
## 2 Phase:Reward_Category 0.988 1.98, 235.12 0.710           1.004 2.01, 239.06
##   p[HF] p[HF]<.05
## 1 0.195
## 2 0.712
```

The sphericity assumption was not met for the phase factor, W = .93, p = .02, and so Greenhouse-Geisser corrections were applied to the results. The repeated measures ANOVA revealed no significant effects of reward category or phase on d-prime measures. More importantly, there was no significant interaction effect

between encoding phase and the reward category associated with the item F(1,235.12) = 0.340, p = .71, $\eta^2$ < .001. Following this we used t-tests to characterise the effect on memory for items in each encoding phase.

```r
# Create subsets for each phase from data.x1a (all memory)
x1a_ph1 <- subset(data.x1a, Phase == "Pre-conditioning")
x1a_ph2 <- subset(data.x1a, Phase == "Conditioning")
x1a_ph3 <- subset(data.x1a, Phase == "Post-conditioning")
```

```r
# Effect of reward category on memory in phase 1
# (pre-conditioning) Corrected recognition (CR)
t.test(data = x1a_ph1, CR ~ Reward_Category, paired = TRUE)
```

```
##
##  Paired t-test
##
## data:  CR by Reward_Category
## t = 1.8485, df = 119, p-value = 0.06702
## alternative hypothesis: true difference in means is not equal to 0
## 95 percent confidence interval:
##  -0.002293699  0.066715980
## sample estimates:
## mean of the differences
##              0.03221114
```

```r
cohens_dav(data = x1a_ph1, x = CR, group = Reward_Category)
```

```
## # A tibble: 2 x 4
##   Reward_Category count  mean    sd
##   <fct>           <int> <dbl> <dbl>
## 1 High Reward       120 0.497 0.231
## 2 Low Reward        120 0.464 0.232
## [1] "Effect size Cohen's d(av):"
## [1] 0.1388965
```

```r
# d-prime (DP)
t.test(data = x1a_ph1, DP ~ Reward_Category, paired = TRUE)
```

```
##
##  Paired t-test
##
## data:  DP by Reward_Category
## t = 1.2451, df = 119, p-value = 0.2155
## alternative hypothesis: true difference in means is not equal to 0
## 95 percent confidence interval:
##  -0.0590199  0.2589932
## sample estimates:
## mean of the differences
##              0.09998665
```

```r
cohens_dav(data = x1a_ph1, x = DP, group = Reward_Category)
```

```
## # A tibble: 2 x 4
##   Reward_Category count  mean    sd
##   <fct>           <int> <dbl> <dbl>
## 1 High Reward       120  2.33 1.04
## 2 Low Reward        120  2.23 0.970
## [1] "Effect size Cohen's d(av):"
## [1] 0.0997098
```

In the pre-conditioning phase of experiment 1a, there was a non-significant trend for better memory performance for items in the high reward category t(119) = 1.85, p = .07, $d_{av}$ = .14. However, this effect was not significant with d-prime measures t(119) = 1.25, p = .22, $d_{av}$ = .10. The reward category effect meant that items belonging to the high reward category resulted in better recognition memory performance than items from the low reward category. This was a non-significant trend.

```
# Effect of reward category on memory in phase 2
# (conditioning)

# Corrected recognition (CR)
t.test(data = x1a_ph2, CR ~ Reward_Category, paired = TRUE)
```

```
##
##  Paired t-test
##
## data:  CR by Reward_Category
## t = 1.1217, df = 119, p-value = 0.2642
## alternative hypothesis: true difference in means is not equal to 0
## 95 percent confidence interval:
##  -0.01611999  0.05825060
## sample estimates:
## mean of the differences
##              0.02106531
```

```
cohens_dav(data = x1a_ph2, x = CR, group = Reward_Category)
```

```
## # A tibble: 2 x 4
##   Reward_Category count  mean    sd
##   <fct>           <int> <dbl> <dbl>
## 1 High Reward       120 0.475 0.236
## 2 Low Reward        120 0.454 0.238
## [1] "Effect size Cohen's d(av):"
## [1] 0.08889231
```

```
# d-prime (DP)
t.test(data = x1a_ph2, DP ~ Reward_Category, paired = TRUE)
```

```
##
##  Paired t-test
##
## data:  DP by Reward_Category
## t = 0.90005, df = 119, p-value = 0.3699
## alternative hypothesis: true difference in means is not equal to 0
## 95 percent confidence interval:
```

```
##  -0.08926634  0.23804345
## sample estimates:
## mean of the differences
##              0.07438856
```

```
cohens_dav(data = x1a_ph2, x = DP, group = Reward_Category)
```

```
## # A tibble: 2 x 4
##   Reward_Category count  mean    sd
##   <fct>           <int> <dbl> <dbl>
## 1 High Reward       120  2.25  1.02
## 2 Low Reward        120  2.17  1.02
## [1] "Effect size Cohen's d(av):"
## [1] 0.07282288
```

In the conditioning phase of experiment 1a, there was no evidence for a significant effect of reward category neither with corrected recognition $t(119) = 1.85$, $p = .07$, $d_{av} = .14$, nor with d-prime measures $t(119) = 1.25$, $p = .22$, $d_{av} = .10$. This reveals that the reward conditioning was not successful in this experiment, which involved immediate recall without a 24 hour consolidation period after encoding. Thus it casts doubt on the trend level effect seen in the pre-conditioning phase, which could have arisen due to response biases or other factors which will be explored in the control analyses.

```
# Effect of reward category on memory in phase 3
# (post-conditioning)

# Corrected recognition (CR)
t.test(data = x1a_ph3, CR ~ Reward_Category, paired = TRUE)
```

```
##
##  Paired t-test
##
## data:  CR by Reward_Category
## t = 2.3049, df = 119, p-value = 0.02291
## alternative hypothesis: true difference in means is not equal to 0
## 95 percent confidence interval:
##  0.006512898 0.085925257
## sample estimates:
## mean of the differences
##              0.04621908
```

```
cohens_dav(data = x1a_ph3, x = CR, group = Reward_Category)
```

```
## # A tibble: 2 x 4
##   Reward_Category count  mean    sd
##   <fct>           <int> <dbl> <dbl>
## 1 High Reward       120 0.477 0.244
## 2 Low Reward        120 0.431 0.278
## [1] "Effect size Cohen's d(av):"
## [1] 0.1771272
```

```r
# d-prime (DP)
t.test(data = x1a_ph3, DP ~ Reward_Category, paired = TRUE)
```

```
##
##  Paired t-test
##
## data:  DP by Reward_Category
## t = 1.6086, df = 119, p-value = 0.1103
## alternative hypothesis: true difference in means is not equal to 0
## 95 percent confidence interval:
##  -0.03447214  0.33302079
## sample estimates:
## mean of the differences
##               0.1492743
```

```r
cohens_dav(data = x1a_ph3, x = DP, group = Reward_Category)
```

```
## # A tibble: 2 x 4
##   Reward_Category count  mean    sd
##   <fct>           <int> <dbl> <dbl>
## 1 High Reward       120  2.25  1.01
## 2 Low Reward        120  2.10  1.26
## [1] "Effect size Cohen's d(av):"
## [1] 0.1310838
```

Finally, in the post-conditioning phase of experiment 1a, again we found a significant effect of reward category with corrected recognition t(119) = 2.30, p = .02, $d_{av}$ = .18, but not with d-prime measures t(119) = 1.61, p = .11, $d_{av}$ = .13. Knowing that the reward conditioning was not successful in phase 2 (conditioning phase), this effect in the post-conditioning phase is unusual. Moreover, as it is evident in corrected recognition but not in d-primes, this could be due to response biases or other confounding factors which will be explored in the control analyses.

## 1.2 Experiment 1a (High Certainty Memory)

Data from experiment 1a was re-analysed considering only high certainty memory responses. **Corrected recognition (CR) by phase and reward category**

```r
# Summary table and graph
aggregate(CR ~ Reward_Category + Phase, data.x1a.high, FUN = function(CR) c(mean = mean(CR),
    se = std.error(CR)))
```

```
##   Reward_Category             Phase    CR.mean      CR.se
## 1     High Reward  Pre-conditioning 0.54617495 0.02347494
## 2      Low Reward  Pre-conditioning 0.52813855 0.02148373
## 3     High Reward      Conditioning 0.54719991 0.02139180
## 4      Low Reward      Conditioning 0.51808845 0.02153488
## 5     High Reward Post-conditioning 0.52877048 0.02306664
## 6      Low Reward Post-conditioning 0.49206341 0.02527175
```

```r
x1a.high.CR = plot_by_group(data = data.x1a.high, yvar = "CR",
    ylim = c(0, 1.2), ylab = "Corrected Recognition", subtitle = "Experiment 1a (High Certainty)",
    tag = "1.2 A")
x1a.high.CR
```

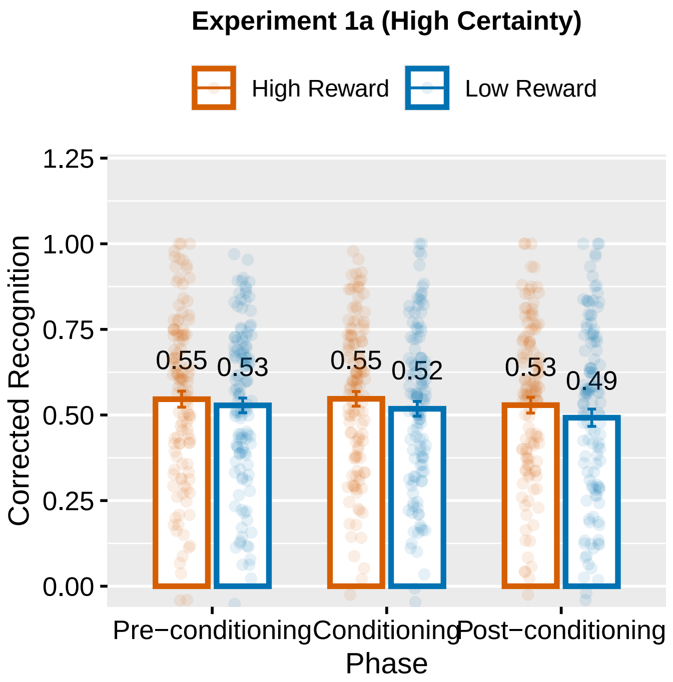


```r
# Repeated measures two-factor ANOVA on corrected
# recognition (high certaintly only)
anova_test(CR ~ Phase * Reward_Category + Error(UserID/(Phase *
    Reward_Category)), data = data.x1a.high)
```

```
## ANOVA Table (type III tests)
##
## $ANOVA
##                  Effect DFn DFd     F     p p<.05      ges
## 1                 Phase   2 238 1.837 0.162       0.002000
## 2       Reward_Category   1 119 3.579 0.061       0.003000
## 3 Phase:Reward_Category   2 238 0.312 0.732       0.000239
##
## $`Mauchly's Test for Sphericity`
##                  Effect     W     p p<.05
## 1                 Phase 0.892 0.001     *
## 2 Phase:Reward_Category 0.970 0.167
##
## $`Sphericity Corrections`
##                  Effect   GGe       DF[GG] p[GG] p[GG]<.05   HFe        DF[HF]
## 1                 Phase 0.903 1.81, 214.87 0.166           0.916 1.83, 217.98
## 2 Phase:Reward_Category 0.971  1.94, 231.1 0.726           0.987 1.97, 234.87
##   p[HF] p[HF]<.05
```

```
## 1 0.165
## 2 0.729
```

The sphericity assumption was not met for the phase factor, W = .89, p = .001, and so Greenhouse-Geisser corrections were applied to the results. The repeated measures ANOVA revealed no significant effects of reward category or phase on corrected recognition. More importantly, there was no significant effect of phase or an interaction between encoding phase and the reward category associated with the item, F(1,231.1) = 0.312, p = .73, $\eta^2 < .001$. We next repeat the same ANOVA analysis for d-prime measures.

**d-prime (DP) by phase and reward category**

```
# Summary table and graph
aggregate(DP ~ Reward_Category + Phase, data.x1a.high, FUN = function(DP) c(mean = mean(DP),
    se = std.error(DP)))
```

```
##   Reward_Category              Phase   DP.mean      DP.se
## 1     High Reward  Pre-conditioning 2.68390084 0.11058959
## 2      Low Reward  Pre-conditioning 2.60237914 0.09097430
## 3     High Reward      Conditioning 2.67009939 0.09340769
## 4      Low Reward      Conditioning 2.57034957 0.09656308
## 5     High Reward Post-conditioning 2.57553055 0.10766307
## 6      Low Reward Post-conditioning 2.45357888 0.11744961
```

```
x1a.high.DP = plot_by_group(data = data.x1a.high, yvar = "DP",
    ylim = c(0, 4.6), ylab = "d-prime", subtitle = "Experiment 1a (High Certainty)",
    tag = "1.2 B")
x1a.high.DP
```

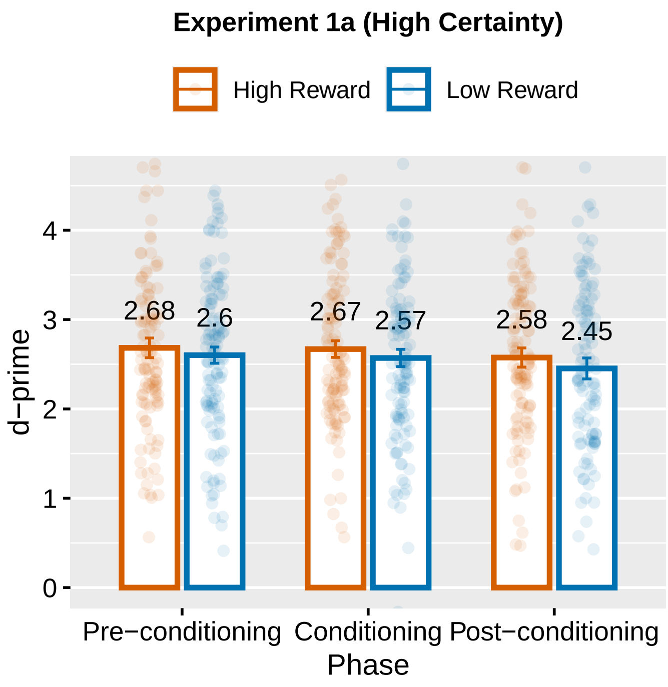

```r
# Repeated measures two-factor ANOVA on d-prime scores
# (high certainty only)
anova_test(DP ~ Phase * Reward_Category + Error(UserID/(Phase *
    Reward_Category)), data = data.x1a.high)
```

```
## ANOVA Table (type III tests)
##
## $ANOVA
##                    Effect DFn DFd     F     p p<.05      ges
## 1                   Phase   2 238 2.217 0.111       2.00e-03
## 2         Reward_Category   1 119 2.084 0.151       2.00e-03
## 3 Phase:Reward_Category   2 238 0.075 0.927       5.39e-05
##
## $`Mauchly's Test for Sphericity`
##                    Effect     W     p p<.05
## 1                   Phase 0.902 0.002     *
## 2 Phase:Reward_Category 0.996 0.804
##
## $`Sphericity Corrections`
##                    Effect   GGe       DF[GG] p[GG] p[GG]<.05   HFe       DF[HF]
## 1                   Phase 0.911 1.82, 216.77 0.116           0.924 1.85, 219.96
## 2 Phase:Reward_Category 0.996 1.99, 237.13 0.927           1.013 2.03, 241.16
##   p[HF] p[HF]<.05
## 1 0.115
## 2 0.927
```

The sphericity assumption was not met for the phase factor, W = .90, p = .002, and so Greenhouse-Geisser corrections were applied to the results. The repeated measures ANOVA revealed no significant effects of reward category or phase on d-prime measures. More importantly, there was no significant effect of phase or an interaction between encoding phase and the reward category associated with the item, $F(1,237.13) = 0.075$, $p = .93$, $\eta^2 < .001$. This analysis was consistent with the results for corrected recognition.

```r
# Create subsets for each phase from data.x1a (high
# certainty)
x1a_high_ph1 <- subset(data.x1a.high, Phase == "Pre-conditioning")
x1a_high_ph2 <- subset(data.x1a.high, Phase == "Conditioning")
x1a_high_ph3 <- subset(data.x1a.high, Phase == "Post-conditioning")
```

```r
# Effect of reward category on high certainty memory in
# phase 1 (pre-conditioning)

# Corrected recognition (CR)
t.test(data = x1a_high_ph1, CR ~ Reward_Category, paired = TRUE)
```

```
##
##  Paired t-test
##
## data:  CR by Reward_Category
## t = 0.96899, df = 119, p-value = 0.3345
## alternative hypothesis: true difference in means is not equal to 0
## 95 percent confidence interval:
##  -0.01882054  0.05489335
```

```
## sample estimates:
## mean of the differences
##              0.01803641
```

```r
cohens_dav(data = x1a_high_ph1, x = CR, group = Reward_Category)
```

```
## # A tibble: 2 x 4
##   Reward_Category count  mean    sd
##   <fct>           <int> <dbl> <dbl>
## 1 High Reward       120 0.546 0.257
## 2 Low Reward        120 0.528 0.235
## [1] "Effect size Cohen's d(av):"
## [1] 0.07324467
```

```r
# d-prime (DP)
t.test(data = x1a_high_ph1, DP ~ Reward_Category, paired = TRUE)
```

```
##
##  Paired t-test
##
## data:  DP by Reward_Category
## t = 0.91172, df = 119, p-value = 0.3638
## alternative hypothesis: true difference in means is not equal to 0
## 95 percent confidence interval:
##  -0.09552924  0.25857266
## sample estimates:
## mean of the differences
##              0.08152171
```

```r
cohens_dav(data = x1a_high_ph1, x = DP, group = Reward_Category)
```

```
## # A tibble: 2 x 4
##   Reward_Category count  mean    sd
##   <fct>           <int> <dbl> <dbl>
## 1 High Reward       120  2.68 1.21
## 2 Low Reward        120  2.60 0.997
## [1] "Effect size Cohen's d(av):"
## [1] 0.0738414
```

When repeating the analysis with high certainty memory trials, t-tests revealed that the effect of reward category on corrected recognition in the pre-conditioning phase observed in the full memory analysis was no longer significant, $t(119) = 0.97$, $p = .33$, $d_{av} = .07$. This was for both d-prime measures as well, $t(119) = 0.91$, $p = .36$, $d_{av} = .07$.

```r
# Effect of reward category on high certainty memory in
# phase 2 (conditioning)

# Corrected recognition (CR)
t.test(data = x1a_high_ph2, CR ~ Reward_Category, paired = TRUE)
```

```
##
##  Paired t-test
##
## data:  CR by Reward_Category
## t = 1.4045, df = 119, p-value = 0.1628
## alternative hypothesis: true difference in means is not equal to 0
## 95 percent confidence interval:
##  -0.01192968  0.07015261
## sample estimates:
## mean of the differences
##              0.02911147
```

```r
cohens_dav(data = x1a_high_ph2, x = CR, group = Reward_Category)
```

```
## # A tibble: 2 x 4
##   Reward_Category count  mean    sd
##   <fct>           <int> <dbl> <dbl>
## 1 High Reward       120 0.547 0.234
## 2 Low Reward        120 0.518 0.236
## [1] "Effect size Cohen's d(av):"
## [1] 0.1238158
```

```r
# d-prime (DP)
t.test(data = x1a_high_ph2, DP ~ Reward_Category, paired = TRUE)
```

```
##
##  Paired t-test
##
## data:  DP by Reward_Category
## t = 1.0475, df = 119, p-value = 0.297
## alternative hypothesis: true difference in means is not equal to 0
## 95 percent confidence interval:
##  -0.08881518  0.28831483
## sample estimates:
## mean of the differences
##              0.09974982
```

```r
cohens_dav(data = x1a_high_ph2, x = DP, group = Reward_Category)
```

```
## # A tibble: 2 x 4
##   Reward_Category count  mean    sd
##   <fct>           <int> <dbl> <dbl>
## 1 High Reward       120  2.67  1.02
## 2 Low Reward        120  2.57  1.06
## [1] "Effect size Cohen's d(av):"
## [1] 0.09586602
```

For items encoded in the conditioning phase, t-tests revealed no evidence for an effect of reward category on corrected recognition, $t(119) = 1.40$, $p = .16$, $d_{av} = .12$, nor on d-primes, $t(119) = 1.05$, $p = .30$, $d_{av} = .10$.

```r
# Effect of reward category on high certainty memory in
# phase 3 (post-conditioning)

# Corrected recognition (CR)
t.test(data = x1a_high_ph3, CR ~ Reward_Category, paired = TRUE)
```

```
##
##  Paired t-test
##
## data:  CR by Reward_Category
## t = 1.7439, df = 119, p-value = 0.08376
## alternative hypothesis: true difference in means is not equal to 0
## 95 percent confidence interval:
##  -0.004972267  0.078386419
## sample estimates:
## mean of the differences
##              0.03670708
```

```r
cohens_dav(data = x1a_high_ph3, x = CR, group = Reward_Category)
```

```
## # A tibble: 2 x 4
##   Reward_Category count  mean    sd
##   <fct>           <int> <dbl> <dbl>
## 1 High Reward       120 0.529 0.253
## 2 Low Reward        120 0.492 0.277
## [1] "Effect size Cohen's d(av):"
## [1] 0.1386427
```

```r
# d-prime (DP)
t.test(data = x1a_high_ph3, DP ~ Reward_Category, paired = TRUE)
```

```
##
##  Paired t-test
##
## data:  DP by Reward_Category
## t = 1.3213, df = 119, p-value = 0.189
## alternative hypothesis: true difference in means is not equal to 0
## 95 percent confidence interval:
##  -0.06081172  0.30471506
## sample estimates:
## mean of the differences
##               0.1219517
```

```r
cohens_dav(data = x1a_high_ph3, x = DP, group = Reward_Category)
```

```
## # A tibble: 2 x 4
##   Reward_Category count  mean    sd
##   <fct>           <int> <dbl> <dbl>
## 1 High Reward       120  2.58  1.18
## 2 Low Reward        120  2.45  1.29
## [1] "Effect size Cohen's d(av):"
## [1] 0.09890704
```

For items encoded in the post-conditioning phase, t-tests revealed no significant evidence for an effect of reward category on corrected recognition, t(119) = 1.74, p = .08, $d_{av}$ = .14, nor on d-primes, t(119) = 1.32, p = .19, $d_{av}$ = .09. The pattern of results casts doubt on the weak effects observed in the main analysis, which showed that items from the high reward category resulted in enhanced corrected recognition, but not d-prime measures.

## 1.3 Experiment 1b (All Memory)

### Corrected recognition (CR) by phase and reward category

```
# Summary table and graph
aggregate(CR ~ Reward_Category + Phase, data.x1b, FUN = function(CR) c(mean = mean(CR),
    se = std.error(CR)))
```

```
##   Reward_Category             Phase    CR.mean      CR.se
## 1     High Reward  Pre-conditioning 0.33585004 0.01766898
## 2      Low Reward  Pre-conditioning 0.34184953 0.01690323
## 3     High Reward      Conditioning 0.31411392 0.01695657
## 4      Low Reward      Conditioning 0.27063425 0.01458825
## 5     High Reward Post-conditioning 0.29612781 0.01943940
## 6      Low Reward Post-conditioning 0.30141798 0.01781441
```

```
x1b.CR = plot_by_group(data = data.x1b, yvar = "CR", ylim = c(0,
    1.2), ylab = "Corrected Recognition", subtitle = "Experiment 1b (All Memory)",
    tag = "1.3 A")
ggsave(file = "x1a.CR.svg", plot = x1b.CR, width = 10, height = 10,
    units = "cm")
x1b.CR
```

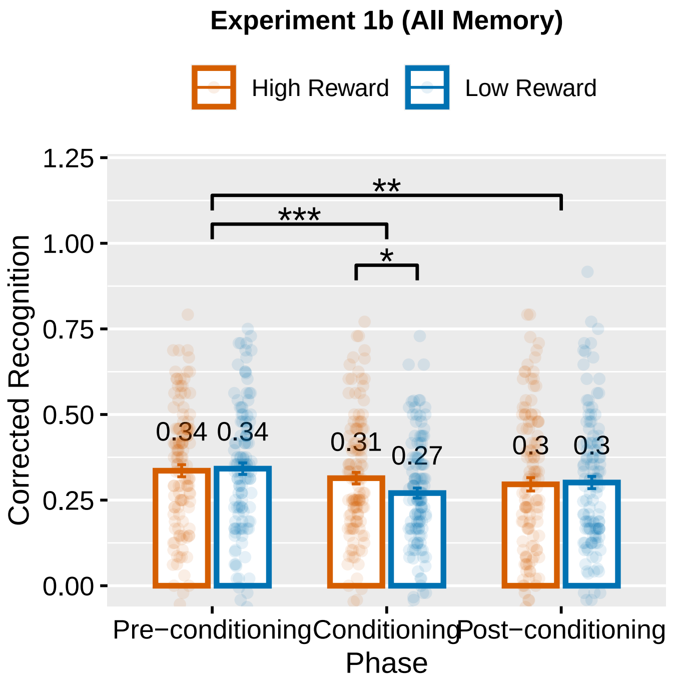

```r
# Repeated measures two-factor ANOVA on corrected
# recognition
anova_test(CR ~ Phase * Reward_Category + Error(UserID/(Phase *
    Reward_Category)), data = data.x1b)
```

```
## ANOVA Table (type III tests)
##
## $ANOVA
##                  Effect DFn DFd     F        p p<.05      ges
## 1                 Phase   2 238 7.288 0.000847     * 0.012000
## 2       Reward_Category   1 119 0.717 0.399000       0.000809
## 3 Phase:Reward_Category   2 238 3.457 0.033000     * 0.004000
##
## $`Mauchly's Test for Sphericity`
##                  Effect     W     p p<.05
## 1                 Phase 0.905 0.003     *
## 2 Phase:Reward_Category 0.975 0.220
##
## $`Sphericity Corrections`
##                  Effect   GGe       DF[GG] p[GG] p[GG]<.05   HFe       DF[HF]
## 1                 Phase 0.913 1.83, 217.31 0.001         * 0.927 1.85, 220.52
## 2 Phase:Reward_Category 0.975 1.95, 232.12 0.034         * 0.991 1.98, 235.93
##   p[HF] p[HF]<.05
## 1 0.001         *
## 2 0.034         *
```

The sphericity assumption was not met for the phase factor, W = .91, p = .003, and so Greenhouse-Geisser corrections were applied to the results. The repeated measures ANOVA revealed an effect of phase, F(1,217.31) = 7.29, p = .001, $\eta^2$ = .005, on corrected recognition. There was a weak but significant interaction effect between encoding phase and reward category, F(1,232.12) = 3.46, p = .03, $\eta^2$ = .004. This meant that the effect of reward category varied with phase, and this will be more specifically characterised using t-tests. We next repeat the same ANOVA analysis for d-prime measures.

**d-prime (DP) by phase and reward category**

```r
# Summary table and graph
aggregate(DP ~ Reward_Category + Phase, data.x1b, FUN = function(DP) c(mean = mean(DP),
    se = std.error(DP)))
```

```
##   Reward_Category             Phase    DP.mean      DP.se
## 1     High Reward  Pre-conditioning 1.68119404 0.07126621
## 2      Low Reward  Pre-conditioning 1.69365539 0.06334560
## 3     High Reward      Conditioning 1.62551178 0.06776095
## 4      Low Reward      Conditioning 1.47095094 0.05959869
## 5     High Reward Post-conditioning 1.53010725 0.08065637
## 6      Low Reward Post-conditioning 1.54814468 0.07487318
```

```r
x1b.DP = plot_by_group(data = data.x1b, yvar = "DP", ylim = c(0,
    4.6), ylab = "d-prime", subtitle = "Experiment 1b (All Memory)",
    tag = "1.3 B")
x1b.DP
```

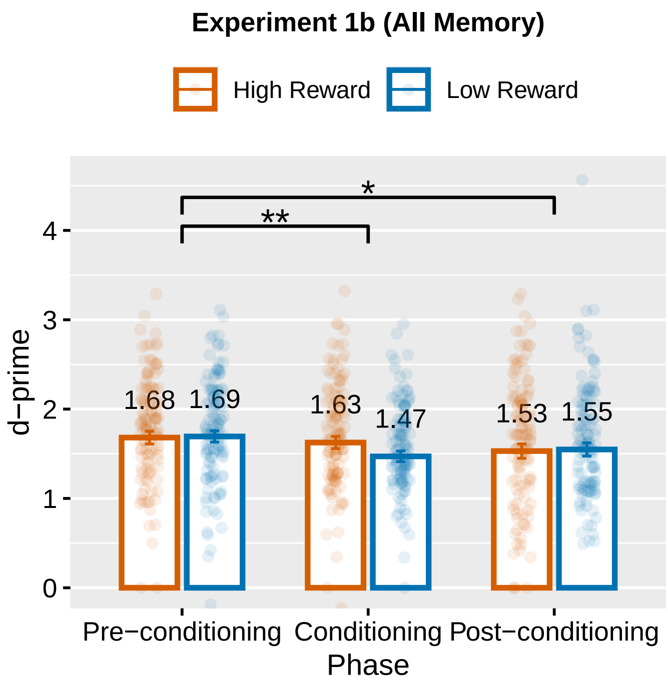


```r
# Repeated measures two-factor ANOVA on d-prime scores
anova_test(DP ~ Phase * Reward_Category + Error(UserID/(Phase *
    Reward_Category)), data = data.x1b)
```

```
## ANOVA Table (type III tests)
##
## $ANOVA
##                  Effect DFn DFd     F     p p<.05      ges
## 1                 Phase   2 238 4.481 0.012     * 0.008000
## 2       Reward_Category   1 119 0.569 0.452       0.000734
## 3 Phase:Reward_Category   2 238 2.452 0.088       0.003000
##
## $`Mauchly's Test for Sphericity`
##                  Effect     W        p p<.05
## 1                 Phase 0.879 0.000486     *
## 2 Phase:Reward_Category 0.914 0.005000     *
##
## $`Sphericity Corrections`
##                  Effect   GGe       DF[GG] p[GG] p[GG]<.05   HFe       DF[HF]
## 1                 Phase 0.892 1.78, 212.25 0.016         * 0.904 1.81, 215.26
## 2 Phase:Reward_Category 0.920 1.84, 219.06 0.093           0.934 1.87, 222.34
##   p[HF] p[HF]<.05
## 1 0.015         *
## 2 0.092
```

The sphericity assumption was not met for the phase and interaction terms and so Greenhouse-Geisser corrections were applied to the results. The repeated measures ANOVA revealed an effect of phase, F(1,212.25) = 4.48, p = .02, $\eta^2$ = .008, on d-prime. There was no significant interaction effect between encoding phase and reward category, F(1,219.06) = 2.45, p = .09, $\eta^2$ = .003.

```r
# Create subsets for each phase from data.x1b (all memory)
x1b_ph1 <- subset(data.x1b, Phase == "Pre-conditioning")
x1b_ph2 <- subset(data.x1b, Phase == "Conditioning")
x1b_ph3 <- subset(data.x1b, Phase == "Post-conditioning")
```

```r
# Effect of reward category on memory in phase 1
# (pre-conditioning)

# Corrected recognition (CR)
t.test(data = x1b_ph1, CR ~ Reward_Category, paired = TRUE)
```

```
##
##  Paired t-test
##
## data:  CR by Reward_Category
## t = -0.37686, df = 119, p-value = 0.7069
## alternative hypothesis: true difference in means is not equal to 0
## 95 percent confidence interval:
##  -0.03752195  0.02552297
## sample estimates:
## mean of the differences
##            -0.005999492
```

```r
cohens_dav(data = x1b_ph1, x = CR, group = Reward_Category)
```

```
## # A tibble: 2 x 4
##   Reward_Category count  mean    sd
##   <fct>           <int> <dbl> <dbl>
## 1 High Reward       120 0.336 0.194
## 2 Low Reward        120 0.342 0.185
## [1] "Effect size Cohen’s d(av):"
## [1] -0.03168304
```

```r
# d-prime (DP)
t.test(data = x1b_ph1, DP ~ Reward_Category, paired = TRUE)
```

```
##
##  Paired t-test
##
## data:  DP by Reward_Category
## t = -0.2134, df = 119, p-value = 0.8314
## alternative hypothesis: true difference in means is not equal to 0
## 95 percent confidence interval:
##  -0.1280889  0.1031662
## sample estimates:
## mean of the differences
##             -0.01246135
```

```r
cohens_dav(data = x1b_ph1, x = DP, group = Reward_Category)
```

```
## # A tibble: 2 x 4
##   Reward_Category count  mean    sd
##   <fct>           <int> <dbl> <dbl>
## 1 High Reward       120  1.68 0.781
## 2 Low Reward        120  1.69 0.694
## [1] "Effect size Cohen's d(av):"
## [1] -0.01690135
```

Further to the repeated measures ANOVA, t-tests revealed no significant evidence for an effect of reward category on corrected recognition, t(119) = -0.38, p = .71, $d_{av}$ = -.03, nor on d-primes, t(119) = -0.21, p = .83, $d_{av}$ = -.01 for items encoded in the pre-conditioning phase,

```
# Effect of reward category on memory in phase 2
# (conditioning)

# Corrected recognition (CR)
t.test(data = x1b_ph2, CR ~ Reward_Category, paired = TRUE)
```

```
##
##  Paired t-test
##
## data:  CR by Reward_Category
## t = 2.3327, df = 119, p-value = 0.02135
## alternative hypothesis: true difference in means is not equal to 0
## 95 percent confidence interval:
##  0.006571674 0.080387674
## sample estimates:
## mean of the differences
##              0.04347967
```

```
cohens_dav(data = x1b_ph2, x = CR, group = Reward_Category)
```

```
## # A tibble: 2 x 4
##   Reward_Category count  mean    sd
##   <fct>           <int> <dbl> <dbl>
## 1 High Reward       120 0.314 0.186
## 2 Low Reward        120 0.271 0.160
## [1] "Effect size Cohen's d(av):"
## [1] 0.2516504
```

```
# d-prime (DP)
t.test(data = x1b_ph2, DP ~ Reward_Category, paired = TRUE)
```

```
##
##  Paired t-test
##
## data:  DP by Reward_Category
## t = 1.9715, df = 119, p-value = 0.05099
## alternative hypothesis: true difference in means is not equal to 0
## 95 percent confidence interval:
##  -0.0006747435  0.3097964210
## sample estimates:
## mean of the differences
##               0.1545608
```

```r
cohens_dav(data = x1b_ph2, x = DP, group = Reward_Category)
```

```
## # A tibble: 2 x 4
##   Reward_Category count  mean    sd
##   <fct>           <int> <dbl> <dbl>
## 1 High Reward       120  1.63 0.742
## 2 Low Reward        120  1.47 0.653
## [1] "Effect size Cohen's d(av):"
## [1] 0.221568
```

For items encoded in the conditioning phase, t-tests revealed a significant evidence for an effect of reward category on corrected recognition, t(119) = 2.33, p = .02, $d_{av}$ = .25, and on d-primes, t(119) = 1.97, p = .05, $d_{av}$ =.22. Although it is only a weakly significant effect, this means that reward conditioning was successful and emerged after a 24 hour post consolidation period, as there was no effect seen in experiment 1a.

```r
# Effect of reward category on memory in phase 3
# (post-conditioning)

# Corrected recognition (CR)
t.test(data = x1b_ph3, CR ~ Reward_Category, paired = TRUE)
```

```
##
##  Paired t-test
##
## data:  CR by Reward_Category
## t = -0.28425, df = 119, p-value = 0.7767
## alternative hypothesis: true difference in means is not equal to 0
## 95 percent confidence interval:
##  -0.04214194  0.03156160
## sample estimates:
## mean of the differences
##            -0.005290167
```

```r
cohens_dav(data = x1b_ph3, x = CR, group = Reward_Category)
```

```
## # A tibble: 2 x 4
##   Reward_Category count  mean    sd
##   <fct>           <int> <dbl> <dbl>
## 1 High Reward       120 0.296 0.213
## 2 Low Reward        120 0.301 0.195
## [1] "Effect size Cohen's d(av):"
## [1] -0.02592615
```

```r
# d-prime (DP)
t.test(data = x1b_ph3, DP ~ Reward_Category, paired = TRUE)
```

```
##
##  Paired t-test
##
## data:  DP by Reward_Category
## t = -0.21108, df = 119, p-value = 0.8332
```

```
## alternative hypothesis: true difference in means is not equal to 0
## 95 percent confidence interval:
##  -0.1872415  0.1511666
## sample estimates:
## mean of the differences
##             -0.01803742
```

```
cohens_dav(data = x1b_ph3, x = DP, group = Reward_Category)
```

```
## # A tibble: 2 x 4
##   Reward_Category count  mean    sd
##   <fct>           <int> <dbl> <dbl>
## 1 High Reward       120  1.53 0.884
## 2 Low Reward        120  1.55 0.820
## [1] "Effect size Cohen's d(av):"
## [1] -0.02117391
```

Further to the repeated measures ANOVA, t-tests revealed no significant evidence for an effect of reward category on corrected recognition, t(119) = -0.28, p = .78, $d_{av}$ = -.01, nor on d-primes, t(119) = -0.21, p = .83, $d_{av}$ = -.02 for items encoded in the post-conditioning phase.

## 1.4 Experiment 1b (High Certainty Memory)

### Corrected recognition (CR) by phase and reward category

```
# Summary table and graph
aggregate(CR ~ Reward_Category + Phase, data.x1b.high, FUN = function(CR) c(mean = mean(CR),
    se = std.error(CR)))
```

```
##   Reward_Category             Phase    CR.mean      CR.se
## 1     High Reward  Pre-conditioning 0.38880533 0.02031628
## 2      Low Reward  Pre-conditioning 0.40016679 0.02019819
## 3     High Reward      Conditioning 0.37164815 0.02149016
## 4      Low Reward      Conditioning 0.32864808 0.01848773
## 5     High Reward Post-conditioning 0.34250803 0.02221628
## 6      Low Reward Post-conditioning 0.35742407 0.01922293
```

```
x1b.high.CR = plot_by_group(data = data.x1b.high, yvar = "CR",
    ylim = c(0, 1.2), ylab = "Corrected Recognition", subtitle = "Experiment 1b (High Certainty)",
    tag = "1.2 A")
x1b.high.CR
```

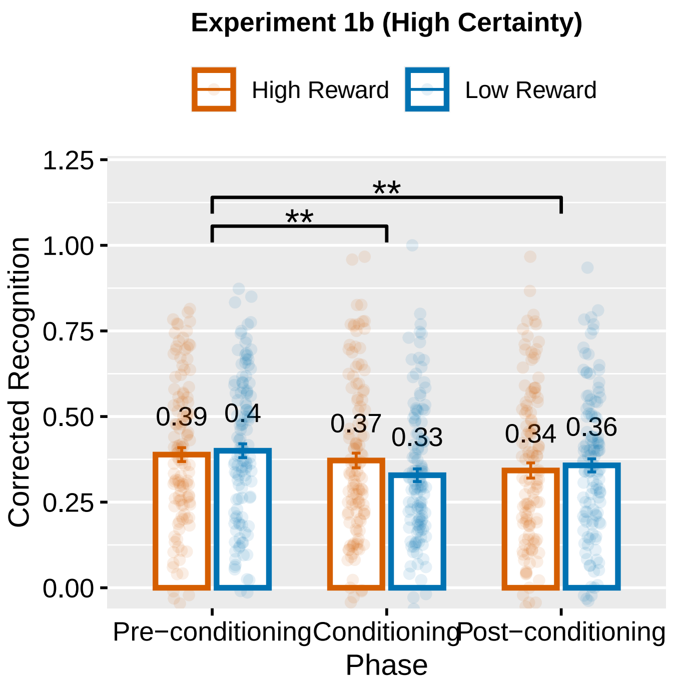


```
# Repeated measures two-factor ANOVA on corrected
# recognition (high certainty only)
anova_test(CR ~ Phase * Reward_Category + Error(UserID/(Phase *
    Reward_Category)), data = data.x1b.high)
```

```
## ANOVA Table (type III tests)
##
## $ANOVA
##                  Effect DFn DFd     F     p p<.05      ges
## 1                 Phase   2 238 5.596 0.004     * 0.009000
## 2       Reward_Category   1 119 0.106 0.745       0.000157
## 3 Phase:Reward_Category   2 238 3.277 0.039     * 0.004000
##
## $`Mauchly's Test for Sphericity`
##                  Effect     W     p p<.05
## 1                 Phase 0.972 0.190
## 2 Phase:Reward_Category 0.927 0.012     *
##
## $`Sphericity Corrections`
##                  Effect   GGe       DF[GG] p[GG] p[GG]<.05   HFe       DF[HF]
## 1                 Phase 0.973 1.95, 231.57 0.005         * 0.989 1.98, 235.36
## 2 Phase:Reward_Category 0.932 1.86, 221.85 0.043         * 0.946 1.89, 225.24
##   p[HF] p[HF]<.05
## 1 0.004         *
## 2 0.042         *
```

The sphericity assumption was not met for the reward category and phase interaction term, W = 0.93, p = .01, and so Greenhouse-Geisser corrections were applied to the results. The repeated measures ANOVA

revealed an effect of phase, F(1,231.57) = 5.60, p = .004, $\eta^2$ = .009, on corrected recognition. There was a significant interaction effect between encoding phase and reward category, F(1,221.85) = 3.28, p = .04, $\eta^2$ = .004, this meant that the effect of reward category (high vs. low) on memory of items differed across encoding phases and this will be analysed further through t-tests.

**d-prime (DP) by phase and reward category**

```
# Summary table and graph
aggregate(DP ~ Reward_Category + Phase, data.x1b.high, FUN = function(DP) c(mean = mean(DP),
    se = std.error(DP)))
```

```
##   Reward_Category             Phase    DP.mean      DP.se
## 1     High Reward  Pre-conditioning 1.98000985 0.08550673
## 2      Low Reward  Pre-conditioning 2.03364624 0.08288401
## 3     High Reward      Conditioning 1.97278902 0.08998575
## 4      Low Reward      Conditioning 1.84507603 0.08026657
## 5     High Reward Post-conditioning 1.80507953 0.10130466
## 6      Low Reward Post-conditioning 1.90286798 0.08517401
```

```
x1b.high.DP = plot_by_group(data = data.x1b.high, yvar = "DP",
    ylim = c(0, 4.6), ylab = "d-prime", subtitle = "Experiment 1b (High Certainty)",
    tag = "1.2 B")
x1b.high.DP
```

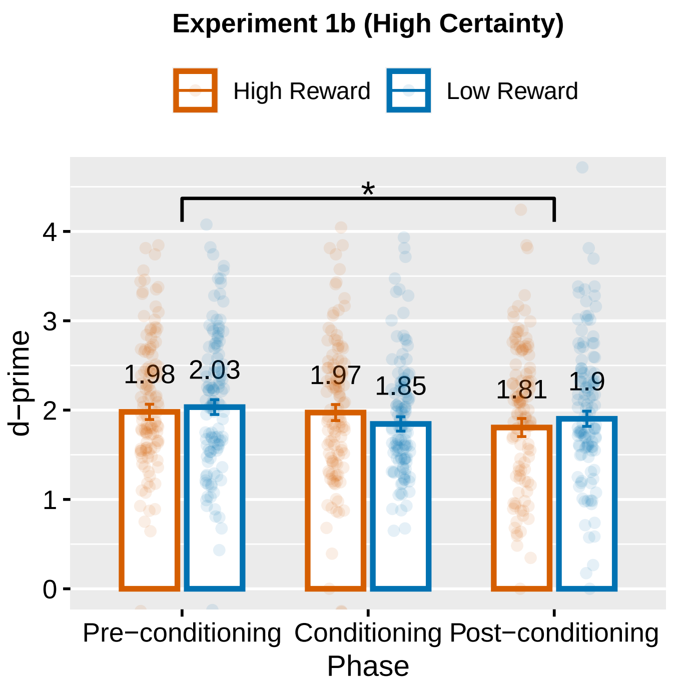


```
# Repeated measures two-factor ANOVA on d-prime scores
# (high certainty only)
anova_test(DP ~ Phase * Reward_Category + Error(UserID/(Phase *
    Reward_Category)), data = data.x1b.high)
```

```
## ANOVA Table (type III tests)
##
## $ANOVA
##                  Effect DFn DFd     F     p p<.05     ges
## 1                 Phase   2 238 2.517 0.083       4.0e-03
## 2       Reward_Category   1 119 0.011 0.918       1.7e-05
## 3 Phase:Reward_Category   2 238 2.332 0.099       3.0e-03
##
## $`Mauchly's Test for Sphericity`
##                  Effect     W     p p<.05
## 1                 Phase 0.989 0.507
## 2 Phase:Reward_Category 0.906 0.003     *
##
## $`Sphericity Corrections`
##                  Effect   GGe       DF[GG] p[GG] p[GG]<.05   HFe       DF[HF]
## 1                 Phase 0.989 1.98, 235.31 0.083           1.005 2.01, 239.26
## 2 Phase:Reward_Category 0.914 1.83, 217.59 0.104           0.928 1.86, 220.81
##   p[HF] p[HF]<.05
## 1 0.083
## 2 0.104
```

The sphericity assumption was not met for the reward category and phase interaction term, W = 0.91, p = .003, and so Greenhouse-Geisser corrections were applied to the results. The repeated measures ANOVA revealed no effect of phase or reward category on d-primes. More importantly, there was no significant interaction effect between encoding phase and reward category, F(1,217.59) = 2.33, p = .04, $\eta^2$ = .004.

```
# Create subsets for each phase from data.x2b (high
# certainty)
x1b_high_ph1 <- subset(data.x1b.high, Phase == "Pre-conditioning")
x1b_high_ph2 <- subset(data.x1b.high, Phase == "Conditioning")
x1b_high_ph3 <- subset(data.x1b.high, Phase == "Post-conditioning")
```

```
# Effect of reward category on high certainty memory in
# phase 1 (pre-conditioning)

# Corrected recognition (CR)
t.test(data = x1b_high_ph1, CR ~ Reward_Category, paired = TRUE)
```

```
##
##  Paired t-test
##
## data:  CR by Reward_Category
## t = -0.56018, df = 119, p-value = 0.5764
## alternative hypothesis: true difference in means is not equal to 0
## 95 percent confidence interval:
##  -0.05152132  0.02879841
## sample estimates:
## mean of the differences
##             -0.01136146
```

```
cohens_dav(data = x1b_high_ph1, x = CR, group = Reward_Category)
```

```
## # A tibble: 2 x 4
```

```
##   Reward_Category count  mean    sd
##   <fct>           <int> <dbl> <dbl>
## 1 High Reward       120 0.389 0.223
## 2 Low Reward        120 0.400 0.221
## [1] "Effect size Cohen's d(av):"
## [1] -0.05119921
```

```r
# d-prime (DP)
t.test(data = x1b_high_ph1, DP ~ Reward_Category, paired = TRUE)
```

```
##
##  Paired t-test
##
## data:  DP by Reward_Category
## t = -0.66708, df = 119, p-value = 0.506
## alternative hypothesis: true difference in means is not equal to 0
## 95 percent confidence interval:
##  -0.2128447  0.1055719
## sample estimates:
## mean of the differences
##             -0.05363639
```

```r
cohens_dav(data = x1b_high_ph1, x = DP, group = Reward_Category)
```

```
## # A tibble: 2 x 4
##   Reward_Category count  mean    sd
##   <fct>           <int> <dbl> <dbl>
## 1 High Reward       120  1.98 0.937
## 2 Low Reward        120  2.03 0.908
## [1] "Effect size Cohen's d(av):"
## [1] -0.05815414
```

For items encoded in the pre-conditioning phase, t-tests revealed no significant evidence for an effect of reward category on corrected recognition, $t(119) = -0.56$, $p = .58$, $d_{av} = -.05$, nor on d-primes, $t(119) = -0.67$, $p = .51$, $d_{av} = -.06$.

```r
# Effect of reward category on high certainty memory in
# phase 2 (conditioning)

# Corrected recognition (CR)
t.test(data = x1b_high_ph2, CR ~ Reward_Category, paired = TRUE)
```

```
##
##  Paired t-test
##
## data:  CR by Reward_Category
## t = 1.6541, df = 119, p-value = 0.1007
## alternative hypothesis: true difference in means is not equal to 0
## 95 percent confidence interval:
##  -0.008474089  0.094474214
## sample estimates:
## mean of the differences
##              0.04300006
```

```r
cohens_dav(data = x1b_high_ph2, x = CR, group = Reward_Category)
```

```
## # A tibble: 2 x 4
##   Reward_Category count  mean    sd
##   <fct>           <int> <dbl> <dbl>
## 1 High Reward       120 0.372 0.235
## 2 Low Reward        120 0.329 0.203
## [1] "Effect size Cohen's d(av):"
## [1] 0.1963761
```

```r
# d-prime (DP)
t.test(data = x1b_high_ph2, DP ~ Reward_Category, paired = TRUE)
```

```
##
##  Paired t-test
##
## data:  DP by Reward_Category
## t = 1.1354, df = 119, p-value = 0.2585
## alternative hypothesis: true difference in means is not equal to 0
## 95 percent confidence interval:
##  -0.09500444  0.35043042
## sample estimates:
## mean of the differences
##                0.127713
```

```r
cohens_dav(data = x1b_high_ph2, x = DP, group = Reward_Category)
```

```
## # A tibble: 2 x 4
##   Reward_Category count  mean    sd
##   <fct>           <int> <dbl> <dbl>
## 1 High Reward       120  1.97 0.986
## 2 Low Reward        120  1.85 0.879
## [1] "Effect size Cohen's d(av):"
## [1] 0.1369561
```

While we saw successful reward conditioning for items in the high reward category when considering all memory trials, t-tests for high certainty memory trials revealed did not show evidence for a significant effect of reward category on corrected recognition, $t(119) = 1.65$, $p = .10$, $d_{av} = .04$, nor on d-primes, $t(119) = 1.14$, $p = .26$, $d_{av} = .14$. Although a weak reward conditioning effect emerged after a 24 hour post consolidation period with all memory trials, this effect disappears when considering high certainty memory and thus it can be largely attributed to guessing behaviour. This casts doubt on the success reward conditioning in this experiment.

```r
# Effect of reward category on high certainty memory in
# phase 3 (post-conditioning)

# Corrected recognition (CR)
t.test(data = x1b_high_ph3, CR ~ Reward_Category, paired = TRUE)
```

```
##
##  Paired t-test
```

```
##
## data:  CR by Reward_Category
## t = -0.71659, df = 119, p-value = 0.475
## alternative hypothesis: true difference in means is not equal to 0
## 95 percent confidence interval:
##  -0.05613220  0.02630012
## sample estimates:
## mean of the differences
##             -0.01491604
```

```
cohens_dav(data = x1b_high_ph3, x = CR, group = Reward_Category)
```

```
## # A tibble: 2 x 4
##   Reward_Category count  mean    sd
##   <fct>           <int> <dbl> <dbl>
## 1 High Reward       120 0.343 0.243
## 2 Low Reward        120 0.357 0.211
## [1] "Effect size Cohen's d(av):"
## [1] -0.06571755
```

```
# d-prime (DP)
t.test(data = x1b_high_ph3, DP ~ Reward_Category, paired = TRUE)
```

```
##
##  Paired t-test
##
## data:  DP by Reward_Category
## t = -0.94621, df = 119, p-value = 0.346
## alternative hypothesis: true difference in means is not equal to 0
## 95 percent confidence interval:
##  -0.3024274  0.1068505
## sample estimates:
## mean of the differences
##             -0.09778845
```

```
cohens_dav(data = x1b_high_ph3, x = DP, group = Reward_Category)
```

```
## # A tibble: 2 x 4
##   Reward_Category count  mean    sd
##   <fct>           <int> <dbl> <dbl>
## 1 High Reward       120  1.81 1.11
## 2 Low Reward        120  1.90 0.933
## [1] "Effect size Cohen's d(av):"
## [1] -0.09574096
```

For items encoded in the post-conditioning phase, t-tests revealed no significant evidence for an effect of reward category on corrected recognition, $t(119) = -0.72$, $p = .48$, $d_{av} = -.07$, nor on d-primes, $t(119) = -0.95$, $p = .35$, $d_{av} = -.10$.

# 2. Complementary Bayesian t-tests

As complementary analysis to classical paired t-tests conducted above, which have resulted in inconclusive evidence for category selective retrospective or prospective memory enhancement effects, we additionally used Bayesian analysis to confirm whether our data supported the null hypothesis of no effect. We used Bayesian paired t-tests using ttestBF function in R, with the alternative hypothesis (H1) supporting a positive memory effect for high reward items compared to low reward items overall and from each phase, whereas the null hypothesis (H0) represents zero effect [Jarosz and Wiley, 2014,Rouder et al., 2009]

Bayes factors were calculated to test whether the null hypothesis H0 (true effect is equal to zero) holds against the one-sided alternative hypothesis H1 (effect is greater than zero). In the below analysis we used a Cauchy prior distribution with a default scale parameter of r = .707 interpreted the Bayes factor ($BF_{10}$) as follows:

- $BF_{10} < 1/3$ : Substantial evidence for H0
- $1/3 < BF_{10} < 1$ : Anecdotal evidence for H0
- $1 < BF_{10} < 3$ : Anecdotal evidence for H1
- $BF_{10} > 3$ : Substantial evidence for H1

## 2.1 Experiment 1a (All Memory)

```r
# Effect of reward category on memory in phase 1
# (pre-conditioning) Corrected recognition (CR) Two-sided
# test
ttestBF(x = x1a_ph1$CR[x1a_ph1$Reward_Category == "High Reward"],
    y = x1a_ph1$CR[x1a_ph1$Reward_Category == "Low Reward"],
    paired = TRUE)
```

```
## Bayes factor analysis
## --------------
## [1] Alt., r=0.707 : 0.5260488 ±0%
##
## Against denominator:
##   Null, mu = 0
## ---
## Bayes factor type: BFoneSample, JZS
```

```r
# One-sided test
ttestBF(x = x1a_ph1$CR[x1a_ph1$Reward_Category == "High Reward"],
    y = x1a_ph1$CR[x1a_ph1$Reward_Category == "Low Reward"],
    nullInterval = c(-Inf, 0), paired = TRUE)
```

```
## Bayes factor analysis
## --------------
## [1] Alt., r=0.707 -Inf<d<0    : 0.03691728 ±0.21%
## [2] Alt., r=0.707 !(-Inf<d<0) : 1.01518    ±0%
##
## Against denominator:
##   Null, mu = 0
## ---
## Bayes factor type: BFoneSample, JZS
```

```r
# d-prime Two-sided test
ttestBF(x = x1a_ph1$DP[x1a_ph1$Reward_Category == "High Reward"],
    y = x1a_ph1$DP[x1a_ph1$Reward_Category == "Low Reward"],
    paired = TRUE)
```

```
## Bayes factor analysis
## --------------
## [1] Alt., r=0.707 : 0.215197 ±0%
##
## Against denominator:
##   Null, mu = 0
## ---
## Bayes factor type: BFoneSample, JZS
```

```r
# One-sided test
ttestBF(x = x1a_ph1$DP[x1a_ph1$Reward_Category == "High Reward"],
    y = x1a_ph1$DP[x1a_ph1$Reward_Category == "Low Reward"],
    nullInterval = c(-Inf, 0), paired = TRUE)
```

```
## Bayes factor analysis
## --------------
## [1] Alt., r=0.707 -Inf<d<0    : 0.04752184 ±0%
## [2] Alt., r=0.707 !(-Inf<d<0) : 0.3828722  ±0%
##
## Against denominator:
##   Null, mu = 0
## ---
## Bayes factor type: BFoneSample, JZS
```

In the pre-conditioning phase of experiment 1a, Bayesian t-tests showed anecdotal evidence that data is more probable under the null hypothesis (H0: that there is no effect of reward category on memory) with $BF_{10} = 0.53$ for corrected recognition and stronger evidence for the H0 for d-prime measures, $BF_{10} = 0.22$. The one-sided t-test with alternative hypothesis only showed anecdotal evidence for a positive effect (in favor of high reward category) with $BF_{10} = 1.01$ when considering corrected recognition. The equivalent analysis with d-primes revealed evidence in favor of H0, $BF_{10} = 0.38$. These results are consistent with the findings from classical t-tests performed in section 1.1 of this document which showed a trend level effect of reward category on corrected recognition but not d-primes.

```r
# Effect of reward category on memory in phase 2
# (conditioning) Corrected recognition (CR) Two-sided test
ttestBF(x = x1a_ph2$CR[x1a_ph2$Reward_Category == "High Reward"],
    y = x1a_ph2$CR[x1a_ph2$Reward_Category == "Low Reward"],
    paired = TRUE)
```

```
## Bayes factor analysis
## --------------
## [1] Alt., r=0.707 : 0.1868772 ±0%
##
## Against denominator:
##   Null, mu = 0
## ---
## Bayes factor type: BFoneSample, JZS
```

```r
# One-sided test
ttestBF(x = x1a_ph2$CR[x1a_ph2$Reward_Category == "High Reward"],
    y = x1a_ph2$CR[x1a_ph2$Reward_Category == "Low Reward"],
    nullInterval = c(-Inf, 0), paired = TRUE)
```

```
## Bayes factor analysis
## --------------
## [1] Alt., r=0.707 -Inf<d<0    : 0.05041064 ±0%
## [2] Alt., r=0.707 !(-Inf<d<0) : 0.3233437  ±0%
##
## Against denominator:
##   Null, mu = 0
## ---
## Bayes factor type: BFoneSample, JZS
```

```r
# d-prime Two-sided test
ttestBF(x = x1a_ph2$DP[x1a_ph2$Reward_Category == "High Reward"],
    y = x1a_ph2$DP[x1a_ph2$Reward_Category == "Low Reward"],
    paired = TRUE)
```

```
## Bayes factor analysis
## --------------
## [1] Alt., r=0.707 : 0.1504002 ±0%
##
## Against denominator:
##   Null, mu = 0
## ---
## Bayes factor type: BFoneSample, JZS
```

```r
# One-sided test
ttestBF(x = x1a_ph2$DP[x1a_ph2$Reward_Category == "High Reward"],
    y = x1a_ph2$DP[x1a_ph2$Reward_Category == "Low Reward"],
    nullInterval = c(-Inf, 0), paired = TRUE)
```

```
## Bayes factor analysis
## --------------
## [1] Alt., r=0.707 -Inf<d<0    : 0.05646522 ±0.06%
## [2] Alt., r=0.707 !(-Inf<d<0) : 0.2443352  ±0%
##
## Against denominator:
##   Null, mu = 0
## ---
## Bayes factor type: BFoneSample, JZS
```

In the conditioning phase of experiment 1a, the Bayes factors suggested substantial evidence in favor of the null hypothesis that there is no memory enhancement for items specifically in the high or low reward category, $BF_{10} = .19$ with corrected recognition and $BF_{10} = .15$ with d-prime scores. The one-sided t-tests also provided substantial evidence for the null hypothesis, all Bayes factors $< 0.33$.

```r
# Effect of reward category on memory in phase 3
# (post-conditioning) Corrected recognition (CR) Two-sided
```

```r
# test
ttestBF(x = x1a_ph3$CR[x1a_ph3$Reward_Category == "High Reward"],
    y = x1a_ph3$CR[x1a_ph3$Reward_Category == "Low Reward"],
    paired = TRUE)
```

```
## Bayes factor analysis
## --------------
## [1] Alt., r=0.707 : 1.286457 ±0%
##
## Against denominator:
##   Null, mu = 0
## ---
## Bayes factor type: BFoneSample, JZS
```

```r
# One-sided test
ttestBF(x = x1a_ph3$CR[x1a_ph3$Reward_Category == "High Reward"],
    y = x1a_ph3$CR[x1a_ph3$Reward_Category == "Low Reward"],
    nullInterval = c(-Inf, 0), paired = TRUE)
```

```
## Bayes factor analysis
## --------------
## [1] Alt., r=0.707 -Inf<d<0    : 0.03150594 ±0.01%
## [2] Alt., r=0.707 !(-Inf<d<0) : 2.541409   ±0%
##
## Against denominator:
##   Null, mu = 0
## ---
## Bayes factor type: BFoneSample, JZS
```

```r
# d-prime Two-sided test
ttestBF(x = x1a_ph3$DP[x1a_ph3$Reward_Category == "High Reward"],
    y = x1a_ph3$DP[x1a_ph3$Reward_Category == "Low Reward"],
    paired = TRUE)
```

```
## Bayes factor analysis
## --------------
## [1] Alt., r=0.707 : 0.3542357 ±0%
##
## Against denominator:
##   Null, mu = 0
## ---
## Bayes factor type: BFoneSample, JZS
```

```r
# One-sided test
ttestBF(x = x1a_ph3$DP[x1a_ph3$Reward_Category == "High Reward"],
    y = x1a_ph3$DP[x1a_ph3$Reward_Category == "Low Reward"],
    nullInterval = c(-Inf, 0), paired = TRUE)
```

```
## Bayes factor analysis
## --------------
## [1] Alt., r=0.707 -Inf<d<0    : 0.0405485 ±0%
```

```
## [2] Alt., r=0.707 !(-Inf<d<0) : 0.6679229 ±0%
##
## Against denominator:
##   Null, mu = 0
## ---
## Bayes factor type: BFoneSample, JZS
```

In the post-conditioning phase of experiment 1a, Bayesian t-tests suggested that data is marginally more probable under the alternative hypothesis (H1: that there is an effect of reward category on memory) with $BF_{10} = 1.29$ for corrected recognition. The one-sided t-test with alternative hypothesis of a positive effect supported this only anecdotally with a $BF_{10} = 2.54$. A parallel analysis with d-primes did not reveal any evidence in support for the alternative hypothesis, $BF_{10} < 1$. These results are consistent with the findings from classical t-tests performed in section 1.1 of this document.

## 2.2 Experiment 1a (High Certainty Memory)

```
# Effect of reward category on high certainty memory in
# phase 1 (pre-conditioning) Corrected recognition (CR)
# Two-sided test
ttestBF(x = x1a_high_ph1$CR[x1a_high_ph1$Reward_Category == "High Reward"],
    y = x1a_high_ph1$CR[x1a_high_ph1$Reward_Category == "Low Reward"],
    paired = TRUE)
```

```
## Bayes factor analysis
## --------------
## [1] Alt., r=0.707 : 0.1601026 ±0%
##
## Against denominator:
##   Null, mu = 0
## ---
## Bayes factor type: BFoneSample, JZS
```

```
# One-sided test
ttestBF(x = x1a_high_ph1$CR[x1a_high_ph1$Reward_Category == "High Reward"],
    y = x1a_high_ph1$CR[x1a_high_ph1$Reward_Category == "Low Reward"],
    nullInterval = c(-Inf, 0), paired = TRUE)
```

```
## Bayes factor analysis
## --------------
## [1] Alt., r=0.707 -Inf<d<0    : 0.05444961 ±0.06%
## [2] Alt., r=0.707 !(-Inf<d<0) : 0.2657555  ±0%
##
## Against denominator:
##   Null, mu = 0
## ---
## Bayes factor type: BFoneSample, JZS
```

```
# d-prime Two-sided test
ttestBF(x = x1a_high_ph1$DP[x1a_high_ph1$Reward_Category == "High Reward"],
    y = x1a_high_ph1$DP[x1a_high_ph1$Reward_Category == "Low Reward"],
    paired = TRUE)
```

```
## Bayes factor analysis
## --------------
## [1] Alt., r=0.707 : 0.1519525 ±0%
##
## Against denominator:
##   Null, mu = 0
## ---
## Bayes factor type: BFoneSample, JZS
```

```r
# One-sided test
ttestBF(x = x1a_high_ph1$DP[x1a_high_ph1$Reward_Category == "High Reward"],
    y = x1a_high_ph1$DP[x1a_high_ph1$Reward_Category == "Low Reward"],
    nullInterval = c(-Inf, 0), paired = TRUE)
```

```
## Bayes factor analysis
## --------------
## [1] Alt., r=0.707 -Inf<d<0    : 0.05611469 ±0.03%
## [2] Alt., r=0.707 !(-Inf<d<0) : 0.2477902  ±0%
##
## Against denominator:
##   Null, mu = 0
## ---
## Bayes factor type: BFoneSample, JZS
```

When considering only high certainty memory in the pre-conditioning phase of experiment 1a, the Bayes factors suggested substantial evidence in favor of the null hypothesis that there is no memory enhancement for items specifically in the high or low reward category, $BF_{10} = .16$ with corrected recognition and $BF_{10} = .15$ with d-prime scores. The one-sided t-tests also provided substantial evidence for the null hypothesis, all Bayes factors $< 0.33$.

```r
# Effect of reward category on high certainty memory in
# phase 2 (conditioning) Corrected recognition (CR)
# Two-sided test
ttestBF(x = x1a_high_ph2$CR[x1a_high_ph2$Reward_Category == "High Reward"],
    y = x1a_high_ph2$CR[x1a_high_ph2$Reward_Category == "Low Reward"],
    paired = TRUE)
```

```
## Bayes factor analysis
## --------------
## [1] Alt., r=0.707 : 0.2637489 ±0%
##
## Against denominator:
##   Null, mu = 0
## ---
## Bayes factor type: BFoneSample, JZS
```

```r
# One-sided test
ttestBF(x = x1a_high_ph2$CR[x1a_high_ph2$Reward_Category == "High Reward"],
    y = x1a_high_ph2$CR[x1a_high_ph2$Reward_Category == "Low Reward"],
    nullInterval = c(-Inf, 0), paired = TRUE)
```

```
## Bayes factor analysis
```

```
## ---------------
## [1] Alt., r=0.707 -Inf<d<0    : 0.04421021 ±0%
## [2] Alt., r=0.707 !(-Inf<d<0) : 0.4832876  ±0%
##
## Against denominator:
##   Null, mu = 0
## ---
## Bayes factor type: BFoneSample, JZS
```

```
# d-prime Two-sided test
ttestBF(x = x1a_high_ph2$DP[x1a_high_ph2$Reward_Category == "High Reward"],
    y = x1a_high_ph2$DP[x1a_high_ph2$Reward_Category == "Low Reward"],
    paired = TRUE)
```

```
## Bayes factor analysis
## ---------------
## [1] Alt., r=0.707 : 0.1728626 ±0%
##
## Against denominator:
##   Null, mu = 0
## ---
## Bayes factor type: BFoneSample, JZS
```

```
# One-sided test
ttestBF(x = x1a_high_ph2$DP[x1a_high_ph2$Reward_Category == "High Reward"],
    y = x1a_high_ph2$DP[x1a_high_ph2$Reward_Category == "Low Reward"],
    nullInterval = c(-Inf, 0), paired = TRUE)
```

```
## Bayes factor analysis
## ---------------
## [1] Alt., r=0.707 -Inf<d<0    : 0.05230521 ±0%
## [2] Alt., r=0.707 !(-Inf<d<0) : 0.29342    ±0%
##
## Against denominator:
##   Null, mu = 0
## ---
## Bayes factor type: BFoneSample, JZS
```

When considering only high certainty memory in the conditioning phase of experiment 1a, the Bayes factors suggested substantial evidence in favor of the null hypothesis that there is no memory enhancement for items specifically in the high or low reward category, $BF_{10} = .26$ with corrected recognition and $BF_{10} = .17$ with d-prime scores. The one-sided t-tests also provided evidence for the null hypothesis, all Bayes factors $< 0.48$.

```
# Effect of reward category on high certainty memory in
# phase 3 (post-conditioning) Corrected recognition (CR)
# Two-sided test
ttestBF(x = x1a_high_ph3$CR[x1a_high_ph3$Reward_Category == "High Reward"],
    y = x1a_high_ph3$CR[x1a_high_ph3$Reward_Category == "Low Reward"],
    paired = TRUE)
```

```
## Bayes factor analysis
## ---------------
```

```
## [1] Alt., r=0.707 : 0.4398938 ±0%
##
## Against denominator:
##   Null, mu = 0
## ---
## Bayes factor type: BFoneSample, JZS
```

```
# One-sided test
ttestBF(x = x1a_high_ph3$CR[x1a_high_ph3$Reward_Category == "High Reward"],
    y = x1a_high_ph3$CR[x1a_high_ph3$Reward_Category == "Low Reward"],
    nullInterval = c(-Inf, 0), paired = TRUE)
```

```
## Bayes factor analysis
## --------------
## [1] Alt., r=0.707 -Inf<d<0    : 0.03842147 ±0%
## [2] Alt., r=0.707 !(-Inf<d<0) : 0.8413661  ±0%
##
## Against denominator:
##   Null, mu = 0
## ---
## Bayes factor type: BFoneSample, JZS
```

```
# d-prime Two-sided test
ttestBF(x = x1a_high_ph3$DP[x1a_high_ph3$Reward_Category == "High Reward"],
    y = x1a_high_ph3$DP[x1a_high_ph3$Reward_Category == "Low Reward"],
    paired = TRUE)
```

```
## Bayes factor analysis
## --------------
## [1] Alt., r=0.707 : 0.2364512 ±0%
##
## Against denominator:
##   Null, mu = 0
## ---
## Bayes factor type: BFoneSample, JZS
```

```
# One-sided test
ttestBF(x = x1a_high_ph3$DP[x1a_high_ph3$Reward_Category == "High Reward"],
    y = x1a_high_ph3$DP[x1a_high_ph3$Reward_Category == "Low Reward"],
    nullInterval = c(-Inf, 0), paired = TRUE)
```

```
## Bayes factor analysis
## --------------
## [1] Alt., r=0.707 -Inf<d<0    : 0.04588566 ±0%
## [2] Alt., r=0.707 !(-Inf<d<0) : 0.4270167  ±0%
##
## Against denominator:
##   Null, mu = 0
## ---
## Bayes factor type: BFoneSample, JZS
```

When considering only high certainty memory in the conditioning phase of experiment 1a, the Bayes factors suggested substantial evidence in favor of the null hypothesis that there is no memory enhancement for items

specifically in the high or low reward category, $BF_{10} = .44$ with corrected recognition and $BF_{10} = .24$ with d-prime scores. The one-sided t-tests also provided evidence for the null hypothesis, all Bayes factors < 1, although some evidence was only anecdotal.

## 2.3 Experiment 1b (All Memory)

```
# Effect of reward category on memory in phase 1
# (pre-conditioning) Corrected recognition (CR) Two-sided
# test
ttestBF(x = x1b_ph1$CR[x1b_ph1$Reward_Category == "High Reward"],
    y = x1b_ph1$CR[x1b_ph1$Reward_Category == "Low Reward"],
    paired = TRUE)
```

```
## Bayes factor analysis
## --------------
## [1] Alt., r=0.707 : 0.1086495 ±0%
##
## Against denominator:
##   Null, mu = 0
## ---
## Bayes factor type: BFoneSample, JZS
```

```
# One-sided test
ttestBF(x = x1b_ph1$CR[x1b_ph1$Reward_Category == "High Reward"],
    y = x1b_ph1$CR[x1b_ph1$Reward_Category == "Low Reward"],
    nullInterval = c(-Inf, 0), paired = TRUE)
```

```
## Bayes factor analysis
## --------------
## [1] Alt., r=0.707 -Inf<d<0    : 0.1401397  ±0%
## [2] Alt., r=0.707 !(-Inf<d<0) : 0.07715937 ±0%
##
## Against denominator:
##   Null, mu = 0
## ---
## Bayes factor type: BFoneSample, JZS
```

```
# d-prime Two-sided test
ttestBF(x = x1b_ph1$DP[x1b_ph1$Reward_Category == "High Reward"],
    y = x1b_ph1$DP[x1b_ph1$Reward_Category == "Low Reward"],
    paired = TRUE)
```

```
## Bayes factor analysis
## --------------
## [1] Alt., r=0.707 : 0.1036496 ±0%
##
## Against denominator:
##   Null, mu = 0
## ---
## Bayes factor type: BFoneSample, JZS
```

```r
# One-sided test
ttestBF(x = x1b_ph1$DP[x1b_ph1$Reward_Category == "High Reward"],
    y = x1b_ph1$DP[x1b_ph1$Reward_Category == "Low Reward"],
    nullInterval = c(-Inf, 0), paired = TRUE)
```

```
## Bayes factor analysis
## --------------
## [1] Alt., r=0.707 -Inf<d<0    : 0.1209286  ±0%
## [2] Alt., r=0.707 !(-Inf<d<0) : 0.08637067 ±0%
##
## Against denominator:
##   Null, mu = 0
## ---
## Bayes factor type: BFoneSample, JZS
```

In the pre-conditioning phase of experiment 1b, the Bayes factors suggested substantial evidence in favor of the null hypothesis that there is no memory enhancement for items specifically in the high or low reward category, $BF_{10} = .11$ with corrected recognition and $BF_{10} = .10$ with d-prime scores. The one-sided t-tests also provided substantial evidence for the null hypothesis, all Bayes factors $< 0.33$.

```r
# Effect of reward category on memory in phase 2
# (conditioning) Corrected recognition (CR) Two-sided test
ttestBF(x = x1b_ph2$CR[x1b_ph2$Reward_Category == "High Reward"],
    y = x1b_ph2$CR[x1b_ph2$Reward_Category == "Low Reward"],
    paired = TRUE)
```

```
## Bayes factor analysis
## --------------
## [1] Alt., r=0.707 : 1.366394 ±0%
##
## Against denominator:
##   Null, mu = 0
## ---
## Bayes factor type: BFoneSample, JZS
```

```r
# One-sided test
ttestBF(x = x1b_ph2$CR[x1b_ph2$Reward_Category == "High Reward"],
    y = x1b_ph2$CR[x1b_ph2$Reward_Category == "Low Reward"],
    nullInterval = c(-Inf, 0), paired = TRUE)
```

```
## Bayes factor analysis
## --------------
## [1] Alt., r=0.707 -Inf<d<0    : 0.03122698 ±0.02%
## [2] Alt., r=0.707 !(-Inf<d<0) : 2.701561   ±0%
##
## Against denominator:
##   Null, mu = 0
## ---
## Bayes factor type: BFoneSample, JZS
```

```r
# d-prime Two-sided test
ttestBF(x = x1b_ph2$DP[x1b_ph2$Reward_Category == "High Reward"],
    y = x1b_ph2$DP[x1b_ph2$Reward_Category == "Low Reward"],
    paired = TRUE)
```

```
## Bayes factor analysis
## --------------
## [1] Alt., r=0.707 : 0.6574278 ±0%
##
## Against denominator:
##   Null, mu = 0
## ---
## Bayes factor type: BFoneSample, JZS
```

```r
# One-sided test
ttestBF(x = x1b_ph2$DP[x1b_ph2$Reward_Category == "High Reward"],
    y = x1b_ph2$DP[x1b_ph2$Reward_Category == "Low Reward"],
    nullInterval = c(-Inf, 0), paired = TRUE)
```

```
## Bayes factor analysis
## --------------
## [1] Alt., r=0.707 -Inf<d<0    : 0.03528741 ±0.09%
## [2] Alt., r=0.707 !(-Inf<d<0) : 1.279568   ±0%
##
## Against denominator:
##   Null, mu = 0
## ---
## Bayes factor type: BFoneSample, JZS
```

In the conditioning phase of experiment 1b, Bayesian t-tests showed anecdotal evidence that data is more probable under the alternate hypothesis (H1: that there is an effect of reward category on memory) with $BF_{10} = 1.37$ for corrected recognition and for the H0 for d-prime measures, $BF_{10} = 0.66$. The one-sided t-test with alternative hypothesis again showed only anecdotal evidence for a positive effect (in favor of high reward category) with $BF_{10} = 2.70$ when considering corrected recognition. The equivalent analysis with d-primes revealed a weaker evidence in favor of H1, $BF_{10} = 1.28$. These results are consistent with the findings from classical t-tests performed in section 1.3 of this document which showed a weakly significant effect of reward category on corrected recognition and trend level effect on d-prime scores.

```r
# Effect of reward category on memory in phase 3
# (post-conditioning) Corrected recognition (CR) Two-sided
# test
ttestBF(x = x1b_ph3$CR[x1b_ph3$Reward_Category == "High Reward"],
    y = x1b_ph3$CR[x1b_ph3$Reward_Category == "Low Reward"],
    paired = TRUE)
```

```
## Bayes factor analysis
## --------------
## [1] Alt., r=0.707 : 0.1054499 ±0%
##
## Against denominator:
##   Null, mu = 0
## ---
## Bayes factor type: BFoneSample, JZS
```

```r
# One-sided test
ttestBF(x = x1b_ph3$CR[x1b_ph3$Reward_Category == "High Reward"],
    y = x1b_ph3$CR[x1b_ph3$Reward_Category == "Low Reward"],
    nullInterval = c(-Inf, 0), paired = TRUE)
```

```
## Bayes factor analysis
## --------------
## [1] Alt., r=0.707 -Inf<d<0    : 0.1287314  ±0.09%
## [2] Alt., r=0.707 !(-Inf<d<0) : 0.08216845 ±0%
##
## Against denominator:
##   Null, mu = 0
## ---
## Bayes factor type: BFoneSample, JZS
```

```r
# d-prime Two-sided test
ttestBF(x = x1b_ph3$DP[x1b_ph3$Reward_Category == "High Reward"],
    y = x1b_ph3$DP[x1b_ph3$Reward_Category == "Low Reward"],
    paired = TRUE)
```

```
## Bayes factor analysis
## --------------
## [1] Alt., r=0.707 : 0.1035999 ±0%
##
## Against denominator:
##   Null, mu = 0
## ---
## Bayes factor type: BFoneSample, JZS
```

```r
# One-sided test
ttestBF(x = x1b_ph3$DP[x1b_ph3$Reward_Category == "High Reward"],
    y = x1b_ph3$DP[x1b_ph3$Reward_Category == "Low Reward"],
    nullInterval = c(-Inf, 0), paired = TRUE)
```

```
## Bayes factor analysis
## --------------
## [1] Alt., r=0.707 -Inf<d<0    : 0.1206858  ±0%
## [2] Alt., r=0.707 !(-Inf<d<0) : 0.08651393 ±0%
##
## Against denominator:
##   Null, mu = 0
## ---
## Bayes factor type: BFoneSample, JZS
```

In the post-conditioning phase of experiment 1b, the Bayes factors suggested substantial evidence in favor of the null hypothesis that there is no memory enhancement for items specifically in the high or low reward category, $BF_{10} = .11$ with corrected recognition and $BF_{10} = .10$ with d-prime scores. The one-sided t-tests also provided substantial evidence for the null hypothesis, all Bayes factors $< 0.33$.

## 2.4 Experiment 1b (High Certainty Memory)

```
# Effect of reward category on high certainty memory in
# phase 1 (pre-conditioning) Corrected recognition (CR)
# Two-sided test
ttestBF(x = x1b_high_ph1$CR[x1b_high_ph1$Reward_Category == "High Reward"],
    y = x1b_high_ph1$CR[x1b_high_ph1$Reward_Category == "Low Reward"],
    paired = TRUE)
```

```
## Bayes factor analysis
## --------------
## [1] Alt., r=0.707 : 0.1181446 ±0%
##
## Against denominator:
##   Null, mu = 0
## ---
## Bayes factor type: BFoneSample, JZS
```

```
# One-sided test
ttestBF(x = x1b_high_ph1$CR[x1b_high_ph1$Reward_Category == "High Reward"],
    y = x1b_high_ph1$CR[x1b_high_ph1$Reward_Category == "Low Reward"],
    nullInterval = c(-Inf, 0), paired = TRUE)
```

```
## Bayes factor analysis
## --------------
## [1] Alt., r=0.707 -Inf<d<0    : 0.1676706  ±0%
## [2] Alt., r=0.707 !(-Inf<d<0) : 0.06861871 ±0%
##
## Against denominator:
##   Null, mu = 0
## ---
## Bayes factor type: BFoneSample, JZS
```

```
# d-prime Two-sided test
ttestBF(x = x1b_high_ph1$DP[x1b_high_ph1$Reward_Category == "High Reward"],
    y = x1b_high_ph1$DP[x1b_high_ph1$Reward_Category == "Low Reward"],
    paired = TRUE)
```

```
## Bayes factor analysis
## --------------
## [1] Alt., r=0.707 : 0.1259418 ±0%
##
## Against denominator:
##   Null, mu = 0
## ---
## Bayes factor type: BFoneSample, JZS
```

```
# One-sided test
ttestBF(x = x1b_high_ph1$DP[x1b_high_ph1$Reward_Category == "High Reward"],
    y = x1b_high_ph1$DP[x1b_high_ph1$Reward_Category == "Low Reward"],
    nullInterval = c(-Inf, 0), paired = TRUE)
```

```
## Bayes factor analysis
## --------------
## [1] Alt., r=0.707 -Inf<d<0    : 0.1875385  ±0%
## [2] Alt., r=0.707 !(-Inf<d<0) : 0.06434516 ±0%
##
## Against denominator:
##   Null, mu = 0
## ---
## Bayes factor type: BFoneSample, JZS
```

With high certainty memory in the pre-conditioning phase of experiment 1b, the Bayes factors suggested substantial evidence in favor of the null hypothesis that there is no memory enhancement for items specifically in the high or low reward category, $BF_{10} = .12$ with corrected recognition and $BF_{10} = .13$ with d-prime scores. The one-sided t-tests also provided substantial evidence for the null hypothesis, all Bayes factors $< 0.33$.

```r
# Effect of reward category on high certainty memory in
# phase 2 (conditioning) Corrected recognition (CR)
# Two-sided test
ttestBF(x = x1b_high_ph2$CR[x1b_high_ph2$Reward_Category == "High Reward"],
    y = x1b_high_ph2$CR[x1b_high_ph2$Reward_Category == "Low Reward"],
    paired = TRUE)
```

```
## Bayes factor analysis
## --------------
## [1] Alt., r=0.707 : 0.3802972 ±0%
##
## Against denominator:
##   Null, mu = 0
## ---
## Bayes factor type: BFoneSample, JZS
```

```r
# One-sided test
ttestBF(x = x1b_high_ph2$CR[x1b_high_ph2$Reward_Category == "High Reward"],
    y = x1b_high_ph2$CR[x1b_high_ph2$Reward_Category == "Low Reward"],
    nullInterval = c(-Inf, 0), paired = TRUE)
```

```
## Bayes factor analysis
## --------------
## [1] Alt., r=0.707 -Inf<d<0    : 0.03980855 ±0%
## [2] Alt., r=0.707 !(-Inf<d<0) : 0.7207858  ±0%
##
## Against denominator:
##   Null, mu = 0
## ---
## Bayes factor type: BFoneSample, JZS
```

```r
# d-prime Two-sided test
ttestBF(x = x1b_high_ph2$DP[x1b_high_ph2$Reward_Category == "High Reward"],
    y = x1b_high_ph2$DP[x1b_high_ph2$Reward_Category == "Low Reward"],
    paired = TRUE)
```

```
## Bayes factor analysis
## --------------
## [1] Alt., r=0.707 : 0.1896994 ±0%
##
## Against denominator:
##   Null, mu = 0
## ---
## Bayes factor type: BFoneSample, JZS
```

```
# One-sided test
ttestBF(x = x1b_high_ph2$DP[x1b_high_ph2$Reward_Category == "High Reward"],
    y = x1b_high_ph2$DP[x1b_high_ph2$Reward_Category == "Low Reward"],
    nullInterval = c(-Inf, 0), paired = TRUE)
```

```
## Bayes factor analysis
## --------------
## [1] Alt., r=0.707 -Inf<d<0    : 0.0500737 ±0%
## [2] Alt., r=0.707 !(-Inf<d<0) : 0.329325  ±0%
##
## Against denominator:
##   Null, mu = 0
## ---
## Bayes factor type: BFoneSample, JZS
```

In the conditioning phase, we found some anecdotal evidence for a positive effect of reward on corrected recognition in the analysis with all memory trials. High certainty memory analysis revealed marginal evidence in favor of the null hypothesis, $BF_{10} = .72$ with corrected recognition. The parallel analysis with d-primes revealed more substantial evidence in favour of H0, $BF_{10} < .33$, thus casting doubt on the robustness of the conditioning achieved in this phase, although there was some evidence when analysing all the memory trials. These results follow the same pattern as the main analysis with ANOVA and t-tests in section 1.4.

```
# Effect of reward category on high certainty memory in
# phase 3 (post-conditioning) Corrected recognition (CR)
# Two-sided test
ttestBF(x = x1b_high_ph3$CR[x1b_high_ph3$Reward_Category == "High Reward"],
    y = x1b_high_ph3$CR[x1b_high_ph3$Reward_Category == "Low Reward"],
    paired = TRUE)
```

```
## Bayes factor analysis
## --------------
## [1] Alt., r=0.707 : 0.1302119 ±0%
##
## Against denominator:
##   Null, mu = 0
## ---
## Bayes factor type: BFoneSample, JZS
```

```
# One-sided test
ttestBF(x = x1b_high_ph3$CR[x1b_high_ph3$Reward_Category == "High Reward"],
    y = x1b_high_ph3$CR[x1b_high_ph3$Reward_Category == "Low Reward"],
    nullInterval = c(-Inf, 0), paired = TRUE)
```

```
## Bayes factor analysis
## --------------
## [1] Alt., r=0.707 -Inf<d<0    : 0.1979067  ±0%
## [2] Alt., r=0.707 !(-Inf<d<0) : 0.06251719 ±0%
##
## Against denominator:
##   Null, mu = 0
## ---
## Bayes factor type: BFoneSample, JZS
```

```
# d-prime Two-sided test
ttestBF(x = x1b_high_ph3$DP[x1b_high_ph3$Reward_Category == "High Reward"],
    y = x1b_high_ph3$DP[x1b_high_ph3$Reward_Category == "Low Reward"],
    paired = TRUE)
```

```
## Bayes factor analysis
## --------------
## [1] Alt., r=0.707 : 0.1567506 ±0%
##
## Against denominator:
##   Null, mu = 0
## ---
## Bayes factor type: BFoneSample, JZS
```

```
# One-sided test
ttestBF(x = x1b_high_ph3$DP[x1b_high_ph3$Reward_Category == "High Reward"],
    y = x1b_high_ph3$DP[x1b_high_ph3$Reward_Category == "Low Reward"],
    nullInterval = c(-Inf, 0), paired = TRUE)
```

```
## Bayes factor analysis
## --------------
## [1] Alt., r=0.707 -Inf<d<0    : 0.2583999  ±0%
## [2] Alt., r=0.707 !(-Inf<d<0) : 0.05510139 ±0.01%
##
## Against denominator:
##   Null, mu = 0
## ---
## Bayes factor type: BFoneSample, JZS
```

With high certainty memory in the post-conditioning phase of experiment 1b, the Bayes factors suggested substantial evidence in favor of the null hypothesis that there is no memory enhancement for items specifically in the high or low reward category, $BF_{10} = .13$ with corrected recognition and $BF_{10} = .16$ with d-prime scores. The one-sided t-tests also provided substantial evidence for the null hypothesis, all Bayes factors $<$ 0.33.

# 3. Summary Graphs & Tables

## 3.1 Memory Performance Graphs

```r
x1a.CR = plot_by_group(data = data.x1a, yvar = "CR", ylim = c(0,
    1.2), ylab = "Corrected Recognition", xlab = NULL, subtitle = "All Memory",
    tag = "A")
x1a.high.CR = plot_by_group(data = data.x1a.high, yvar = "CR",
    ylim = c(0, 1.2), ylab = "Corrected Recognition", xlab = NULL,
    subtitle = "High Certainty Memory", tag = "B")
x1a.DP = plot_by_group(data = data.x1a, yvar = "DP", ylim = c(0,
    4.6), ylab = "d-prime", xlab = NULL, subtitle = "All Memory",
    tag = "C")
x1a.high.DP = plot_by_group(data = data.x1a.high, yvar = "DP",
    ylim = c(0, 4.6), ylab = "d-prime", xlab = NULL, subtitle = "High Certainty Memory",
    tag = "D")

summary.x1a <- ggarrange(x1a.CR, x1a.high.CR, x1a.DP, x1a.high.DP,
    ncol = 2, nrow = 2, common.legend = TRUE, legend = "top")
summary.x1a
```

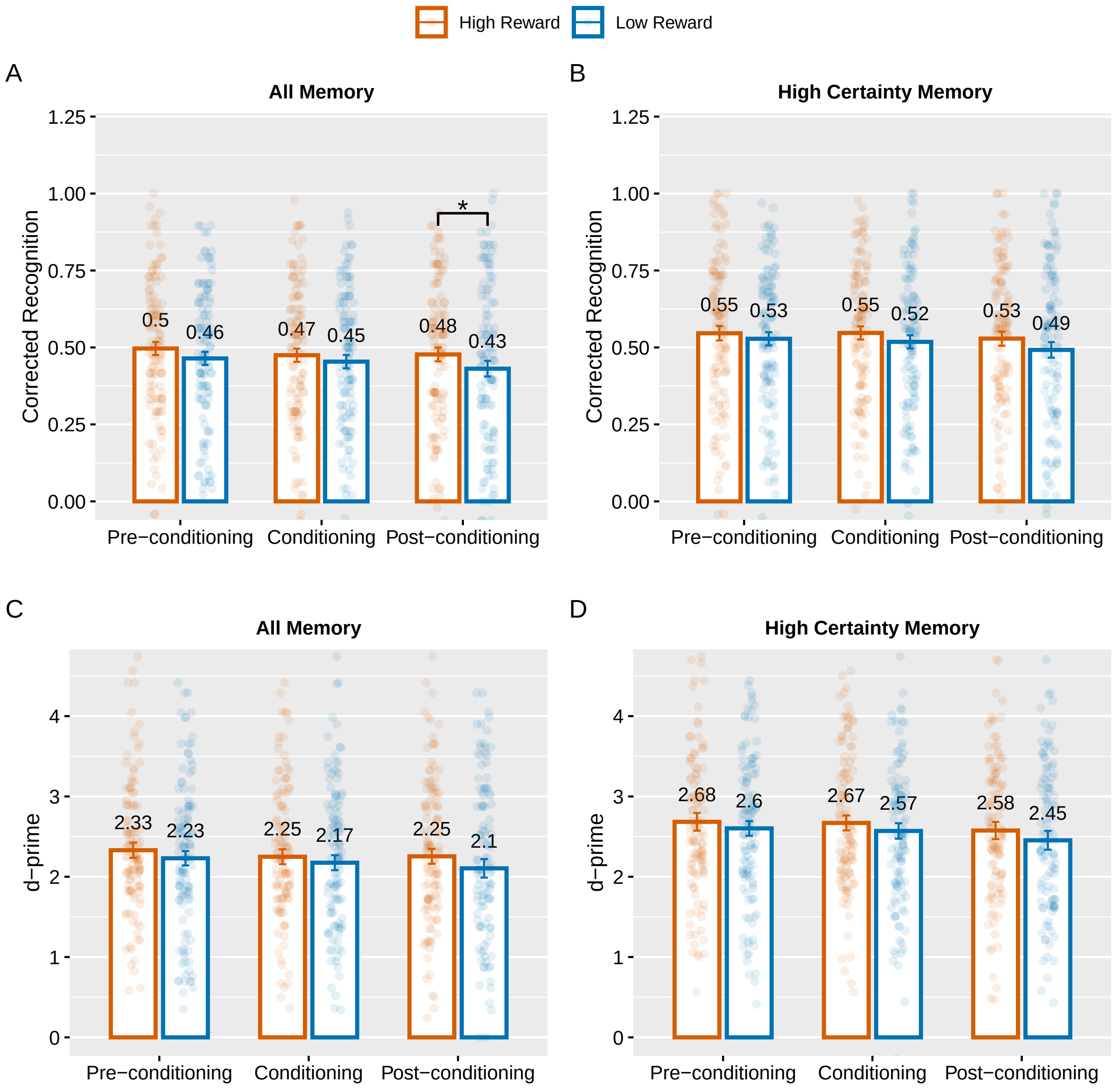


```
ggsave(file = "summary.x1a.svg", plot = summary.x1a, width = 8,
    height = 6.5)
ggsave(file = "summary.x1a.jpg", plot = summary.x1a, width = 8,
    height = 6.5)
```

```
# black and white figures
x1a.CR = plot_by_group_bw(data = data.x1a, yvar = "CR", ylim = c(0,
    1.2), ylab = "Corrected Recognition", xlab = NULL, subtitle = "All Memory",
    tag = "A")
x1a.high.CR = plot_by_group_bw(data = data.x1a.high, yvar = "CR",
    ylim = c(0, 1.2), ylab = "Corrected Recognition", xlab = NULL,
```

```
    subtitle = "High Certainty Memory", tag = "B")
x1a.DP = plot_by_group_bw(data = data.x1a, yvar = "DP", ylim = c(0,
    4.6), ylab = "d-prime", xlab = NULL, subtitle = "All Memory",
    tag = "C")
x1a.high.DP = plot_by_group_bw(data = data.x1a.high, yvar = "DP",
    ylim = c(0, 4.6), ylab = "d-prime", xlab = NULL, subtitle = "High Certainty Memory",
    tag = "D")

bw.summary.x1a <- ggarrange(x1a.CR, x1a.high.CR, x1a.DP, x1a.high.DP,
    ncol = 2, nrow = 2, common.legend = TRUE, legend = "top")


ggsave(file = "bw.summary.x1a.svg", plot = bw.summary.x1a, width = 8,
    height = 6.5)
ggsave(file = "bw.summary.x1a.jpg", plot = bw.summary.x1a, width = 8,
    height = 6.5)
```

```
x1b.CR = plot_by_group(data = data.x1b, yvar = "CR", ylim = c(0,
    1.2), ylab = "Corrected Recognition", xlab = NULL, subtitle = "All Memory",
    tag = "A")
x1b.high.CR = plot_by_group(data = data.x1b.high, yvar = "CR",
    ylim = c(0, 1.2), ylab = "Corrected Recognition", xlab = NULL,
    subtitle = "High Certainty Memory", tag = "B")
x1b.DP = plot_by_group(data = data.x1b, yvar = "DP", ylim = c(0,
    4.6), ylab = "d-prime", xlab = NULL, subtitle = "All Memory",
    tag = "C")
x1b.high.DP = plot_by_group(data = data.x1b.high, yvar = "DP",
    ylim = c(0, 4.6), ylab = "d-prime", xlab = NULL, subtitle = "High Certainty Memory",
    tag = "D")

summary.x1b <- ggarrange(x1b.CR, x1b.high.CR, x1b.DP, x1b.high.DP,
    ncol = 2, nrow = 2, common.legend = TRUE, legend = "top")
summary.x1b
```

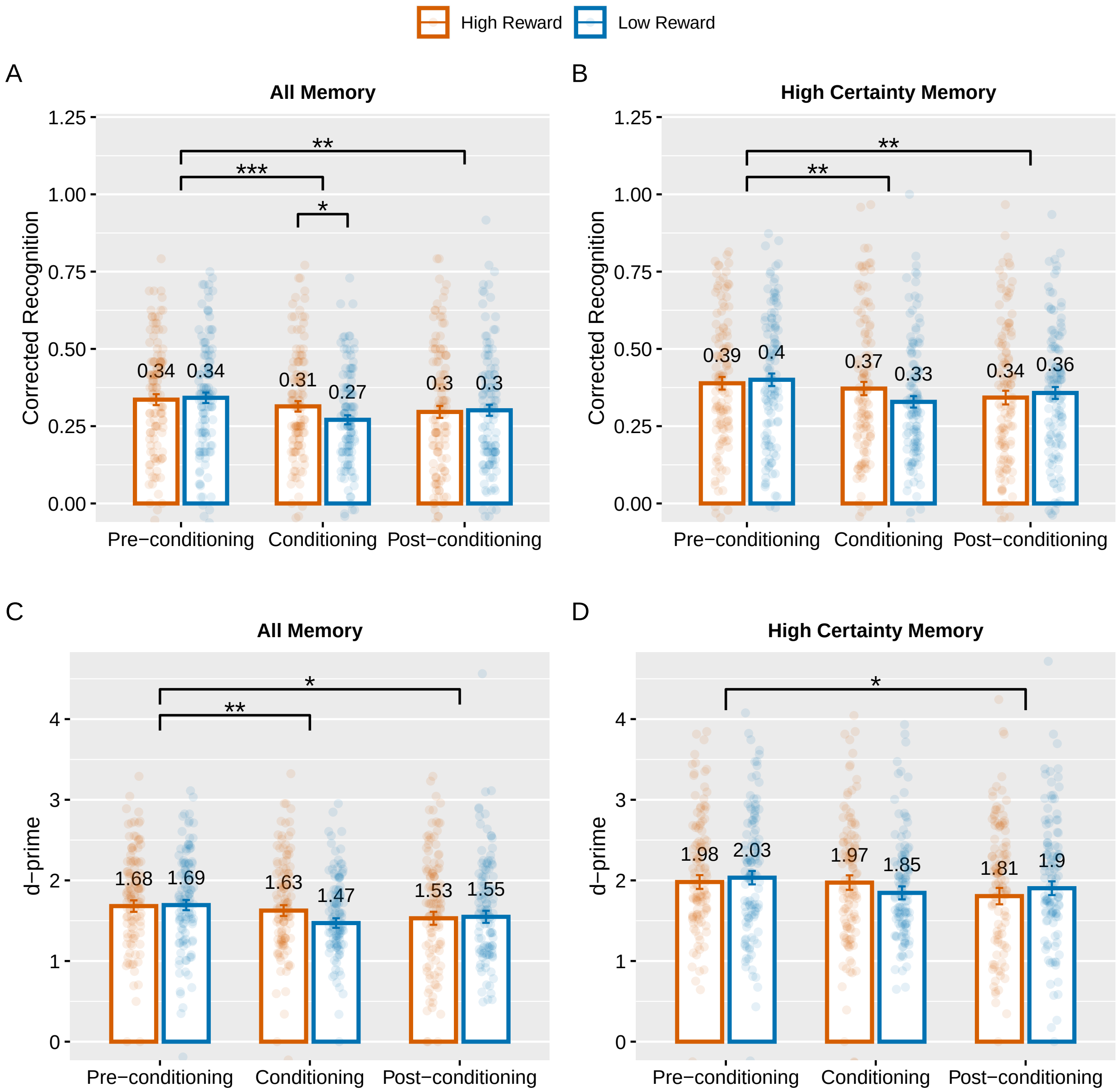


```
ggsave(file = "summary.x1b.svg", plot = summary.x1b, width = 8,
    height = 6.5)
ggsave(file = "summary.x1b.jpg", plot = summary.x1b, width = 8,
    height = 6.5)
```

```
# black and white figures
x1b.CR = plot_by_group_bw(data = data.x1b, yvar = "CR", ylim = c(0,
    1.2), ylab = "Corrected Recognition", xlab = NULL, subtitle = "All Memory",
    tag = "A")
x1b.high.CR = plot_by_group_bw(data = data.x1b.high, yvar = "CR",
    ylim = c(0, 1.2), ylab = "Corrected Recognition", xlab = NULL,
```

```
    subtitle = "High Certainty Memory", tag = "B")
x1b.DP = plot_by_group_bw(data = data.x1b, yvar = "DP", ylim = c(0,
    4.6), ylab = "d-prime", xlab = NULL, subtitle = "All Memory",
    tag = "C")
x1b.high.DP = plot_by_group_bw(data = data.x1b.high, yvar = "DP",
    ylim = c(0, 4.6), ylab = "d-prime", xlab = NULL, subtitle = "High Certainty Memory",
    tag = "D")

bw.summary.x1b <- ggarrange(x1b.CR, x1b.high.CR, x1b.DP, x1b.high.DP,
    ncol = 2, nrow = 2, common.legend = TRUE, legend = "top")

ggsave(file = "bw.summary.x1b.svg", plot = bw.summary.x1b, width = 8,
    height = 6.5)
ggsave(file = "bw.summary.x1b.jpg", plot = bw.summary.x1b, width = 8,

    height = 6.5)
```

### 3.2 Memory Performance by Certainty

Create tables to see how memory responses vary by certainty, coded: 0 = definitely old; 12 = likely old; 24 = maybe old; 48 = maybe new; 60 = likely new, 72 = definitely new.

```
# Experiment 1a
data.cert.x1a <- read.csv("Exp1a_CleanData/Supp/x1a_Certainty.csv")
ph1_hr <- subset(data.cert.x1a, Phase == "1" & Reward_Category ==
    "1") %>%
    group_by(Certainty) %>%
    summarize(mean_size = mean(Proportion, na.rm = TRUE))
ph1_lr <- subset(data.cert.x1a, Phase == "1" & Reward_Category ==
    "-1") %>%
    group_by(Certainty) %>%
    summarize(mean_size = mean(Proportion, na.rm = TRUE))
ph2_hr <- subset(data.cert.x1a, Phase == "2" & Reward_Category ==
    "1") %>%
    group_by(Certainty) %>%
    summarize(mean_size = mean(Proportion, na.rm = TRUE))
ph2_lr <- subset(data.cert.x1a, Phase == "2" & Reward_Category ==
    "-1") %>%
    group_by(Certainty) %>%
    summarize(mean_size = mean(Proportion, na.rm = TRUE))
ph3_hr <- subset(data.cert.x1a, Phase == "3" & Reward_Category ==
    "1") %>%
    group_by(Certainty) %>%
    summarize(mean_size = mean(Proportion, na.rm = TRUE))
ph3_lr <- subset(data.cert.x1a, Phase == "3" & Reward_Category ==
    "-1") %>%
    group_by(Certainty) %>%
    summarize(mean_size = mean(Proportion, na.rm = TRUE))
new_hr <- subset(data.cert.x1a, Phase == "New" & Reward_Category ==
    "1") %>%
    group_by(Certainty) %>%
    summarize(mean_size = mean(Proportion, na.rm = TRUE))
```

```r
new_lr <- subset(data.cert.x1a, Phase == "New" & Reward_Category ==
    "-1") %>%
    group_by(Certainty) %>%
    summarize(mean_size = mean(Proportion, na.rm = TRUE))
```

```r
# Experiment 1b
data.cert.x1b <- read.csv("Exp1b_CleanData/Supp/x1b_Certainty.csv")
ph1_hr <- subset(data.cert.x1b, Phase == "1" & Reward_Category ==
    "1") %>%
    group_by(Certainty) %>%
    summarize(mean_size = mean(Proportion, na.rm = TRUE))
ph1_lr <- subset(data.cert.x1b, Phase == "1" & Reward_Category ==
    "-1") %>%
    group_by(Certainty) %>%
    summarize(mean_size = mean(Proportion, na.rm = TRUE))
ph2_hr <- subset(data.cert.x1b, Phase == "2" & Reward_Category ==
    "1") %>%
    group_by(Certainty) %>%
    summarize(mean_size = mean(Proportion, na.rm = TRUE))
ph2_lr <- subset(data.cert.x1b, Phase == "2" & Reward_Category ==
    "-1") %>%
    group_by(Certainty) %>%
    summarize(mean_size = mean(Proportion, na.rm = TRUE))
ph3_hr <- subset(data.cert.x1b, Phase == "3" & Reward_Category ==
    "1") %>%
    group_by(Certainty) %>%
    summarize(mean_size = mean(Proportion, na.rm = TRUE))
ph3_lr <- subset(data.cert.x1b, Phase == "3" & Reward_Category ==
    "-1") %>%
    group_by(Certainty) %>%
    summarize(mean_size = mean(Proportion, na.rm = TRUE))
new_hr <- subset(data.cert.x1b, Phase == "New" & Reward_Category ==
    "1") %>%
    group_by(Certainty) %>%
    summarize(mean_size = mean(Proportion, na.rm = TRUE))
new_lr <- subset(data.cert.x1b, Phase == "New" & Reward_Category ==
    "-1") %>%
    group_by(Certainty) %>%
    summarize(mean_size = mean(Proportion, na.rm = TRUE))
```

# 4. Supplementary

## 4.1 Performance on Guessing Task (During Encoding)

As part of control analyses, performance on the guessing tasks were summarised and analysed for any biases between treatment groups. We tested whether there were significant differences in matching performance between items from different stimuli categories (animal vs. object) and reward categories (high vs. low).

**Experiment 1a**

We conducted paired t-tests and ANOVA to test whether guessing accuracy in each phase varied by stimuli category (animal vs. objects), and whether guessing accuracy for items from the conditioning phase varied with reward category (high vs. low reward).

```
# Repeated measures ANOVA on memory by phase and category
anova_test(MA ~ Phase * Category + Error(UserID/(Phase * Category)),
    data = data.x1a)
```

```
## ANOVA Table (type III tests)
##
## $ANOVA
##           Effect DFn DFd     F     p p<.05      ges
## 1          Phase   2 238 0.816 0.443       0.002000
## 2       Category   1 119 2.360 0.127       0.003000
## 3 Phase:Category   2 238 0.050 0.951       0.000134
##
## $`Mauchly's Test for Sphericity`
##           Effect     W     p p<.05
## 1          Phase 0.998 0.912
## 2 Phase:Category 0.956 0.070
##
## $`Sphericity Corrections`
##           Effect   GGe       DF[GG] p[GG] p[GG]<.05   HFe       DF[HF] p[HF]
## 1          Phase 0.998    2, 237.63 0.443           1.015 2.03, 241.68 0.443
## 2 Phase:Category 0.958 1.92, 227.97 0.946           0.973 1.95, 231.62 0.948
##   p[HF]<.05
## 1
## 2
```

```
# Matching accuracy by categories (animal vs. objects)
aggregate(MA ~ Category + Phase, data.x1a, FUN = function(MA) c(mean = mean(MA),
    se = std.error(MA)))
```

```
##   Category             Phase    MA.mean      MA.se
## 1   Animal  Pre-conditioning 0.53802083 0.01233912
## 2   Object  Pre-conditioning 0.52552083 0.01220986
## 3   Animal      Conditioning 0.55104167 0.01321037
## 4   Object      Conditioning 0.53802083 0.01195631
## 5   Animal Post-conditioning 0.54479167 0.01036005
## 6   Object Post-conditioning 0.52552083 0.01284315
```

```
# Phase 1 (pre-conditioning)
t.test(data = x1a_ph1, MA ~ Category, paired = TRUE)
```

```
##
##  Paired t-test
##
## data:  MA by Category
## t = 0.73784, df = 119, p-value = 0.4621
## alternative hypothesis: true difference in means is not equal to 0
```

```
## 95 percent confidence interval:
##  -0.02104534  0.04604534
## sample estimates:
## mean of the differences
##                  0.0125
```

```r
cohens_dav(data = x1a_ph1, x = MA, group = Category)
```

```
## # A tibble: 2 x 4
##   Category count  mean    sd
##   <chr>    <int> <dbl> <dbl>
## 1 Animal     120 0.538 0.135
## 2 Object     120 0.526 0.134
## [1] "Effect size Cohen's d(av):"
## [1] 0.09296422
```

```r
# Phase 2 (conditioning)
t.test(data = x1a_ph2, MA ~ Category, paired = TRUE)
```

```
##
##  Paired t-test
##
## data:  MA by Category
## t = 0.76984, df = 119, p-value = 0.4429
## alternative hypothesis: true difference in means is not equal to 0
## 95 percent confidence interval:
##  -0.02046997  0.04651164
## sample estimates:
## mean of the differences
##              0.01302083
```

```r
cohens_dav(data = x1a_ph2, x = MA, group = Category)
```

```
## # A tibble: 2 x 4
##   Category count  mean    sd
##   <chr>    <int> <dbl> <dbl>
## 1 Animal     120 0.551 0.145
## 2 Object     120 0.538 0.131
## [1] "Effect size Cohen's d(av):"
## [1] 0.09446094
```

```r
# Phase 3 (post-conditioning)
t.test(data = x1a_ph3, MA ~ Category, paired = TRUE)
```

```
##
##  Paired t-test
##
## data:  MA by Category
## t = 1.1517, df = 119, p-value = 0.2518
## alternative hypothesis: true difference in means is not equal to 0
## 95 percent confidence interval:
```

```
##  -0.01386260  0.05240426
## sample estimates:
## mean of the differences
##              0.01927083
```

```r
cohens_dav(data = x1a_ph3, x = MA, group = Category)
```

```
## # A tibble: 2 x 4
##   Category count  mean    sd
##   <chr>    <int> <dbl> <dbl>
## 1 Animal     120 0.545 0.113
## 2 Object     120 0.526 0.141
## [1] "Effect size Cohen's d(av):"
## [1] 0.1516324
```

The ANOVA showed no evidence for a significant phase or stimuli category on matching accuracy, all p-values > 0.13. The follow up t-tests also confirmed this, all p-values > .25.

Furthermore, we checked how matching accuracy and reaction time in each phase varies by reward category (high vs. low).

```r
# Matching accuracy by reward category (high vs. low) Phase
# 2 (conditioning)
t.test(data = x1a_ph2, MA ~ Reward_Category, paired = TRUE)
```

```
##
##  Paired t-test
##
## data:  MA by Reward_Category
## t = -1.3935, df = 119, p-value = 0.1661
## alternative hypothesis: true difference in means is not equal to 0
## 95 percent confidence interval:
##  -0.056740973  0.009865973
## sample estimates:
## mean of the differences
##              -0.0234375
```

```r
cohens_dav(data = x1a_ph2, x = MA, group = Reward_Category)
```

```
## # A tibble: 2 x 4
##   Reward_Category count  mean    sd
##   <fct>           <int> <dbl> <dbl>
## 1 High Reward       120 0.533 0.129
## 2 Low Reward        120 0.556 0.146
## [1] "Effect size Cohen's d(av):"
## [1] -0.1705941
```

Matching accuracy did not significantly differ between item reward categories (high vs. low), thus suggesting that equal attention was paid to all items, regardless of reward category, during encoding.

```r
# Repeated measures ANOVA on matching accuracy by phase and
# reward category
anova_test(MA ~ Phase * Reward_Category + Error(UserID/(Phase *
    Reward_Category)), data = data.x1a)
```

```
## ANOVA Table (type III tests)
##
## $ANOVA
##                    Effect DFn DFd     F     p p<.05      ges
## 1                   Phase   2 238 0.816 0.443       0.002000
## 2         Reward_Category   1 119 0.363 0.548       0.000493
## 3 Phase:Reward_Category   2 238 1.146 0.320       0.003000
##
## $`Mauchly's Test for Sphericity`
##                    Effect     W     p p<.05
## 1                   Phase 0.998 0.912
## 2 Phase:Reward_Category 0.956 0.070
##
## $`Sphericity Corrections`
##                    Effect   GGe       DF[GG] p[GG] p[GG]<.05   HFe       DF[HF]
## 1                   Phase 0.998    2, 237.63 0.443           1.015 2.03, 241.68
## 2 Phase:Reward_Category 0.958 1.92, 227.93 0.318           0.973 1.95, 231.58
##   p[HF] p[HF]<.05
## 1 0.443
## 2 0.319
```

```r
# Repeated measures ANOVA on reaction time by phase and
# reward category
anova_test(RT ~ Phase * Reward_Category + Error(UserID/(Phase *
    Reward_Category)), data = data.x1a)
```

```
## ANOVA Table (type III tests)
##
## $ANOVA
##                    Effect DFn DFd      F        p p<.05   ges
## 1                   Phase   2 238 69.321 1.89e-24     * 0.090
## 2         Reward_Category   1 119 10.626 1.00e-03     * 0.002
## 3 Phase:Reward_Category   2 238  9.611 9.68e-05     * 0.003
##
## $`Mauchly's Test for Sphericity`
##                    Effect     W     p p<.05
## 1                   Phase 0.996 0.798
## 2 Phase:Reward_Category 0.964 0.117
##
## $`Sphericity Corrections`
##                    Effect   GGe       DF[GG]    p[GG] p[GG]<.05   HFe
## 1                   Phase 0.996 1.99, 237.09 2.29e-24         * 1.013
## 2 Phase:Reward_Category 0.966  1.93, 229.8 1.21e-04         * 0.981
##         DF[HF]    p[HF] p[HF]<.05
## 1 2.03, 241.12 1.89e-24         *
## 2 1.96, 233.52 1.09e-04         *
```

```r
# Graph: guessing accuracy by phase and category
x1a.MA = plot_by_group_cat(data = data.x1a, yvar = "MA", ylim = c(0,
    1.2), ylab = "Guessing Accuracy", subtitle = "Experiment 1a",
    lab.vjust = 2.5)
ggsave(file = "x1a.MA.svg", plot = x1a.MA, width = 10, height = 10,
    units = "cm")
x1a.MA
```

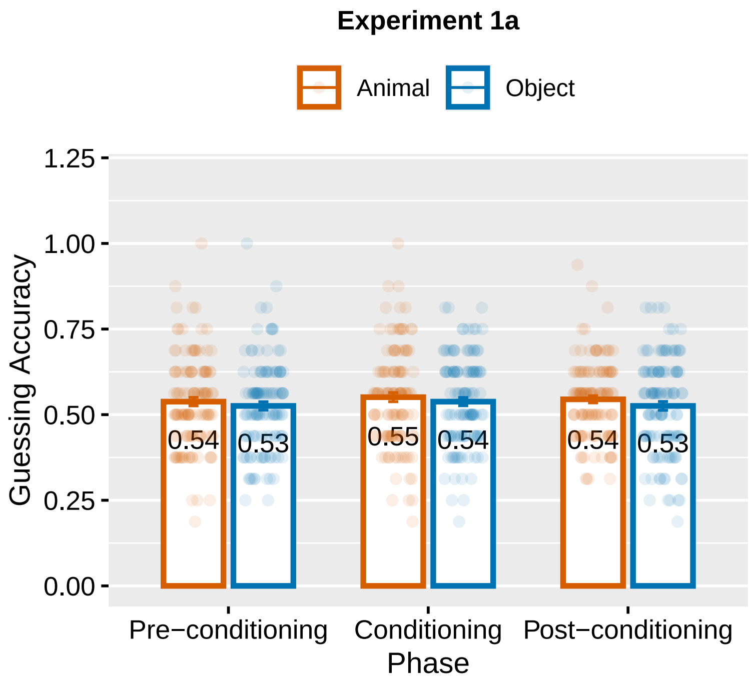


```r
# Graph: guessing accuracy by phase and reward category
x1a.MA = plot_by_group(data = data.x1a, yvar = "MA", ylim = c(0,
    1.2), ylab = "Guessing Accuracy", subtitle = "Experiment 1a",
    lab.vjust = 2.5)
ggsave(file = "x1a.MA.svg", plot = x1a.MA, width = 10, height = 10,
    units = "cm")
x1a.MA
```

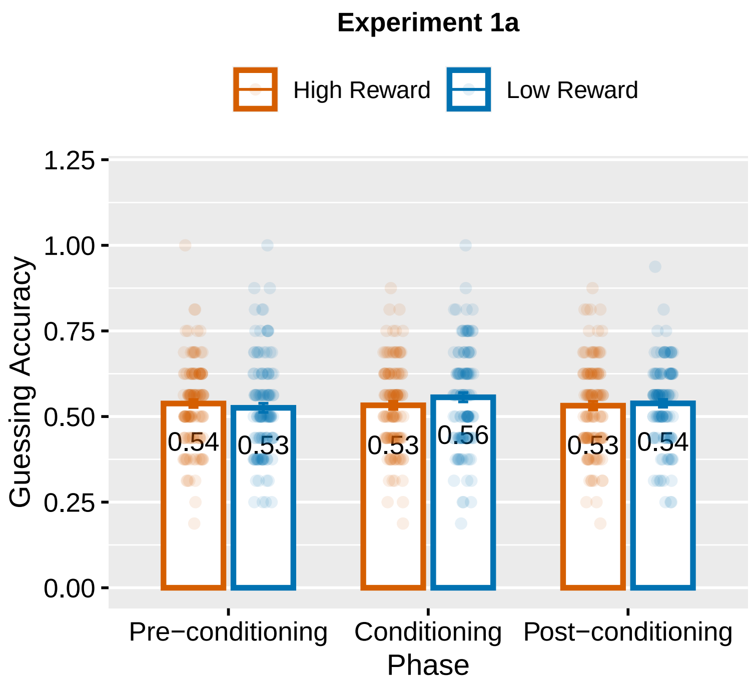


```
# Graph: guessing reaction time by phase and reward
# category
x1a.RT = plot_by_group(data = data.x1a, yvar = "RT", ylim = c(0,
    4000), ylab = "Guess Reaction Time (ms)", subtitle = "Experiment 1a",
    lab.sf = 0, lab.vjust = 2.5)
ggsave(file = "x1a.RT.svg", plot = x1a.RT, width = 10, height = 10,
    units = "cm")
x1a.RT
```

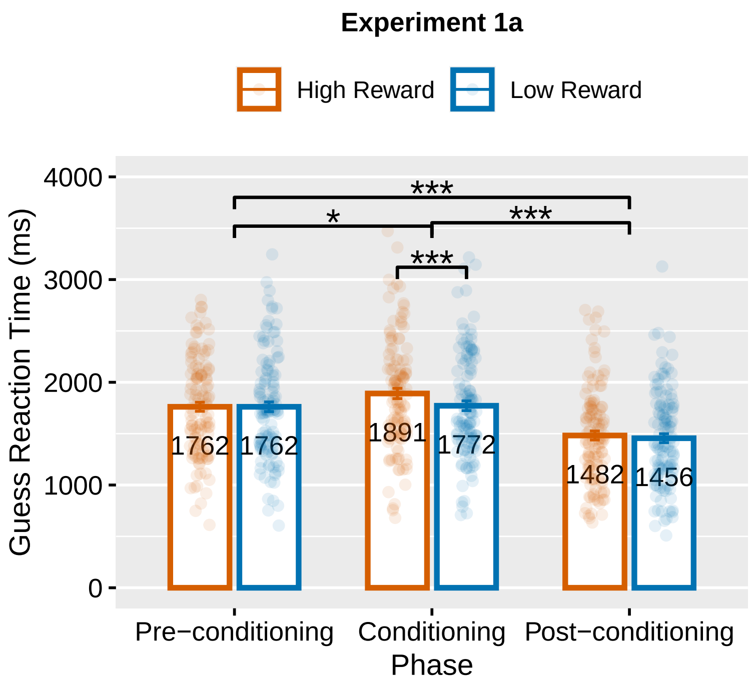


### Experiment 1b

Next we checked whether matching accuracy in each phase varied by stimuli category (animal vs. objects) and the reward category it was associated with (high vs. low reward)

```
# Matching accuracy by categories (animal vs. objects)
aggregate(MA ~ Category + Phase, data.x1b, FUN = function(MA) c(mean = mean(MA),
    se = std.error(MA)))
```

```
##   Category            Phase    MA.mean      MA.se
## 1   Animal  Pre-conditioning 0.53958333 0.01104380
## 2   Object  Pre-conditioning 0.51510417 0.01274694
## 3   Animal      Conditioning 0.53333333 0.01333301
## 4   Object      Conditioning 0.53854167 0.01186185
## 5   Animal Post-conditioning 0.52500000 0.01417753
## 6   Object Post-conditioning 0.52656250 0.01108654
```

```
# Phase 1 (pre-conditioning)
t.test(data = x1b_ph1, MA ~ Category, paired = TRUE)
```

```
##
##  Paired t-test
##
## data:  MA by Category
## t = 1.4358, df = 119, p-value = 0.1537
## alternative hypothesis: true difference in means is not equal to 0
## 95 percent confidence interval:
```

```
##  -0.009279572  0.058237905
## sample estimates:
## mean of the differences
##              0.02447917
```

```r
cohens_dav(data = x1b_ph1, x = MA, group = Category)
```

```
## # A tibble: 2 x 4
##   Category count  mean    sd
##   <chr>    <int> <dbl> <dbl>
## 1 Animal     120 0.540 0.121
## 2 Object     120 0.515 0.140
## [1] "Effect size Cohen's d(av):"
## [1] 0.1878573
```

```r
# Phase 2 (conditioning)
t.test(data = x1b_ph2, MA ~ Category, paired = TRUE)
```

```
##
##  Paired t-test
##
## data:  MA by Category
## t = -0.3023, df = 119, p-value = 0.763
## alternative hypothesis: true difference in means is not equal to 0
## 95 percent confidence interval:
##  -0.03932379  0.02890713
## sample estimates:
## mean of the differences
##            -0.005208333
```

```r
cohens_dav(data = x1b_ph2, x = MA, group = Category)
```

```
## # A tibble: 2 x 4
##   Category count  mean    sd
##   <chr>    <int> <dbl> <dbl>
## 1 Animal     120 0.533 0.146
## 2 Object     120 0.539 0.130
## [1] "Effect size Cohen's d(av):"
## [1] -0.03774212
```

```r
# Phase 3 (post-conditioning)
t.test(data = x1b_ph3, MA ~ Category, paired = TRUE)
```

```
##
##  Paired t-test
##
## data:  MA by Category
## t = -0.090284, df = 119, p-value = 0.9282
## alternative hypothesis: true difference in means is not equal to 0
## 95 percent confidence interval:
##  -0.03583101  0.03270601
```

```
## sample estimates:
## mean of the differences
##              -0.0015625
```

```
cohens_dav(data = x1b_ph3, x = MA, group = Category)
```

```
## # A tibble: 2 x 4
##   Category count  mean    sd
##   <chr>    <int> <dbl> <dbl>
## 1 Animal     120 0.525 0.155
## 2 Object     120 0.527 0.121
## [1] "Effect size Cohen's d(av):"
## [1] -0.01129161
```

```
# Repeated measures ANOVA on matching accuracy by phase and
# category
anova_test(MA ~ Phase * Category + Error(UserID/(Phase * Category)),
    data = data.x1b)
```

```
## ANOVA Table (type III tests)
##
## $ANOVA
##           Effect DFn DFd     F     p p<.05      ges
## 1          Phase   2 238 0.483 0.617       0.001000
## 2       Category   1 119 0.302 0.584       0.000474
## 3 Phase:Category   2 238 0.970 0.381       0.002000
##
## $`Mauchly's Test for Sphericity`
##           Effect     W     p p<.05
## 1          Phase 0.993 0.670
## 2 Phase:Category 1.000 0.993
##
## $`Sphericity Corrections`
##           Effect   GGe      DF[GG] p[GG] p[GG]<.05   HFe       DF[HF] p[HF]
## 1          Phase 0.993 1.99, 236.4 0.616           1.010  2.02, 240.4 0.617
## 2 Phase:Category 1.000   2, 237.97 0.381           1.017 2.03, 242.04 0.381
##   p[HF]<.05
## 1
## 2
```

Furthermore, we check how matching accuracy varies by reward category (high vs. low).

```
# Matching accuracy by reward category (high vs. low) Phase
# 2 (conditioning)
t.test(data = x1b_ph2, MA ~ Reward_Category, paired = TRUE)
```

```
##
##  Paired t-test
##
## data:  MA by Reward_Category
## t = -1.5256, df = 119, p-value = 0.1298
## alternative hypothesis: true difference in means is not equal to 0
```

```
## 95 percent confidence interval:
##  -0.059841285  0.007757951
## sample estimates:
## mean of the differences
##             -0.02604167
```

```r
cohens_dav(data = x1b_ph2, x = MA, group = Reward_Category)
```

```
## # A tibble: 2 x 4
##   Reward_Category count  mean    sd
##   <fct>           <int> <dbl> <dbl>
## 1 High Reward       120 0.523 0.124
## 2 Low Reward        120 0.549 0.150
## [1] "Effect size Cohen's d(av):"
## [1] -0.1900023
```

```r
# Repeated measures ANOVA on matching accuracy by phase and
# reward category
anova_test(MA ~ Phase * Reward_Category + Error(UserID/(Phase *
    Reward_Category)), data = data.x1b)
```

```
## ANOVA Table (type III tests)
##
## $ANOVA
##                  Effect DFn DFd     F     p p<.05   ges
## 1                 Phase   2 238 0.483 0.617       0.001
## 2       Reward_Category   1 119 1.216 0.272       0.002
## 3 Phase:Reward_Category   2 238 4.117 0.017     * 0.010
##
## $`Mauchly's Test for Sphericity`
##                  Effect     W     p p<.05
## 1                 Phase 0.993 0.670
## 2 Phase:Reward_Category 0.999 0.948
##
## $`Sphericity Corrections`
##                  Effect   GGe      DF[GG] p[GG] p[GG]<.05   HFe       DF[HF]
## 1                 Phase 0.993 1.99, 236.4 0.616           1.010  2.02, 240.4
## 2 Phase:Reward_Category 0.999   2, 237.79 0.018         * 1.016 2.03, 241.85
##   p[HF] p[HF]<.05
## 1 0.617
## 2 0.017         *
```

```r
# Repeated measures ANOVA on reaction time by phase and
# reward category
anova_test(RT ~ Phase * Reward_Category + Error(UserID/(Phase *
    Reward_Category)), data = data.x1b)
```

```
## ANOVA Table (type III tests)
##
## $ANOVA
##                  Effect DFn DFd      F        p p<.05      ges
## 1                 Phase   2 238 85.362 1.13e-28     * 0.106000
```

```
## 2       Reward_Category   1 119 10.999 1.00e-03     * 0.002000
## 3 Phase:Reward_Category   2 238  2.593 7.70e-02       0.000614
##
## $`Mauchly's Test for Sphericity`
##                  Effect     W     p p<.05
## 1                 Phase 0.967 0.141
## 2 Phase:Reward_Category 0.978 0.267
##
## $`Sphericity Corrections`
##                  Effect   GGe        DF[GG]    p[GG] p[GG]<.05   HFe        DF[HF]
## 1                 Phase 0.968 1.94, 230.47 7.5e-28         * 0.984 1.97, 234.22
## 2 Phase:Reward_Category 0.978 1.96, 232.85 7.8e-02           0.995  1.99, 236.7
##       p[HF] p[HF]<.05
## 1 2.92e-28         *
## 2 7.70e-02
```

```
# Graph: matching accuracy by phase and category
x1b.MA = plot_by_group_cat(data = data.x1b, yvar = "MA", ylim = c(0,
    1.2), ylab = "Matching Accuracy", subtitle = "Experiment 1b",
    lab.vjust = 2.5)
ggsave(file = "x1a.MA.svg", plot = x1b.MA, width = 10, height = 10,
    units = "cm")
x1b.MA
```

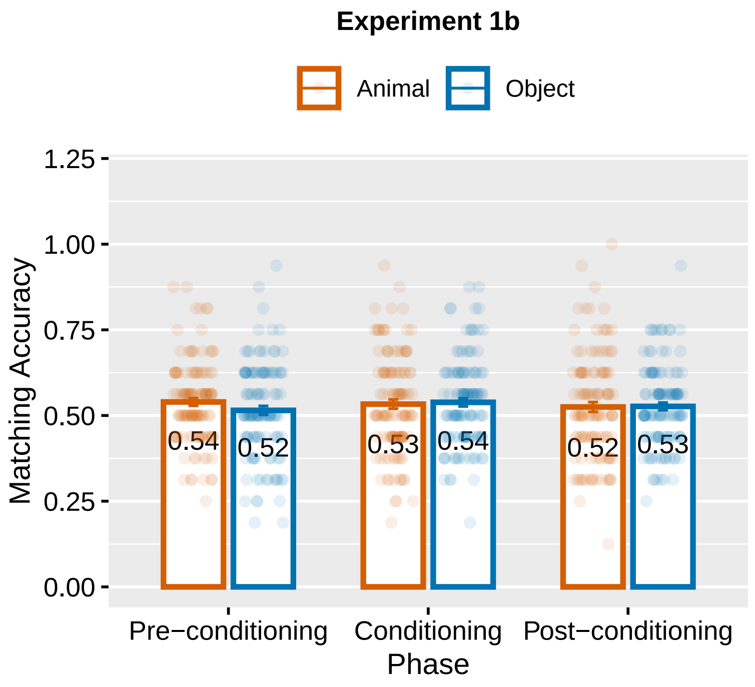


```
# Graph: matching accuracy by phase and reward category
x1b.MA = plot_by_group(data = data.x1b, "1b", yvar = "MA", ylim = c(0,
    1.2), ylab = "Matching Accuracy", subtitle = "Experiment 1b",
    lab.vjust = 2.5)
x1b.MA
```

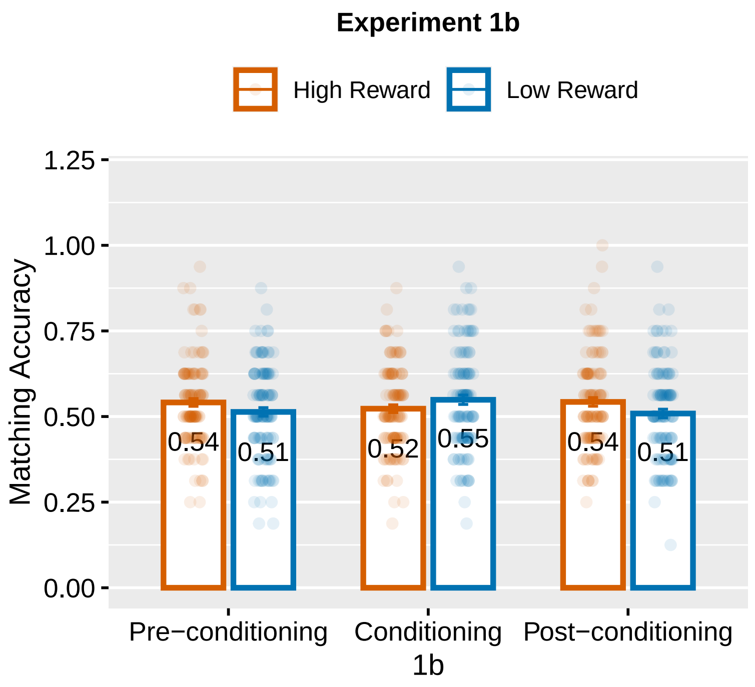


```
ggsave(file = "x1b.MA.svg", plot = x1b.MA, width = 10, height = 10,
    units = "cm")
```

```
# Graph: matching reaction time by phase and reward
# category
x1b.RT = plot_by_group(data = data.x1b, yvar = "RT", ylim = c(0,
    4000), ylab = "Reaction Time (ms)", subtitle = "Experiment 1b",
    lab.sf = 0, lab.vjust = 2.5)
ggsave(file = "x1b.RT.svg", plot = x1b.RT, width = 10, height = 10,
    units = "cm")
x1b.RT
```

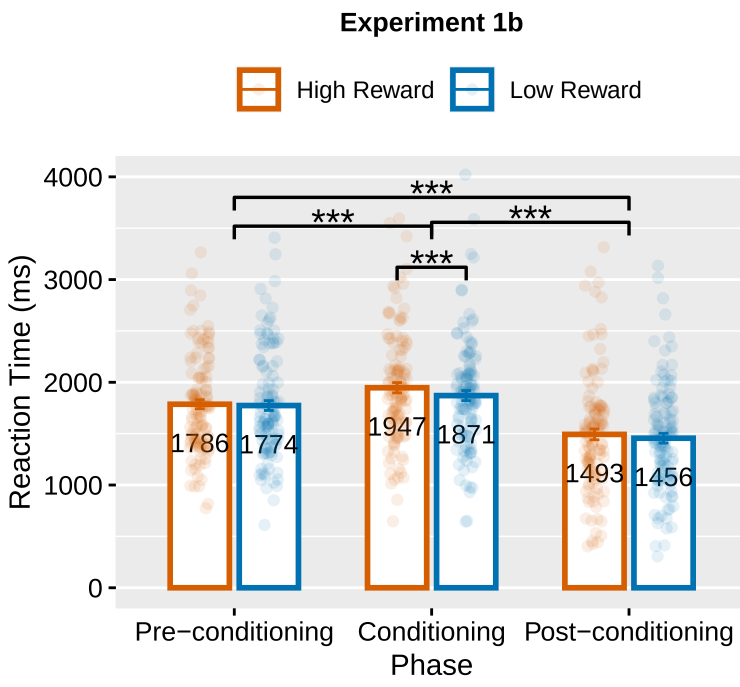


## 4.2 Comparison of Response Biases

### Experiment 1a (All Memory)

```
# Effect of reward category on response bias in phase 1
# (pre-conditioning)
t.test(data = x1a_ph1, RB ~ Reward_Category, paired = TRUE)
```

```
##
##  Paired t-test
##
## data:  RB by Reward_Category
## t = -0.46308, df = 119, p-value = 0.6442
## alternative hypothesis: true difference in means is not equal to 0
## 95 percent confidence interval:
##  -0.11498813  0.07139868
## sample estimates:
## mean of the differences
##             -0.02179473
```

```
cohens_dav(data = x1a_ph1, x = RB, group = Reward_Category)
```

```
## # A tibble: 2 x 4
##   Reward_Category count  mean    sd
##   <fct>           <int> <dbl> <dbl>
## 1 High Reward       120 0.413 0.492
```

```
## 2 Low Reward        120 0.435 0.466
## [1] "Effect size Cohen's d(av):"
## [1] -0.04550652
```

```r
# Effect of reward category on response bias in phase 2
# (conditioning)
t.test(data = x1a_ph2, RB ~ Reward_Category, paired = TRUE)
```

```
##
##  Paired t-test
##
## data:  RB by Reward_Category
## t = -0.11691, df = 119, p-value = 0.9071
## alternative hypothesis: true difference in means is not equal to 0
## 95 percent confidence interval:
##  -0.09479985  0.08422932
## sample estimates:
## mean of the differences
##            -0.005285269
```

```r
cohens_dav(data = x1a_ph2, x = RB, group = Reward_Category)
```

```
## # A tibble: 2 x 4
##   Reward_Category count  mean    sd
##   <fct>           <int> <dbl> <dbl>
## 1 High Reward       120 0.449 0.486
## 2 Low Reward        120 0.454 0.461
## [1] "Effect size Cohen's d(av):"
## [1] -0.01116316
```

```r
# Effect of reward category on response bias in phase 3
# (post-conditioning)
t.test(data = x1a_ph3, RB ~ Reward_Category, paired = TRUE)
```

```
##
##  Paired t-test
##
## data:  RB by Reward_Category
## t = -0.54812, df = 119, p-value = 0.5846
## alternative hypothesis: true difference in means is not equal to 0
## 95 percent confidence interval:
##  -0.12005009  0.06799625
## sample estimates:
## mean of the differences
##             -0.02602692
```

```r
cohens_dav(data = x1a_ph3, x = RB, group = Reward_Category)
```

```
## # A tibble: 2 x 4
##   Reward_Category count  mean    sd
##   <fct>           <int> <dbl> <dbl>
```

```
## 1 High Reward       120 0.445 0.451
## 2 Low Reward        120 0.471 0.519
## [1] "Effect size Cohen's d(av):"
## [1] -0.05368299
```

### Experiment 1a (High Certainty Memory)

```r
# Effect of reward category on response bias in phase 1
# (pre-conditioning)
t.test(data = x1a_high_ph1, RB ~ Reward_Category, paired = TRUE)
```

```
##
##  Paired t-test
##
## data:  RB by Reward_Category
## t = -0.34997, df = 119, p-value = 0.727
## alternative hypothesis: true difference in means is not equal to 0
## 95 percent confidence interval:
##  -0.12237166  0.08561195
## sample estimates:
## mean of the differences
##             -0.01837986
```

```r
cohens_dav(data = x1a_high_ph1, x = RB, group = Reward_Category)
```

```
## # A tibble: 2 x 4
##   Reward_Category count  mean    sd
##   <fct>           <int> <dbl> <dbl>
## 1 High Reward       120 0.459 0.587
## 2 Low Reward        120 0.477 0.577
## [1] "Effect size Cohen's d(av):"
## [1] -0.03158073
```

```r
# Effect of reward category on response bias in phase 2
# (conditioning)
t.test(data = x1a_high_ph2, RB ~ Reward_Category, paired = TRUE)
```

```
##
##  Paired t-test
##
## data:  RB by Reward_Category
## t = -0.54425, df = 119, p-value = 0.5873
## alternative hypothesis: true difference in means is not equal to 0
## 95 percent confidence interval:
##  -0.12231206  0.06957103
## sample estimates:
## mean of the differences
##             -0.02637052
```

```r
cohens_dav(data = x1a_high_ph2, x = RB, group = Reward_Category)
```

```
## # A tibble: 2 x 4
##   Reward_Category count  mean    sd
##   <fct>           <int> <dbl> <dbl>
## 1 High Reward       120 0.466 0.612
## 2 Low Reward        120 0.492 0.560
## [1] "Effect size Cohen's d(av):"
## [1] -0.04499846
```

```r
# Effect of reward category on response bias in phase 3
# (post-conditioning)
t.test(data = x1a_high_ph3, RB ~ Reward_Category, paired = TRUE)
```

```
##
##  Paired t-test
##
## data:  RB by Reward_Category
## t = -0.52296, df = 119, p-value = 0.602
## alternative hypothesis: true difference in means is not equal to 0
## 95 percent confidence interval:
##  -0.13815586  0.08042644
## sample estimates:
## mean of the differences
##             -0.02886471
```

```r
cohens_dav(data = x1a_high_ph3, x = RB, group = Reward_Category)
```

```
## # A tibble: 2 x 4
##   Reward_Category count  mean    sd
##   <fct>           <int> <dbl> <dbl>
## 1 High Reward       120 0.506 0.567
## 2 Low Reward        120 0.534 0.611
## [1] "Effect size Cohen's d(av):"
## [1] -0.04903073
```

### Experiment 1b (All Memory)

```r
# Effect of reward category on response bias in phase 1
# (pre-conditioning)
t.test(data = x1b_ph1, RB ~ Reward_Category, paired = TRUE)
```

```
##
##  Paired t-test
##
## data:  RB by Reward_Category
## t = -0.18706, df = 119, p-value = 0.8519
## alternative hypothesis: true difference in means is not equal to 0
## 95 percent confidence interval:
```

```
##  -0.1248922  0.1033320
## sample estimates:
## mean of the differences
##              -0.0107801
```

```r
cohens_dav(data = x1b_ph1, x = RB, group = Reward_Category)
```

```
## # A tibble: 2 x 4
##   Reward_Category count  mean    sd
##   <fct>           <int> <dbl> <dbl>
## 1 High Reward       120 0.441 0.550
## 2 Low Reward        120 0.452 0.490
## [1] "Effect size Cohen's d(av):"
## [1] -0.02073527
```

```r
# Effect of reward category on response bias in phase 2
# (conditioning)
t.test(data = x1b_ph2, RB ~ Reward_Category, paired = TRUE)
```

```
##
##  Paired t-test
##
## data:  RB by Reward_Category
## t = -1.4062, df = 119, p-value = 0.1623
## alternative hypothesis: true difference in means is not equal to 0
## 95 percent confidence interval:
##  -0.19816338  0.03358683
## sample estimates:
## mean of the differences
##             -0.08228828
```

```r
cohens_dav(data = x1b_ph2, x = RB, group = Reward_Category)
```

```
## # A tibble: 2 x 4
##   Reward_Category count  mean    sd
##   <fct>           <int> <dbl> <dbl>
## 1 High Reward       120 0.471 0.537
## 2 Low Reward        120 0.553 0.511
## [1] "Effect size Cohen's d(av):"
## [1] -0.157092
```

```r
# Effect of reward category on response bias in phase 3
# (post-conditioning)
t.test(data = x1b_ph3, RB ~ Reward_Category, paired = TRUE)
```

```
##
##  Paired t-test
##
## data:  RB by Reward_Category
## t = -0.27179, df = 119, p-value = 0.7863
## alternative hypothesis: true difference in means is not equal to 0
```

```
## 95 percent confidence interval:
##  -0.12385948  0.09396131
## sample estimates:
## mean of the differences
##             -0.01494909
```

```r
cohens_dav(data = x1b_ph3, x = RB, group = Reward_Category)
```

```
## # A tibble: 2 x 4
##   Reward_Category count  mean    sd
##   <fct>           <int> <dbl> <dbl>
## 1 High Reward       120 0.498 0.542
## 2 Low Reward        120 0.513 0.559
## [1] "Effect size Cohen's d(av):"
## [1] -0.02714721
```

### Experiment 1b (High Certainty Memory)

Trend in response bias for items in the conditioning phase, t(119) = -1.76, p = .08, $d_{av}$ = -.19, showing a lower response bias for items in the high reward category. Lower response biases pertain to more liberal response behaviour whereby participants are more likely to respond 'old' to items. Our t-tests below reveal that participants were more likely to respond 'old' to items from the high reward category.

```r
# Effect of reward category on response bias in phase 1
# (pre-conditioning)
t.test(data = x1b_high_ph1, RB ~ Reward_Category, paired = TRUE)
```

```
##
##  Paired t-test
##
## data:  RB by Reward_Category
## t = -0.73583, df = 119, p-value = 0.4633
## alternative hypothesis: true difference in means is not equal to 0
## 95 percent confidence interval:
##  -0.18751729  0.08590824
## sample estimates:
## mean of the differences
##             -0.05080453
```

```r
cohens_dav(data = x1b_high_ph1, x = RB, group = Reward_Category)
```

```
## # A tibble: 2 x 4
##   Reward_Category count  mean    sd
##   <fct>           <int> <dbl> <dbl>
## 1 High Reward       120 0.487 0.685
## 2 Low Reward        120 0.538 0.623
## [1] "Effect size Cohen's d(av):"
## [1] -0.07771462
```

```r
# Effect of reward category on response bias in phase 2
# (conditioning)
t.test(data = x1b_high_ph2, RB ~ Reward_Category, paired = TRUE)
```

```
##
##  Paired t-test
##
## data:  RB by Reward_Category
## t = -1.7632, df = 119, p-value = 0.08044
## alternative hypothesis: true difference in means is not equal to 0
## 95 percent confidence interval:
##  -0.28253451  0.01637371
## sample estimates:
## mean of the differences
##              -0.1330804
```

```r
cohens_dav(data = x1b_high_ph2, x = RB, group = Reward_Category)
```

```
## # A tibble: 2 x 4
##   Reward_Category count  mean    sd
##   <fct>           <int> <dbl> <dbl>
## 1 High Reward       120 0.493 0.701
## 2 Low Reward        120 0.626 0.684
## [1] "Effect size Cohen's d(av):"
## [1] -0.1921697
```

```r
# Effect of reward category on response bias in phase 3
# (post-conditioning)
t.test(data = x1b_high_ph3, RB ~ Reward_Category, paired = TRUE)
```

```
##
##  Paired t-test
##
## data:  RB by Reward_Category
## t = -0.48943, df = 119, p-value = 0.6254
## alternative hypothesis: true difference in means is not equal to 0
## 95 percent confidence interval:
##  -0.1698325  0.1025149
## sample estimates:
## mean of the differences
##              -0.0336588
```

```r
cohens_dav(data = x1b_high_ph3, x = RB, group = Reward_Category)
```

```
## # A tibble: 2 x 4
##   Reward_Category count  mean    sd
##   <fct>           <int> <dbl> <dbl>
## 1 High Reward       120 0.556 0.677
## 2 Low Reward        120 0.590 0.721
## [1] "Effect size Cohen's d(av):"
## [1] -0.04814412
```

# Experiment 1 - Linear Model

```
# Load necessary packages
library(dplyr)
library(tidyverse)
library(rstatix)
library(svglite)
library(rlang)
library(plotrix)
library(gridExtra)
library(tinytex)
library(formatR)
library(knitr)
library(lme4)
```

**This section contains linear mixed model analyses and results for Experiment 1.**

## Data loading

```
# Load Experiment 1a and 1b data
data.x1a <- read.csv("Exp1a_CleanData/Main/x1a_Regression.csv")  # all trial data
data.x1b <- read.csv("Exp1b_CleanData/Main/x1b_Regression.csv")  # all trial data

# Filter to create dataset with only high certainty memory
# trials
data.high.x1a <- subset(data.x1a, Certainty == 0 | Certainty ==
    1 | Certainty == 4 | Certainty == 5)
data.high.x1b <- subset(data.x1b, Certainty == 0 | Certainty ==
    1 | Certainty == 4 | Certainty == 5)

# Insert Say_Old column based on memory responses Trials
# where participants were too slow are omitted (taken as
# NA)
data.x1a <- data.x1a %>%
    mutate(Say_Old = NA, Say_Old = ifelse(Certainty == 0, 1,
        Say_Old), Say_Old = ifelse(Certainty == 1, 1, Say_Old),
        Say_Old = ifelse(Certainty == 2, 1, Say_Old), Say_Old = ifelse(Certainty ==
            3, 0, Say_Old), Say_Old = ifelse(Certainty == 4,
            0, Say_Old), Say_Old = ifelse(Certainty == 5, 0,
            Say_Old))

data.x1b <- data.x1b %>%
    mutate(Say_Old = NA, Say_Old = ifelse(Certainty == 0, 1,
        Say_Old), Say_Old = ifelse(Certainty == 1, 1, Say_Old),
        Say_Old = ifelse(Certainty == 2, 1, Say_Old), Say_Old = ifelse(Certainty ==
```

```
            3, 0, Say_Old), Say_Old = ifelse(Certainty == 4,
            0, Say_Old), Say_Old = ifelse(Certainty == 5, 0,
            Say_Old))

data.high.x1a <- data.high.x1a %>%
    mutate(Say_Old = NA, Say_Old = ifelse(Certainty == 0, 1,
        Say_Old), Say_Old = ifelse(Certainty == 1, 1, Say_Old),
        Say_Old = ifelse(Certainty == 4, 0, Say_Old), Say_Old = ifelse(Certainty ==
            5, 0, Say_Old))

data.high.x1b <- data.high.x1b %>%
    mutate(Say_Old = NA, Say_Old = ifelse(Certainty == 0, 1,
        Say_Old), Say_Old = ifelse(Certainty == 1, 1, Say_Old),
        Say_Old = ifelse(Certainty == 4, 0, Say_Old), Say_Old = ifelse(Certainty ==
            5, 0, Say_Old))

data.x1a <- data.x1a[!is.na(data.x1a$Say_Old), ]
data.x1b <- data.x1b[!is.na(data.x1b$Say_Old), ]
data.high.x1a <- data.high.x1a[!is.na(data.high.x1a$Say_Old),
    ]
data.high.x1b <- data.high.x1b[!is.na(data.high.x1b$Say_Old),
    ]
```

# Prepare data for regression

```
# Prepare coded and factored data for regression analysis
data.x1a <- data.x1a %>%
    mutate(Phase = replace(Phase, Phase == "Rec_Memory", 0),
        Reward_Category = replace(Reward_Category, Reward_Category ==
            -1, 0))

data.x1b <- data.x1b %>%
    mutate(Phase = replace(Phase, Phase == "Rec_Memory", 0),
        Reward_Category = replace(Reward_Category, Reward_Category ==
            -1, 0))

data.high.x1a <- data.high.x1a %>%
    mutate(Phase = replace(Phase, Phase == "Rec_Memory", 0),
        Reward_Category = replace(Reward_Category, Reward_Category ==
            -1, 0))

data.high.x1b <- data.high.x1b %>%
    mutate(Phase = replace(Phase, Phase == "Rec_Memory", 0),
        Reward_Category = replace(Reward_Category, Reward_Category ==
            -1, 0))
```

# Data format

**Datasets:** Trial by trial summary of performance on the matching and memory tasks for all participants.

**Data variables:**

1. UserID: unique user identification
2. Reward_Category: stimuli reward category ("1":High Reward, "0":Low Reward)
3. Phase: phase in which stimuli was encoded ("0":New Items, 1":Pre-conditioning,"2":Conditioning,"3":Post-conditioning)
4. Stim: word describing the stimuli image

Further unused variables: 5. Category: stimuli category ("Animal", "Object") 6. Rew_Subgroup: allocation of stimuli category to high reward ("Reward_Animals", "Reward_Objects") 7. Memory_RT: memory trial reaction time in ms 7. Memory_Correct: memory trial ("1" correct, "0" wrong) 8. Match_RT: matching trial reaction time in ms 10. Match_Correct: matching trial ("1":correct, "0":wrong) 11. Sex 12. Age 13. Stim_Type: ("old_img", "new_img") 14. Certainty: memory trial certainty response ("0":definitely old, "12":likely old, "24":maybe old, "48":maybe new, "60":likley new, "72":definitely new)

# 1. Main Analysis (LM Model)

As another complementary analysis of the effects of reward category on recognition memory performance across phases, we estimated generalized linear mixed-effect models (GLMMs) with a logit-link function using the lme4 R package (Bates et al., 2015). The dependent variable (Say_Old) was participants' categorical response to the memory test collapsed across response certainty with responding old (Say_Old = 1) or responding new (Say_Old = 0). We included main effects of reward category, with high reward category (Reward_Category = 1) and low reward category (Reward_Category = 0) and encoding phase for which we used dummy coding. New items (Phase = 0) were taken as the reference category for the other three phases (Phase = 1, 2, 3). In terms of random effects, we first ran models with random intercepts for each participant (UserID) and stimuli item (Stim). Note that adding random slopes for each predictor did not result in model convergence, thus we omit this from our models and only retain random intercepts.

Confidence intervals were calculated using the confint function with bootstrapping method. Instead of relying on Wald's method obtained from the summary() function, we have used bootMer function calculate bootstrapped parametric p-values. For each fixed effect, we calculated the proportion of estimates $> 0$ (when beta is negative) or $< 0$ (when beta is positive) and output a p-value based on this.

```
# Set number of iterations for bootstrapping
Nsim = 100
```

## 1.1 Experiment 1a Immediate Memory (All Memory)

```
glm1.1 <- glmer(Say_Old ~ 1 + Phase * Reward_Category + (1 |
    UserID) + (1 | Stim), family = binomial(link = "logit"),
    data = data.x1a, glmerControl(optimizer = "bobyqa"))
summary(glm1.1)
```

```
## Generalized linear mixed model fit by maximum likelihood (Laplace
##   Approximation) [glmerMod]
##  Family: binomial  ( logit )
## Formula: Say_Old ~ 1 + Phase * Reward_Category + (1 | UserID) + (1 | Stim)
##    Data: data.x1a
## Control: glmerControl(optimizer = "bobyqa")
##
```

```
##      AIC      BIC   logLik deviance df.resid
##  23699.3  23779.8 -11839.7  23679.3    22984
##
## Scaled residuals:
##     Min      1Q  Median      3Q     Max
## -3.3320 -0.5119 -0.3280  0.6660  5.3649
##
## Random effects:
##  Groups Name        Variance Std.Dev.
##  UserID (Intercept) 0.3421   0.5849
##  Stim   (Intercept) 0.1416   0.3763
## Number of obs: 22994, groups:  UserID, 120; Stim, 97
##
## Fixed effects:
##                         Estimate Std. Error z value Pr(>|z|)
## (Intercept)             -1.85254    0.37273  -4.970 6.69e-07 ***
## Phase1                   2.35800    0.37406   6.304 2.90e-10 ***
## Phase2                   2.31592    0.37403   6.192 5.95e-10 ***
## Phase3                   2.21717    0.37399   5.928 3.06e-09 ***
## Reward_Category         -0.10790    0.05415  -1.993  0.04629 *
## Phase1:Reward_Category  0.20442    0.08869   2.305  0.02117 *
## Phase2:Reward_Category  0.13347    0.08825   1.513  0.13040
## Phase3:Reward_Category  0.26232    0.08814   2.976  0.00292 **
## ---
## Signif. codes:  0 '***' 0.001 '**' 0.01 '*' 0.05 '.' 0.1 ' ' 1
##
## Correlation of Fixed Effects:
##             (Intr) Phase1 Phase2 Phase3 Rwrd_C P1:R_C P2:R_C
## Phase1      -0.976
## Phase2      -0.976  0.983
## Phase3      -0.976  0.983  0.983
## Rewrd_Ctgry -0.067  0.067  0.067  0.067
## Phs1:Rwrd_C  0.040 -0.114 -0.040 -0.040 -0.610
## Phs2:Rwrd_C  0.040 -0.040 -0.114 -0.040 -0.613  0.373
## Phs3:Rwrd_C  0.040 -0.040 -0.040 -0.113 -0.614  0.372  0.375
```

```r
confint.1.1 <- confint.merMod(glm1.1, method = "boot", nsim = Nsim,
    parallel = "multicore", ncpus = 4)
pvals.1.1 <- bootMer(glm1.1, FUN = fixef, nsim = Nsim, parallel = "multicore",
    ncpus = 4)  #
saveRDS(confint.1.1, "confint.1.1.rds")
saveRDS(pvals.1.1, "pvals.1.1.rds")
```

```r
# load previously run results
confint.1.1 <- readRDS(file = "confint.1.1.rds")
confint.1.1
```

```
##                              2.5 %      97.5 %
## .sig01                  0.49657618  0.68119126
## .sig02                  0.31256512  0.45065443
## (Intercept)            -2.52512987 -1.13266440
## Phase1                  1.73624487  3.06796300
## Phase2                  1.64942118  2.98842620
```

```
## Phase3                   1.55711504  2.89857350
## Reward_Category        -0.23381416  0.02306021
## Phase1:Reward_Category  0.05277066  0.37228800
## Phase2:Reward_Category -0.04654316  0.33448022
## Phase3:Reward_Category  0.12611117  0.47016469
```

```
pvals.1.1 <- readRDS(file = "pvals.1.1.rds")
pvals.1.1
```

```
##
## PARAMETRIC BOOTSTRAP
##
##
## Call:
## bootMer(x = glm1.1, FUN = fixef, nsim = Nsim, parallel = "multicore",
##     ncpus = 4)
##
##
## Bootstrap Statistics :
##       original       bias    std. error
## t1* -1.8525439 -0.032235811  0.39548742
## t2*  2.3580035  0.018023890  0.39717973
## t3*  2.3159169  0.020670187  0.39738365
## t4*  2.2171671  0.017467913  0.40240067
## t5* -0.1079036 -0.002425424  0.05949964
## t6*  0.2044238  0.001490218  0.08939784
## t7*  0.1334734  0.009106062  0.08846372
## t8*  0.2623151  0.012079044  0.09813236
```

```
# proportion of estimates > 0 (when beta is negative) or <
# 0 (when beta is positive)
pvals.1.1.list <- mean(pvals.1.1$t[, 1] > 0) * 2
pvals.1.1.list[2] <- mean(pvals.1.1$t[, 2] < 0) * 2
pvals.1.1.list[3] <- mean(pvals.1.1$t[, 3] < 0) * 2
pvals.1.1.list[4] <- mean(pvals.1.1$t[, 4] < 0) * 2
pvals.1.1.list[5] <- mean(pvals.1.1$t[, 5] > 0) * 2
pvals.1.1.list[6] <- mean(pvals.1.1$t[, 6] < 0) * 2
pvals.1.1.list[7] <- mean(pvals.1.1$t[, 7] < 0) * 2
pvals.1.1.list[8] <- mean(pvals.1.1$t[, 8] < 0) * 2

# label output
pvals.1.1.out <- as.list(pvals.1.1.list)
names(pvals.1.1.out) <- row.names(as.data.frame(summary(glm1.1)$coefficients))
pvals.1.1.out
```

```
## $`(Intercept)`
## [1] 0
##
## $Phase1
```

```
## [1] 0
##
## $Phase2
## [1] 0
##
## $Phase3
## [1] 0
##
## $Reward_Category
## [1] 0.08
##
## $`Phase1:Reward_Category`
## [1] 0.06
##
## $`Phase2:Reward_Category`
## [1] 0.08
##
## $`Phase3:Reward_Category`
## [1] 0
```

Firstly, the GLMMM analysis on Say_Old responses can be used to analyse participants overall performance on the memory task. The 'Intercept' term which is negative, β = -1.852, 95% CI [-2.252, -1.133], p < .001, represents the log odds of answering 'old' to a new item. Whereas, the 'Phase' predictor estimates are positive, showing that participants have successfully remembered previously seen items.
In terms of main effects, there were some interaction effects be tween reward category and pre-conditioning phase, β = 0.204, 95% CI [1.736, 3.068], p = .06, and in the conditioning phase, β = 0.133, 95% CI [1.649, 2.988], p = .08, and in the post-conditoning phase, β = 0.262, 95% CI [1.557, 2.899], p < 0.001. The positive beta value translates to participants being more likely to correctly respond 'old' to previously seen items from the high reward category in both phases. It is surprising that this effect is the strongest for items in the post-conditioning phase.

## 1.2 Experiment 1a (High Certainty Memory)

```
glm1.2 <- glmer(Say_Old ~ 1 + Phase * Reward_Category + (1 |
    UserID) + (1 | Stim), family = binomial(link = "logit"),
    data = data.high.x1a, glmerControl(optimizer = "bobyqa"))
summary(glm1.2)
```

```
## Generalized linear mixed model fit by maximum likelihood (Laplace
##   Approximation) [glmerMod]
##  Family: binomial  ( logit )
## Formula: Say_Old ~ 1 + Phase * Reward_Category + (1 | UserID) + (1 | Stim)
##    Data: data.high.x1a
```

```
## Control: glmerControl(optimizer = "bobyqa")
##
##      AIC      BIC   logLik deviance df.resid
##  16675.4  16753.7  -8327.7  16655.4    18604
##
## Scaled residuals:
##     Min      1Q  Median      3Q     Max
## -5.0888 -0.4065 -0.2444  0.5586  8.1987
##
## Random effects:
##  Groups Name        Variance Std.Dev.
##  UserID (Intercept) 0.6416   0.8010
##  Stim   (Intercept) 0.1919   0.4381
## Number of obs: 18614, groups:  UserID, 120; Stim, 97
##
## Fixed effects:
##                        Estimate Std. Error z value Pr(>|z|)
## (Intercept)            -2.37579    0.43724  -5.434 5.52e-08 ***
## Phase1                  3.04969    0.43704   6.978 2.99e-12 ***
## Phase2                  3.02333    0.43712   6.916 4.63e-12 ***
## Phase3                  2.94224    0.43688   6.735 1.64e-11 ***
## Reward_Category        -0.07794    0.07286  -1.070   0.2848
## Phase1:Reward_Category  0.16841    0.10862   1.550   0.1210
## Phase2:Reward_Category  0.15645    0.10902   1.435   0.1513
## Phase3:Reward_Category  0.24746    0.10854   2.280   0.0226 *
## ---
## Signif. codes:  0 '***' 0.001 '**' 0.01 '*' 0.05 '.' 0.1 ' ' 1
##
## Correlation of Fixed Effects:
##             (Intr) Phase1 Phase2 Phase3 Rwrd_C P1:R_C P2:R_C
## Phase1      -0.972
## Phase2      -0.972  0.983
## Phase3      -0.972  0.983  0.983
## Rewrd_Ctgry -0.076  0.076  0.076  0.076
## Phs1:Rwrd_C  0.051 -0.118 -0.050 -0.050 -0.670
## Phs2:Rwrd_C  0.051 -0.051 -0.119 -0.051 -0.667  0.447
## Phs3:Rwrd_C  0.049 -0.049 -0.049 -0.116 -0.670  0.447  0.445
```

```
confint.1.2 <- confint.merMod(glm1.2, method = "boot", nsim = Nsim,
    parallel = "multicore", ncpus = 4)
pvals.1.2 <- bootMer(glm1.2, FUN = fixef, nsim = Nsim, parallel = "multicore",
    ncpus = 4)  #
saveRDS(confint.1.2, "confint.1.2.rds")
saveRDS(pvals.1.2, "pvals.1.2.rds")
```

```
# load previously run results
confint.1.2 <- readRDS(file = "confint.1.2.rds")
confint.1.2
```

```
##                              2.5 %     97.5 %
## .sig01                  0.66638042  0.8998181
## .sig02                  0.35490081  0.5395837
## (Intercept)            -3.28720309 -1.4841005
```

```
## Phase1                    2.01142772  3.9382309
## Phase2                    2.05279804  3.9515476
## Phase3                    1.94829190  3.8279044
## Reward_Category          -0.23345943  0.0695049
## Phase1:Reward_Category -0.05859881  0.4454720
## Phase2:Reward_Category -0.07244481  0.4020371
## Phase3:Reward_Category  0.05257982  0.4786499
```

```
pvals.1.2 <- readRDS(file = "pvals.1.2.rds")
pvals.1.2
```

```
##
## PARAMETRIC BOOTSTRAP
##
##
## Call:
## bootMer(x = glm1.2, FUN = fixef, nsim = Nsim, parallel = "multicore",
##     ncpus = 4)
##
##
## Bootstrap Statistics :
##       original        bias    std. error
## t1* -2.3757897  0.042407950  0.49037992
## t2*  3.0496894 -0.030887834  0.48971780
## t3*  3.0233254 -0.042931871  0.48482835
## t4*  2.9422437 -0.042567856  0.48573812
## t5* -0.0779356 -0.002776918  0.08012742
## t6*  0.1684096 -0.001572688  0.10403184
## t7*  0.1564537  0.017575610  0.11651632
## t8*  0.2474573  0.013433458  0.10910138
```

```
pvals.1.2.list <- mean(pvals.1.2$t[, 1] > 0) * 2
pvals.1.2.list[2] <- mean(pvals.1.2$t[, 2] < 0) * 2
pvals.1.2.list[3] <- mean(pvals.1.2$t[, 3] < 0) * 2
pvals.1.2.list[4] <- mean(pvals.1.2$t[, 4] < 0) * 2
pvals.1.2.list[5] <- mean(pvals.1.2$t[, 5] > 0) * 2
pvals.1.2.list[6] <- mean(pvals.1.2$t[, 6] < 0) * 2
pvals.1.2.list[7] <- mean(pvals.1.2$t[, 7] < 0) * 2
pvals.1.2.list[8] <- mean(pvals.1.2$t[, 8] < 0) * 2

# label output
pvals.1.2.out <- as.list(pvals.1.2.list)
names(pvals.1.2.out) <- row.names(as.data.frame(summary(glm1.2)$coefficients))
pvals.1.2.out
```

```
## $`(Intercept)`
## [1] 0
##
## $Phase1
## [1] 0
##
## $Phase2
## [1] 0
```

```
##
## $Phase3
## [1] 0
##
## $Reward_Category
## [1] 0.22
##
## $`Phase1:Reward_Category`
## [1] 0.08
##
## $`Phase2:Reward_Category`
## [1] 0.16
##
## $`Phase3:Reward_Category`
## [1] 0
```

When considering only high certainty trials from the memory test, the interaction between reward category and encoding phases were diminished compared to when considering all memory trials. More specifically, there was no interaction effect between reward category and conditioning phase β = 0.156, 95% CI [-0.072, 0.402], p = .16. Again, surprisingly, there were some interaction effects between reward category and the pre-conditioning phase, $\beta$ = 0.168, 95% CI [-0.059, 0.445], p = .08, and stronger effects in the conditioning phase, $\beta$ = 0.247, 95% CI [0.053, 0.479], $p < 0.001$.

## 1.3 Experiment 1b 24-hour Delayed Memory (All Memory)

In experiment 1b, participants underwent memory test 24 hour after the encoding phases.

```
glm1.3 <- glmer(Say_Old ~ 1 + Phase + Reward_Category + Phase:Reward_Category +
    (1 | UserID) + (1 | Stim), family = binomial(link = "logit"),
    data = data.x1b, glmerControl(optimizer = "bobyqa"))
summary(glm1.3)
```

```
## Generalized linear mixed model fit by maximum likelihood (Laplace
##   Approximation) [glmerMod]
##  Family: binomial  ( logit )
## Formula: Say_Old ~ 1 + Phase + Reward_Category + Phase:Reward_Category +
##     (1 | UserID) + (1 | Stim)
##    Data: data.x1b
## Control: glmerControl(optimizer = "bobyqa")
##
##      AIC      BIC   logLik deviance df.resid
##  25721.7  25802.1 -12850.9  25701.7    22966
##
## Scaled residuals:
##     Min      1Q  Median      3Q     Max
## -3.3098 -0.6267 -0.4266  0.7922  5.0609
##
## Random effects:
##  Groups Name        Variance Std.Dev.
```

```
##  UserID (Intercept) 0.3834   0.6192
##  Stim   (Intercept) 0.2240   0.4733
## Number of obs: 22976, groups:  UserID, 120; Stim, 97
##
## Fixed effects:
##                          Estimate Std. Error z value Pr(>|z|)
## (Intercept)              -1.55346    0.46538  -3.338 0.000844 ***
## Phase1                    1.69751    0.46682   3.636 0.000277 ***
## Phase2                    1.38209    0.46677   2.961 0.003067 **
## Phase3                    1.51932    0.46687   3.254 0.001137 **
## Reward_Category           0.04481    0.04825   0.929 0.353017
## Phase1:Reward_Category   -0.03653    0.08436  -0.433 0.664952
## Phase2:Reward_Category    0.17314    0.08423   2.056 0.039825 *
## Phase3:Reward_Category   -0.03530    0.08414  -0.419 0.674862
## ---
## Signif. codes:  0 '***' 0.001 '**' 0.01 '*' 0.05 '.' 0.1 ' ' 1
##
## Correlation of Fixed Effects:
##             (Intr) Phase1 Phase2 Phase3 Rwrd_C P1:R_C P2:R_C
## Phase1      -0.982
## Phase2      -0.982  0.989
## Phase3      -0.982  0.989  0.989
## Rewrd_Ctgry -0.051  0.050  0.050  0.050
## Phs1:Rwrd_C  0.028 -0.088 -0.028 -0.028 -0.572
## Phs2:Rwrd_C  0.027 -0.027 -0.088 -0.027 -0.573  0.326
## Phs3:Rwrd_C  0.030 -0.029 -0.029 -0.090 -0.573  0.327  0.327
```

```
confint.1.3 <- confint.merMod(glm1.3, method = "boot", nsim = Nsim,
    parallel = "multicore", ncpus = 4)
pvals.1.3 <- bootMer(glm1.3, FUN = fixef, nsim = Nsim, parallel = "multicore",
    ncpus = 4)  #
saveRDS(confint.1.3, "confint.1.3.rds")
saveRDS(pvals.1.3, "pvals.1.3.rds")
```

```
# load previously run results
confint.1.3 <- readRDS(file = "confint.1.3.rds")
confint.1.3
```

```
##                              2.5 %     97.5 %
## .sig01                  0.53471894  0.7099724
## .sig02                  0.38328625  0.5476027
## (Intercept)            -2.48548057 -0.4543760
## Phase1                  0.56196103  2.6640054
## Phase2                  0.24688355  2.2768203
## Phase3                  0.34165711  2.3971623
## Reward_Category        -0.06210865  0.1513584
## Phase1:Reward_Category -0.24628628  0.1336110
## Phase2:Reward_Category -0.02574389  0.3277373
## Phase3:Reward_Category -0.20912519  0.1513219
```

```
pvals.1.3 <- readRDS(file = "pvals.1.3.rds")
pvals.1.3
```

```
##
## PARAMETRIC BOOTSTRAP
##
##
## Call:
## bootMer(x = glm1.3, FUN = fixef, nsim = Nsim, parallel = "multicore",
##     ncpus = 4)
##
##
## Bootstrap Statistics :
##        original        bias    std. error
## t1* -1.55346272 -0.0246194275  0.47895676
## t2*  1.69751222  0.0035653473  0.48188390
## t3*  1.38209140  0.0131082658  0.48229988
## t4*  1.51931766  0.0147825571  0.48564843
## t5*  0.04481005  0.0005554625  0.04754149
## t6* -0.03653306  0.0101408851  0.07396320
## t7*  0.17313549 -0.0050333269  0.08990428
## t8* -0.03529513  0.0002992793  0.08493342
```

```
pvals.1.3.list <- mean(pvals.1.3$t[, 1] > 0) * 2
pvals.1.3.list[2] <- mean(pvals.1.3$t[, 2] < 0) * 2
pvals.1.3.list[3] <- mean(pvals.1.3$t[, 3] < 0) * 2
pvals.1.3.list[4] <- mean(pvals.1.3$t[, 4] < 0) * 2
pvals.1.3.list[5] <- mean(pvals.1.3$t[, 5] < 0) * 2
pvals.1.3.list[6] <- mean(pvals.1.3$t[, 6] < 0) * 2
pvals.1.3.list[7] <- mean(pvals.1.3$t[, 7] < 0) * 2
pvals.1.3.list[8] <- mean(pvals.1.3$t[, 8] > 0) * 2

# label output
pvals.1.3.out <- as.list(pvals.1.3.list)
names(pvals.1.3.out) <- row.names(as.data.frame(summary(glm1.3)$coefficients))
pvals.1.3.out
```

```
## $`(Intercept)`
## [1] 0
##
## $Phase1
## [1] 0
##
## $Phase2
## [1] 0
##
## $Phase3
## [1] 0
##
## $Reward_Category
## [1] 0.34
##
## $`Phase1:Reward_Category`
```

```
## [1] 1.28
##
## $`Phase2:Reward_Category`
## [1] 0.08
##
## $`Phase3:Reward_Category`
## [1] 0.66
```

The model did not show an effect of reward category on response bias, $\beta$ = 0.045, 95%CI [-0.062, 0.151], p = .34. In other words, participants were not biased in responding 'old' to items from a specific category.

In terms of main effects, there was some evidence for an interaction between reward category and the conditioning phase, $\beta$ = 0.173, 95% CI [-0.026, 0.328], p = .08. There were no effects between reward category and the pre-conditioning phase, $\beta$ = -0.037, 95% CI [-0.246, 0.134], p = 1.28, and the post-conditioning phase, $\beta$ = -0.035, 95% CI [-0.209, 0.151], p = 0.66. The beta values confidence intervals extend from negative to positive values, showing that there is no significant effect in either direction. In other words, participants were equally likely to correctly responded 'old' to previously seen items from high and low reward categories. Again this agrees with what was found in the main analysis using classical t-tests as well as Bayes Factors

## 1.4 Experiment 1b (High Certainty Memory)

```
glm1.4 <- glmer(Say_Old ~ 1 + Phase + Reward_Category + Phase:Reward_Category +
    (1 | UserID) + (1 | Stim), family = binomial(link = "logit"),
    data = data.high.x1b, glmerControl(optimizer = "bobyqa"))
summary(glm1.4)
```

```
## Generalized linear mixed model fit by maximum likelihood (Laplace
##   Approximation) [glmerMod]
##  Family: binomial  ( logit )
## Formula: Say_Old ~ 1 + Phase + Reward_Category + Phase:Reward_Category +
##     (1 | UserID) + (1 | Stim)
##    Data: data.high.x1b
## Control: glmerControl(optimizer = "bobyqa")
##
##      AIC      BIC   logLik deviance df.resid
##  17602.3  17679.9  -8791.1  17582.3    17330
##
## Scaled residuals:
##     Min      1Q  Median      3Q     Max
## -3.7413 -0.5451 -0.3468  0.6539  6.3919
##
## Random effects:
##  Groups Name        Variance Std.Dev.
##  UserID (Intercept) 0.6169   0.7854
##  Stim   (Intercept) 0.3382   0.5815
## Number of obs: 17340, groups:  UserID, 120; Stim, 97
##
## Fixed effects:
##                         Estimate Std. Error z value Pr(>|z|)
## (Intercept)             -1.96111    0.57803  -3.393 0.000692 ***
## Phase1                   2.17669    0.57931   3.757 0.000172 ***
```

```
## Phase2                  1.81419    0.57929   3.132 0.001738 **
## Phase3                  1.99831    0.57938   3.449 0.000563 ***
## Reward_Category         0.17261    0.06262   2.756 0.005844 **
## Phase1:Reward_Category -0.11401    0.10341  -1.103 0.270231
## Phase2:Reward_Category  0.16968    0.10379   1.635 0.102089
## Phase3:Reward_Category -0.12109    0.10318  -1.174 0.240533
## ---
## Signif. codes:  0 '***' 0.001 '**' 0.01 '*' 0.05 '.' 0.1 ' ' 1
##
## Correlation of Fixed Effects:
##             (Intr) Phase1 Phase2 Phase3 Rwrd_C P1:R_C P2:R_C
## Phase1      -0.982
## Phase2      -0.982  0.990
## Phase3      -0.982  0.990  0.990
## Rewrd_Ctgry -0.056  0.056  0.056  0.056
## Phs1:Rwrd_C  0.033 -0.089 -0.033 -0.033 -0.605
## Phs2:Rwrd_C  0.033 -0.033 -0.089 -0.033 -0.603  0.364
## Phs3:Rwrd_C  0.034 -0.034 -0.034 -0.090 -0.606  0.366  0.366
```

```
confint.1.4 <- confint.merMod(glm1.4, method = "boot", nsim = Nsim,
    parallel = "multicore", ncpus = 4)
pvals.1.4 <- bootMer(glm1.4, FUN = fixef, nsim = Nsim, parallel = "multicore",
    ncpus = 4)  #
saveRDS(confint.1.4, "confint.1.4.rds")
saveRDS(pvals.1.4, "pvals.1.4.rds")
```

```
# load previously run results
confint.1.4 <- readRDS(file = "confint.1.4.rds")
confint.1.4
```

```
##                                2.5 %      97.5 %
## .sig01                  0.7016341505  0.90000874
## .sig02                  0.4647714278  0.70459353
## (Intercept)            -2.8617447920 -0.51874578
## Phase1                  0.7854244807  3.16606263
## Phase2                  0.4007252292  2.72960246
## Phase3                  0.6435991936  3.02855779
## Reward_Category         0.0547414906  0.30767925
## Phase1:Reward_Category -0.2747880731  0.07682914
## Phase2:Reward_Category -0.0006465694  0.42469201
## Phase3:Reward_Category -0.3154277619  0.07334256
```

```
pvals.1.4 <- readRDS(file = "pvals.1.4.rds")
pvals.1.4
```

```
##
## PARAMETRIC BOOTSTRAP
##
##
## Call:
## bootMer(x = glm1.4, FUN = fixef, nsim = Nsim, parallel = "multicore",
##     ncpus = 4)
```

```
##
##
## Bootstrap Statistics :
##        original       bias    std. error
## t1* -1.9611084 -0.094141185  0.52771944
## t2*  2.1766905  0.115691123  0.52219844
## t3*  1.8141860  0.114144305  0.53013090
## t4*  1.9983095  0.104657166  0.52596100
## t5*  0.1726073  0.006051592  0.06822537
## t6* -0.1140106 -0.006200521  0.10538534
## t7*  0.1696810 -0.007834959  0.10094565
## t8* -0.1210931 -0.007524560  0.09966152
```

```
pvals.1.4.list <- mean(pvals.1.4$t[, 1] > 0) * 2
pvals.1.4.list[2] <- mean(pvals.1.4$t[, 2] < 0) * 2
pvals.1.4.list[3] <- mean(pvals.1.4$t[, 3] < 0) * 2
pvals.1.4.list[4] <- mean(pvals.1.4$t[, 4] < 0) * 2
pvals.1.4.list[5] <- mean(pvals.1.4$t[, 5] < 0) * 2
pvals.1.4.list[6] <- mean(pvals.1.4$t[, 6] > 0) * 2
pvals.1.4.list[7] <- mean(pvals.1.4$t[, 7] < 0) * 2
pvals.1.4.list[8] <- mean(pvals.1.4$t[, 8] > 0) * 2

# label output
pvals.1.4.out <- as.list(pvals.1.4.list)
names(pvals.1.4.out) <- row.names(as.data.frame(summary(glm1.4)$coefficients))
pvals.1.4.out
```

```
## $`(Intercept)`
## [1] 0
##
## $Phase1
## [1] 0
##
## $Phase2
## [1] 0
##
## $Phase3
## [1] 0
##
## $Reward_Category
## [1] 0
##
## $`Phase1:Reward_Category`
## [1] 0.18
##
## $`Phase2:Reward_Category`
## [1] 0.12
##
## $`Phase3:Reward_Category`
## [1] 0.16
```

The model of the high certainty memory trials showed no evidence for an interaction between reward category and the conditioning phase, $\beta = 0.170$, 95% CI [-0.001, 0.425], p = .12. Similar to the analysis of all memory trials. there were no effects between reward category and the pre- or post-conditioning phases. In other words, participants were equally likely to correctly responded 'old' to previously seen items from high and low reward categories. Again this agrees with what was found in the main analysis using classical t-tests as well as Bayes Factors.

# 2. Supplementary Analysis

## 2.1 Does Guessing Correctly Predict Memory?

We estimated GLMMs on categorical responses from the memory test with phase and guess outcome as a predictor. Experiment 1a showed no effects of guess outcome on memory whatsoever. However, in Experiment 1b, there was a significant effect of guess outcome on recognition memory in the post-conditioning phase. In other words, correct guesses during encoding resulted in better memory for that item. However, since we also found that guessing accuracy did not differ between reward categories, this effect is expected to equally influence all items.

```
# Prepare coded and factored data for regression analysis
data.x1a <- data.x1a %>%
    mutate(Phase = replace(Phase, Phase == "0", "New")) %>%
    mutate(Phase = replace(Phase, Phase == "1", "Ph1")) %>%
    mutate(Phase = replace(Phase, Phase == "2", "Ph2")) %>%
    mutate(Phase = replace(Phase, Phase == "3", "Ph3"))
data.x1a$Phase = as.factor(data.x1a$Phase)
data.x1b <- data.x1b %>%
    mutate(Phase = replace(Phase, Phase == "0", "New")) %>%
    mutate(Phase = replace(Phase, Phase == "1", "Ph1")) %>%
    mutate(Phase = replace(Phase, Phase == "2", "Ph2")) %>%
    mutate(Phase = replace(Phase, Phase == "3", "Ph3"))
data.x1b$Phase = as.factor(data.x1b$Phase)
```

### Experiment 1a

```
glmGuess <- glmer(Say_Old ~ 1 + Match_Correct * Phase + (1 |
    UserID) + (1 | Stim), family = binomial(link = "logit"),
    data = data.x1a, glmerControl(optimizer = "bobyqa"))
summary(glmGuess)
```

```
## Generalized linear mixed model fit by maximum likelihood (Laplace
##   Approximation) [glmerMod]
##  Family: binomial  ( logit )
## Formula: Say_Old ~ 1 + Match_Correct * Phase + (1 | UserID) + (1 | Stim)
##    Data: data.x1a
## Control: glmerControl(optimizer = "bobyqa")
##
##      AIC      BIC   logLik deviance df.resid
```

```
##   14087.7  14146.5  -7035.8  14071.7    11492
##
## Scaled residuals:
##     Min      1Q  Median      3Q     Max
## -3.8725 -0.9236  0.4607  0.7462  2.7280
##
## Random effects:
##  Groups Name        Variance Std.Dev.
##  UserID (Intercept) 0.6796   0.8244
##  Stim   (Intercept) 0.1632   0.4039
## Number of obs: 11500, groups:  UserID, 120; Stim, 96
##
## Fixed effects:
##                          Estimate Std. Error z value Pr(>|z|)
## (Intercept)               0.61544    0.10099   6.094  1.1e-09 ***
## Match_Correct            -0.03870    0.07310  -0.529    0.597
## PhasePh2                 -0.02441    0.07483  -0.326    0.744
## PhasePh3                 -0.09157    0.07449  -1.229    0.219
## Match_Correct:PhasePh2 -0.10539    0.10241  -1.029    0.303
## Match_Correct:PhasePh3 -0.04928    0.10217  -0.482    0.630
## ---
## Signif. codes:  0 '***' 0.001 '**' 0.01 '*' 0.05 '.' 0.1 ' ' 1
##
## Correlation of Fixed Effects:
##             (Intr) Mtch_C PhsPh2 PhsPh3 M_C:PP2
## Match_Crrct -0.383
## PhasePh2    -0.366  0.506
## PhasePh3    -0.371  0.512  0.499
## Mtch_Cr:PP2  0.267 -0.702 -0.732 -0.364
## Mtch_Cr:PP3  0.270 -0.708 -0.363 -0.728  0.504
```

```
aggregate(Memory_Correct ~ Match_Correct * Phase, data.x1a, FUN = function(CR) c(mean = mean(CR),
    se = std.error(CR)))
```

```
##   Match_Correct Phase Memory_Correct.mean Memory_Correct.se
## 1             0   Ph1          0.62207358        0.01145076
## 2             1   Ph1          0.62500000        0.01072129
## 3             0   Ph2          0.61216294        0.01167439
## 4             1   Ph2          0.60382775        0.01070112
## 5             0   Ph3          0.60033632        0.01160029
## 6             1   Ph3          0.59492435        0.01084761
```

### Experiment 1b

```
glmGuess <- glmer(Say_Old ~ 1 + Match_Correct * Phase + (1 |
    UserID) + (1 | Stim), family = binomial(link = "logit"),
    data = data.x1b, glmerControl(optimizer = "bobyqa"))
summary(glmGuess)
```

```
## Generalized linear mixed model fit by maximum likelihood (Laplace
##   Approximation) [glmerMod]
```

```
##  Family: binomial  ( logit )
## Formula: Say_Old ~ 1 + Match_Correct * Phase + (1 | UserID) + (1 | Stim)
##    Data: data.x1b
## Control: glmerControl(optimizer = "bobyqa")
##
##      AIC      BIC   logLik deviance df.resid
##  14823.3  14882.1  -7403.7  14807.3    11481
##
## Scaled residuals:
##     Min      1Q  Median      3Q     Max
## -3.2633 -0.8660  0.3691  0.8645  3.0481
##
## Random effects:
##  Groups Name        Variance Std.Dev.
##  UserID (Intercept) 0.4331   0.6581
##  Stim   (Intercept) 0.2329   0.4826
## Number of obs: 11489, groups:  UserID, 120; Stim, 96
##
## Fixed effects:
##                         Estimate Std. Error z value Pr(>|z|)
## (Intercept)              0.14821    0.09289   1.595   0.1106
## Match_Correct            0.01068    0.07050   0.151   0.8796
## PhasePh2                -0.14137    0.07186  -1.967   0.0491 *
## PhasePh3                -0.29053    0.07166  -4.054 5.03e-05 ***
## Match_Correct:PhasePh2 -0.13393    0.09882  -1.355   0.1753
## Match_Correct:PhasePh3  0.21064    0.09889   2.130   0.0332 *
## ---
## Signif. codes:  0 '***' 0.001 '**' 0.01 '*' 0.05 '.' 0.1 ' ' 1
##
## Correlation of Fixed Effects:
##             (Intr) Mtch_C PhsPh2 PhsPh3 M_C:PP2
## Match_Crrct -0.399
## PhasePh2    -0.384  0.506
## PhasePh3    -0.387  0.512  0.499
## Mtch_Cr:PP2  0.280 -0.701 -0.729 -0.363
## Mtch_Cr:PP3  0.281 -0.704 -0.361 -0.726  0.500
```

```
aggregate(Memory_Correct ~ Match_Correct * Phase, data.x1b, FUN = function(CR) c(mean = mean(CR),
    se = std.error(CR)))
```

```
##   Match_Correct Phase Memory_Correct.mean Memory_Correct.se
## 1             0   Ph1          0.52794687        0.01174713
## 2             1   Ph1          0.53857567        0.01108895
## 3             0   Ph2          0.49606299        0.01186077
## 4             1   Ph2          0.47927840        0.01103367
## 5             0   Ph3          0.46424642        0.01169982
## 6             1   Ph3          0.52011923        0.01113792
```

# Experiment 2 - Main Analysis

```
# Load necessary packages
library(dplyr)
library(tidyverse)
library(rstatix)
library(ggplot2)
library(ggpubr)
library(ggprism)
library(svglite)
library(rlang)
library(plotrix)
library(gridExtra)
library(BayesFactor)
library(tinytex)
library(formatR)
library(knitr)
source("funcs.R")
```

This section contains the analysis and results associated with Experiment 2 in the article 'Reward conditioning may not have an effect on category-specific memory'.

Experiment 2a consists of pre-conditioning and conditioning phases, and experiment 2b consists of conditioning and post-conditioning phases. The encoding phases were followed by a 24-hour delayed memory test in both experiments.

## Data loading

```
# Load Experiment 2a data
data.x2a <- read.csv("adaptiveMemoryReplication/Exp2a_CleanData/Main/x2a_Anova.csv")  # all memory data
data.x2a.high <- read.csv("adaptiveMemoryReplication/Exp2a_CleanData/Main/x2a_High_Anova.csv")  # only
# Change phase labels for readability
data.x2a$Phase[data.x2a$Phase == "Ph1"] <- "Pre-conditioning"
data.x2a$Phase[data.x2a$Phase == "Ph2"] <- "Conditioning"
data.x2a.high$Phase[data.x2a.high$Phase == "Ph1"] <- "Pre-conditioning"
data.x2a.high$Phase[data.x2a.high$Phase == "Ph2"] <- "Conditioning"
# Reorder variables for graphs
data.x2a$Reward_Category <- factor(data.x2a$Reward_Category,
    levels = c("High Reward", "Low Reward"))
data.x2a$Phase <- factor(data.x2a$Phase, levels = c("Pre-conditioning",
    "Conditioning"))
data.x2a.high$Reward_Category <- factor(data.x2a.high$Reward_Category,
    levels = c("High Reward", "Low Reward"))
data.x2a.high$Phase <- factor(data.x2a.high$Phase, levels = c("Pre-conditioning",
    "Conditioning"))
```

```r
# Load Experiment 2b data
data.x2b <- read.csv("adaptiveMemoryReplication/Exp2b_CleanData/Main/x2b_Anova.csv")  # all memory data
data.x2b.high <- read.csv("adaptiveMemoryReplication/Exp2b_CleanData/Main/x2b_High_Anova.csv")  # only 
# Change phase labels for readability
data.x2b$Phase[data.x2b$Phase == "Ph1"] <- "Conditioning"
data.x2b$Phase[data.x2b$Phase == "Ph2"] <- "Post-conditioning"
data.x2b.high$Phase[data.x2b.high$Phase == "Ph1"] <- "Conditioning"
data.x2b.high$Phase[data.x2b.high$Phase == "Ph2"] <- "Post-conditioning"
# Reorder variables for graphs
data.x2b$Reward_Category <- factor(data.x2b$Reward_Category,
    levels = c("High Reward", "Low Reward"))
data.x2b$Phase <- factor(data.x2b$Phase, levels = c("Conditioning",
    "Post-conditioning"))
data.x2b.high$Reward_Category <- factor(data.x2b.high$Reward_Category,
    levels = c("High Reward", "Low Reward"))
data.x2b.high$Phase <- factor(data.x2b.high$Phase, levels = c("Conditioning",
    "Post-conditioning"))
```

## Data format

**Datasets:**

By participant summary of performance on the matching and memory tasks. There are two summary datasets for each experiment:

1. data.x2a & data.x2b summarises all memory trials
2. data.x2a.high & data.x2b.high summarises memory trials in which participants responded with higher certainty (confidence rating). This includes trials with 'Definitely Old/New' and 'Likely Old/New' responses, and excludes 'Maybe Old/New' responses.

**Data variables:**

1. UserID: unique user identification
2. Category: stimuli category ("Animal", "Object")
3. Reward_Category: stimuli reward category (High Reward",”Low Reward")
4. Phase: phase in which stimuli was encoded
    - Experiment 2a: ("Pre-conditioning", "Conditioning")
    - Experiment 2b: ("Conditioning", "Post-conditioning")
5. CR: corrected recognition scores from memory task
6. DP: d-prime memory sensitivity in memory task (as per signal detection theory)
7. MA: matching accuracy in matching task
8. RT: reaction time (ms) in matching task
9. RB: response bias in memory task (as per signal detection theory)

Further unused variables: 10. Rew_Subgroup: allocation of situmuli category to high reward ("Reward_Animals", "Reward_Objects") 11. Age 12. Sex 13. HR: hit rate in memory task 14. FA: false alarm rate in memory task

# 1. Main Analysis (Frequentists statistics)

Recognition memory performance was calculated using two measures: corrected recognition (hit rate - false alarm rate) and (d-prime) memory sensitivity as per signal detection theory. Parametric tests were used since the sample size (n = 60) is large enough (n > 30) to assume that data follows normality requirements.

Firstly, a 2x2 factor repeated measures Anova was done to characterise memory by phase and reward category on the memory of items. This analysis was performed on both measures of memory. Following this, more specifically, the effect of reward category (high vs. low reward) on the memory of items from each phase was quantified using two-tailed paired t-tests with alpha = .05.

For each experiment, we then repeated the analysis taking into account only high-certainty memory responses.

## 1.1 Experiment 2a (All Memory)

We conducted a repeated measures two-factor ANOVA on memory performance (both corrected recognition and d-prime) with phase (pre-conditioning, conditioning) and reward category (high reward, low reward) to summarize the main effects.

**Corrected recognition (CR) by phase and reward category**

```
# Summary table and graph
aggregate(CR ~ Reward_Category + Phase, data.x2a, FUN = function(CR) c(mean = mean(CR),
    se = std.error(CR)))
```

```
##   Reward_Category            Phase    CR.mean      CR.se
## 1     High Reward Pre-conditioning 0.44079482 0.02650613
## 2      Low Reward Pre-conditioning 0.50891985 0.02698867
## 3     High Reward     Conditioning 0.49660216 0.02700774
## 4      Low Reward     Conditioning 0.53927699 0.02455970
```

```
x2a.CR = plot_by_group(data = data.x2a, yvar = "CR", ylim = c(0,
    1.2), ylab = "Corrected Recognition", subtitle = "Experiment 2a (All Memory)",
    tag = "1.1 A")
x2a.CR
```

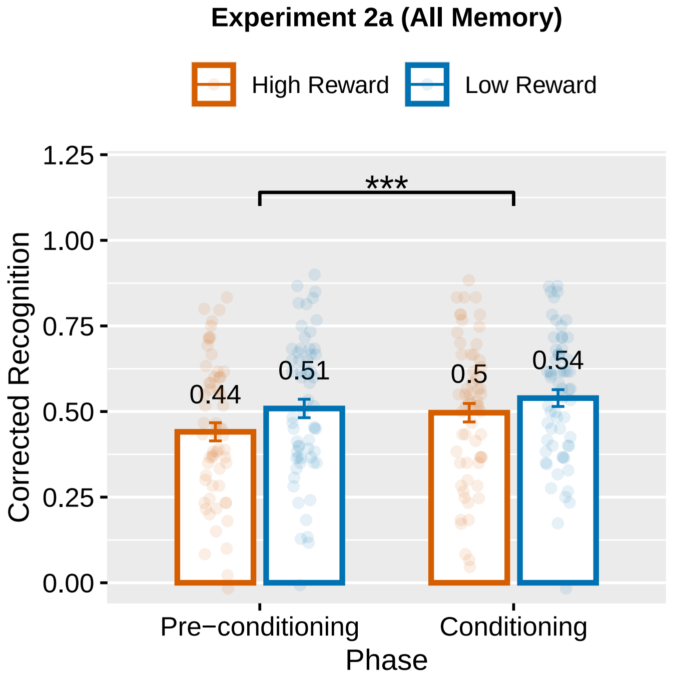


```
# Repeated measures two-factor ANOVA on corrected
# recognition
anova_test(CR ~ Phase * Reward_Category + Error(UserID/(Phase *
    Reward_Category)), data = data.x2a)
```

```
## ANOVA Table (type III tests)
##
##                    Effect DFn DFd      F        p p<.05      ges
## 1                   Phase   1  59 12.757 0.000714     * 0.011000
## 2         Reward_Category   1  59 11.949 0.001000     * 0.018000
## 3 Phase:Reward_Category   1  59  1.429 0.237000       0.000992
```

The repeated measures ANOVA revealed a strong effect of phase, F(1,59) = 12.76, p < .001, $\eta^2$ = .01, and reward category F(1,59) = 11.95, p = .001, $\eta^2$ = .02 on corrected recognition. However there was no significant interaction between encoding phase and the reward category associated with the item F(1,59) = 12.76, p = .24, $\eta^2$ = 0.001. This indicates that memory, as measured by corrected recognition, was not consistently influenced by an item's reward category across phases. We next repeat the same analysis for d-prime scores.

**d-prime (DP) by phase and reward category**

```
# Summary table and graph
aggregate(DP ~ Reward_Category + Phase, data.x2a, FUN = function(DP) c(mean = mean(DP),
    se = std.error(DP)))
```

```
##   Reward_Category            Phase    DP.mean      DP.se
## 1     High Reward Pre-conditioning 1.96915097 0.09698200
## 2      Low Reward Pre-conditioning 2.21611930 0.10704780
```

```
## 3     High Reward     Conditioning 2.19917138 0.10240067
## 4      Low Reward     Conditioning 2.29376978 0.09776017
```

```
x2a.DP = plot_by_group(data = data.x2a, yvar = "DP", ylim = c(0,
    4.6), ylab = "d-prime", subtitle = "Experiment 2a (All Memory)",
    tag = "1.1 B")
ggsave(file = "x2a.DP.svg", plot = x2a.DP, width = 10, height = 10,
    units = "cm")
x2a.DP
```

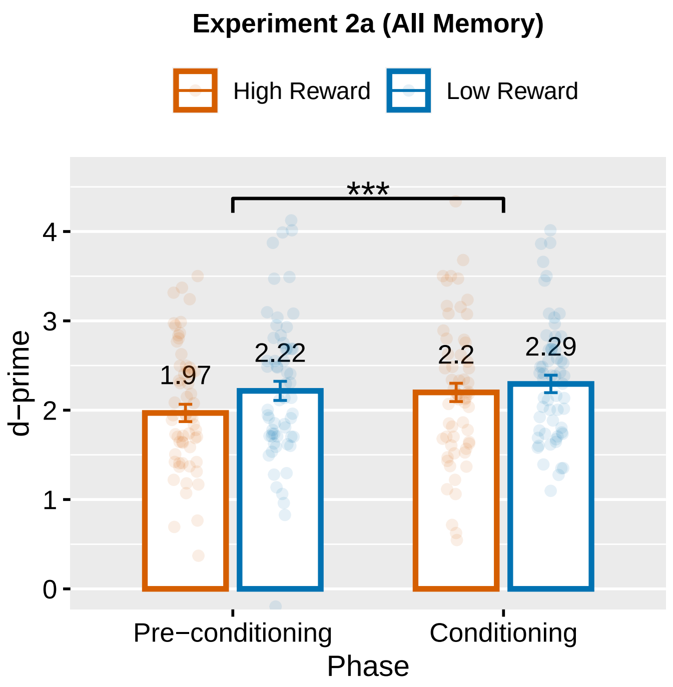


```
# Repeated measures two-factor ANOVA on d-prime scores
anova_test(DP ~ Phase * Reward_Category + Error(UserID/(Phase *
    Reward_Category)), data = data.x2a)
```

```
## ANOVA Table (type III tests)
##
##                  Effect DFn DFd      F     p p<.05   ges
## 1                 Phase   1  59 10.974 0.002     * 0.010
## 2       Reward_Category   1  59  7.990 0.006     * 0.012
## 3 Phase:Reward_Category   1  59  3.136 0.082       0.002
```

In line with corrected recognition scores, the repeated measures ANOVA on d-primes revealed a strong effect of phase, F(1,59) = 10.97, p = .002, $\eta^2$ = .01, and reward category F(1,59) = 7.99, p = .006, $\eta^2$ = .01. Again, there was no significant interaction between encoding phase and the reward category of item F(1,59) = 3.14, p = .082, $\eta^2$ = 0.002, although the effect is stronger compared to corrected recognition analysis.

Following this, we conducted paired t-tests to more specifically characterise the effect of reward category on memory of items from each encoding phase.

```r
# Create subsets for each phase from data.x2a (all memory)
x2a_ph1 <- subset(data.x2a, Phase == "Pre-conditioning")
x2a_ph2 <- subset(data.x2a, Phase == "Conditioning")
```

```r
# Effect of reward category on memory in phase 1
# (pre-conditioning) Corrected recognition (CR)
t.test(data = x2a_ph1, CR ~ Reward_Category, paired = TRUE)
```

```
##
##  Paired t-test
##
## data:  CR by Reward_Category
## t = -3.4233, df = 59, p-value = 0.001131
## alternative hypothesis: true difference in means is not equal to 0
## 95 percent confidence interval:
##  -0.10794557 -0.02830448
## sample estimates:
## mean of the differences
##             -0.06812502
```

```r
cohens_dav(data = x2a_ph1, x = CR, group = Reward_Category)
```

```
## # A tibble: 2 x 4
##   Reward_Category count  mean    sd
##   <fct>           <int> <dbl> <dbl>
## 1 High Reward        60 0.441 0.205
## 2 Low Reward         60 0.509 0.209
## [1] "Effect size Cohen's d(av):"
## [1] -0.3288134
```

```r
# d-prime (DP)
t.test(data = x2a_ph1, DP ~ Reward_Category, paired = TRUE)
```

```
##
##  Paired t-test
##
## data:  DP by Reward_Category
## t = -3.2397, df = 59, p-value = 0.001968
## alternative hypothesis: true difference in means is not equal to 0
## 95 percent confidence interval:
##  -0.39950910 -0.09442758
## sample estimates:
## mean of the differences
##              -0.2469683
```

```r
cohens_dav(data = x2a_ph1, x = DP, group = Reward_Category)
```

```
## # A tibble: 2 x 4
##   Reward_Category count  mean    sd
##   <fct>           <int> <dbl> <dbl>
```

```
## 1 High Reward        60  1.97 0.751
## 2 Low Reward         60  2.22 0.829
## [1] "Effect size Cohen's d(av):"
## [1] -0.3125374
```

In the pre-conditioning phase of experiment 2a, there was a significant effect of reward category both with corrected recognition t(59) = -3.42, p = .001, $d_{av}$ = -.33, and d-prime t(59) = -3.24, p = .002, $d_{av}$ = -.31, although in the opposite direction than expected. Items belonging to the high reward category resulted in lower memory performance than items from the low reward category, which resulted in enhanced corrected recognition. This result was in the opposite direction than demonstrated by the original study (Patil et al., 2017).

```
# Effect of reward category on memory in phase 2
# (conditioning)

# Corrected recognition (CR)
t.test(data = x2a_ph2, CR ~ Reward_Category, paired = TRUE)
```

```
##
##  Paired t-test
##
## data:  CR by Reward_Category
## t = -2.2998, df = 59, p-value = 0.02501
## alternative hypothesis: true difference in means is not equal to 0
## 95 percent confidence interval:
##  -0.079804518 -0.005545148
## sample estimates:
## mean of the differences
##             -0.04267483
```

```
cohens_dav(data = x2a_ph2, x = CR, group = Reward_Category)
```

```
## # A tibble: 2 x 4
##   Reward_Category count  mean    sd
##   <fct>           <int> <dbl> <dbl>
## 1 High Reward        60 0.497 0.209
## 2 Low Reward         60 0.539 0.190
## [1] "Effect size Cohen's d(av):"
## [1] -0.2136735
```

```
# d-prime (DP)
t.test(data = x2a_ph2, DP ~ Reward_Category, paired = TRUE)
```

```
##
##  Paired t-test
##
## data:  DP by Reward_Category
## t = -1.313, df = 59, p-value = 0.1943
## alternative hypothesis: true difference in means is not equal to 0
## 95 percent confidence interval:
##  -0.2387682  0.0495714
## sample estimates:
## mean of the differences
##              -0.0945984
```

```r
cohens_dav(data = x2a_ph2, x = DP, group = Reward_Category)
```

```
## # A tibble: 2 x 4
##   Reward_Category count  mean    sd
##   <fct>           <int> <dbl> <dbl>
## 1 High Reward        60  2.20 0.793
## 2 Low Reward         60  2.29 0.757
## [1] "Effect size Cohen's d(av):"
## [1] -0.1220279
```

We expected a stronger effect of reward category (high vs. low) on the items from the conditioning phase (phase 2). However we found a less significant effect of reward category on corrected recognition t(59) = -2.30, p = .025, $d_{av}$ = -.21, although this was the conditioning phase where participants learn the high/low reward associations with animal/object categories. The effect was even weaker when considering d-prime measures t(59) = -1.31, p = .19, $d_{av}$ = -.12. The trend suggests, as in the pre-conditioning phase, that items in the low reward category resulted in enhanced memory. It is noteworthy that this is a case where analysing corrected recognition scores have resulted in a significant effect whereas considering d-primes did not. Thus deeming it important to consider both measures of recognition memory for comparative analyses and calling for more analysis into the response biases.

## 1.2 Experiment 2a (High Certainty Memory)

Data from experiment 2a was re-analysed considering only high certainty memory responses. **Corrected recognition (CR) by phase and reward category**

```r
# Summary table and graph
aggregate(CR ~ Reward_Category + Phase, data.x2a.high, FUN = function(CR) c(mean = mean(CR),
    se = std.error(CR)))
```

```
##   Reward_Category            Phase    CR.mean      CR.se
## 1     High Reward Pre-conditioning 0.53705925 0.03014934
## 2      Low Reward Pre-conditioning 0.57964365 0.02887545
## 3     High Reward     Conditioning 0.59085261 0.02826604
## 4      Low Reward     Conditioning 0.60546421 0.02836734
```

```r
x2a.high.CR = plot_by_group(data = data.x2a.high, yvar = "CR",
    ylim = c(0, 1.2), ylab = "Corrected Recognition", subtitle = "Experiment 2a (High Certainty)",
    tag = "1.2 A")
x2a.high.CR
```

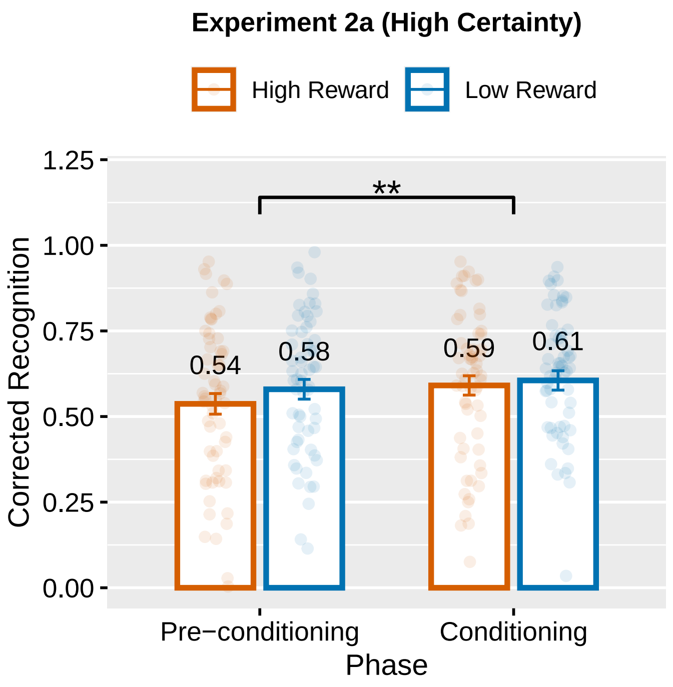


```
# Repeated measures two-factor ANOVA on corrected
# recognition (high certaintly only)
anova_test(CR ~ Phase * Reward_Category + Error(UserID/(Phase *
    Reward_Category)), data = data.x2a.high)
```

```
## ANOVA Table (type III tests)
##
##                    Effect DFn DFd     F     p p<.05     ges
## 1                   Phase   1  59 7.881 0.007     * 0.00800
## 2         Reward_Category   1  59 1.849 0.179       0.00400
## 3 Phase:Reward_Category     1  59 1.209 0.276       0.00099
```

### d-prime (DP) by phase and reward category

```
# Summary table and graph
aggregate(DP ~ Reward_Category + Phase, data.x2a.high, FUN = function(DP) c(mean = mean(DP),
    se = std.error(DP)))
```

```
##   Reward_Category            Phase   DP.mean     DP.se
## 1     High Reward Pre-conditioning 2.4775483 0.1251021
## 2      Low Reward Pre-conditioning 2.6058525 0.1297874
## 3     High Reward     Conditioning 2.7296049 0.1216415
## 4      Low Reward     Conditioning 2.6898960 0.1280482
```

```
x2a.high.DP = plot_by_group(data = data.x2a.high, yvar = "DP",
    ylim = c(0, 4.6), ylab = "d-prime", subtitle = "Experiment 2a (High Certainty)",
    tag = "1.2 B")
x2a.high.DP
```

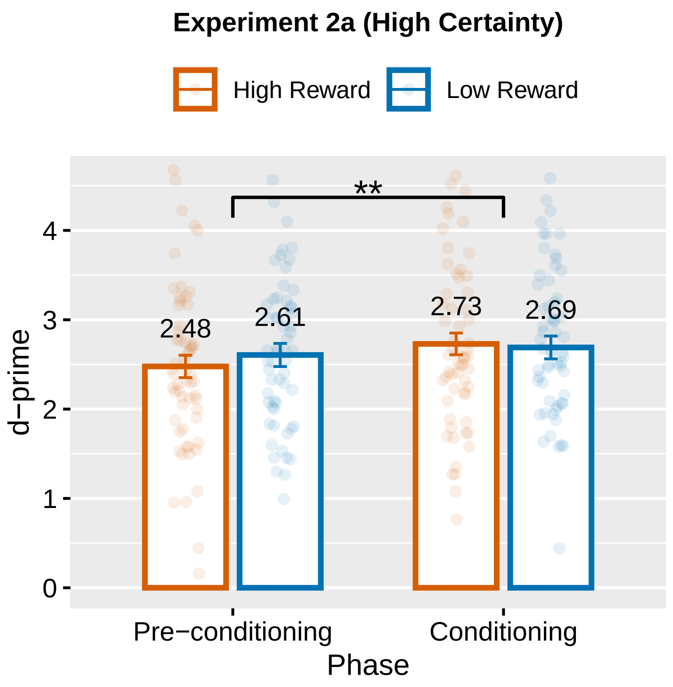


```
# Repeated measures two-factor ANOVA on d-prime scores
# (high certainty only)
anova_test(DP ~ Phase * Reward_Category + Error(UserID/(Phase *
    Reward_Category)), data = data.x2a.high)
```

```
## ANOVA Table (type III tests)
##
##                  Effect DFn DFd     F     p p<.05      ges
## 1                 Phase   1  59 6.747 0.012     * 0.007000
## 2       Reward_Category   1  59 0.339 0.562       0.000522
## 3 Phase:Reward_Category   1  59 2.299 0.135       0.002000
```

```
# Create subsets for each phase from data.x2a (high
# certainty)
x2a_high_ph1 <- subset(data.x2a.high, Phase == "Pre-conditioning")
x2a_high_ph2 <- subset(data.x2a.high, Phase == "Conditioning")
```

```
# Effect of reward category on high certainty memory in
# phase 1 (pre-conditioning)

# Corrected recognition (CR)
t.test(data = x2a_high_ph1, CR ~ Reward_Category, paired = TRUE)
```

```
##
##  Paired t-test
##
## data:  CR by Reward_Category
```

```
## t = -1.621, df = 59, p-value = 0.1104
## alternative hypothesis: true difference in means is not equal to 0
## 95 percent confidence interval:
##  -0.095152786  0.009983987
## sample estimates:
## mean of the differences
##              -0.0425844
```

```
cohens_dav(data = x2a_high_ph1, x = CR, group = Reward_Category)
```

```
## # A tibble: 2 x 4
##   Reward_Category count  mean    sd
##   <fct>           <int> <dbl> <dbl>
## 1 High Reward        60 0.537 0.234
## 2 Low Reward         60 0.580 0.224
## [1] "Effect size Cohen's d(av):"
## [1] -0.1862818
```

```
# d-prime (DP)
t.test(data = x2a_high_ph1, DP ~ Reward_Category, paired = TRUE)
```

```
##
##  Paired t-test
##
## data:  DP by Reward_Category
## t = -1.3151, df = 59, p-value = 0.1936
## alternative hypothesis: true difference in means is not equal to 0
## 95 percent confidence interval:
##  -0.32352149  0.06691309
## sample estimates:
## mean of the differences
##              -0.1283042
```

```
cohens_dav(data = x2a_high_ph1, x = DP, group = Reward_Category)
```

```
## # A tibble: 2 x 4
##   Reward_Category count  mean    sd
##   <fct>           <int> <dbl> <dbl>
## 1 High Reward        60  2.48 0.969
## 2 Low Reward         60  2.61 1.01
## [1] "Effect size Cohen's d(av):"
## [1] -0.12997
```

```
# Effect of reward category on high certainty memory in
# phase 2 (conditioning)

# Corrected recognition (CR)
t.test(data = x2a_high_ph2, CR ~ Reward_Category, paired = TRUE)
```

```
##
##  Paired t-test
```

```
##
## data:  CR by Reward_Category
## t = -0.64193, df = 59, p-value = 0.5234
## alternative hypothesis: true difference in means is not equal to 0
## 95 percent confidence interval:
##  -0.06015857  0.03093537
## sample estimates:
## mean of the differences
##              -0.0146116
```

```
cohens_dav(data = x2a_high_ph2, x = CR, group = Reward_Category)
```

```
## # A tibble: 2 x 4
##   Reward_Category count  mean    sd
##   <fct>           <int> <dbl> <dbl>
## 1 High Reward        60 0.591 0.219
## 2 Low Reward         60 0.605 0.220
## [1] "Effect size Cohen's d(av):"
## [1] -0.06661617
```

```
# d-prime (DP)
t.test(data = x2a_high_ph2, DP ~ Reward_Category, paired = TRUE)
```

```
##
##  Paired t-test
##
## data:  DP by Reward_Category
## t = 0.43894, df = 59, p-value = 0.6623
## alternative hypothesis: true difference in means is not equal to 0
## 95 percent confidence interval:
##  -0.1413114  0.2207293
## sample estimates:
## mean of the differences
##              0.03970896
```

```
cohens_dav(data = x2a_high_ph2, x = DP, group = Reward_Category)
```

```
## # A tibble: 2 x 4
##   Reward_Category count  mean    sd
##   <fct>           <int> <dbl> <dbl>
## 1 High Reward        60  2.73 0.942
## 2 Low Reward         60  2.69 0.992
## [1] "Effect size Cohen's d(av):"
## [1] 0.04106221
```

When repeating the analysis with high certainty memory trials, the effect of reward category in the conditioning phase observed in the main analysis were no longer significant. This was true for both corrected recognition , $t(59) = -0.64$, $p = .52$, $d_{av} = -.07$, and d-prime scores, $t(59) = 0.44$, $p = .66$, $d_{av} = .04$. From the ANOVA, the main effect of phase on memory enhancement remained significant but not as strong, both on corrected recognition $F(1,59) = 7.88$, $p < .007$, $\eta^2 = .008$, and on d-prime scores, $F(1,59) = 6.75$, $p < .012$, $\eta^2 = .002$.

## 1.3 Experiment 2b (All Memory)

The same analysis was repeated for experiment 2b in which participants underwent a conditioning phase where stimuli were paired with high/low reward followed by a post-conditioning phase with no reward conditioning on trials.

**Corrected recognition (CR) by phase and reward category**

```
# Summary table and graph
aggregate(CR ~ Reward_Category + Phase, data.x2b, FUN = function(CR) c(mean = mean(CR),
    se = std.error(CR)))
```

```
##   Reward_Category             Phase   CR.mean      CR.se
## 1     High Reward      Conditioning 0.51476715 0.02333079
## 2      Low Reward      Conditioning 0.50166471 0.02904490
## 3     High Reward Post-conditioning 0.41819764 0.02654339
## 4      Low Reward Post-conditioning 0.41194796 0.02825218
```

```
x2b.CR = plot_by_group(data = data.x2b, yvar = "CR", ylim = c(0,
    1.2), ylab = "Corrected Recognition", subtitle = "Experiment 2b (All Memory)",
    tag = "1.3 A")
x2b.CR
```

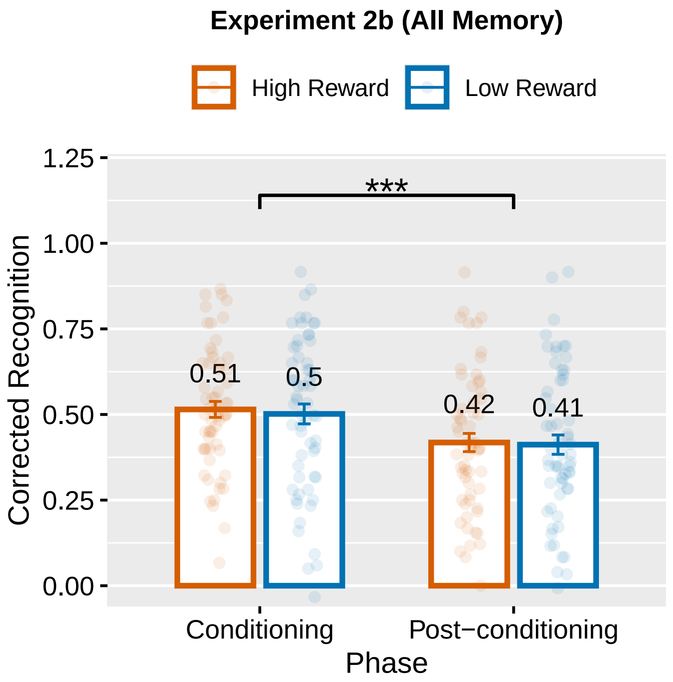


```
# Repeated measures two-factor ANOVA on corrected
# recognition
anova_test(CR ~ Phase * Reward_Category + Error(UserID/(Phase *
    Reward_Category)), data = data.x2b)
```

```
## ANOVA Table (type III tests)
##
##                  Effect DFn DFd      F        p p<.05      ges
## 1                 Phase   1  59 43.634 1.25e-08     * 4.80e-02
## 2       Reward_Category   1  59  0.281 5.98e-01       5.49e-04
## 3 Phase:Reward_Category   1  59  0.115 7.35e-01       6.88e-05
```

**d-prime (DP) by phase and reward category**

```
# Summary table and graph
aggregate(DP ~ Reward_Category + Phase, data.x2b, FUN = function(DP) c(mean = mean(DP),
    se = std.error(DP)))
```

```
##   Reward_Category             Phase    DP.mean      DP.se
## 1     High Reward      Conditioning 2.24322861 0.08396633
## 2      Low Reward      Conditioning 2.18725911 0.11189025
## 3     High Reward Post-conditioning 1.90154382 0.09704440
## 4      Low Reward Post-conditioning 1.89215403 0.10934956
```

```
x2b.DP = plot_by_group(data = data.x2b, yvar = "DP", ylim = c(0,
    4.6), ylab = "d-prime", subtitle = "Experiment 2b (All Memory)",
    tag = "1.3 B")
x2b.DP
```

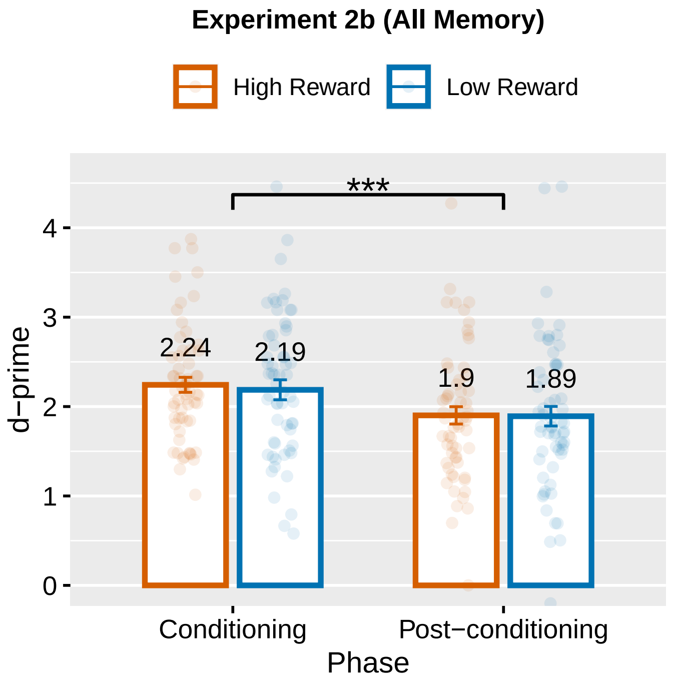


```
# Repeated measures two-factor ANOVA on d-prime scores
anova_test(DP ~ Phase * Reward_Category + Error(UserID/(Phase *
    Reward_Category)), data = data.x2b)
```

```
## ANOVA Table (type III tests)
##
##                  Effect DFn DFd      F        p p<.05      ges
## 1                 Phase   1  59 42.186 1.92e-08     * 0.040000
## 2       Reward_Category   1  59  0.221 6.40e-01       0.000442
## 3 Phase:Reward_Category   1  59  0.337 5.64e-01       0.000224
```

As found in experiment 2a, the ANOVA showed evidence for an effect of phase on corrected recognition, F(1,59) = 43.63, p < .001, $\eta^2$ = .05, and on d-prime scores F(1,59) = 42.19, p < .001, $\eta^2$ = .04. There was no significance of category-specific memory enhancement for items associated with high reward in either phases.

```
# Create subsets for each phase from data.x2a (all memory)
x2b_ph1 <- subset(data.x2b, Phase == "Conditioning")
x2b_ph2 <- subset(data.x2b, Phase == "Post-conditioning")
```

```
# Effect of reward category on memory in phase 1
# (conditioning)

# Corrected recognition (CR)
t.test(data = x2b_ph1, CR ~ Reward_Category, paired = TRUE)
```

```
##
##  Paired t-test
##
## data:  CR by Reward_Category
## t = 0.62091, df = 59, p-value = 0.5371
## alternative hypothesis: true difference in means is not equal to 0
## 95 percent confidence interval:
##  -0.02912284  0.05532771
## sample estimates:
## mean of the differences
##              0.01310244
```

```
cohens_dav(data = x2b_ph1, x = CR, group = Reward_Category)
```

```
## # A tibble: 2 x 4
##   Reward_Category count  mean    sd
##   <fct>           <int> <dbl> <dbl>
## 1 High Reward        60 0.515 0.181
## 2 Low Reward         60 0.502 0.225
## [1] "Effect size Cohen's d(av):"
## [1] 0.06459171
```

```
# d-prime (DP)
t.test(data = x2b_ph1, DP ~ Reward_Category, paired = TRUE)
```

```
##
##  Paired t-test
##
## data:  DP by Reward_Category
```

```
## t = 0.66777, df = 59, p-value = 0.5069
## alternative hypothesis: true difference in means is not equal to 0
## 95 percent confidence interval:
##  -0.1117444  0.2236834
## sample estimates:
## mean of the differences
##               0.0559695
```

```
cohens_dav(data = x2b_ph1, x = DP, group = Reward_Category)
```

```
## # A tibble: 2 x 4
##   Reward_Category count  mean    sd
##   <fct>           <int> <dbl> <dbl>
## 1 High Reward        60  2.24 0.650
## 2 Low Reward         60  2.19 0.867
## [1] "Effect size Cohen's d(av):"
## [1] 0.07378492
```

Paired t-tests reveal no significant effect of reward category in the conditioning phase (phase 1) on corrected recognition, t(59) = 0.62, p = .54, $d_{av}$ = .06, nor on d-prime scores, t(59) = 0.67, p = .51, $d_{av}$ = .07.

```
# Effect of reward category on memory in phase 2
# (post-conditioning)

# Corrected recognition (CR)
t.test(data = x2b_ph2, CR ~ Reward_Category, paired = TRUE)
```

```
##
##  Paired t-test
##
## data:  CR by Reward_Category
## t = 0.30355, df = 59, p-value = 0.7625
## alternative hypothesis: true difference in means is not equal to 0
## 95 percent confidence interval:
##  -0.03494854  0.04744789
## sample estimates:
## mean of the differences
##             0.006249674
```

```
cohens_dav(data = x2b_ph2, x = CR, group = Reward_Category)
```

```
## # A tibble: 2 x 4
##   Reward_Category count  mean    sd
##   <fct>           <int> <dbl> <dbl>
## 1 High Reward        60 0.418 0.206
## 2 Low Reward         60 0.412 0.219
## [1] "Effect size Cohen's d(av):"
## [1] 0.02944871
```

```
# d-prime (DP)
t.test(data = x2b_ph2, DP ~ Reward_Category, paired = TRUE)
```

```
##
##  Paired t-test
##
## data:  DP by Reward_Category
## t = 0.12256, df = 59, p-value = 0.9029
## alternative hypothesis: true difference in means is not equal to 0
## 95 percent confidence interval:
##  -0.1439164  0.1626960
## sample estimates:
## mean of the differences
##             0.009389787
```

```
cohens_dav(data = x2b_ph2, x = DP, group = Reward_Category)
```

```
## # A tibble: 2 x 4
##   Reward_Category count  mean    sd
##   <fct>           <int> <dbl> <dbl>
## 1 High Reward        60  1.90 0.752
## 2 Low Reward         60  1.89 0.847
## [1] "Effect size Cohen's d(av):"
## [1] 0.01174663
```

In the post-conditioning phase, again there was no evidence for a significant effect of reward category on corrected recognition, $t(59) = 0.30$, $p = .76$, $d_{av} = .01$, nor on d-prime scores, $t(59) = 0.12$, $p = .90$, $d_{av} = .01$.

## 1.4 Experiment 2b (High Certainty Memory)

**Corrected recognition (CR) by phase and reward category**

```
# Summary table and graph
aggregate(CR ~ Reward_Category + Phase, data.x2b.high, FUN = function(CR) c(mean = mean(CR),
    se = std.error(CR)))
```

```
##   Reward_Category              Phase    CR.mean      CR.se
## 1     High Reward       Conditioning 0.61648541 0.02509095
## 2      Low Reward       Conditioning 0.61330873 0.03004107
## 3     High Reward Post-conditioning 0.53097948 0.02857535
## 4      Low Reward Post-conditioning 0.51213367 0.03092514
```

```
x2b.high.CR = plot_by_group(data = data.x2b.high, yvar = "CR",
    ylim = c(0, 1.2), ylab = "Corrected Recognition", subtitle = "Experiment 2b (High Certainty)",
    tag = "1.4 A")
x2b.high.CR
```

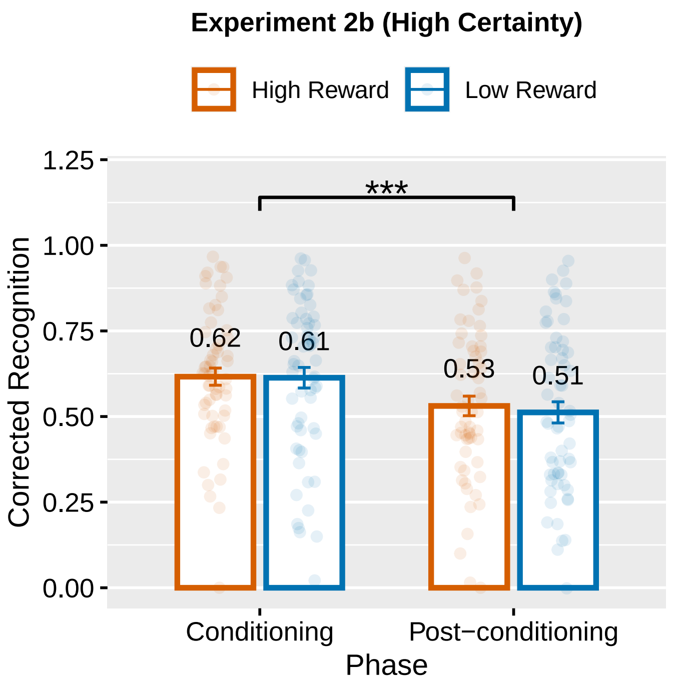


```
# Repeated measures two-factor ANOVA on corrected
# recognition (high certainty only)
anova_test(CR ~ Phase * Reward_Category + Error(UserID/(Phase *
    Reward_Category)), data = data.x2b.high)
```

```
## ANOVA Table (type III tests)
##
##                  Effect DFn DFd      F        p p<.05      ges
## 1                 Phase   1  59 35.781 1.39e-07     * 0.043000
## 2       Reward_Category   1  59  0.276 6.02e-01       0.000621
## 3 Phase:Reward_Category   1  59  0.477 4.92e-01       0.000315
```

### d-prime (DP) by phase and reward category

```
# Summary table and graph
aggregate(DP ~ Reward_Category + Phase, data.x2b.high, FUN = function(DP) c(mean = mean(DP),
    se = std.error(DP)))
```

```
##   Reward_Category            Phase   DP.mean     DP.se
## 1     High Reward     Conditioning 2.7783642 0.1203362
## 2      Low Reward     Conditioning 2.7648337 0.1310299
## 3     High Reward Post-conditioning 2.3956837 0.1189006
## 4      Low Reward Post-conditioning 2.3636009 0.1315188
```

```
x2b.high.DP = plot_by_group(data = data.x2b.high, yvar = "DP",
    ylim = c(0, 4.6), ylab = "d-prime", subtitle = "Experiment 2b (High Certainty)",
    tag = "1.4 B")
x2b.high.DP
```

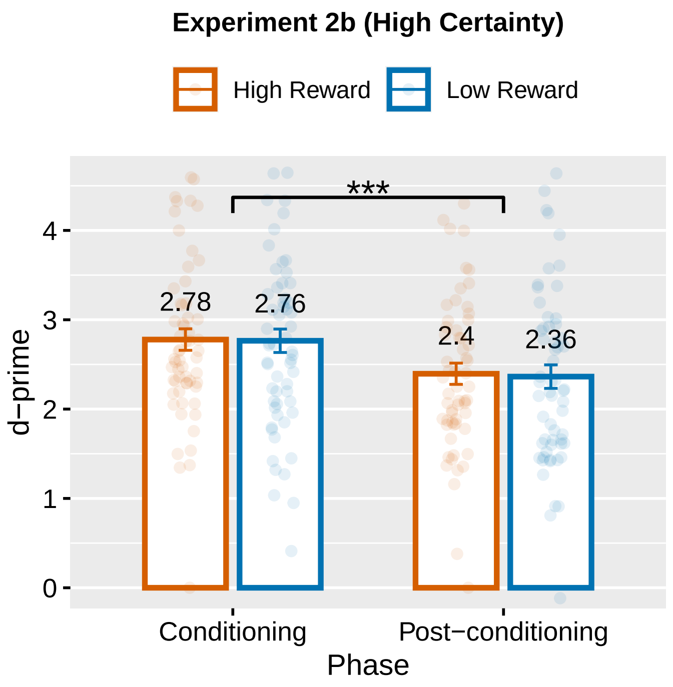


```
# Repeated measures two-factor ANOVA on d-prime scores
# (high certainty only)
anova_test(DP ~ Phase * Reward_Category + Error(UserID/(Phase *
    Reward_Category)), data = data.x2b.high)
```

```
## ANOVA Table (type III tests)
##
##                    Effect DFn DFd      F        p p<.05      ges
## 1                   Phase   1  59 40.731 2.98e-08     * 4.00e-02
## 2         Reward_Category   1  59  0.063 8.03e-01       1.40e-04
## 3 Phase:Reward_Category   1  59  0.039 8.44e-01       2.31e-05
```

```
# Create subsets for each phase from data.x2b (high
# certainty)
x2b_high_ph1 <- subset(data.x2b.high, Phase == "Conditioning")
x2b_high_ph2 <- subset(data.x2b.high, Phase == "Post-conditioning")
```

```
# Effect of reward category on high certainty memory in
# phase 1 (conditioning)

# Corrected recognition (CR)
t.test(data = x2b_high_ph1, CR ~ Reward_Category, paired = TRUE)
```

```
##
##  Paired t-test
##
## data:  CR by Reward_Category
```

```
## t = 0.13244, df = 59, p-value = 0.8951
## alternative hypothesis: true difference in means is not equal to 0
## 95 percent confidence interval:
##  -0.04481743  0.05117080
## sample estimates:
## mean of the differences
##             0.003176682
```

```
cohens_dav(data = x2b_high_ph1, x = CR, group = Reward_Category)
```

```
## # A tibble: 2 x 4
##   Reward_Category count  mean    sd
##   <fct>           <int> <dbl> <dbl>
## 1 High Reward        60 0.616 0.194
## 2 Low Reward         60 0.613 0.233
## [1] "Effect size Cohen's d(av):"
## [1] 0.0148773
```

```
# d-prime (DP)
t.test(data = x2b_high_ph1, DP ~ Reward_Category, paired = TRUE)
```

```
##
##  Paired t-test
##
## data:  DP by Reward_Category
## t = 0.13176, df = 59, p-value = 0.8956
## alternative hypothesis: true difference in means is not equal to 0
## 95 percent confidence interval:
##  -0.1919528  0.2190138
## sample estimates:
## mean of the differences
##              0.01353049
```

```
cohens_dav(data = x2b_high_ph1, x = DP, group = Reward_Category)
```

```
## # A tibble: 2 x 4
##   Reward_Category count  mean    sd
##   <fct>           <int> <dbl> <dbl>
## 1 High Reward        60  2.78 0.932
## 2 Low Reward         60  2.76 1.01
## [1] "Effect size Cohen's d(av):"
## [1] 0.01389828
```

```
# Effect of reward category on high certainty memory in
# phase 2 (post-conditioning)

# Corrected recognition (CR)
t.test(data = x2b_high_ph2, CR ~ Reward_Category, paired = TRUE)
```

```
##
##  Paired t-test
```

```
##
## data:  CR by Reward_Category
## t = 0.79503, df = 59, p-value = 0.4298
## alternative hypothesis: true difference in means is not equal to 0
## 95 percent confidence interval:
##  -0.02858703  0.06627866
## sample estimates:
## mean of the differences
##              0.01884581
```

```
cohens_dav(data = x2b_high_ph2, x = CR, group = Reward_Category)
```

```
## # A tibble: 2 x 4
##   Reward_Category count  mean    sd
##   <fct>           <int> <dbl> <dbl>
## 1 High Reward        60 0.531 0.221
## 2 Low Reward         60 0.512 0.240
## [1] "Effect size Cohen's d(av):"
## [1] 0.0817803
```

```
# d-prime (DP)
t.test(data = x2b_high_ph2, DP ~ Reward_Category, paired = TRUE)
```

```
##
##  Paired t-test
##
## data:  DP by Reward_Category
## t = 0.31422, df = 59, p-value = 0.7545
## alternative hypothesis: true difference in means is not equal to 0
## 95 percent confidence interval:
##  -0.1722272  0.2363928
## sample estimates:
## mean of the differences
##              0.03208279
```

```
cohens_dav(data = x2b_high_ph2, x = DP, group = Reward_Category)
```

```
## # A tibble: 2 x 4
##   Reward_Category count  mean    sd
##   <fct>           <int> <dbl> <dbl>
## 1 High Reward        60  2.40 0.921
## 2 Low Reward         60  2.36 1.02
## [1] "Effect size Cohen's d(av):"
## [1] 0.03307947
```

When considering only high certainty responses on the memory task from experiment 2b, the results were consistent with the full analysis (on all memory trials). Again, the main effect of phase was evidence both on corrected recognition and d-prime scores (all p values $< .001$) and no effect on reward category or an interaction effect between phase and reward category.

# 2. Complementary Bayesian t-tests

As complementary analysis to classical paired t-tests conducted above, which have resulted in inconclusive evidence for category selective retrospective or prospective memory enhancement effects, we additionally used Bayesian analysis to confirm whether our data supported the null hypothesis of no effect. We used Bayesian paired t-tests using ttestBF function in R, with the alternative hypothesis (H1) supporting a positive memory effect for high reward items compared to low reward items overall and from each phase, whereas the null hypothesis (H0) represents zero effect [Jarosz and Wiley, 2014,Rouder et al., 2009]

Bayes factors were calculated to test whether the null hypothesis H0 (true effect is equal to zero) holds against the one-sided alternative hypothesis H1 (effect is greater than zero). In the below analysis we used a Cauchy prior distribution with a default scale parameter of r = .707 and interpreted the Bayes factor ($BF_{10}$) as follows:

- $BF_{10} < 1/3$ : Substantial evidence for H0
- $1/3 < BF_{10} < 1$ : Anecdotal evidence for H0
- $1 < BF_{10} < 3$ : Anecdotal evidence for H1
- $BF_{10} > 3$ : Substantial evidence for H1

## 2.1 Experiment 2a (All Memory)

```
# Effect of reward category on memory in phase 1
# (pre-conditioning) Corrected recognition (CR) Two-sided
# test
ttestBF(x = x2a_ph1$CR[x2a_ph1$Reward_Category == "High Reward"],
    y = x2a_ph1$CR[x2a_ph1$Reward_Category == "Low Reward"],
    paired = TRUE)
```

```
## Bayes factor analysis
## --------------
## [1] Alt., r=0.707 : 24.00207 ±0%
##
## Against denominator:
##   Null, mu = 0
## ---
## Bayes factor type: BFoneSample, JZS
```

```
# One-sided test
ttestBF(x = x2a_ph1$CR[x2a_ph1$Reward_Category == "High Reward"],
    y = x2a_ph1$CR[x2a_ph1$Reward_Category == "Low Reward"],
    nullInterval = c(-Inf, 0), paired = TRUE)
```

```
## Bayes factor analysis
## --------------
## [1] Alt., r=0.707 -Inf<d<0    : 47.97006   ±0%
## [2] Alt., r=0.707 !(-Inf<d<0) : 0.03408772 ±0.36%
##
## Against denominator:
##   Null, mu = 0
## ---
## Bayes factor type: BFoneSample, JZS
```

```r
# d-prime Two-sided test
ttestBF(x = x2a_ph1$DP[x2a_ph1$Reward_Category == "High Reward"],
    y = x2a_ph1$DP[x2a_ph1$Reward_Category == "Low Reward"],
    paired = TRUE)
```

```
## Bayes factor analysis
## --------------
## [1] Alt., r=0.707 : 14.61927 ±0%
##
## Against denominator:
##   Null, mu = 0
## ---
## Bayes factor type: BFoneSample, JZS
```

```r
# One-sided test
ttestBF(x = x2a_ph1$DP[x2a_ph1$Reward_Category == "High Reward"],
    y = x2a_ph1$DP[x2a_ph1$Reward_Category == "Low Reward"],
    nullInterval = c(-Inf, 0), paired = TRUE)
```

```
## Bayes factor analysis
## --------------
## [1] Alt., r=0.707 -Inf<d<0    : 29.20309   ±0%
## [2] Alt., r=0.707 !(-Inf<d<0) : 0.03544927 ±0%
##
## Against denominator:
##   Null, mu = 0
## ---
## Bayes factor type: BFoneSample, JZS
```

In the pre-conditioning phase of experiment 2a, Bayesian t-tests suggested that data is more probable under the alternative hypothesis (H1: that there is an effect of reward category on memory) with $BF_{10} = 24.00$ for corrected recognition and $BF_{10} = 12.62$ for d-prime scores. The one-sided t-test with alternative hypothesis of a negative effect supported this with a $BF_{10} = 47.97$ for corrected recognition and $BF_{10} = 29.20$ for d-prime scores. Again suggesting a strong effect of reward category, albeit in the opposite direction to the original study (items belonging to low reward category resulted in enhanced memory). These results are consistent with the findings from classical t-tests performed in section 1.1 of this document.

```r
# Effect of reward category on memory in phase 2
# (conditioning) Corrected recognition (CR) Two-sided test
ttestBF(x = x2a_ph2$CR[x2a_ph2$Reward_Category == "High Reward"],
    y = x2a_ph2$CR[x2a_ph2$Reward_Category == "Low Reward"],
    paired = TRUE)
```

```
## Bayes factor analysis
## --------------
## [1] Alt., r=0.707 : 1.604747 ±0%
##
## Against denominator:
##   Null, mu = 0
## ---
## Bayes factor type: BFoneSample, JZS
```

```r
# One-sided test
ttestBF(x = x2a_ph2$CR[x2a_ph2$Reward_Category == "High Reward"],
    y = x2a_ph2$CR[x2a_ph2$Reward_Category == "Low Reward"],
    nullInterval = c(-Inf, 0), paired = TRUE)
```

```
## Bayes factor analysis
## --------------
## [1] Alt., r=0.707 -Inf<d<0    : 3.164267   ±0%
## [2] Alt., r=0.707 !(-Inf<d<0) : 0.04522745 ±0%
##
## Against denominator:
##   Null, mu = 0
## ---
## Bayes factor type: BFoneSample, JZS
```

```r
# d-prime Two-sided test
ttestBF(x = x2a_ph2$DP[x2a_ph2$Reward_Category == "High Reward"],
    y = x2a_ph2$DP[x2a_ph2$Reward_Category == "Low Reward"],
    paired = TRUE)
```

```
## Bayes factor analysis
## --------------
## [1] Alt., r=0.707 : 0.3189318 ±0%
##
## Against denominator:
##   Null, mu = 0
## ---
## Bayes factor type: BFoneSample, JZS
```

```r
# One-sided test
ttestBF(x = x2a_ph2$DP[x2a_ph2$Reward_Category == "High Reward"],
    y = x2a_ph2$DP[x2a_ph2$Reward_Category == "Low Reward"],
    nullInterval = c(-Inf, 0), paired = TRUE)
```

```
## Bayes factor analysis
## --------------
## [1] Alt., r=0.707 -Inf<d<0    : 0.572838   ±0%
## [2] Alt., r=0.707 !(-Inf<d<0) : 0.06502551 ±0.06%
##
## Against denominator:
##   Null, mu = 0
## ---
## Bayes factor type: BFoneSample, JZS
```

In the conditioning phase of experiment 2a, Bayesian t-tests suggested that data is marginally more probable under the alternative hypothesis (H1: that there is an effect of reward category on memory) with $BF_{10} = 1.60$ for corrected recognition. When considering d-primes scores, the Bayes factor suggested comparatively stronger evidence for the null hypothesis, $BF_{10} = 0.32$. The one-sided t-test showed anecdotal evidence for the hypothesis that there is a negative memory effect (items from low reward category are enhanced in memory over items from high reward category), $BF_{10} = 3.16$ for corrected recognition. However, the same was not true with d-prime scores, $BF_{10} = 0.573$, which in turn marginally favors the null. These results are consistent with the findings from classical t-tests performed in section 1.1 of this document which also suggested weak effects. Additionally, it is again noteworthy that analysing corrected recognition scores have resulted in different conclusions than when considering d-primes scores.

## 2.2 Experiment 2a (High Certainty Memory)

```
# Effect of reward category on high certainty memory in
# phase 1 (pre-conditioning) Corrected recognition (CR)
# Two-sided test
ttestBF(x = x2a_high_ph1$CR[x2a_high_ph1$Reward_Category == "High Reward"],
    y = x2a_high_ph1$CR[x2a_high_ph1$Reward_Category == "Low Reward"],
    paired = TRUE)
```

```
## Bayes factor analysis
## --------------
## [1] Alt., r=0.707 : 0.4844523 ±0%
##
## Against denominator:
##   Null, mu = 0
## ---
## Bayes factor type: BFoneSample, JZS
```

```
# One-sided test
ttestBF(x = x2a_high_ph1$CR[x2a_high_ph1$Reward_Category == "High Reward"],
    y = x2a_high_ph1$CR[x2a_high_ph1$Reward_Category == "Low Reward"],
    nullInterval = c(-Inf, 0), paired = TRUE)
```

```
## Bayes factor analysis
## --------------
## [1] Alt., r=0.707 -Inf<d<0    : 0.9117099  ±0%
## [2] Alt., r=0.707 !(-Inf<d<0) : 0.05719458 ±0.04%
##
## Against denominator:
##   Null, mu = 0
## ---
## Bayes factor type: BFoneSample, JZS
```

```
# d-prime Two-sided test
ttestBF(x = x2a_high_ph1$DP[x2a_high_ph1$Reward_Category == "High Reward"],
    y = x2a_high_ph1$DP[x2a_high_ph1$Reward_Category == "Low Reward"],
    paired = TRUE)
```

```
## Bayes factor analysis
## --------------
## [1] Alt., r=0.707 : 0.3197755 ±0%
##
## Against denominator:
##   Null, mu = 0
## ---
## Bayes factor type: BFoneSample, JZS
```

```
# One-sided test
ttestBF(x = x2a_high_ph1$DP[x2a_high_ph1$Reward_Category == "High Reward"],
    y = x2a_high_ph1$DP[x2a_high_ph1$Reward_Category == "Low Reward"],
    nullInterval = c(-Inf, 0), paired = TRUE)
```

```
## Bayes factor analysis
## --------------
## [1] Alt., r=0.707 -Inf<d<0    : 0.5745876  ±0%
## [2] Alt., r=0.707 !(-Inf<d<0) : 0.06496347 ±0.06%
##
## Against denominator:
##   Null, mu = 0
## ---
## Bayes factor type: BFoneSample, JZS
```

When considering only high certainty memory, Bayes factors suggested anecdotal evidence in favor of the null hypothesis that there is no effect of reward category on memory, $BF_{10} = .484$ with corrected recognition and $BF_{10} = .320$ with d-prime scores. The one-sided t-tests also favor the null hypothesis although the evidence was only anecdotal, $BF_{10} = .912$ for corrected recognition and $BF_{10} = .575$ for d-prime scores. This is consistent with results of the frequentist t-tests.

```
# Effect of reward category on high certainty memory in
# phase 2 (conditioning) Corrected recognition (CR)
# Two-sided test
ttestBF(x = x2a_high_ph2$CR[x2a_high_ph2$Reward_Category == "High Reward"],
    y = x2a_high_ph2$CR[x2a_high_ph2$Reward_Category == "Low Reward"],
    paired = TRUE)
```

```
## Bayes factor analysis
## --------------
## [1] Alt., r=0.707 : 0.1719523 ±0%
##
## Against denominator:
##   Null, mu = 0
## ---
## Bayes factor type: BFoneSample, JZS
```

```
# One-sided test
ttestBF(x = x2a_high_ph2$CR[x2a_high_ph2$Reward_Category == "High Reward"],
    y = x2a_high_ph2$CR[x2a_high_ph2$Reward_Category == "Low Reward"],
    nullInterval = c(-Inf, 0), paired = TRUE)
```

```
## Bayes factor analysis
## --------------
## [1] Alt., r=0.707 -Inf<d<0    : 0.2523732  ±0%
## [2] Alt., r=0.707 !(-Inf<d<0) : 0.09153136 ±0%
##
## Against denominator:
##   Null, mu = 0
## ---
## Bayes factor type: BFoneSample, JZS
```

```
# d-prime Two-sided test
ttestBF(x = x2a_high_ph2$DP[x2a_high_ph2$Reward_Category == "High Reward"],
    y = x2a_high_ph2$DP[x2a_high_ph2$Reward_Category == "Low Reward"],
    paired = TRUE)
```

```
## Bayes factor analysis
## --------------
## [1] Alt., r=0.707 : 0.1548738 ±0%
##
## Against denominator:
##   Null, mu = 0
## ---
## Bayes factor type: BFoneSample, JZS
```

```
# One-sided test
ttestBF(x = x2a_high_ph2$DP[x2a_high_ph2$Reward_Category == "High Reward"],
    y = x2a_high_ph2$DP[x2a_high_ph2$Reward_Category == "Low Reward"],
    nullInterval = c(-Inf, 0), paired = TRUE)
```

```
## Bayes factor analysis
## --------------
## [1] Alt., r=0.707 -Inf<d<0    : 0.103624  ±0%
## [2] Alt., r=0.707 !(-Inf<d<0) : 0.2061236 ±0%
##
## Against denominator:
##   Null, mu = 0
## ---
## Bayes factor type: BFoneSample, JZS
```

With only high certainty memory for the conditioning phase of experiment 2a, Bayes factors suggested substantial evidence in favor of the null hypothesis that there is no effect of reward category on memory, $BF_{10} = .172$ with corrected recognition and $BF_{10} = .155$ with d-prime scores. The one-sided t-tests also provided substantial evidence for the null hypothesis, all Bayes factors $< 0.33$.

## 2.3 Experiment 2b (All Memory)

```
# Effect of reward category on memory in phase 1
# (conditioning) Corrected recognition (CR) Two-sided test
ttestBF(x = x2b_ph1$CR[x2b_ph1$Reward_Category == "High Reward"],
    y = x2b_ph1$CR[x2b_ph1$Reward_Category == "Low Reward"],
    paired = TRUE)
```

```
## Bayes factor analysis
## --------------
## [1] Alt., r=0.707 : 0.1697932 ±0%
##
## Against denominator:
##   Null, mu = 0
## ---
## Bayes factor type: BFoneSample, JZS
```

```
# One-sided test
ttestBF(x = x2b_ph1$CR[x2b_ph1$Reward_Category == "High Reward"],
    y = x2b_ph1$CR[x2b_ph1$Reward_Category == "Low Reward"],
    nullInterval = c(-Inf, 0), paired = TRUE)
```

```
## Bayes factor analysis
## --------------
## [1] Alt., r=0.707 -Inf<d<0    : 0.09266861 ±0%
## [2] Alt., r=0.707 !(-Inf<d<0) : 0.2469178  ±0%
##
## Against denominator:
##   Null, mu = 0
## ---
## Bayes factor type: BFoneSample, JZS
```

```
# d-prime Two-sided test
ttestBF(x = x2b_ph1$DP[x2b_ph1$Reward_Category == "High Reward"],
    y = x2b_ph1$DP[x2b_ph1$Reward_Category == "Low Reward"],
    paired = TRUE)
```

```
## Bayes factor analysis
## --------------
## [1] Alt., r=0.707 : 0.1747443 ±0%
##
## Against denominator:
##   Null, mu = 0
## ---
## Bayes factor type: BFoneSample, JZS
```

```
# One-sided test
ttestBF(x = x2b_ph1$DP[x2b_ph1$Reward_Category == "High Reward"],
    y = x2b_ph1$DP[x2b_ph1$Reward_Category == "Low Reward"],
    nullInterval = c(-Inf, 0), paired = TRUE)
```

```
## Bayes factor analysis
## --------------
## [1] Alt., r=0.707 -Inf<d<0    : 0.09016576 ±0%
## [2] Alt., r=0.707 !(-Inf<d<0) : 0.2593229  ±0%
##
## Against denominator:
##   Null, mu = 0
## ---
## Bayes factor type: BFoneSample, JZS
```

In the conditioning phase of experiment 2b, the Bayes factors suggested substantial evidence in favor of the null hypothesis that there is no memory enhancement for items specifically in the high or low reward category, $BF_{10} = .170$ with corrected recognition and $BF_{10} = .175$ with d-prime scores. The one-sided t-tests also provided substantial evidence for the null hypothesis, all Bayes factors $< 0.33$.

```
# Effect of reward category on memory in phase 2
# (post-conditioning) Corrected recognition (CR) Two-sided
# test
ttestBF(x = x2b_ph2$CR[x2b_ph2$Reward_Category == "High Reward"],
    y = x2b_ph2$CR[x2b_ph2$Reward_Category == "Low Reward"],
    paired = TRUE)
```

```
## Bayes factor analysis
```

```
## ---------------
## [1] Alt., r=0.707 : 0.147607 ±0%
##
## Against denominator:
##   Null, mu = 0
## ---
## Bayes factor type: BFoneSample, JZS
```

```
# One-sided test
ttestBF(x = x2b_ph2$CR[x2b_ph2$Reward_Category == "High Reward"],
    y = x2b_ph2$CR[x2b_ph2$Reward_Category == "Low Reward"],
    nullInterval = c(-Inf, 0), paired = TRUE)
```

```
## Bayes factor analysis
## ---------------
## [1] Alt., r=0.707 -Inf<d<0    : 0.1132838 ±0%
## [2] Alt., r=0.707 !(-Inf<d<0) : 0.1819302 ±0%
##
## Against denominator:
##   Null, mu = 0
## ---
## Bayes factor type: BFoneSample, JZS
```

```
# d-prime Two-sided test
ttestBF(x = x2b_ph2$DP[x2b_ph2$Reward_Category == "High Reward"],
    y = x2b_ph2$DP[x2b_ph2$Reward_Category == "Low Reward"],
    paired = TRUE)
```

```
## Bayes factor analysis
## ---------------
## [1] Alt., r=0.707 : 0.1422567 ±0%
##
## Against denominator:
##   Null, mu = 0
## ---
## Bayes factor type: BFoneSample, JZS
```

```
# One-sided test
ttestBF(x = x2b_ph2$DP[x2b_ph2$Reward_Category == "High Reward"],
    y = x2b_ph2$DP[x2b_ph2$Reward_Category == "Low Reward"],
    nullInterval = c(-Inf, 0), paired = TRUE)
```

```
## Bayes factor analysis
## ---------------
## [1] Alt., r=0.707 -Inf<d<0    : 0.1287339 ±0%
## [2] Alt., r=0.707 !(-Inf<d<0) : 0.1557796 ±0%
##
## Against denominator:
##   Null, mu = 0
## ---
## Bayes factor type: BFoneSample, JZS
```

As in the conditioning phase, for the post-conditioning phase of experiment 2b the Bayes factor showed substantial evidence in favor of the null hypothesis that there is no memory advantage for items specifically in the high or low reward category, $BF_{10} = .148$ with corrected recognition and $BF_{10} = .142$ with d-prime scores. The one-sided t-tests also provided substantial evidence for the null hypothesis, all Bayes factors < 0.33.

## 2.4 Experiment 2b (High Certainty Memory)

```
# Effect of reward category on high certainty memory in
# phase 1 (conditioning) Corrected recognition (CR)
# Two-sided test
ttestBF(x = x2b_high_ph1$CR[x2b_high_ph1$Reward_Category == "High Reward"],
    y = x2b_high_ph1$CR[x2b_high_ph1$Reward_Category == "Low Reward"],
    paired = TRUE)
```

```
## Bayes factor analysis
## --------------
## [1] Alt., r=0.707 : 0.1424286 ±0%
##
## Against denominator:
##   Null, mu = 0
## ---
## Bayes factor type: BFoneSample, JZS
```

```
# One-sided test
ttestBF(x = x2b_high_ph1$CR[x2b_high_ph1$Reward_Category == "High Reward"],
    y = x2b_high_ph1$CR[x2b_high_ph1$Reward_Category == "Low Reward"],
    nullInterval = c(-Inf, 0), paired = TRUE)
```

```
## Bayes factor analysis
## --------------
## [1] Alt., r=0.707 -Inf<d<0    : 0.1278033 ±0%
## [2] Alt., r=0.707 !(-Inf<d<0) : 0.1570539 ±0%
##
## Against denominator:
##   Null, mu = 0
## ---
## Bayes factor type: BFoneSample, JZS
```

```
# d-prime Two-sided test
ttestBF(x = x2b_high_ph1$DP[x2b_high_ph1$Reward_Category == "High Reward"],
    y = x2b_high_ph1$DP[x2b_high_ph1$Reward_Category == "Low Reward"],
    paired = TRUE)
```

```
## Bayes factor analysis
## --------------
## [1] Alt., r=0.707 : 0.1424163 ±0%
##
## Against denominator:
##   Null, mu = 0
## ---
## Bayes factor type: BFoneSample, JZS
```

```r
# One-sided test
ttestBF(x = x2b_high_ph1$DP[x2b_high_ph1$Reward_Category == "High Reward"],
    y = x2b_high_ph1$DP[x2b_high_ph1$Reward_Category == "Low Reward"],
    nullInterval = c(-Inf, 0), paired = TRUE)
```

```
## Bayes factor analysis
## --------------
## [1] Alt., r=0.707 -Inf<d<0    : 0.1278674 ±0%
## [2] Alt., r=0.707 !(-Inf<d<0) : 0.1569652 ±0%
##
## Against denominator:
##   Null, mu = 0
## ---
## Bayes factor type: BFoneSample, JZS
```

With high certainty memory in the conditioning phase of experiment 2b, the Bayes factors suggested substantial evidence in favor of the null hypothesis that there is no memory enhancement for items specifically in the high or low reward category, $BF_{10} = .142$ with corrected recognition and $BF_{10} = .142$ with d-prime scores. The one-sided t-tests also provided substantial evidence for the null hypothesis, all Bayes factors $<$ 0.33.

```r
# Effect of reward category on high certainty memory in
# phase 2 (post-conditioning) Corrected recognition (CR)
# Two-sided test
ttestBF(x = x2b_high_ph2$CR[x2b_high_ph2$Reward_Category == "High Reward"],
    y = x2b_high_ph2$CR[x2b_high_ph2$Reward_Category == "Low Reward"],
    paired = TRUE)
```

```
## Bayes factor analysis
## --------------
## [1] Alt., r=0.707 : 0.1908973 ±0%
##
## Against denominator:
##   Null, mu = 0
## ---
## Bayes factor type: BFoneSample, JZS
```

```r
# One-sided test
ttestBF(x = x2b_high_ph2$CR[x2b_high_ph2$Reward_Category == "High Reward"],
    y = x2b_high_ph2$CR[x2b_high_ph2$Reward_Category == "Low Reward"],
    nullInterval = c(-Inf, 0), paired = TRUE)
```

```
## Bayes factor analysis
## --------------
## [1] Alt., r=0.707 -Inf<d<0    : 0.08393302 ±0.06%
## [2] Alt., r=0.707 !(-Inf<d<0) : 0.2978615  ±0%
##
## Against denominator:
##   Null, mu = 0
## ---
## Bayes factor type: BFoneSample, JZS
```

```r
# d-prime Two-sided test
ttestBF(x = x2b_high_ph2$DP[x2b_high_ph2$Reward_Category == "High Reward"],
    y = x2b_high_ph2$DP[x2b_high_ph2$Reward_Category == "Low Reward"],
    paired = TRUE)
```

```
## Bayes factor analysis
## --------------
## [1] Alt., r=0.707 : 0.1480732 ±0%
##
## Against denominator:
##   Null, mu = 0
## ---
## Bayes factor type: BFoneSample, JZS
```

```r
# One-sided test
ttestBF(x = x2b_high_ph2$DP[x2b_high_ph2$Reward_Category == "High Reward"],
    y = x2b_high_ph2$DP[x2b_high_ph2$Reward_Category == "Low Reward"],
    nullInterval = c(-Inf, 0), paired = TRUE)
```

```
## Bayes factor analysis
## --------------
## [1] Alt., r=0.707 -Inf<d<0    : 0.1124689 ±0%
## [2] Alt., r=0.707 !(-Inf<d<0) : 0.1836775 ±0%
##
## Against denominator:
##   Null, mu = 0
## ---
## Bayes factor type: BFoneSample, JZS
```

As in the conditioning phase, high certainty memory in the post-conditioning phase of experiment 2b showed substantial evidence in favor of the null hypothesis that there is effect of reward category on memory, $BF_{10}$ = .191 with corrected recognition and $BF_{10}$ = .148 with d-prime scores. The one-sided t-tests also provided substantial evidence for the null hypothesis, all Bayes factors $< 0.33$.

# 3. Summary Graphs & Tables

## 3.1 Memory Performance Graphs

```r
x2a.CR = plot_by_group(data = data.x2a, yvar = "CR", ylim = c(0,
    1.2), ylab = "Corrected Recognition", xlab = NULL, subtitle = "All Memory",
    tag = "A")
x2a.high.CR = plot_by_group(data = data.x2a.high, yvar = "CR",
    ylim = c(0, 1.2), ylab = "Corrected Recognition", xlab = NULL,
    subtitle = "High Certainty Memory", tag = "B")
x2a.DP = plot_by_group(data = data.x2a, yvar = "DP", ylim = c(0,
    4.6), ylab = "d-prime", xlab = NULL, subtitle = "All Memory",
    tag = "C")
x2a.high.DP = plot_by_group(data = data.x2a.high, yvar = "DP",
    ylim = c(0, 4.6), ylab = "d-prime", xlab = NULL, subtitle = "High Certainty Memory",
```

```
    tag = "D")

summary.x2a <- ggarrange(x2a.CR, x2a.high.CR, x2a.DP, x2a.high.DP,
    ncol = 2, nrow = 2, common.legend = TRUE, legend = "top")
# summary.x2a <- annotate_figure(summary.x2a, top =
# text_grob('Experiment 2a', face = 'bold', size = 12))
ggsave(file = "summary.x2a.svg", plot = summary.x2a, width = 8,
    height = 6.5)
ggsave(file = "summary.x2a.jpg", plot = summary.x2a, width = 8,
    height = 6.5)
```

```
# black and white figures
x2a.CR = plot_by_group_bw(data = data.x2a, yvar = "CR", ylim = c(0,
    1.2), ylab = "Corrected Recognition", xlab = NULL, subtitle = "All Memory",
    tag = "A")
x2a.high.CR = plot_by_group_bw(data = data.x2a.high, yvar = "CR",
    ylim = c(0, 1.2), ylab = "Corrected Recognition", xlab = NULL,
    subtitle = "High Certainty Memory", tag = "B")
x2a.DP = plot_by_group_bw(data = data.x2a, yvar = "DP", ylim = c(0,
    4.6), ylab = "d-prime", xlab = NULL, subtitle = "All Memory",
    tag = "C")
x2a.high.DP = plot_by_group_bw(data = data.x2a.high, yvar = "DP",
    ylim = c(0, 4.6), ylab = "d-prime", xlab = NULL, subtitle = "High Certainty Memory",
    tag = "D")

bw.summary.x2a <- ggarrange(x2a.CR, x2a.high.CR, x2a.DP, x2a.high.DP,
    ncol = 2, nrow = 2, common.legend = TRUE, legend = "top")
bw.summary.x2a <- annotate_figure(bw.summary.x2a, top = text_grob("Experiment 2a",
    face = "bold", size = 12))
ggsave(file = "bw.summary.x2a.svg", plot = bw.summary.x2a, width = 8,
    height = 6.5)
ggsave(file = "bw.summary.x2a.jpg", plot = bw.summary.x2a, width = 8,
    height = 6.5)
```

```
x2b.CR = plot_by_group(data = data.x2b, yvar = "CR", ylim = c(0,
    1.2), ylab = "Corrected Recognition", xlab = NULL, subtitle = "All Memory",
    tag = "A")
x2b.high.CR = plot_by_group(data = data.x2b.high, yvar = "CR",
    ylim = c(0, 1.2), ylab = "Corrected Recognition", xlab = NULL,
    subtitle = "High Certainty Memory", tag = "B")
x2b.DP = plot_by_group(data = data.x2b, yvar = "DP", ylim = c(0,
    4.6), ylab = "d-prime", xlab = NULL, subtitle = "All Memory",
    tag = "C")
x2b.high.DP = plot_by_group(data = data.x2b.high, yvar = "DP",
    ylim = c(0, 4.6), ylab = "d-prime", xlab = NULL, subtitle = "High Certainty Memory",
    tag = "D")

summary.x2b <- ggarrange(x2b.CR, x2b.high.CR, x2b.DP, x2b.high.DP,
    ncol = 2, nrow = 2, common.legend = TRUE, legend = "top")
# summary.x2b <- annotate_figure(summary.x2b, top =
# text_grob('Experiment 2b', face = 'bold', size = 12))
ggsave(file = "summary.x2b.svg", plot = summary.x2b, width = 8,
    height = 6.5)
```

```
ggsave(file = "summary.x2b.jpg", plot = summary.x2b, width = 8,
    height = 6.5)
```

```
# black and white figures
x2b.CR = plot_by_group_bw(data = data.x2b, yvar = "CR", ylim = c(0,
    1.2), ylab = "Corrected Recognition", xlab = NULL, subtitle = "All Memory",
    tag = "A")
x2b.high.CR = plot_by_group_bw(data = data.x2b.high, yvar = "CR",
    ylim = c(0, 1.2), ylab = "Corrected Recognition", xlab = NULL,
    subtitle = "High Certainty Memory", tag = "B")
x2b.DP = plot_by_group_bw(data = data.x2b, yvar = "DP", ylim = c(0,
    4.6), ylab = "d-prime", xlab = NULL, subtitle = "All Memory",
    tag = "C")
x2b.high.DP = plot_by_group_bw(data = data.x2b.high, yvar = "DP",
    ylim = c(0, 4.6), ylab = "d-prime", xlab = NULL, subtitle = "High Certainty Memory",
    tag = "D")

bw.summary.x2b <- ggarrange(x2b.CR, x2b.high.CR, x2b.DP, x2b.high.DP,
    ncol = 2, nrow = 2, common.legend = TRUE, legend = "top")
bw.summary.x2b <- annotate_figure(bw.summary.x2b, top = text_grob("Experiment 2b",
    face = "bold", size = 12))
ggsave(file = "bw.summary.x2b.svg", plot = bw.summary.x2b, width = 8,
    height = 6.5)
ggsave(file = "bw.summary.x2b.jpg", plot = bw.summary.x2b, width = 8,
    height = 6.5)
```

## 3.2 Memory Performance by Certainty

Create tables to see how memory responses vary by certainty, coded: 0 = definitely old; 12 = likely old; 24 = maybe old; 48 = maybe new; 60 = likely new, 72 = definitely new.

```
# Experiment 2a
data.cert.x2a <- read.csv("adaptiveMemoryReplication/Exp2a_CleanData/Supp/x2a_Certainty.csv")
ph1_hr <- subset(data.cert.x2a, Phase == "1" & Reward_Category ==
    "1") %>%
    group_by(Certainty) %>%
    summarize(mean_size = mean(Proportion, na.rm = TRUE))
ph1_lr <- subset(data.cert.x2a, Phase == "1" & Reward_Category ==
    "-1") %>%
    group_by(Certainty) %>%
    summarize(mean_size = mean(Proportion, na.rm = TRUE))
ph2_hr <- subset(data.cert.x2a, Phase == "2" & Reward_Category ==
    "1") %>%
    group_by(Certainty) %>%
    summarize(mean_size = mean(Proportion, na.rm = TRUE))
ph2_lr <- subset(data.cert.x2a, Phase == "2" & Reward_Category ==
    "-1") %>%
    group_by(Certainty) %>%
    summarize(mean_size = mean(Proportion, na.rm = TRUE))
new_hr <- subset(data.cert.x2a, Phase == "New" & Reward_Category ==
    "1") %>%
    group_by(Certainty) %>%
```

**Table 1**

*Experiment 2a, Mean Proportion of Memory Responses by Certainty*

| | High Reward | | | | | |
|---|---|---|---|---|---|---|
| Measure | DO | LO | MO | MN | LN | DN |
| Pre-conditioning | 0.407 | 0.145 | 0.136 | 0.161 | 0.144 | 0.117 |
| Conditioning | 0.452 | 0.141 | 0.157 | 0.146 | 0.119 | 0.114 |
| New | 0.058 | 0.074 | 0.107 | 0.235 | 0.246 | 0.322 |
| | **Low Reward** | | | | | |
| Measure | DO | LO | MO | MN | LN | DN |
| Pre-conditioning | 0.404 | 0.180 | 0.141 | 0.141 | 0.138 | 0.107 |
| Conditioning | 0.451 | 0.171 | 0.149 | 0.128 | 0.121 | 0.089 |
| New | 0.054 | 0.064 | 0.107 | 0.235 | 0.273 | 0.323 |

*Note:* DO = definitely old; LO = likely old; MO = maybe old; MN = maybe new; LN = likely new, DN = definitely new.

```
    summarize(mean_size = mean(Proportion, na.rm = TRUE))
new_lr <- subset(data.cert.x2a, Phase == "New" & Reward_Category ==
    "-1") %>%
    group_by(Certainty) %>%
    summarize(mean_size = mean(Proportion, na.rm = TRUE))
```

```
# Experiment 2b
data.cert.x2b <- read.csv("adaptiveMemoryReplication/Exp2b_CleanData/Supp/x2b_Certainty.csv")
ph1_hr <- subset(data.cert.x2b, Phase == "1" & Reward_Category ==
    "1") %>%
    group_by(Certainty) %>%
    summarize(mean_size = mean(Proportion, na.rm = TRUE))
ph1_lr <- subset(data.cert.x2b, Phase == "1" & Reward_Category ==
    "-1") %>%
    group_by(Certainty) %>%
    summarize(mean_size = mean(Proportion, na.rm = TRUE))
ph2_hr <- subset(data.cert.x2b, Phase == "2" & Reward_Category ==
    "1") %>%
    group_by(Certainty) %>%
    summarize(mean_size = mean(Proportion, na.rm = TRUE))
ph2_lr <- subset(data.cert.x2b, Phase == "2" & Reward_Category ==
    "-1") %>%
    group_by(Certainty) %>%
    summarize(mean_size = mean(Proportion, na.rm = TRUE))
new_hr <- subset(data.cert.x2b, Phase == "New" & Reward_Category ==
    "1") %>%
    group_by(Certainty) %>%
    summarize(mean_size = mean(Proportion, na.rm = TRUE))
new_lr <- subset(data.cert.x2b, Phase == "New" & Reward_Category ==
    "-1") %>%
    group_by(Certainty) %>%
    summarize(mean_size = mean(Proportion, na.rm = TRUE))
```

**Table 2**

*Experiment 2b, Mean Proportion of Memory Responses by Certainty*

| | High Reward | | | | | |
|---|---|---|---|---|---|---|
| Measure | DO | LO | MO | MN | LN | DN |
| Conditioning | 0.418 | 0.187 | 0.155 | 0.131 | 0.117 | 0.104 |
| Post-conditioning | 0.332 | 0.172 | 0.171 | 0.171 | 0.139 | 0.130 |
| New | 0.050 | 0.075 | 0.139 | 0.245 | 0.300 | 0.264 |
| | Low Reward | | | | | |
| Measure | DO | LO | MO | MN | LN | DN |
| Conditioning | 0.423 | 0.200 | 0.129 | 0.158 | 0.112 | 0.109 |
| Post-conditioning | 0.288 | 0.202 | 0.163 | 0.182 | 0.140 | 0.136 |
| New | 0.048 | 0.084 | 0.109 | 0.255 | 0.291 | 0.271 |

*Note:* DO = definitely old; LO = likely old; MO = maybe old;
MN = maybe new; LN = likely new, DN = definitely new.

# 4. Supplementary

## 4.1 Performance on Matching Task

As part of control analyses, performance on the matching tasks were summarised and analysed for any biases between treatment groups. We first tested whether matching accuracy is above chance in each phase of encoding to ascertain participant's attention during encoding. Secondly, we tested whether there were significant differences in matching performance between items from different stimuli categories (animal vs. object) and reward categories (high vs. low).

### Experiment 2a

```
# Matching accuracy above chance in each phase
aggregate(MA ~ Phase, data.x2a, FUN = function(MA) c(mean = mean(MA),
    se = std.error(MA)))
```

```
##              Phase     MA.mean       MA.se
## 1 Pre-conditioning 0.867500000 0.010335656
## 2     Conditioning 0.909722222 0.008528488
```

```
# Phase 1 (pre-conditioning)
t.test(data = x2a_ph1, mu = 0.5, MA ~ Category, alternative = "two.sided")
```

```
##
##  Welch Two Sample t-test
##
## data:  MA by Category
## t = -23.902, df = 116.35, p-value < 2.2e-16
## alternative hypothesis: true difference in means is not equal to 0.5
## 95 percent confidence interval:
##  -0.03721894  0.04499672
## sample estimates:
## mean in group Animal mean in group Object
##            0.8694444            0.8655556
```

```r
# Phase 2 (conditioning)
t.test(data = x2a_ph2, mu = 0.5, MA ~ Category, alternative = "two.sided")
```

```
##
##  Welch Two Sample t-test
##
## data:  MA by Category
## t = -30.244, df = 115.87, p-value < 2.2e-16
## alternative hypothesis: true difference in means is not equal to 0.5
## 95 percent confidence interval:
##  -0.04991045  0.01768823
## sample estimates:
## mean in group Animal mean in group Object
##            0.9016667            0.9177778
```

Two sample t-tests showed that matching accuracy was well over chance level (0.5) in both phases, P values < .001, and thus suggested that participants were paying attention in both encoding phases.

Next we conducted paired t-tests and ANOVA to test whether matching accuracy in each phase varied by stimuli category (animal vs. objects), and whether matching accuracy for items from the conditioning phase varied with reward category (high vs. low reward).

```r
# Matching accuracy by categories (animal vs. objects)
aggregate(MA ~ Category + Phase, data.x2a, FUN = function(MA) c(mean = mean(MA),
    se = std.error(MA)))
```

```
##   Category            Phase    MA.mean      MA.se
## 1   Animal Pre-conditioning 0.86944444 0.01377543
## 2   Object Pre-conditioning 0.86555556 0.01552526
## 3   Animal     Conditioning 0.90166667 0.01285940
## 4   Object     Conditioning 0.91777778 0.01121794
```

```r
# Phase 1 (pre-conditioning)
t.test(data = x2a_ph1, MA ~ Category, paired = TRUE)
```

```
##
##  Paired t-test
##
## data:  MA by Category
## t = 0.35694, df = 59, p-value = 0.7224
## alternative hypothesis: true difference in means is not equal to 0
## 95 percent confidence interval:
##  -0.01791201  0.02568979
## sample estimates:
## mean of the differences
##             0.003888889
```

```r
cohens_dav(data = x2a_ph1, x = MA, group = Category)
```

```
## # A tibble: 2 x 4
##   Category count  mean    sd
```

```
##   <chr>    <int> <dbl> <dbl>
## 1 Animal      60 0.869 0.107
## 2 Object      60 0.866 0.120
## [1] "Effect size Cohen's d(av):"
## [1] 0.03426905
```

```
# Phase 2 (conditioning)
t.test(data = x2a_ph2, MA ~ Category, paired = TRUE)
```

```
##
##  Paired t-test
##
## data:  MA by Category
## t = -1.7534, df = 59, p-value = 0.08472
## alternative hypothesis: true difference in means is not equal to 0
## 95 percent confidence interval:
##  -0.034497053  0.002274831
## sample estimates:
## mean of the differences
##             -0.01611111
```

```
cohens_dav(data = x2a_ph2, x = MA, group = Category)
```

```
## # A tibble: 2 x 4
##   Category count  mean     sd
##   <chr>    <int> <dbl>  <dbl>
## 1 Animal      60 0.902 0.0996
## 2 Object      60 0.918 0.0869
## [1] "Effect size Cohen's d(av):"
## [1] -0.1727712
```

```
# Repeated measures ANOVA on memory by phase and category
anova_test(MA ~ Phase * Category + Error(UserID/(Phase * Category)),
    data = data.x2a)
```

```
## ANOVA Table (type III tests)
##
##           Effect DFn DFd     F     p p<.05      ges
## 1          Phase   1  59 8.735 0.004     * 0.040000
## 2       Category   1  59 1.048 0.310       0.000876
## 3 Phase:Category   1  59 1.516 0.223       0.002000
```

We find that in the conditioning phase (phase 2) of experiment 2a, at trend level, matching accuracy is higher for stimuli in the 'Object' category, t(59) = -1.75, p = .08, $d_{av}$ = -.02. The ANOVA also supported a non-significant main effect of category, F(1,59) = 1.05, p = .31, $\eta^2$ = .001. Since this effect is not significant, and the study design being equally counter-balanced when allocating Object/Animal as the highly rewarded category, this effect should not have interfered with memory effects.

Furthermore, we check how matching accuracy and reaction time in each phase varies by reward category (high vs. low).

```r
# Matching accuracy by reward category (high vs. low) Phase
# 2 (conditioning)
t.test(data = x2a_ph2, MA ~ Reward_Category, paired = TRUE)
```

```
##
##  Paired t-test
##
## data:  MA by Reward_Category
## t = -0.77015, df = 59, p-value = 0.4443
## alternative hypothesis: true difference in means is not equal to 0
## 95 percent confidence interval:
##  -0.02598704  0.01154260
## sample estimates:
## mean of the differences
##            -0.007222222
```

```r
cohens_dav(data = x2a_ph2, x = MA, group = Reward_Category)
```

```
## # A tibble: 2 x 4
##   Reward_Category count  mean     sd
##   <fct>           <int> <dbl>  <dbl>
## 1 High Reward        60 0.906 0.0985
## 2 Low Reward         60 0.913 0.0888
## [1] "Effect size Cohen's d(av):"
## [1] -0.07714126
```

Matching accuracy did not significantly differ between item reward categories (high vs. low), thus suggesting that equal attention was paid to all items, regardless of reward category, during encoding.

```r
# Repeated measures ANOVA on matching accuracy by phase and
# reward category
anova_test(MA ~ Phase * Reward_Category + Error(UserID/(Phase *
    Reward_Category)), data = data.x2a)
```

```
## ANOVA Table (type III tests)
##
##                  Effect DFn DFd         F     p p<.05      ges
## 1                 Phase   1  59 8.735e+00 0.004     * 4.00e-02
## 2       Reward_Category   1  59 8.500e-29 1.000       7.22e-32
## 3 Phase:Reward_Category   1  59 7.810e-01 0.380       1.00e-03
```

```r
# Repeated measures ANOVA on reaction time by phase and
# reward category
anova_test(RT ~ Phase * Reward_Category + Error(UserID/(Phase *
    Reward_Category)), data = data.x2a)
```

```
## ANOVA Table (type III tests)
##
##                  Effect DFn DFd      F        p p<.05      ges
## 1                 Phase   1  59 52.490 1.04e-09     * 7.60e-02
## 2       Reward_Category   1  59  0.224 6.38e-01       7.81e-05
## 3 Phase:Reward_Category   1  59  0.561 4.57e-01       2.31e-04
```

```r
# Graph: matching accuracy by phase and category
x2a.MA = plot_by_group_cat(data = data.x2a, yvar = "MA", ylim = c(0,
    1.2), ylab = "Matching Accuracy", subtitle = "Experiment 2a",
    lab.vjust = 2.5)
ggsave(file = "x2a.MA.svg", plot = x2a.MA, width = 10, height = 10,
    units = "cm")
x2a.MA
```

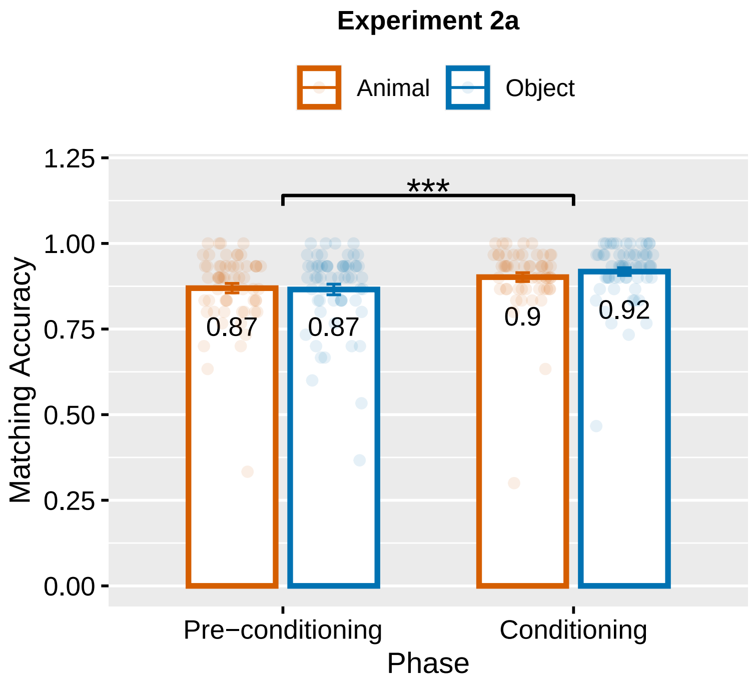


```r
# Graph: matching accuracy by phase and reward category
x2a.MA = plot_by_group(data = data.x2a, yvar = "MA", ylim = c(0,
    1.2), ylab = "Matching Accuracy", subtitle = "Experiment 2a",
    lab.vjust = 2.5)
ggsave(file = "x2a.MA.svg", plot = x2a.MA, width = 10, height = 10,
    units = "cm")
x2a.MA
```

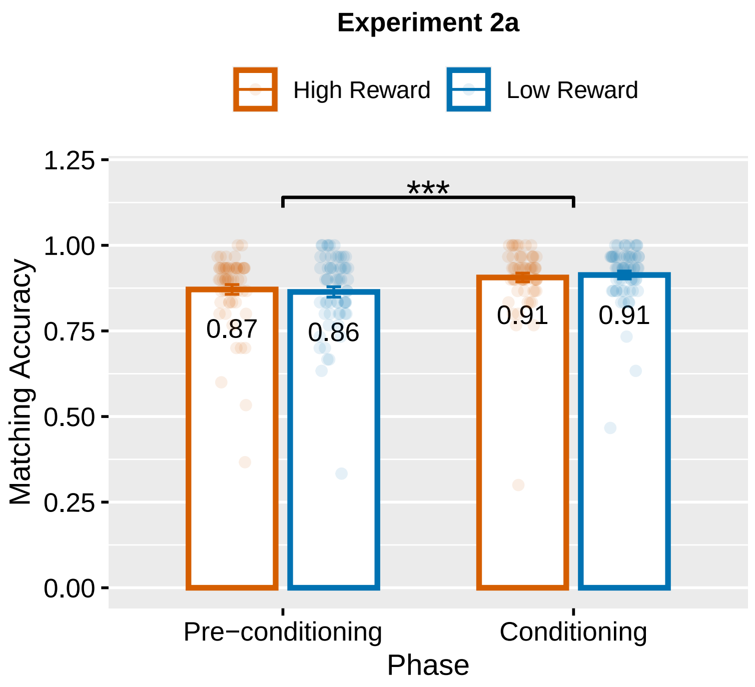


```
# Graph: matching reaction time by phase and reward
# category
x2a.RT = plot_by_group(data = data.x2a, yvar = "RT", ylim = c(0,
    600), ylab = "Reaction Time (ms)", subtitle = "Experiment 2a",
    lab.sf = 0, lab.vjust = 2.5)
ggsave(file = "x2a.RT.svg", plot = x2a.RT, width = 10, height = 10,
    units = "cm")
x2a.RT
```

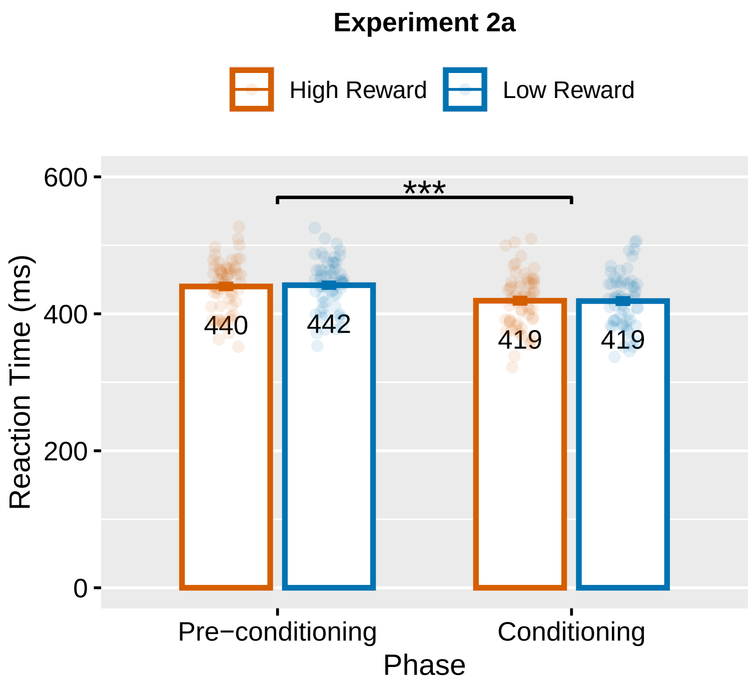


### Experiment 2b

```
# Matching accuracy above chance in each phase Phase 1
# (conditioning)
t.test(data = x2b_ph1, mu = 0.5, MA ~ Category, alternative = "two.sided")
```

```
##
##  Welch Two Sample t-test
##
## data:  MA by Category
## t = -33.658, df = 117.48, p-value < 2.2e-16
## alternative hypothesis: true difference in means is not equal to 0.5
## 95 percent confidence interval:
##  -0.03647794  0.02314460
## sample estimates:
## mean in group Animal mean in group Object
##            0.9105556            0.9172222
```

```
# Phase 2 (post-conditioning)
t.test(data = x2b_ph2, mu = 0.5, MA ~ Category, alternative = "two.sided")
```

```
##
##  Welch Two Sample t-test
##
## data:  MA by Category
## t = -22.654, df = 117.79, p-value < 2.2e-16
```

```
## alternative hypothesis: true difference in means is not equal to 0.5
## 95 percent confidence interval:
##  -0.02679213  0.05790325
## sample estimates:
## mean in group Animal mean in group Object
##            0.8666667            0.8511111
```

Two sample t-tests show that matching accuracy is well over chance level (0.5) in both phases, P values < .001, and thus suggesting that participants were paying attention in both encoding phases.

Next we check whether matching accuracy in each phase varied by stimuli category (animal vs. objects) and the reward category it was associated with (high vs. low reward)

```
# Matching accuracy by categories (animal vs. objects)
aggregate(MA ~ Category + Phase, data.x2b, FUN = function(MA) c(mean = mean(MA),
    se = std.error(MA)))
```

```
##   Category            Phase    MA.mean      MA.se
## 1   Animal      Conditioning 0.91055556 0.01028446
## 2   Object      Conditioning 0.91722222 0.01099254
## 3   Animal Post-conditioning 0.86666667 0.01480147
## 4   Object Post-conditioning 0.85111111 0.01543402
```

```
# Phase 1 (conditioning)
t.test(data = x2b_ph1, MA ~ Category, paired = TRUE)
```

```
##
##  Paired t-test
##
## data:  MA by Category
## t = -0.73572, df = 59, p-value = 0.4648
## alternative hypothesis: true difference in means is not equal to 0
## 95 percent confidence interval:
##  -0.02479851  0.01146518
## sample estimates:
## mean of the differences
##            -0.006666667
```

```
cohens_dav(data = x2b_ph1, x = MA, group = Category)
```

```
## # A tibble: 2 x 4
##   Category count  mean     sd
##   <chr>    <int> <dbl>  <dbl>
## 1 Animal      60 0.911 0.0797
## 2 Object      60 0.917 0.0851
## [1] "Effect size Cohen's d(av):"
## [1] -0.08090079
```

```
# Phase 2 (post-conditioning)
t.test(data = x2b_ph2, MA ~ Category, paired = TRUE)
```

```
##
##  Paired t-test
##
## data:  MA by Category
## t = 1.7458, df = 59, p-value = 0.08604
## alternative hypothesis: true difference in means is not equal to 0
## 95 percent confidence interval:
##  -0.002273414  0.033384525
## sample estimates:
## mean of the differences
##              0.01555556
```

```
cohens_dav(data = x2b_ph2, x = MA, group = Category)
```

```
## # A tibble: 2 x 4
##   Category count  mean    sd
##   <chr>    <int> <dbl> <dbl>
## 1 Animal      60 0.867 0.115
## 2 Object      60 0.851 0.120
## [1] "Effect size Cohen's d(av):"
## [1] 0.1328382
```

```
# Repeated measures ANOVA on matching accuracy by phase and
# category
anova_test(MA ~ Phase * Category + Error(UserID/(Phase * Category)),
    data = data.x2b)
```

```
## ANOVA Table (type III tests)
##
##           Effect DFn DFd      F        p p<.05      ges
## 1          Phase   1  59 20.072 3.48e-05     * 0.070000
## 2       Category   1  59  0.570 4.53e-01       0.000489
## 3 Phase:Category   1  59  2.679 1.07e-01       0.003000
```

Furthermore, we check how matching accuracy varies by reward category (high vs. low).

```
# Matching accuracy by reward category (high vs. low) Phase
# 1 (conditioning)
t.test(data = x2b_ph1, MA ~ Reward_Category, paired = TRUE)
```

```
##
##  Paired t-test
##
## data:  MA by Reward_Category
## t = 0.12208, df = 59, p-value = 0.9033
## alternative hypothesis: true difference in means is not equal to 0
## 95 percent confidence interval:
##  -0.01710142  0.01932364
## sample estimates:
## mean of the differences
##             0.001111111
```

```r
cohens_dav(data = x2b_ph1, x = MA, group = Reward_Category)
```

```
## # A tibble: 2 x 4
##   Reward_Category count  mean     sd
##   <fct>           <int> <dbl>  <dbl>
## 1 High Reward        60 0.914 0.0787
## 2 Low Reward         60 0.913 0.0862
## [1] "Effect size Cohen's d(av):"
## [1] 0.01347906
```

```r
# Phase 2 (post-conditioning)
t.test(data = x2b_ph2, MA ~ Reward_Category, paired = TRUE)
```

```
##
##  Paired t-test
##
## data:  MA by Reward_Category
## t = 0.73292, df = 59, p-value = 0.4665
## alternative hypothesis: true difference in means is not equal to 0
## 95 percent confidence interval:
##  -0.01153436  0.02486769
## sample estimates:
## mean of the differences
##             0.006666667
```

```r
cohens_dav(data = x2b_ph2, x = MA, group = Reward_Category)
```

```
## # A tibble: 2 x 4
##   Reward_Category count  mean    sd
##   <fct>           <int> <dbl> <dbl>
## 1 High Reward        60 0.862 0.116
## 2 Low Reward         60 0.856 0.119
## [1] "Effect size Cohen's d(av):"
## [1] 0.05682086
```

```r
# Repeated measures ANOVA on matching accuracy by phase and
# reward category
anova_test(MA ~ Phase * Reward_Category + Error(UserID/(Phase *
    Reward_Category)), data = data.x2b)
```

```
## ANOVA Table (type III tests)
##
##                  Effect DFn DFd      F        p p<.05      ges
## 1                 Phase   1  59 20.072 3.48e-05     * 0.070000
## 2       Reward_Category   1  59  0.435 5.12e-01       0.000374
## 3 Phase:Reward_Category   1  59  0.161 6.90e-01       0.000191
```

```r
# Repeated measures ANOVA on reaction time by phase and
# reward category
anova_test(RT ~ Phase * Reward_Category + Error(UserID/(Phase *
    Reward_Category)), data = data.x2b)
```

```
## ANOVA Table (type III tests)
##
##                   Effect DFn DFd      F        p p<.05      ges
## 1                  Phase   1  59 18.769 5.82e-05     * 0.030000
## 2        Reward_Category   1  59  0.880 3.52e-01       0.000536
## 3 Phase:Reward_Category   1  59  0.469 4.96e-01       0.000208
```

```
# Graph: matching accuracy by phase and category
x2b.MA = plot_by_group_cat(data = data.x2b, yvar = "MA", ylim = c(0,
    1.2), ylab = "Matching Accuracy", subtitle = "Experiment 2b",
    lab.vjust = 2.5)
ggsave(file = "x2a.MA.svg", plot = x2b.MA, width = 10, height = 10,
    units = "cm")
x2b.MA
```

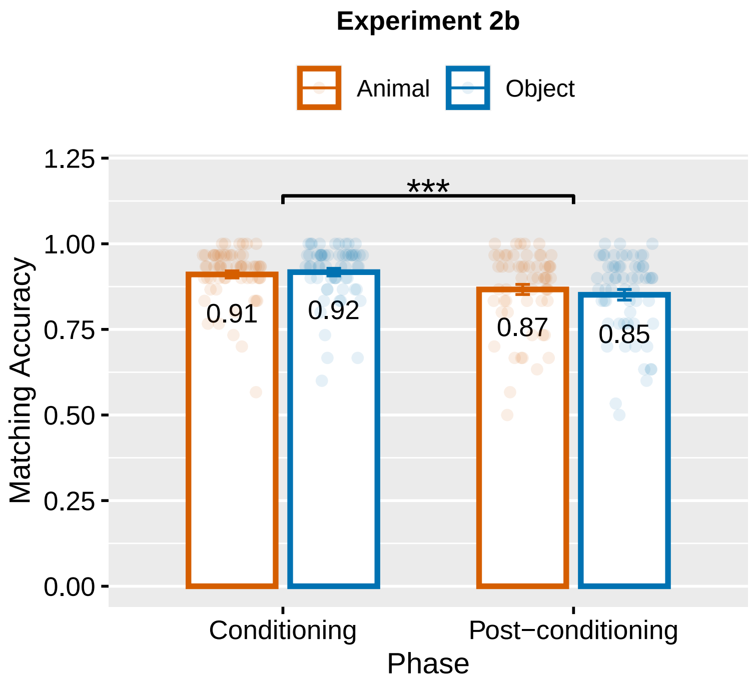


```
# Graph: matching accuracy by phase and reward category
x2b.MA = plot_by_group(data = data.x2b, "2b", yvar = "MA", ylim = c(0,
    1.2), ylab = "Matching Accuracy", subtitle = "Experiment 2b",
    lab.vjust = 2.5)
x2b.MA
```

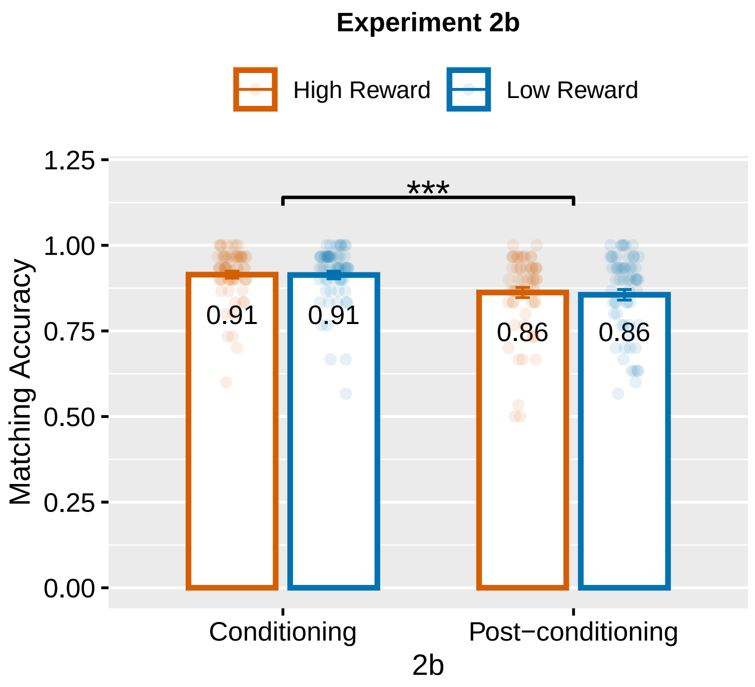


```
ggsave(file = "x2b.MA.svg", plot = x2b.MA, width = 10, height = 10,
    units = "cm")
```

```
# Graph: matching reaction time by phase and reward
# category
x2b.RT = plot_by_group(data = data.x2b, yvar = "RT", ylim = c(0,
    600), ylab = "Reaction Time (ms)", subtitle = "Experiment 2b",
    lab.sf = 0, lab.vjust = 2.5)
ggsave(file = "x2b.RT.svg", plot = x2b.RT, width = 10, height = 10,
    units = "cm")
x2b.RT
```

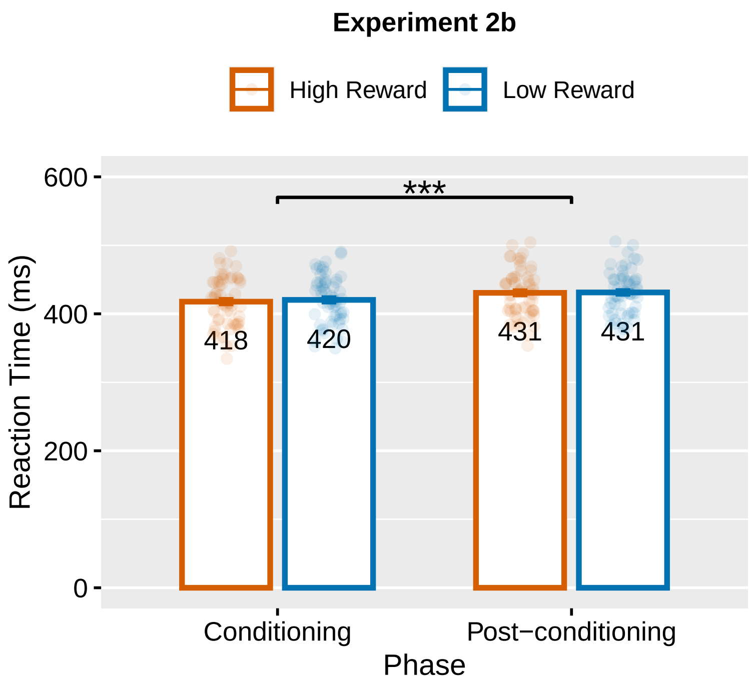


## 4.2 Exclusion Based on Surprisal

One way in which our study differed from the original study is that participants who were not surprised by the memory test were excluded from all analysis. Here we show that this does not significantly change the pattern of our main results by repeating the analysis after excluding participants who were not surprised. After exclusions, experiment 2a had N=48 and experiment 2b had N=44 participants.

The ANOVAs revealed significant effects and trends of encoding phase, but no interaction effects between the encoding phase and reward category that the item belonged to. Furthermore, as found in the analysis prior to exclusions, t-tests revealed a significant effect of reward category in the pre-conditioning phase when considering all memory trials in experiment 2a, $p < .001$, which did not hold when considering high certainty memory trials. As in the main analysis, this reward category effect on memory was in favor of the low reward category. There were no further effects of reward category on memory, both corrected recognition and d-primes, in other phases of each experiment.

```
# Participants to exclude as they anticipated the memory
# test
data.x2a.exclude <- read.csv("adaptiveMemoryReplication/Exp2a_CleanData/Supp/x2a_Surprisal_Exclude.csv"
data.x2b.exclude <- read.csv("adaptiveMemoryReplication/Exp2b_CleanData/Supp/x2b_Surprisal_Exclude.csv"
x2a.exclude <- subset(data.x2a.exclude, Exclude.Surprise == 1,
    UserID)
x2b.exclude <- subset(data.x2b.exclude, Exclude.Surprise == 1,
    UserID)

# Data subset (not surprised)
data.x2a.ns <- data.x2a[!data.x2a$UserID %in% x2a.exclude$UserID,
    ]
data.x2b.ns <- data.x2b[!data.x2b$UserID %in% x2b.exclude$UserID,
```

```
    ]
data.x2a.high.ns <- data.x2a.high[!data.x2a.high$UserID %in%
    x2a.exclude$UserID, ]
data.x2b.high.ns <- data.x2b.high[!data.x2b.high$UserID %in%
    x2b.exclude$UserID, ]

# Change phase labels for readability
data.x2a.ns$Phase[data.x2a.ns$Phase == "Ph1"] <- "Pre-conditioning"
data.x2a.ns$Phase[data.x2a.ns$Phase == "Ph2"] <- "Conditioning"
data.x2a.high.ns$Phase[data.x2a.high.ns$Phase == "Ph1"] <- "Pre-conditioning"
data.x2a.high.ns$Phase[data.x2a.high.ns$Phase == "Ph2"] <- "Conditioning"
# Reorder variables
data.x2a.ns$Reward_Category <- factor(data.x2a.ns$Reward_Category,
    levels = c("High Reward", "Low Reward"))
data.x2a.ns$Phase <- factor(data.x2a.ns$Phase, levels = c("Pre-conditioning",
    "Conditioning"))
data.x2a.high.ns$Reward_Category <- factor(data.x2a.high.ns$Reward_Category,
    levels = c("High Reward", "Low Reward"))
data.x2a.high.ns$Phase <- factor(data.x2a.high.ns$Phase, levels = c("Pre-conditioning",
    "Conditioning"))

# Change phase labels for readability
data.x2b.ns$Phase[data.x2b.ns$Phase == "Ph1"] <- "Conditioning"
data.x2b.ns$Phase[data.x2b.ns$Phase == "Ph2"] <- "Post-conditioning"
data.x2b.high.ns$Phase[data.x2b.high.ns$Phase == "Ph1"] <- "Conditioning"
data.x2b.high.ns$Phase[data.x2b.high.ns$Phase == "Ph2"] <- "Post-conditioning"
# Reorder variables
data.x2b.ns$Reward_Category <- factor(data.x2b.ns$Reward_Category,
    levels = c("High Reward", "Low Reward"))
data.x2b.ns$Phase <- factor(data.x2b.ns$Phase, levels = c("Conditioning",
    "Post-conditioning"))
data.x2b.high.ns$Reward_Category <- factor(data.x2b.high.ns$Reward_Category,
    levels = c("High Reward", "Low Reward"))
data.x2b.high.ns$Phase <- factor(data.x2b.high.ns$Phase, levels = c("Conditioning",
    "Post-conditioning"))
```

### Experiment 2a (All Memory)

```
# Repeated measures two-factor ANOVA on corrected
# recognition (all memory) Corrected recognition (CR)
anova_test(CR ~ Phase * Reward_Category + Error(UserID/(Phase *
    Reward_Category)), data = data.x2a.ns)
```

```
## ANOVA Table (type III tests)
##
##                  Effect DFn DFd     F     p p<.05   ges
## 1                 Phase   1  47 6.890 0.012     * 0.008
## 2       Reward_Category   1  47 6.196 0.016     * 0.011
## 3 Phase:Reward_Category   1  47 2.259 0.140       0.002
```

```r
# d-prime (DP)
anova_test(DP ~ Phase * Reward_Category + Error(UserID/(Phase *
    Reward_Category)), data = data.x2a.ns)
```

```
## ANOVA Table (type III tests)
##
##                    Effect DFn DFd     F     p p<.05   ges
## 1                   Phase   1  47 6.132 0.017     * 0.007
## 2         Reward_Category   1  47 5.499 0.023     * 0.009
## 3 Phase:Reward_Category   1  47 3.161 0.082       0.003
```

```r
# Create subsets for each phase from data.x2a
x2a_ns_ph1 <- subset(data.x2a.ns, Phase == "Pre-conditioning")
x2a_ns_ph2 <- subset(data.x2a.ns, Phase == "Conditioning")
```

```r
# Effect of reward category on high certainty memory in
# phase 1 (pre-conditioning) Corrected recognition (CR)
t.test(data = x2a_ns_ph1, CR ~ Reward_Category, paired = TRUE)
```

```
##
##  Paired t-test
##
## data:  CR by Reward_Category
## t = -2.8322, df = 47, p-value = 0.006786
## alternative hypothesis: true difference in means is not equal to 0
## 95 percent confidence interval:
##  -0.10312632 -0.01746756
## sample estimates:
## mean of the differences
##             -0.06029694
```

```r
cohens_dav(data = x2a_ns_ph1, x = CR, group = Reward_Category)
```

```
## # A tibble: 2 x 4
##   Reward_Category count  mean    sd
##   <fct>           <int> <dbl> <dbl>
## 1 High Reward        48 0.458 0.209
## 2 Low Reward         48 0.518 0.218
## [1] "Effect size Cohen's d(av):"
## [1] -0.2828026
```

```r
# d-prime (DP)
t.test(data = x2a_ns_ph1, DP ~ Reward_Category, paired = TRUE)
```

```
##
##  Paired t-test
##
## data:  DP by Reward_Category
## t = -2.7974, df = 47, p-value = 0.007443
## alternative hypothesis: true difference in means is not equal to 0
```

```
## 95 percent confidence interval:
##  -0.40239246 -0.06574023
## sample estimates:
## mean of the differences
##              -0.2340663
```

```r
cohens_dav(data = x2a_ns_ph1, x = DP, group = Reward_Category)
```

```
## # A tibble: 2 x 4
##   Reward_Category count  mean    sd
##   <fct>           <int> <dbl> <dbl>
## 1 High Reward        48  2.02 0.765
## 2 Low Reward         48  2.25 0.881
## [1] "Effect size Cohen's d(av):"
## [1] -0.2843568
```

```r
# Effect of reward category on high certainty memory in
# phase 2 (conditioning) Corrected recognition (CR)
t.test(data = x2a_ns_ph2, CR ~ Reward_Category, paired = TRUE)
```

```
##
##  Paired t-test
##
## data:  CR by Reward_Category
## t = -1.3079, df = 47, p-value = 0.1973
## alternative hypothesis: true difference in means is not equal to 0
## 95 percent confidence interval:
##  -0.06701905  0.01421047
## sample estimates:
## mean of the differences
##             -0.02640429
```

```r
cohens_dav(data = x2a_ns_ph2, x = CR, group = Reward_Category)
```

```
## # A tibble: 2 x 4
##   Reward_Category count  mean    sd
##   <fct>           <int> <dbl> <dbl>
## 1 High Reward        48 0.512 0.206
## 2 Low Reward         48 0.538 0.203
## [1] "Effect size Cohen's d(av):"
## [1] -0.129124
```

```r
# d-prime (DP)
t.test(data = x2a_ns_ph2, DP ~ Reward_Category, paired = TRUE)
```

```
##
##  Paired t-test
##
## data:  DP by Reward_Category
## t = -0.86395, df = 47, p-value = 0.392
## alternative hypothesis: true difference in means is not equal to 0
```

```
## 95 percent confidence interval:
##  -0.21621584  0.08629975
## sample estimates:
## mean of the differences
##             -0.06495804
```

```
cohens_dav(data = x2a_ns_ph2, x = DP, group = Reward_Category)
```

```
## # A tibble: 2 x 4
##   Reward_Category count  mean    sd
##   <fct>           <int> <dbl> <dbl>
## 1 High Reward        48  2.24 0.750
## 2 Low Reward         48  2.30 0.817
## [1] "Effect size Cohen's d(av):"
## [1] -0.08291897
```

### Experiment 2a (High Certainty Memory)

```
# Repeated measures two-factor ANOVA on corrected
# recognition (high certainty only) Corrected recognition
# (CR)
anova_test(CR ~ Phase * Reward_Category + Error(UserID/(Phase *
    Reward_Category)), data = data.x2a.high.ns)
```

```
## ANOVA Table (type III tests)
##
##                  Effect DFn DFd     F     p p<.05      ges
## 1                 Phase   1  47 3.422 0.071       0.004000
## 2       Reward_Category   1  47 0.248 0.620       0.000672
## 3 Phase:Reward_Category   1  47 2.028 0.161       0.002000
```

```
# d-prime (DP)
anova_test(DP ~ Phase * Reward_Category + Error(UserID/(Phase *
    Reward_Category)), data = data.x2a.high.ns)
```

```
## ANOVA Table (type III tests)
##
##                  Effect DFn DFd     F     p p<.05      ges
## 1                 Phase   1  47 3.100 0.085       4.00e-03
## 2       Reward_Category   1  47 0.032 0.858       6.03e-05
## 3 Phase:Reward_Category   1  47 2.335 0.133       2.00e-03
```

```
# Create subsets for each phase from data.x2a
x2a_high_ns_ph1 <- subset(data.x2a.high.ns, Phase == "Pre-conditioning")
x2a_high_ns_ph2 <- subset(data.x2a.high.ns, Phase == "Conditioning")
```

```
# Effect of reward category on high certainty memory in
# phase 1 (pre-conditioning) Corrected recognition (CR)
t.test(data = x2a_high_ns_ph1, CR ~ Reward_Category, paired = TRUE)
```

```
##
##  Paired t-test
##
## data:  CR by Reward_Category
## t = -1.1001, df = 47, p-value = 0.2769
## alternative hypothesis: true difference in means is not equal to 0
## 95 percent confidence interval:
##  -0.09116143  0.02670744
## sample estimates:
## mean of the differences
##             -0.03222699
```

```
cohens_dav(data = x2a_high_ns_ph1, x = CR, group = Reward_Category)
```

```
## # A tibble: 2 x 4
##   Reward_Category count  mean    sd
##   <fct>           <int> <dbl> <dbl>
## 1 High Reward        48 0.557 0.241
## 2 Low Reward         48 0.589 0.230
## [1] "Effect size Cohen's d(av):"
## [1] -0.1367958
```

```
# d-prime (DP)
t.test(data = x2a_high_ns_ph1, DP ~ Reward_Category, paired = TRUE)
```

```
##
##  Paired t-test
##
## data:  DP by Reward_Category
## t = -0.7507, df = 47, p-value = 0.4566
## alternative hypothesis: true difference in means is not equal to 0
## 95 percent confidence interval:
##  -0.3112893  0.1421014
## sample estimates:
## mean of the differences
##             -0.08459394
```

```
cohens_dav(data = x2a_high_ns_ph1, x = DP, group = Reward_Category)
```

```
## # A tibble: 2 x 4
##   Reward_Category count  mean    sd
##   <fct>           <int> <dbl> <dbl>
## 1 High Reward        48  2.57  1.01
## 2 Low Reward         48  2.65  1.06
## [1] "Effect size Cohen's d(av):"
## [1] -0.08137582
```

```
# Effect of reward category on high certainty memory in
# phase 2 (conditioning) Corrected recognition (CR)
t.test(data = x2a_high_ns_ph2, CR ~ Reward_Category, paired = TRUE)
```

```
##
##  Paired t-test
##
## data:  CR by Reward_Category
## t = 0.32939, df = 47, p-value = 0.7433
## alternative hypothesis: true difference in means is not equal to 0
## 95 percent confidence interval:
##  -0.04380491  0.06095790
## sample estimates:
## mean of the differences
##             0.008576495
```

```
cohens_dav(data = x2a_high_ns_ph2, x = CR, group = Reward_Category)
```

```
## # A tibble: 2 x 4
##   Reward_Category count  mean    sd
##   <fct>           <int> <dbl> <dbl>
## 1 High Reward        48 0.607 0.216
## 2 Low Reward         48 0.598 0.233
## [1] "Effect size Cohen's d(av):"
## [1] 0.03815482
```

```
# d-prime (DP)
t.test(data = x2a_high_ns_ph2, DP ~ Reward_Category, paired = TRUE)
```

```
##
##  Paired t-test
##
## data:  DP by Reward_Category
## t = 1.1052, df = 47, p-value = 0.2747
## alternative hypothesis: true difference in means is not equal to 0
## 95 percent confidence interval:
##  -0.09506624  0.32685111
## sample estimates:
## mean of the differences
##               0.1158924
```

```
cohens_dav(data = x2a_high_ns_ph2, x = DP, group = Reward_Category)
```

```
## # A tibble: 2 x 4
##   Reward_Category count  mean    sd
##   <fct>           <int> <dbl> <dbl>
## 1 High Reward        48  2.80 0.929
## 2 Low Reward         48  2.68 1.06
## [1] "Effect size Cohen's d(av):"
## [1] 0.1165282
```

### Experiment 2b (All Memory)

```r
# Repeated measures two-factor ANOVA on corrected
# recognition (all memory) Corrected recognition (CR)
anova_test(CR ~ Phase * Reward_Category + Error(UserID/(Phase *
    Reward_Category)), data = data.x2b.ns)
```

```
## ANOVA Table (type III tests)
##
##                  Effect DFn DFd      F        p p<.05      ges
## 1                 Phase   1  43 27.926 3.97e-06     * 0.042000
## 2       Reward_Category   1  43  0.874 3.55e-01       0.003000
## 3 Phase:Reward_Category   1  43  0.208 6.50e-01       0.000164
```

```r
# d-prime (DP)
anova_test(DP ~ Phase * Reward_Category + Error(UserID/(Phase *
    Reward_Category)), data = data.x2b.ns)
```

```
## ANOVA Table (type III tests)
##
##                  Effect DFn DFd      F        p p<.05      ges
## 1                 Phase   1  43 26.528 6.17e-06     * 0.036000
## 2       Reward_Category   1  43  0.834 3.66e-01       0.003000
## 3 Phase:Reward_Category   1  43  0.327 5.70e-01       0.000287
```

```r
# Create subsets for each phase from data.x2a
x2b_ns_ph1 <- subset(data.x2b.ns, Phase == "Conditioning")
x2b_ns_ph2 <- subset(data.x2b.ns, Phase == "Post-conditioning")
```

```r
# Effect of reward category on high certainty memory in
# phase 1 (conditioning) Corrected recognition (CR)
t.test(data = x2b_ns_ph1, CR ~ Reward_Category, paired = TRUE)
```

```
##
##  Paired t-test
##
## data:  CR by Reward_Category
## t = 1.0253, df = 43, p-value = 0.311
## alternative hypothesis: true difference in means is not equal to 0
## 95 percent confidence interval:
##  -0.02556558  0.07844510
## sample estimates:
## mean of the differences
##              0.02643976
```

```r
cohens_dav(data = x2b_ns_ph1, x = CR, group = Reward_Category)
```

```
## # A tibble: 2 x 4
##   Reward_Category count  mean    sd
##   <fct>           <int> <dbl> <dbl>
## 1 High Reward        44 0.523 0.181
## 2 Low Reward         44 0.496 0.228
## [1] "Effect size Cohen's d(av):"
## [1] 0.129187
```

```r
# d-prime (DP)
t.test(data = x2b_ns_ph1, DP ~ Reward_Category, paired = TRUE)
```

```
##
##  Paired t-test
##
## data:  DP by Reward_Category
## t = 1.028, df = 43, p-value = 0.3097
## alternative hypothesis: true difference in means is not equal to 0
## 95 percent confidence interval:
##  -0.1016037  0.3128852
## sample estimates:
## mean of the differences
##               0.1056407
```

```r
cohens_dav(data = x2b_ns_ph1, x = DP, group = Reward_Category)
```

```
## # A tibble: 2 x 4
##   Reward_Category count  mean    sd
##   <fct>           <int> <dbl> <dbl>
## 1 High Reward        44  2.26 0.687
## 2 Low Reward         44  2.15 0.863
## [1] "Effect size Cohen's d(av):"
## [1] 0.1362979
```

```r
# Effect of reward category on high certainty memory in
# phase 2 (post-conditioning) Corrected recognition (CR)
t.test(data = x2b_ns_ph2, CR ~ Reward_Category, paired = TRUE)
```

```
##
##  Paired t-test
##
## data:  CR by Reward_Category
## t = 0.64099, df = 43, p-value = 0.5249
## alternative hypothesis: true difference in means is not equal to 0
## 95 percent confidence interval:
##  -0.03445533  0.06656310
## sample estimates:
## mean of the differences
##              0.01605388
```

```r
cohens_dav(data = x2b_ns_ph2, x = CR, group = Reward_Category)
```

```
## # A tibble: 2 x 4
##   Reward_Category count  mean    sd
##   <fct>           <int> <dbl> <dbl>
## 1 High Reward        44 0.432 0.192
## 2 Low Reward         44 0.416 0.217
## [1] "Effect size Cohen's d(av):"
## [1] 0.07861152
```

```r
# d-prime (DP)
t.test(data = x2b_ns_ph2, DP ~ Reward_Category, paired = TRUE)
```

```
##
##  Paired t-test
##
## data:  DP by Reward_Category
## t = 0.58006, df = 43, p-value = 0.5649
## alternative hypothesis: true difference in means is not equal to 0
## 95 percent confidence interval:
##  -0.1348374  0.2437233
## sample estimates:
## mean of the differences
##              0.05444293
```

```r
cohens_dav(data = x2b_ns_ph2, x = DP, group = Reward_Category)
```

```
## # A tibble: 2 x 4
##   Reward_Category count  mean    sd
##   <fct>           <int> <dbl> <dbl>
## 1 High Reward        44  1.94 0.667
## 2 Low Reward         44  1.89 0.818
## [1] "Effect size Cohen's d(av):"
## [1] 0.07328106
```

### Experiment 2b (High Certainty Memory)

```r
# Repeated measures two-factor ANOVA on corrected
# recognition (high certainty only) Corrected recognition
# (CR)
anova_test(CR ~ Phase * Reward_Category + Error(UserID/(Phase *
    Reward_Category)), data = data.x2b.high.ns)
```

```
## ANOVA Table (type III tests)
##
##                  Effect DFn DFd      F        p p<.05      ges
## 1                 Phase   1  43 26.715 5.82e-06     * 0.041000
## 2       Reward_Category   1  43  1.144 2.91e-01       0.004000
## 3 Phase:Reward_Category   1  43  0.181 6.73e-01       0.000183
```

```r
# d-prime (DP)
anova_test(DP ~ Phase * Reward_Category + Error(UserID/(Phase *
    Reward_Category)), data = data.x2b.high.ns)
```

```
## ANOVA Table (type III tests)
##
##                  Effect DFn DFd      F        p p<.05      ges
## 1                 Phase   1  43 31.733 1.25e-06     * 4.50e-02
## 2       Reward_Category   1  43  1.037 3.14e-01       4.00e-03
## 3 Phase:Reward_Category   1  43  0.014 9.08e-01       1.42e-05
```

```r
# Create subsets for each phase from data.x2a
x2b_high_ns_ph1 <- subset(data.x2b.high.ns, Phase == "Conditioning")
x2b_high_ns_ph2 <- subset(data.x2b.high.ns, Phase == "Post-conditioning")
```

```r
# Effect of reward category on high certainty memory in
# phase 1 (conditioning) Corrected recognition (CR)
t.test(data = x2b_high_ns_ph1, CR ~ Reward_Category, paired = TRUE)
```

```
##
##  Paired t-test
##
## data:  CR by Reward_Category
## t = 0.74008, df = 43, p-value = 0.4633
## alternative hypothesis: true difference in means is not equal to 0
## 95 percent confidence interval:
##  -0.03586795  0.07745505
## sample estimates:
## mean of the differences
##              0.02079355
```

```r
cohens_dav(data = x2b_high_ns_ph1, x = CR, group = Reward_Category)
```

```
## # A tibble: 2 x 4
##   Reward_Category count  mean    sd
##   <fct>           <int> <dbl> <dbl>
## 1 High Reward        44 0.646 0.187
## 2 Low Reward         44 0.625 0.230
## [1] "Effect size Cohen's d(av):"
## [1] 0.09977119
```

```r
# d-prime (DP)
t.test(data = x2b_high_ns_ph1, DP ~ Reward_Category, paired = TRUE)
```

```
##
##  Paired t-test
##
## data:  DP by Reward_Category
## t = 0.96243, df = 43, p-value = 0.3412
## alternative hypothesis: true difference in means is not equal to 0
## 95 percent confidence interval:
##  -0.1293859  0.3656153
## sample estimates:
## mean of the differences
##               0.1181147
```

```r
cohens_dav(data = x2b_high_ns_ph1, x = DP, group = Reward_Category)
```

```
## # A tibble: 2 x 4
##   Reward_Category count  mean    sd
##   <fct>           <int> <dbl> <dbl>
```

```
## 1 High Reward        44  2.90 0.918
## 2 Low Reward         44  2.78 0.976
## [1] "Effect size Cohen's d(av):"
## [1] 0.1247098
```

```
# Effect of reward category on high certainty memory in
# phase 2 (post-conditioning) Corrected recognition (CR)
t.test(data = x2b_high_ns_ph2, CR ~ Reward_Category, paired = TRUE)
```

```
##
##  Paired t-test
##
## data:  CR by Reward_Category
## t = 1.1404, df = 43, p-value = 0.2604
## alternative hypothesis: true difference in means is not equal to 0
## 95 percent confidence interval:
##  -0.02472758  0.08909083
## sample estimates:
## mean of the differences
##              0.03218162
```

```
cohens_dav(data = x2b_high_ns_ph2, x = CR, group = Reward_Category)
```

```
## # A tibble: 2 x 4
##   Reward_Category count  mean    sd
##   <fct>           <int> <dbl> <dbl>
## 1 High Reward        44 0.564 0.198
## 2 Low Reward         44 0.532 0.234
## [1] "Effect size Cohen's d(av):"
## [1] 0.1490164
```

```
# d-prime (DP)
t.test(data = x2b_high_ns_ph2, DP ~ Reward_Category, paired = TRUE)
```

```
##
##  Paired t-test
##
## data:  DP by Reward_Category
## t = 0.83552, df = 43, p-value = 0.408
## alternative hypothesis: true difference in means is not equal to 0
## 95 percent confidence interval:
##  -0.1477032  0.3566652
## sample estimates:
## mean of the differences
##                0.104481
```

```
cohens_dav(data = x2b_high_ns_ph2, x = DP, group = Reward_Category)
```

```
## # A tibble: 2 x 4
##   Reward_Category count  mean    sd
##   <fct>           <int> <dbl> <dbl>
```

```
## 1 High Reward        44  2.50 0.780
## 2 Low Reward         44  2.40 0.974
## [1] "Effect size Cohen's d(av):"
## [1] 0.1191573
```

### Summary Graphs

```
x2a.CR.ns = plot_by_group(data = data.x2a.ns, yvar = "CR", ylim = c(0,
    1.2), ylab = "Corrected Recognition", xlab = NULL, subtitle = "All Memory",
    tag = "A")
x2a.high.CR.ns = plot_by_group(data = data.x2a.high.ns, yvar = "CR",
    ylim = c(0, 1.2), ylab = "Corrected Recognition", xlab = NULL,
    subtitle = "High Certainty Memory", tag = "B")
x2a.DP.ns = plot_by_group(data = data.x2a.ns, yvar = "DP", ylim = c(0,
    4.6), ylab = "d-prime", xlab = NULL, subtitle = "All Memory",
    tag = "C")
x2a.high.DP.ns = plot_by_group(data = data.x2a.high.ns, yvar = "DP",
    ylim = c(0, 4.6), ylab = "d-prime", xlab = NULL, subtitle = "High Certainty Memory",
    tag = "D")

summary.x2a.ns <- ggarrange(x2a.CR.ns, x2a.high.CR.ns, x2a.DP.ns,
    x2a.high.DP.ns, ncol = 2, nrow = 2, common.legend = TRUE,
    legend = "top")
# summary.x2a <- annotate_figure(summary.x2a, top =
# text_grob('Experiment 2a', face = 'bold', size = 12))
ggsave(file = "summary.x2a.ns.svg", plot = summary.x2a.ns, width = 8,
    height = 6.5)
ggsave(file = "summary.x2a.ns.jpg", plot = summary.x2a.ns, width = 8,
    height = 6.5)
```

```
x2b.CR.ns = plot_by_group(data = data.x2b.ns, yvar = "CR", ylim = c(0,
    1.2), ylab = "Corrected Recognition", xlab = NULL, subtitle = "All Memory",
    tag = "A")
x2b.high.CR.ns = plot_by_group(data = data.x2b.high.ns, yvar = "CR",
    ylim = c(0, 1.2), ylab = "Corrected Recognition", xlab = NULL,
    subtitle = "High Certainty Memory", tag = "B")
x2b.DP.ns = plot_by_group(data = data.x2b.ns, yvar = "DP", ylim = c(0,
    4.6), ylab = "d-prime", xlab = NULL, subtitle = "All Memory",
    tag = "C")
x2b.high.DP.ns = plot_by_group(data = data.x2b.high.ns, yvar = "DP",
    ylim = c(0, 4.6), ylab = "d-prime", xlab = NULL, subtitle = "High Certainty Memory",
    tag = "D")

summary.x2b.ns <- ggarrange(x2b.CR.ns, x2b.high.CR.ns, x2b.DP.ns,
    x2b.high.DP.ns, ncol = 2, nrow = 2, common.legend = TRUE,
    legend = "top")
# summary.x2b <- annotate_figure(summary.x2b, top =
# text_grob('Experiment 2b', face = 'bold', size = 12))
ggsave(file = "summary.x2b.ns.svg", plot = summary.x2b.ns, width = 8,
    height = 6.5)
ggsave(file = "summary.x2b.ns.jpg", plot = summary.x2b.ns, width = 8,
    height = 6.5)
```

## 4.3 Comparison of Response Biases

Response bias, calculated as per signal detection theory, was calculated for trials in each phase and reward category. We used paired t-tests to check if there were significant differences between response biases for items in high vs. low reward categories in any phases which could have influenced our results. The analysis below did not reveal any such effects.

### Experiment 2a (All Memory)

```
# Effect of reward category on response bias in phase 1
# (pre-conditioning)
t.test(data = x2a_ph1, RB ~ Reward_Category, paired = TRUE)
```

```
##
##  Paired t-test
##
## data:  RB by Reward_Category
## t = 0.96465, df = 59, p-value = 0.3387
## alternative hypothesis: true difference in means is not equal to 0
## 95 percent confidence interval:
##  -0.04634389  0.13261913
## sample estimates:
## mean of the differences
##              0.04313762
```

```
cohens_dav(data = x2a_ph1, x = RB, group = Reward_Category)
```

```
## # A tibble: 2 x 4
##   Reward_Category count  mean    sd
##   <fct>           <int> <dbl> <dbl>
## 1 High Reward        60 0.200 0.393
## 2 Low Reward         60 0.157 0.340
## [1] "Effect size Cohen's d(av):"
## [1] 0.117703
```

```
# Effect of reward category on response bias in phase 2
# (conditioning)
t.test(data = x2a_ph2, RB ~ Reward_Category, paired = TRUE)
```

```
##
##  Paired t-test
##
## data:  RB by Reward_Category
## t = -0.45723, df = 59, p-value = 0.6492
## alternative hypothesis: true difference in means is not equal to 0
## 95 percent confidence interval:
##  -0.11844178  0.07438172
## sample estimates:
## mean of the differences
##             -0.02203003
```

```r
cohens_dav(data = x2a_ph2, x = RB, group = Reward_Category)
```

```
## # A tibble: 2 x 4
##   Reward_Category count   mean    sd
##   <fct>           <int>  <dbl> <dbl>
## 1 High Reward        60 0.0969 0.419
## 2 Low Reward         60 0.119  0.309
## [1] "Effect size Cohen's d(av):"
## [1] -0.06057145
```

#### Experiment 2a (High Certainty Memory)

```r
# Effect of reward category on response bias in phase 1
# (pre-conditioning)
t.test(data = x2a_high_ph1, RB ~ Reward_Category, paired = TRUE)
```

```
##
##  Paired t-test
##
## data:  RB by Reward_Category
## t = 0.37186, df = 59, p-value = 0.7113
## alternative hypothesis: true difference in means is not equal to 0
## 95 percent confidence interval:
##  -0.0982859  0.1431548
## sample estimates:
## mean of the differences
##              0.02243445
```

```r
cohens_dav(data = x2a_high_ph1, x = RB, group = Reward_Category)
```

```
## # A tibble: 2 x 4
##   Reward_Category count  mean    sd
##   <fct>           <int> <dbl> <dbl>
## 1 High Reward        60 0.174 0.546
## 2 Low Reward         60 0.152 0.477
## [1] "Effect size Cohen's d(av):"
## [1] 0.04387066
```

```r
# Effect of reward category on response bias in phase 2
# (conditioning)
t.test(data = x2a_high_ph2, RB ~ Reward_Category, paired = TRUE)
```

```
##
##  Paired t-test
##
## data:  RB by Reward_Category
## t = -0.8236, df = 59, p-value = 0.4135
## alternative hypothesis: true difference in means is not equal to 0
## 95 percent confidence interval:
```

```
##  -0.16839160  0.07019206
## sample estimates:
## mean of the differences
##             -0.04909977
```

```r
cohens_dav(data = x2a_high_ph2, x = RB, group = Reward_Category)
```

```
## # A tibble: 2 x 4
##   Reward_Category count   mean    sd
##   <fct>           <int>  <dbl> <dbl>
## 1 High Reward        60 0.0598 0.581
## 2 Low Reward         60 0.109  0.464
## [1] "Effect size Cohen's d(av):"
## [1] -0.09397362
```

### Experiment 2b (All Memory)

```r
# Effect of reward category on response bias in phase 1
# (conditioning)
t.test(data = x2b_ph1, RB ~ Reward_Category, paired = TRUE)
```

```
##
##  Paired t-test
##
## data:  RB by Reward_Category
## t = -0.6231, df = 59, p-value = 0.5356
## alternative hypothesis: true difference in means is not equal to 0
## 95 percent confidence interval:
##  -0.18853314  0.09899785
## sample estimates:
## mean of the differences
##             -0.04476765
```

```r
cohens_dav(data = x2b_ph1, x = RB, group = Reward_Category)
```

```
## # A tibble: 2 x 4
##   Reward_Category count   mean    sd
##   <fct>           <int>  <dbl> <dbl>
## 1 High Reward        60 0.0600 0.446
## 2 Low Reward         60 0.105  0.404
## [1] "Effect size Cohen's d(av):"
## [1] -0.1053517
```

```r
# Effect of reward category on response bias in phase 2
# (post-conditioning)
t.test(data = x2b_ph2, RB ~ Reward_Category, paired = TRUE)
```

```
##
##  Paired t-test
```

```
##
## data:  RB by Reward_Category
## t = -0.42965, df = 59, p-value = 0.669
## alternative hypothesis: true difference in means is not equal to 0
## 95 percent confidence interval:
##  -0.1680474  0.1086377
## sample estimates:
## mean of the differences
##             -0.02970486
```

```
cohens_dav(data = x2b_ph2, x = RB, group = Reward_Category)
```

```
## # A tibble: 2 x 4
##   Reward_Category count  mean    sd
##   <fct>           <int> <dbl> <dbl>
## 1 High Reward        60 0.217 0.416
## 2 Low Reward         60 0.247 0.362
## [1] "Effect size Cohen's d(av):"
## [1] -0.07643966
```

### Experiment 2b (High Certainty Memory)

```
# Effect of reward category on response bias in phase 1
# (conditioning)
t.test(data = x2b_high_ph1, RB ~ Reward_Category, paired = TRUE)
```

```
##
##  Paired t-test
##
## data:  RB by Reward_Category
## t = 0.081713, df = 59, p-value = 0.9352
## alternative hypothesis: true difference in means is not equal to 0
## 95 percent confidence interval:
##  -0.1554430  0.1686789
## sample estimates:
## mean of the differences
##             0.006617941
```

```
cohens_dav(data = x2b_high_ph1, x = RB, group = Reward_Category)
```

```
## # A tibble: 2 x 4
##   Reward_Category count   mean    sd
##   <fct>           <int>  <dbl> <dbl>
## 1 High Reward        60 0.0292 0.586
## 2 Low Reward         60 0.0226 0.481
## [1] "Effect size Cohen's d(av):"
## [1] 0.01240013
```

```r
# Effect of reward category on response bias in phase 2
# (post-conditioning)
t.test(data = x2b_high_ph2, RB ~ Reward_Category, paired = TRUE)
```

```
##
##  Paired t-test
##
## data:  RB by Reward_Category
## t = -0.011532, df = 59, p-value = 0.9908
## alternative hypothesis: true difference in means is not equal to 0
## 95 percent confidence interval:
##  -0.1590630  0.1572401
## sample estimates:
## mean of the differences
##           -0.0009114496
```

```r
cohens_dav(data = x2b_high_ph2, x = RB, group = Reward_Category)
```

```
## # A tibble: 2 x 4
##   Reward_Category count  mean    sd
##   <fct>           <int> <dbl> <dbl>
## 1 High Reward        60 0.209 0.565
## 2 Low Reward         60 0.210 0.471
## [1] "Effect size Cohen's d(av):"
## [1] -0.001759444
```

# Experiment 2 - Linear Model

```
# Load necessary packages
library(dplyr)
library(tidyverse)
library(rstatix)
library(svglite)
library(rlang)
library(plotrix)
library(gridExtra)
library(tinytex)
library(formatR)
library(knitr)
library(lme4)
```

**This section contains linear model analyses and results for Experiment 2.**

## Data loading

```
# Load Experiment 2a and 2b data
data.x2a <- read.csv("adaptiveMemoryReplication/Exp2a_CleanData/Main/x2a_Regression.csv")  # all trial
data.x2b <- read.csv("adaptiveMemoryReplication/Exp2b_CleanData/Main/x2b_Regression.csv")  # all trial

# Filter to create dataset with only high certainty memory
# trials
data.high.x2a <- subset(data.x2a, Certainty == 0 | Certainty ==
    12 | Certainty == 60 | Certainty == 72)
data.high.x2b <- subset(data.x2b, Certainty == 0 | Certainty ==
    12 | Certainty == 60 | Certainty == 72)

# Insert Say_Old column based on memory responses Trials
# where participants were too slow are omitted (taken as
# NA)
data.x2a <- data.x2a %>%
    mutate(Say_Old = NA, Say_Old = ifelse(Certainty == 0, 1,
        Say_Old), Say_Old = ifelse(Certainty == 12, 1, Say_Old),
        Say_Old = ifelse(Certainty == 24, 1, Say_Old), Say_Old = ifelse(Certainty ==
            48, 0, Say_Old), Say_Old = ifelse(Certainty == 60,
            0, Say_Old), Say_Old = ifelse(Certainty == 72, 0,
            Say_Old))

data.x2b <- data.x2b %>%
    mutate(Say_Old = NA, Say_Old = ifelse(Certainty == 0, 1,
        Say_Old), Say_Old = ifelse(Certainty == 12, 1, Say_Old),
        Say_Old = ifelse(Certainty == 24, 1, Say_Old), Say_Old = ifelse(Certainty ==
```

```
            48, 0, Say_Old), Say_Old = ifelse(Certainty == 60,
            0, Say_Old), Say_Old = ifelse(Certainty == 72, 0,
            Say_Old))

data.high.x2a <- data.high.x2a %>%
    mutate(Say_Old = NA, Say_Old = ifelse(Certainty == 0, 1,
        Say_Old), Say_Old = ifelse(Certainty == 12, 1, Say_Old),
        Say_Old = ifelse(Certainty == 60, 0, Say_Old), Say_Old = ifelse(Certainty ==
            72, 0, Say_Old))

data.high.x2b <- data.high.x2b %>%
    mutate(Say_Old = NA, Say_Old = ifelse(Certainty == 0, 1,
        Say_Old), Say_Old = ifelse(Certainty == 12, 1, Say_Old),
        Say_Old = ifelse(Certainty == 60, 0, Say_Old), Say_Old = ifelse(Certainty ==
            72, 0, Say_Old))

data.x2a <- data.x2a[!is.na(data.x2a$Say_Old), ]
data.x2b <- data.x2b[!is.na(data.x2b$Say_Old), ]
data.high.x2a <- data.high.x2a[!is.na(data.high.x2a$Say_Old),
    ]
data.high.x2b <- data.high.x2b[!is.na(data.high.x2b$Say_Old),
    ]
```

## Prepare data for regression

```
# Prepare coded and factored data for regression analysis
data.x2a <- data.x2a %>%
    mutate(Phase = replace(Phase, Phase == "Rec_Memory", 0),
        Reward_Category = replace(Reward_Category, Reward_Category ==
            -1, 0))

data.x2b <- data.x2b %>%
    mutate(Phase = replace(Phase, Phase == "Rec_Memory", 0),
        Reward_Category = replace(Reward_Category, Reward_Category ==
            -1, 0))

data.high.x2a <- data.high.x2a %>%
    mutate(Phase = replace(Phase, Phase == "Rec_Memory", 0),
        Reward_Category = replace(Reward_Category, Reward_Category ==
            -1, 0))

data.high.x2b <- data.high.x2b %>%
    mutate(Phase = replace(Phase, Phase == "Rec_Memory", 0),
        Reward_Category = replace(Reward_Category, Reward_Category ==
            -1, 0))
```

## Data format

**Datasets:** Trial by trial summary of performance on the matching and memory tasks for all participants.

**Data variables:**

1. UserID: unique user identification
2. Rew_Subgroup: allocation of stimuli category to high reward ("Reward_Animals", "Reward_Objects")
3. Category: stimuli category ("Animal", "Object")
4. Reward_Category: stimuli reward category ("1":High Reward, "0":Low Reward)
5. Phase: phase in which stimuli was encoded
   - Experiment 2a: ("0":New Items, 1":Pre-conditioning,"2":Conditioning)
   - Experiment 2b: ("0":New Items, 1":Conditioning,"2":Post-conditioning)
6. Memory_RT: memory trial reaction time in ms
7. Memory_Correct: memory trial ("1" correct, "0" wrong)
8. Match_RT: matching trial reaction time in ms
9. Match_Correct: matching trial ("1":correct, "0":wrong)
10. Stim: word describing the stimuli image

Further unused variables: 11. Sex 12. Age 13. Stim_Type: ("old_img", "new_img") 14. Certainty: memory trial certainty response ("0":definitely old, "12":likely old, "24":maybe old, "48":maybe new, "60":likley new, "72":definitely new)

# 1. Main Analysis (LM Model)

As another complementary analysis of the effects of reward category on recognition memory performance across phases, we estimated generalized linear mixed-effect models (GLMMs) with a logit-link function using the lme4 R package (Bates et al., 2015). The dependent variable (Say_Old) was participants' categorical response to the memory test collapsed across response certainty with responding old (Say_Old = 1) or responding new (Say_Old = 0). We included main effects of reward category, with high reward category (Reward_Category = 1) and low reward category (Reward_Category = 0) and encoding phase for which we used dummy coding. New items (Phase = 0) were taken as the reference category for the other two phases (Phase = 1, Phase = 2). In terms of random effects, we first ran models with random intercepts for each participant (UserID) and stimuli item (Stim). Note that adding random slopes for each predictor did not result in model convergence, thus we omit this from our models and only retain random intercepts.

Confidence intervals were calculated using the confint function with bootstrapping method. Instead of relying on Wald's method obtained from the summary() function, we have used bootMer function calculate bootstrapped parametric p-values. For each fixed effect, we calculated the proportion of estimates $> 0$ (when beta is negative) or $< 0$ (when beta is positive) and output a p-value based on this.

```
# Set number of iterations for bootstrapping
Nsim = 100
```

## 1.1 Experiment 2a (All Memory)

```
glm1.1 <- glmer(Say_Old ~ 1 + Phase * Reward_Category + (1 |
    UserID) + (1 | Stim), family = binomial(link = "logit"),
    data = data.x2a, glmerControl(optimizer = "bobyqa"))
summary(glm1.1)
```

```
## Generalized linear mixed model fit by maximum likelihood (Laplace
##   Approximation) [glmerMod]
##  Family: binomial  ( logit )
## Formula: Say_Old ~ 1 + Phase * Reward_Category + (1 | UserID) + (1 | Stim)
##    Data: data.x2a
## Control: glmerControl(optimizer = "bobyqa")
##
##      AIC      BIC   logLik deviance df.resid
##  15009.3  15069.8  -7496.6  14993.3    14322
##
## Scaled residuals:
##     Min      1Q  Median      3Q     Max
## -3.7948 -0.5498 -0.2803  0.6189  5.7571
##
## Random effects:
##  Groups Name        Variance Std.Dev.
##  Stim   (Intercept) 0.3892   0.6238
##  UserID (Intercept) 0.2817   0.5308
## Number of obs: 14330, groups:  Stim, 240; UserID, 60
##
## Fixed effects:
##                         Estimate Std. Error z value Pr(>|z|)
## (Intercept)             -1.62722    0.10042 -16.204  < 2e-16 ***
## Phase1                   2.60220    0.10836  24.015  < 2e-16 ***
## Phase2                   2.76850    0.10932  25.326  < 2e-16 ***
## Reward_Category          0.12277    0.06180   1.987 0.046969 *
## Phase1:Reward_Category -0.35687    0.09779  -3.650 0.000263 ***
## Phase2:Reward_Category -0.26717    0.09999  -2.672 0.007543 **
## ---
## Signif. codes:  0 '***' 0.001 '**' 0.01 '*' 0.05 '.' 0.1 ' ' 1
##
## Correlation of Fixed Effects:
##             (Intr) Phase1 Phase2 Rwrd_C P1:R_C
## Phase1      -0.499
## Phase2      -0.495  0.739
## Rewrd_Ctgry -0.318  0.295  0.293
## Phs1:Rwrd_C  0.202 -0.469 -0.184 -0.632
## Phs2:Rwrd_C  0.197 -0.182 -0.471 -0.618  0.386
```

```r
confint.1.1 <- confint.merMod(glm1.1, method = "boot", nsim = Nsim,
    parallel = "multicore", ncpus = 4)
pvals.1.1 <- bootMer(glm1.1, FUN = fixef, nsim = Nsim, parallel = "multicore",
    ncpus = 4)  #
saveRDS(confint.1.1, "confint.1.1.rds")
saveRDS(pvals.1.1, "pvals.1.1.rds")
```

```r
# load previously run results
confint.1.1 <- readRDS(file = "confint.1.1.rds")
confint.1.1
```

```
##                              2.5 %      97.5 %
## .sig01                  0.552273451  0.70403474
## .sig02                  0.443057011  0.62078716
```

```
## (Intercept)           -1.852065607 -1.37740592
## Phase_Code1            2.386481004  2.85521508
## Phase_Code2            2.510385819  2.98737849
## Rew_Code1              0.006890637  0.24374664
## Phase_Code1:Rew_Code1 -0.590620571 -0.15517838
## Phase_Code2:Rew_Code1 -0.477158069 -0.07630656
```

```
pvals.1.1 <- readRDS(file = "pvals.1.1.rds")
pvals.1.1
```

```
##
## PARAMETRIC BOOTSTRAP
##
##
## Call:
## bootMer(x = glm1.1, FUN = fixef, nsim = Nsim, parallel = "multicore",
##     ncpus = 4)
##
##
## Bootstrap Statistics :
##       original        bias    std. error
## t1* -1.6272222 -0.0019058568  0.10351017
## t2*  2.6021966 -0.0149292632  0.11812854
## t3*  2.7685011 -0.0224876342  0.11438879
## t4*  0.1227685  0.0009577622  0.05924767
## t5* -0.3568748 -0.0010593196  0.09403050
## t6* -0.2671705  0.0168417640  0.10450239
```

```
pvals.1.1.list <- mean(pvals.1.1$t[, 1] > 0) * 2
pvals.1.1.list[2] <- mean(pvals.1.1$t[, 2] < 0) * 2
pvals.1.1.list[3] <- mean(pvals.1.1$t[, 3] < 0) * 2
pvals.1.1.list[4] <- mean(pvals.1.1$t[, 4] < 0) * 2
pvals.1.1.list[5] <- mean(pvals.1.1$t[, 5] > 0) * 2
pvals.1.1.list[6] <- mean(pvals.1.1$t[, 6] > 0) * 2

# label output
pvals.1.1.out <- as.list(pvals.1.1.list)
names(pvals.1.1.out) <- row.names(as.data.frame(summary(glm1.1)$coefficients))
pvals.1.1.out
```

```
## $`(Intercept)`
## [1] 0
##
## $Phase1
## [1] 0
##
## $Phase2
## [1] 0
##
## $Reward_Category
## [1] 0
##
## $`Phase1:Reward_Category`
```

```
## [1] 0
##
## $`Phase2:Reward_Category`
## [1] 0.02
```

Firstly, the GLMMM analysis on Say_Old responses can be used to analyse participants overall performance on the memory task. The 'Intercept' term which is negative, $\beta$ = -1.627, 95% CI [-1.852, -1.377], $p < .001$, represents the log odds of answering 'old' to a new item. Whereas, the 'Phase 1' and 'Phase 2' predictor estimates are positive, $\beta = 2.602$, 95% CI [2.386, 2.855], $p < .001$, and $\beta = 2.769$, 95% CI [2.510, 2.987], $p < .001$, respectively, showing that participants have successfully remembered previously seen items.

Secondly, it is also noteworthy to comment on the effect of reward category on log odds of response. The model shows a strong significant positive effect on response bias for items in the high reward category, $\beta = 0.123$, 95% CI [0.007, 0.244], $p < .001$. In other words, participants were more likely to respond 'old' to new items in the high reward category in general (a more liberal response bias) which could have influenced memory effects observed below. This needs further exploration through an analysis of the response bias in each phase.

In terms of main effects, there was a significant negative interaction between reward category and pre-conditioning phase, $\beta$ = -0.357, 95% CI [-0.591, -0.155], $p < .001$, and to a lesser extent in the conditioning phase, $\beta$ = -0.267, 95% CI [-0.477, -0.076], $p = .02$. The negative beta value translates to participants being more likely to correctly respond 'old' to previously seen items from the lower reward category in both phases. However, in the pre-conditioning phase, this effect is stronger, as found in the main analysis using classical t-tests as well as Bayes Factors.

## 1.2 Experiment 2a (High Certainty Memory)

```r
glm1.2 <- glmer(Say_Old ~ 1 + Phase * Reward_Category + (1 |
    UserID) + (1 | Stim), family = binomial(link = "logit"),
    data = data.high.x2a, glmerControl(optimizer = "bobyqa"))
summary(glm1.2)
```

```
## Generalized linear mixed model fit by maximum likelihood (Laplace
##   Approximation) [glmerMod]
##  Family: binomial  ( logit )
## Formula: Say_Old ~ 1 + Phase * Reward_Category + (1 | UserID) + (1 | Stim)
##    Data: data.high.x2a
## Control: glmerControl(optimizer = "bobyqa")
##
##      AIC      BIC   logLik deviance df.resid
##   8916.2   8974.0  -4450.1   8900.2    10114
##
## Scaled residuals:
##     Min      1Q  Median      3Q     Max
## -7.4279 -0.4001 -0.1521  0.4862  6.7929
##
## Random effects:
##  Groups Name        Variance Std.Dev.
##  Stim   (Intercept) 0.7113   0.8434
##  UserID (Intercept) 0.6633   0.8144
## Number of obs: 10122, groups:  Stim, 240; UserID, 60
##
```

```
## Fixed effects:
##                         Estimate Std. Error z value Pr(>|z|)
## (Intercept)             -2.07471    0.14725 -14.090   <2e-16 ***
## Phase1                   3.44362    0.14739  23.365   <2e-16 ***
## Phase2                   3.62886    0.14879  24.389   <2e-16 ***
## Reward_Category          0.15347    0.08823   1.740   0.0819 .
## Phase1:Reward_Category -0.31451    0.13063  -2.408   0.0161 *
## Phase2:Reward_Category -0.22354    0.13422  -1.665   0.0958 .
## ---
## Signif. codes:  0 '***' 0.001 '**' 0.01 '*' 0.05 '.' 0.1 ' ' 1
##
## Correlation of Fixed Effects:
##              (Intr) Phase1 Phase2 Rwrd_C P1:R_C
## Phase1       -0.494
## Phase2       -0.490  0.774
## Rewrd_Ctgry -0.313  0.313  0.310
## Phs1:Rwrd_C  0.213 -0.458 -0.209 -0.675
## Phs2:Rwrd_C  0.206 -0.204 -0.458 -0.657  0.439
```

```
confint.1.2 <- confint.merMod(glm1.2, method = "boot", nsim = Nsim,
    parallel = "multicore", ncpus = 4)
pvals.1.2 <- bootMer(glm1.2, FUN = fixef, nsim = Nsim, parallel = "multicore",
    ncpus = 4)  #
saveRDS(confint.1.2, "confint.1.2.rds")
saveRDS(pvals.1.2, "pvals.1.2.rds")
```

```
# load previously run results
confint.1.2 <- readRDS(file = "confint.1.2.rds")
confint.1.2
```

```
##                               2.5 %      97.5 %
## .sig01                   0.72037511  0.94975570
## .sig02                   0.66861866  0.99178011
## (Intercept)             -2.36541066 -1.81526571
## Phase_Code1              3.21776920  3.75004394
## Phase_Code2              3.34237319  3.91809572
## Rew_Code1                0.03117123  0.32265770
## Phase_Code1:Rew_Code1 -0.58341788 -0.13240764
## Phase_Code2:Rew_Code1 -0.46415541  0.06823654
```

```
pvals.1.2 <- readRDS(file = "pvals.1.2.rds")
pvals.1.2
```

```
##
## PARAMETRIC BOOTSTRAP
##
##
## Call:
## bootMer(x = glm1.2, FUN = fixef, nsim = Nsim, parallel = "multicore",
##     ncpus = 4)
##
##
```

```
## Bootstrap Statistics :
##       original       bias    std. error
## t1* -2.0747117 -0.007336701  0.14433769
## t2*  3.4436200  0.019443273  0.14880070
## t3*  3.6288592  0.021090762  0.15924934
## t4*  0.1534738  0.002751471  0.08854889
## t5* -0.3145115  0.001981744  0.14087816
## t6* -0.2235417 -0.003030675  0.13829279
```

```
pvals.1.2.list <- mean(pvals.1.2$t[, 1] > 0) * 2
pvals.1.2.list[2] <- mean(pvals.1.2$t[, 2] < 0) * 2
pvals.1.2.list[3] <- mean(pvals.1.2$t[, 3] < 0) * 2
pvals.1.2.list[4] <- mean(pvals.1.2$t[, 4] < 0) * 2
pvals.1.2.list[5] <- mean(pvals.1.2$t[, 5] > 0) * 2
pvals.1.2.list[6] <- mean(pvals.1.2$t[, 6] > 0) * 2

# label output
pvals.1.2.out <- as.list(pvals.1.2.list)
names(pvals.1.2.out) <- row.names(as.data.frame(summary(glm1.2)$coefficients))
pvals.1.2.out
```

```
## $`(Intercept)`
## [1] 0
##
## $Phase1
## [1] 0
##
## $Phase2
## [1] 0
##
## $Reward_Category
## [1] 0.04
##
## $`Phase1:Reward_Category`
## [1] 0.02
##
## $`Phase2:Reward_Category`
## [1] 0.06
```

When considering only high certainty trials from the memory test, the negative trend in the interaction between reward category and encoding phases still stood, however they were not as strong as when considering all memory trials. More specifically, there was a stronger interaction between reward category and the pre-conditioning phase, $\beta$ = -0.315, 95% CI [-0.583, -0.132], p = .02, and to a lesser extent in the conditioning phase, $\beta$ = -0.224, 95% CI [-0.464, 0.068], p = .06. This agrees with our findings in the main analysis.

Furthermore, the model shows a weakly significant positive effect on response bias for items in the high reward category, $\beta$ = 0.04, 95% CI [0.031, 0.323]. The participants had a more liberal response bias for items in the high reward category, however this effect is not as strong as when considering all memory trials.

## 1.3 Experiment 2b (All Memory)

In experiment 2b, participants first underwent a conditioning phase where stimuli were paired with high/low reward followed by a post-conditioning phase with no reward conditioning on trials.

```
glm1.3 <- glmer(Say_Old ~ 1 + Phase + Reward_Category + Phase:Reward_Category +
    (1 | UserID) + (1 | Stim), family = binomial(link = "logit"),
    data = data.x2b, glmerControl(optimizer = "bobyqa"))
summary(glm1.3)
```

```
## Generalized linear mixed model fit by maximum likelihood (Laplace
##   Approximation) [glmerMod]
##  Family: binomial  ( logit )
## Formula: Say_Old ~ 1 + Phase + Reward_Category + Phase:Reward_Category +
##     (1 | UserID) + (1 | Stim)
##    Data: data.x2b
## Control: glmerControl(optimizer = "bobyqa")
##
##      AIC      BIC   logLik deviance df.resid
##  15481.5  15542.0  -7732.7  15465.5    14297
##
## Scaled residuals:
##     Min      1Q  Median      3Q     Max
## -3.5043 -0.5968 -0.3007  0.6423  6.0225
##
## Random effects:
##  Groups Name        Variance Std.Dev.
##  Stim   (Intercept) 0.4278   0.6540
##  UserID (Intercept) 0.2091   0.4573
## Number of obs: 14305, groups:  Stim, 240; UserID, 60
##
## Fixed effects:
##                        Estimate Std. Error z value Pr(>|z|)
## (Intercept)           -1.492497   0.095161 -15.684   <2e-16 ***
## Phase1                 2.527289   0.110980  22.773   <2e-16 ***
## Phase2                 2.072501   0.108983  19.017   <2e-16 ***
## Reward_Category        0.059483   0.060253   0.987    0.324
## Phase1:Reward_Category  0.054552   0.099382   0.549    0.583
## Phase2:Reward_Category  0.003034   0.094947   0.032    0.975
## ---
## Signif. codes:  0 '***' 0.001 '**' 0.01 '*' 0.05 '.' 0.1 ' ' 1
##
## Correlation of Fixed Effects:
##             (Intr) Phase1 Phase2 Rwrd_C P1:R_C
## Phase1      -0.531
## Phase2      -0.539  0.761
## Rewrd_Ctgry -0.323  0.278  0.283
## Phs1:Rwrd_C  0.196 -0.445 -0.170 -0.607
## Phs2:Rwrd_C  0.205 -0.175 -0.438 -0.635  0.383
```

```
confint.1.3 <- confint.merMod(glm1.3, method = "boot", nsim = Nsim,
    parallel = "multicore", ncpus = 4)
pvals.1.3 <- bootMer(glm1.3, FUN = fixef, nsim = Nsim, parallel = "multicore",
```

```
    ncpus = 4)  #
saveRDS(confint.1.3, "confint.1.3.rds")
saveRDS(pvals.1.3, "pvals.1.3.rds")
```

```
# load previously run results
confint.1.3 <- readRDS(file = "confint.1.3.rds")
confint.1.3
```

```
##                              2.5 %     97.5 %
## .sig01                  0.56627835  0.7263420
## .sig02                  0.34903209  0.5612589
## (Intercept)            -1.71365823 -1.3174433
## Phase_Code1             2.32571749  2.7433312
## Phase_Code2             1.87961382  2.2683693
## Rew_Code1              -0.05546147  0.1641952
## Phase_Code1:Rew_Code1 -0.14215028  0.2366704
## Phase_Code2:Rew_Code1 -0.17493803  0.2228373
```

```
pvals.1.3 <- readRDS(file = "pvals.1.3.rds")
pvals.1.3
```

```
##
## PARAMETRIC BOOTSTRAP
##
##
## Call:
## bootMer(x = glm1.3, FUN = fixef, nsim = Nsim, parallel = "multicore",
##     ncpus = 4)
##
##
## Bootstrap Statistics :
##         original       bias    std. error
## t1* -1.492485031 -0.013909633  0.10416798
## t2*  2.527277477  0.008977058  0.11378228
## t3*  2.072485482  0.007538161  0.11117134
## t4*  0.059481007  0.001451639  0.06743717
## t5*  0.054557267 -0.001219650  0.10780642
## t6*  0.003035009  0.002398011  0.11205552
```

```
pvals.1.3.list <- mean(pvals.1.3$t[, 1] > 0) * 2
pvals.1.3.list[2] <- mean(pvals.1.3$t[, 2] < 0) * 2
pvals.1.3.list[3] <- mean(pvals.1.3$t[, 3] < 0) * 2
pvals.1.3.list[4] <- mean(pvals.1.3$t[, 4] < 0) * 2
pvals.1.3.list[5] <- mean(pvals.1.3$t[, 5] < 0) * 2
pvals.1.3.list[6] <- mean(pvals.1.3$t[, 6] < 0) * 2

# label output
pvals.1.3.out <- as.list(pvals.1.3.list)
names(pvals.1.3.out) <- row.names(as.data.frame(summary(glm1.3)$coefficients))
pvals.1.3.out
```

```
## $`(Intercept)`
## [1] 0
##
## $Phase1
## [1] 0
##
## $Phase2
## [1] 0
##
## $Reward_Category
## [1] 0.34
##
## $`Phase1:Reward_Category`
## [1] 0.58
##
## $`Phase2:Reward_Category`
## [1] 0.94
```

Unlike in experiment 2a, the model did show an effect of reward category on response bias, $\beta = 0.059$, 95% CI [-0.055, 0.164], p = .34. In other words, participants were not biased in responding 'old' to items from a specific category.

In terms of main effects, there was no evidence for an interaction between reward category and the conditioning phase, $\beta = 0.059$, 95% CI [-0.142, 0.237], p = .58, and similarly, no effect between reward category and the post-conditioning phase, $\beta = 0.003$, 95% CI [-0.175, 0.223], p = .94. The beta values confidence intervals extend from negative to positive values, showing that there is no significant effect in either direction. In other words, participants were equally likely to correctly responded 'old' to previously seen items from high and low reward categories. Again this agrees with what was found in the main analysis using classical t-tests as well as Bayes Factors.

## 1.4 Experiment 2b (High Certainty Memory)

```
glm1.4 <- glmer(Say_Old ~ 1 + Phase + Reward_Category + Phase:Reward_Category +
    (1 | UserID) + (1 | Stim), family = binomial(link = "logit"),
    data = data.high.x2b, glmerControl(optimizer = "bobyqa"))
summary(glm1.4)
```

```
## Generalized linear mixed model fit by maximum likelihood (Laplace
##   Approximation) [glmerMod]
##  Family: binomial  ( logit )
## Formula: Say_Old ~ 1 + Phase + Reward_Category + Phase:Reward_Category +
##     (1 | UserID) + (1 | Stim)
##    Data: data.high.x2b
## Control: glmerControl(optimizer = "bobyqa")
##
##      AIC      BIC   logLik deviance df.resid
##   8903.9   8961.4  -4443.9   8887.9     9726
##
## Scaled residuals:
##     Min      1Q  Median      3Q     Max
## -5.3650 -0.4256 -0.1866  0.4999  7.0748
##
```

```
## Random effects:
##  Groups Name        Variance Std.Dev.
##  Stim   (Intercept) 0.6673   0.8169
##  UserID (Intercept) 0.4175   0.6461
## Number of obs: 9734, groups:  Stim, 240; UserID, 60
##
## Fixed effects:
##                          Estimate Std. Error z value Pr(>|z|)
## (Intercept)              -1.77041    0.12861 -13.766   <2e-16 ***
## Phase1                    3.42157    0.14511  23.579   <2e-16 ***
## Phase2                    2.77739    0.14099  19.699   <2e-16 ***
## Reward_Category          -0.15648    0.08712  -1.796   0.0725 .
## Phase1:Reward_Category    0.07183    0.13557   0.530   0.5962
## Phase2:Reward_Category    0.16946    0.12893   1.314   0.1887
## ---
## Signif. codes:  0 '***' 0.001 '**' 0.01 '*' 0.05 '.' 0.1 ' ' 1
##
## Correlation of Fixed Effects:
##              (Intr) Phase1 Phase2 Rwrd_C P1:R_C
## Phase1       -0.517
## Phase2       -0.529  0.753
## Rewrd_Ctgry -0.320  0.285  0.293
## Phs1:Rwrd_C  0.207 -0.463 -0.189 -0.643
## Phs2:Rwrd_C  0.216 -0.192 -0.447 -0.675  0.432
```

```
confint.1.4 <- confint.merMod(glm1.4, method = "boot", nsim = Nsim,
    parallel = "multicore", ncpus = 4)
pvals.1.4 <- bootMer(glm1.4, FUN = fixef, nsim = Nsim, parallel = "multicore",
    ncpus = 4)  #
saveRDS(confint.1.4, "confint.1.4.rds")
saveRDS(pvals.1.4, "pvals.1.4.rds")
```

```
# load previously run results
confint.1.4 <- readRDS(file = "confint.1.4.rds")
confint.1.4
```

```
##                                2.5 %       97.5 %
## .sig01                    0.70679050  0.917751698
## .sig02                    0.52709779  0.760275149
## (Intercept)              -2.07336986 -1.553077809
## Phase_Code1               3.17050188  3.750931525
## Phase_Code2               2.52223299  3.055105782
## Rew_Code1                -0.30209094 -0.001733415
## Phase_Code1:Rew_Code1 -0.21273939  0.365317216
## Phase_Code2:Rew_Code1 -0.08831167  0.406317917
```

```
pvals.1.4 <- readRDS(file = "pvals.1.4.rds")
pvals.1.4
```

```
##
## PARAMETRIC BOOTSTRAP
##
```

```
##
## Call:
## bootMer(x = glm1.4, FUN = fixef, nsim = Nsim, parallel = "multicore",
##     ncpus = 4)
##
##
## Bootstrap Statistics :
##        original       bias    std. error
## t1* -1.77040994 -0.004141587  0.11433021
## t2*  3.42159630  0.012518479  0.12724825
## t3*  2.77741190  0.018026120  0.13309932
## t4* -0.15647508 -0.001131603  0.07610727
## t5*  0.07181186  0.014909306  0.12315930
## t6*  0.16944449 -0.006409801  0.11301704
```

```
pvals.1.4.list <- mean(pvals.1.4$t[, 1] > 0) * 2
pvals.1.4.list[2] <- mean(pvals.1.4$t[, 2] < 0) * 2
pvals.1.4.list[3] <- mean(pvals.1.4$t[, 3] < 0) * 2
pvals.1.4.list[4] <- mean(pvals.1.4$t[, 4] > 0) * 2
pvals.1.4.list[5] <- mean(pvals.1.4$t[, 5] < 0) * 2
pvals.1.4.list[6] <- mean(pvals.1.4$t[, 6] < 0) * 2

# label output
pvals.1.4.out <- as.list(pvals.1.4.list)
names(pvals.1.4.out) <- row.names(as.data.frame(summary(glm1.4)$coefficients))
pvals.1.4.out
```

```
## $`(Intercept)`
## [1] 0
##
## $Phase1
## [1] 0
##
## $Phase2
## [1] 0
##
## $Reward_Category
## [1] 0.04
##
## $`Phase1:Reward_Category`
## [1] 0.44
##
## $`Phase2:Reward_Category`
## [1] 0.18
```

In contrast to the results obtained from analysing all memory trials in experiment 2b, a model of the high certainty memory trials show a weakly significant negative effect reward category on log odds of response, $\beta$ = -0.156, 95% CI [-0.302, -0.002], p = .04. In other words, participants were biased in responding 'old' to items from the low reward category. This effect again needs to be analysed further by phase to determine if it contributes strongly to our main effects (or lack thereof).

Furthermore, as in the analysis with all trials, there was no evidence for an interaction between reward category and the conditioning phase, $\beta$ = 0.072, 95% CI [-0.213, 0.365], p = .44, and similarly, no effect between reward category and the post-conditioning phase, $\beta$ = 0.169, 95% CI [-0.088, 0.406], p = .18. In other words, participants were equally likely to correctly responded 'old' to previously seen items from high and low reward categories. Again this agrees with what was found in the main analysis using classical t-tests as well as Bayes Factors.

## 2. Supplementary Analysis (Exclusion Based on Surprisal)

```
# Participants to exclude as they anticipated the memory
# test
data.x2a.exclude <- read.csv("adaptiveMemoryReplication/Exp2a_CleanData/Supp/x2a_Surprisal_Exclude.csv"
data.x2b.exclude <- read.csv("adaptiveMemoryReplication/Exp2b_CleanData/Supp/x2b_Surprisal_Exclude.csv"
x2a.exclude <- subset(data.x2a.exclude, Exclude.Surprise == 1,
    UserID)
x2b.exclude <- subset(data.x2b.exclude, Exclude.Surprise == 1,
    UserID)

# Data subset (not surprised)
data.x2a.ns <- data.x2a[!data.x2a$UserID %in% x2a.exclude$UserID,
    ]
data.x2b.ns <- data.x2b[!data.x2b$UserID %in% x2b.exclude$UserID,
    ]
data.x2a.high.ns <- data.high.x2a[!data.high.x2a$UserID %in%
    x2a.exclude$UserID, ]
data.x2b.high.ns <- data.high.x2b[!data.high.x2b$UserID %in%
    x2b.exclude$UserID, ]
```

## 2.1 Experiment 2a (All Memory)

```r
glm2.1 <- glmer(Say_Old ~ 1 + Phase * Reward_Category + (1 |
    UserID) + (1 | Stim), family = binomial(link = "logit"),
    data = data.x2a.ns, glmerControl(optimizer = "bobyqa"))
summary(glm2.1)
```

```
## Generalized linear mixed model fit by maximum likelihood (Laplace
##   Approximation) [glmerMod]
##  Family: binomial  ( logit )
## Formula: Say_Old ~ 1 + Phase * Reward_Category + (1 | UserID) + (1 | Stim)
##    Data: data.x2a.ns
## Control: glmerControl(optimizer = "bobyqa")
##
##      AIC      BIC   logLik deviance df.resid
##  11963.8  12022.5  -5973.9  11947.8    11455
##
## Scaled residuals:
##     Min      1Q  Median      3Q     Max
## -3.8425 -0.5445 -0.2774  0.6037  6.0066
##
## Random effects:
##  Groups Name        Variance Std.Dev.
##  Stim   (Intercept) 0.3741   0.6117
##  UserID (Intercept) 0.2981   0.5459
## Number of obs: 11463, groups:  Stim, 240; UserID, 48
##
## Fixed effects:
##                         Estimate Std. Error z value Pr(>|z|)
## (Intercept)             -1.57894    0.10921 -14.457  < 2e-16 ***
## Phase1                   2.64643    0.11347  23.322  < 2e-16 ***
## Phase2                   2.75088    0.11435  24.057  < 2e-16 ***
## Reward_Category          0.06575    0.06863   0.958  0.33804
## Phase1:Reward_Category -0.31848    0.11005  -2.894  0.00381 **
## Phase2:Reward_Category -0.16440    0.11245  -1.462  0.14372
## ---
## Signif. codes:  0 '***' 0.001 '**' 0.01 '*' 0.05 '.' 0.1 ' ' 1
##
## Correlation of Fixed Effects:
##             (Intr) Phase1 Phase2 Rwrd_C P1:R_C
## Phase1      -0.467
## Phase2      -0.463  0.694
## Rewrd_Ctgry -0.320  0.308  0.306
## Phs1:Rwrd_C  0.200 -0.502 -0.189 -0.624
## Phs2:Rwrd_C  0.196 -0.186 -0.501 -0.610  0.374
```

## 2.2 Experiment 2a (High Certainty Memory)

```
glm2.2 <- glmer(Say_Old ~ 1 + Phase * Reward_Category + (1 |
    UserID) + (1 | Stim), family = binomial(link = "logit"),
    data = data.x2a.high.ns, glmerControl(optimizer = "bobyqa"))
summary(glm2.2)
```

```
## Generalized linear mixed model fit by maximum likelihood (Laplace
##   Approximation) [glmerMod]
##  Family: binomial  ( logit )
## Formula: Say_Old ~ 1 + Phase * Reward_Category + (1 | UserID) + (1 | Stim)
##    Data: data.x2a.high.ns
## Control: glmerControl(optimizer = "bobyqa")
##
##      AIC      BIC   logLik deviance df.resid
##   7010.9   7066.9  -3497.5   6994.9     8023
##
## Scaled residuals:
##     Min      1Q  Median      3Q     Max
## -6.7338 -0.3947 -0.1199  0.4679  6.0526
##
## Random effects:
##  Groups Name        Variance Std.Dev.
##  Stim   (Intercept) 0.6907   0.8311
##  UserID (Intercept) 0.6930   0.8325
## Number of obs: 8031, groups:  Stim, 240; UserID, 48
##
## Fixed effects:
##                         Estimate Std. Error z value Pr(>|z|)
## (Intercept)             -1.95243    0.16057 -12.160   <2e-16 ***
## Phase1                   3.53244    0.15589  22.660   <2e-16 ***
## Phase2                   3.58133    0.15622  22.925   <2e-16 ***
## Reward_Category          0.09438    0.09758   0.967   0.3335
## Phase1:Reward_Category -0.31429    0.14874  -2.113   0.0346 *
## Phase2:Reward_Category -0.10871    0.15143  -0.718   0.4728
## ---
## Signif. codes:  0 '***' 0.001 '**' 0.01 '*' 0.05 '.' 0.1 ' ' 1
##
## Correlation of Fixed Effects:
##             (Intr) Phase1 Phase2 Rwrd_C P1:R_C
## Phase1      -0.459
## Phase2      -0.457  0.727
## Rewrd_Ctgry -0.310  0.320  0.320
## Phs1:Rwrd_C  0.206 -0.494 -0.209 -0.655
## Phs2:Rwrd_C  0.200 -0.202 -0.487 -0.644  0.416
```

## 2.3 Experiment 2b (All Memory)

```
glm2.3 <- glmer(Say_Old ~ 1 + Phase * Reward_Category + (1 |
    UserID) + (1 | Stim), family = binomial(link = "logit"),
```

```
    data = data.x2b.ns, glmerControl(optimizer = "bobyqa"))
summary(glm2.3)
```

```
## Generalized linear mixed model fit by maximum likelihood (Laplace
##   Approximation) [glmerMod]
##  Family: binomial  ( logit )
## Formula: Say_Old ~ 1 + Phase * Reward_Category + (1 | UserID) + (1 | Stim)
##    Data: data.x2b.ns
## Control: glmerControl(optimizer = "bobyqa")
##
##      AIC      BIC   logLik deviance df.resid
##  11387.0  11445.1  -5685.5  11371.0    10506
##
## Scaled residuals:
##     Min      1Q  Median      3Q     Max
## -3.4358 -0.5869 -0.3059  0.6388  5.3330
##
## Random effects:
##  Groups Name        Variance Std.Dev.
##  Stim   (Intercept) 0.4288   0.6548
##  UserID (Intercept) 0.1965   0.4432
## Number of obs: 10514, groups:  Stim, 240; UserID, 44
##
## Fixed effects:
##                          Estimate Std. Error z value Pr(>|z|)
## (Intercept)              -1.50647    0.10387 -14.503   <2e-16 ***
## Phase1                    2.50209    0.11901  21.024   <2e-16 ***
## Phase2                    2.09131    0.11677  17.910   <2e-16 ***
## Reward_Category           0.02011    0.07083   0.284    0.777
## Phase1:Reward_Category    0.12083    0.11590   1.043    0.297
## Phase2:Reward_Category    0.07176    0.11123   0.645    0.519
## ---
## Signif. codes:  0 '***' 0.001 '**' 0.01 '*' 0.05 '.' 0.1 ' ' 1
##
## Correlation of Fixed Effects:
##             (Intr) Phase1 Phase2 Rwrd_C P1:R_C
## Phase1      -0.516
## Phase2      -0.524  0.718
## Rewrd_Ctgry -0.345  0.302  0.307
## Phs1:Rwrd_C  0.210 -0.480 -0.185 -0.612
## Phs2:Rwrd_C  0.219 -0.188 -0.476 -0.637  0.385
```

### 2.4 Experiment 2b (High Certainty Memory)

```
glm2.4 <- glmer(Say_Old ~ 1 + Phase * Reward_Category + (1 |
    UserID) + (1 | Stim), family = binomial(link = "logit"),
    data = data.x2b.high.ns, glmerControl(optimizer = "bobyqa"))
summary(glm2.4)
```

```
## Generalized linear mixed model fit by maximum likelihood (Laplace
##   Approximation) [glmerMod]
```

```
##  Family: binomial  ( logit )
## Formula: Say_Old ~ 1 + Phase * Reward_Category + (1 | UserID) + (1 | Stim)
##    Data: data.x2b.high.ns
## Control: glmerControl(optimizer = "bobyqa")
##
##      AIC      BIC   logLik deviance df.resid
##   6573.2   6628.3  -3278.6   6557.2     7241
##
## Scaled residuals:
##     Min      1Q  Median      3Q     Max
## -4.8712 -0.4031 -0.1881  0.4904  6.3897
##
## Random effects:
##  Groups Name        Variance Std.Dev.
##  Stim   (Intercept) 0.6968   0.8347
##  UserID (Intercept) 0.3630   0.6025
## Number of obs: 7249, groups:  Stim, 240; UserID, 44
##
## Fixed effects:
##                        Estimate Std. Error z value Pr(>|z|)
## (Intercept)             -1.8609     0.1399 -13.305   <2e-16 ***
## Phase1                   3.4304     0.1583  21.676   <2e-16 ***
## Phase2                   2.8693     0.1538  18.651   <2e-16 ***
## Reward_Category         -0.2131     0.1027  -2.075   0.0379 *
## Phase1:Reward_Category   0.1996     0.1583   1.261   0.2074
## Phase2:Reward_Category   0.2714     0.1515   1.791   0.0732 .
## ---
## Signif. codes:  0 '***' 0.001 '**' 0.01 '*' 0.05 '.' 0.1 ' ' 1
##
## Correlation of Fixed Effects:
##             (Intr) Phase1 Phase2 Rwrd_C P1:R_C
## Phase1      -0.518
## Phase2      -0.529  0.719
## Rewrd_Ctgry -0.341  0.300  0.310
## Phs1:Rwrd_C  0.221 -0.488 -0.200 -0.648
## Phs2:Rwrd_C  0.230 -0.199 -0.475 -0.677  0.434
```

# Experiment 3 Main Analysis

```r
# Load necessary packages
library(dplyr)
library(tidyverse)
library(rstatix)
library(ggplot2)
library(ggpubr)
library(ggprism)
library(svglite)
library(rlang)
library(plotrix)
library(gridExtra)
library(BayesFactor)
library(tinytex)
library(formatR)
library(knitr)
source("funcs.R")
```

This section contains the analysis and results associated with Experiment 3 in the article 'Reward conditioning may not have an effect on category-specific memory'. Experiment 3 consisted of pre-conditioning and conditioning phases, followed by a 24-hour delayed memory test.

## Data loading

```r
# Load Experiment 3 data
data.x3 <- read.csv("adaptiveMemoryReplication/Exp3_CleanData/Main/x3_Anova.csv")  # all memory data
data.x3.high <- read.csv("adaptiveMemoryReplication/Exp3_CleanData/Main/x3_High_Anova.csv")  # only hig
# Change phase labels for readability
data.x3$Phase[data.x3$Phase == "Ph1"] <- "Pre-conditioning"
data.x3$Phase[data.x3$Phase == "Ph2"] <- "Conditioning"
data.x3.high$Phase[data.x3.high$Phase == "Ph1"] <- "Pre-conditioning"
data.x3.high$Phase[data.x3.high$Phase == "Ph2"] <- "Conditioning"
# Reorder variables for graphs
data.x3$Reward_Category <- factor(data.x3$Reward_Category, levels = c("High Reward",
    "Low Reward"))
data.x3$Phase <- factor(data.x3$Phase, levels = c("Pre-conditioning",
    "Conditioning"))
data.x3.high$Reward_Category <- factor(data.x3.high$Reward_Category,
    levels = c("High Reward", "Low Reward"))
data.x3.high$Phase <- factor(data.x3.high$Phase, levels = c("Pre-conditioning",
    "Conditioning"))
```

# Data format

**Datasets:**

By participant summary of performance on the matching and memory tasks. There are two summary datasets for each experiment:

1. data.x3 summarises all memory trials
2. data.x3.high summarises memory trials in which participants responded with higher certainty (confidence rating). This includes trials with 'Definitely Old/New' and 'Likely Old/New' responses, and excludes 'Maybe Old/New' responses.

**Data variables:**

1. UserID: unique user identification
2. Category: stimuli category ("Animal", "Object")
3. Reward_Category: stimuli reward category (High Reward", "Low Reward")
4. Phase: phase in which stimuli was encoded Experiment 3: ("Pre-conditioning", "Conditioning")
5. CR: corrected recognition scores from memory task
6. DP: d-prime memory sensitivity in memory task (as per signal detection theory)
7. MA: matching accuracy in matching task
8. RT: reaction time (ms) in matching task
9. RB: response bias in memory task (as per signal detection theory)

Further unused variables: 10. Rew_Subgroup: allocation of situmuli category to high reward ("Reward_Animals", "Reward_Objects") 11. Age 12. Sex 13. HR: hit rate in memory task 14. FA: false alarm rate in memory task

# 1. Main Analysis (Frequentists statistics)

Recognition memory performance was calculated using two measures: corrected recognition (hit rate - false alarm rate) and (d-prime) memory sensitivity as per signal detection theory. Parametric tests were used since the sample size (n = 170) is large enough (n > 30) to assume that data follows normality requirements.

Firstly, a 2x2 factor repeated measures Anova was done to characterise memory by phase and reward category on the memory of items. This analysis was performed on both measures of memory. Following this, more specifically, the effect of reward category (high vs. low reward) on the memory of items from each phase was quantified using two-tailed paired t-tests with alpha = .05.

For each experiment, we then repeated the analysis taking into account only high-certainty memory responses.

## 1.1 Experiment 3 (All Memory)

We conducted a repeated measures two-factor ANOVA on memory performance (both corrected recognition and d-prime) with encoding phase (pre-conditioning, conditioning) and reward category (high reward, low reward) to summarize the main effects.

**Corrected recognition (CR) by phase and reward category**

```
# Summary table and graph
aggregate(CR ~ Reward_Category + Phase, data.x3, FUN = function(CR) c(mean = mean(CR),
    se = std.error(CR)))
```

```
##   Reward_Category            Phase    CR.mean      CR.se
## 1     High Reward Pre-conditioning 0.42382787 0.01494930
## 2      Low Reward Pre-conditioning 0.42556051 0.01392110
## 3     High Reward     Conditioning 0.48226794 0.01472890
## 4      Low Reward     Conditioning 0.46310258 0.01484901
```

```
x3.CR = plot_by_group(data = data.x3, yvar = "CR", ylim = c(0,
    1.2), ylab = "Corrected Recognition", subtitle = "Experiment 3 (All Memory)",
    tag = "1.1 A")
x3.CR
```

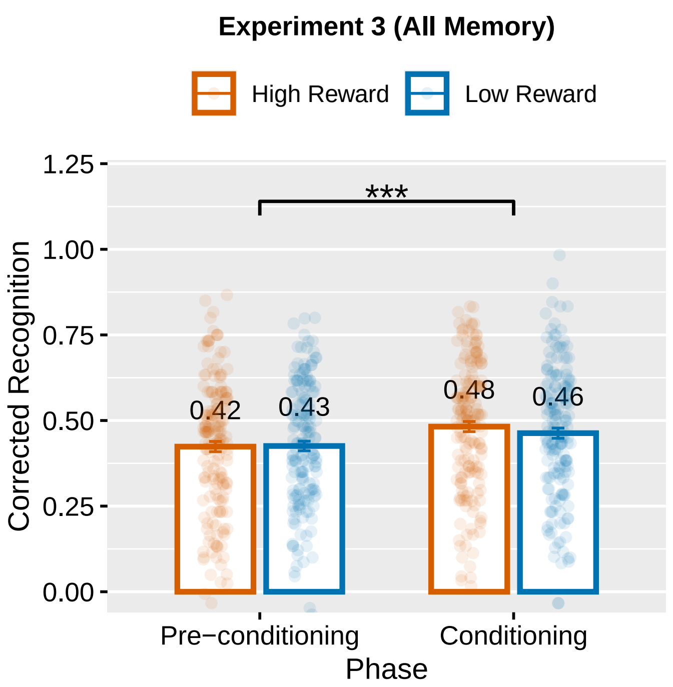


```
# Repeated measures two-factor ANOVA on corrected
# recognition
anova_test(CR ~ Phase * Reward_Category + Error(UserID/(Phase *
    Reward_Category)), data = data.x3)
```

```
## ANOVA Table (type III tests)
##
##                 Effect DFn DFd      F        p p<.05      ges
## 1                Phase   1 169 30.493 1.24e-07     * 0.016000
## 2      Reward_Category   1 169  0.719 3.98e-01       0.000526
## 3 Phase:Reward_Category   1 169  3.384 6.80e-02       0.000755
```

The repeated measures ANOVA revealed an effect of phase, $F(1,169) = 30.49$, $p < .001$, $\eta^2 = .02$. There was no significant effect of reward category $F(1,169) = 0.72$, $p = .40$, $\eta^2 = .001$ on corrected recognition. However there was a weak interaction between encoding phase and the reward category associated with the item $F(1,169) = 3{,}38$, $p = .07$, $\eta^2 = 0.001$. This indicates that memory, as measured by corrected recognition

was influenced by an item's reward category across phases. We next repeat the same analysis for d-prime scores.

**d-prime (DP) by phase and reward category**

```
# Summary table and graph
aggregate(DP ~ Reward_Category + Phase, data.x3, FUN = function(DP) c(mean = mean(DP),
    se = std.error(DP)))
```

```
##   Reward_Category            Phase    DP.mean      DP.se
## 1     High Reward Pre-conditioning 1.93583607 0.05584367
## 2      Low Reward Pre-conditioning 1.90921724 0.04983734
## 3     High Reward     Conditioning 2.14549811 0.05483676
## 4      Low Reward     Conditioning 2.05587529 0.05774848
```

```
x3.DP = plot_by_group(data = data.x3, yvar = "DP", ylim = c(0,
    4.6), ylab = "d-prime", subtitle = "Experiment 3 (All Memory)",
    tag = "1.1 B")
ggsave(file = "x3.DP.svg", plot = x3.DP, width = 10, height = 10,
    units = "cm")
x3.DP
```

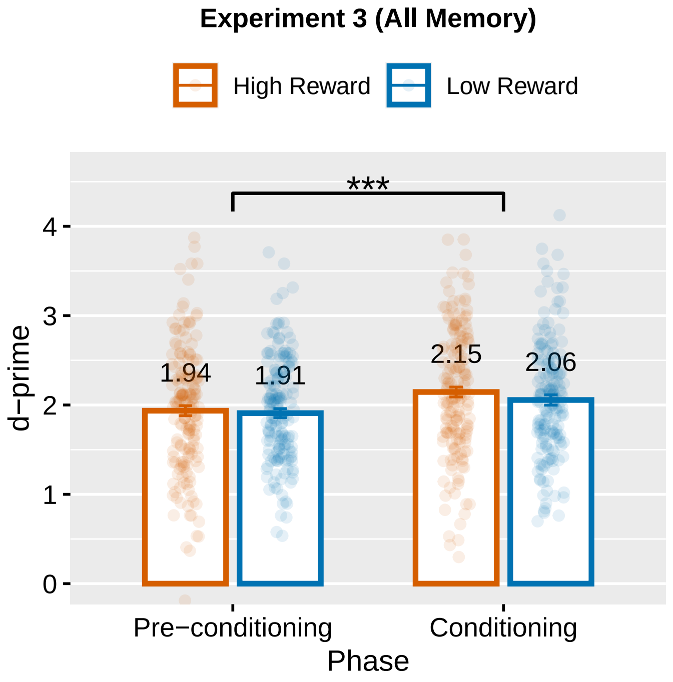


```
# Repeated measures two-factor ANOVA on d-prime scores
anova_test(DP ~ Phase * Reward_Category + Error(UserID/(Phase *
    Reward_Category)), data = data.x3)
```

```
## ANOVA Table (type III tests)
##
```

```
##                  Effect DFn DFd      F        p p<.05      ges
## 1                 Phase   1 169 33.257 3.76e-08     * 0.015000
## 2       Reward_Category   1 169  1.872 1.73e-01       0.002000
## 3 Phase:Reward_Category   1 169  2.232 1.37e-01       0.000491
```

The repeated measures ANOVA on d-primes revealed a strong effect of phase, F(1,169) = 33.26, $p < .001$, $\eta^2 = .015$, but not reward category F(1,169) = 1.87, p = .17, $\eta^2 = .002$. Again, there was no significant interaction between encoding phase and the reward category of item F(1,169) = 2.23, p = .14, $\eta^2 = 0.001$.

Following this, we conducted paired t-tests to more specifically characterise the effect of reward category on memory of items from each encoding phase.

```r
# Create subsets for each phase from data.x3 (all memory)
x3_ph1 <- subset(data.x3, Phase == "Pre-conditioning")
x3_ph2 <- subset(data.x3, Phase == "Conditioning")
```

```r
# Effect of reward category on memory in phase 1
# (pre-conditioning) Corrected recognition (CR)
t.test(data = x3_ph1, CR ~ Reward_Category, paired = TRUE)
```

```
##
##  Paired t-test
##
## data:  CR by Reward_Category
## t = -0.14979, df = 169, p-value = 0.8811
## alternative hypothesis: true mean difference is not equal to 0
## 95 percent confidence interval:
##  -0.02456756  0.02110229
## sample estimates:
## mean difference
##    -0.001732639
```

```r
cohens_dav(data = x3_ph1, x = CR, group = Reward_Category)
```

```
## # A tibble: 2 x 4
##   Reward_Category count  mean    sd
##   <fct>           <int> <dbl> <dbl>
## 1 High Reward       170 0.424 0.195
## 2 Low Reward        170 0.426 0.182
## [1] "Effect size Cohen's d(av):"
## [1] -0.009205783
```

```r
# d-prime (DP)
t.test(data = x3_ph1, DP ~ Reward_Category, paired = TRUE)
```

```
##
##  Paired t-test
##
## data:  DP by Reward_Category
## t = 0.59861, df = 169, p-value = 0.5502
## alternative hypothesis: true mean difference is not equal to 0
## 95 percent confidence interval:
```

```
##  -0.06116513  0.11440279
## sample estimates:
## mean difference
##      0.02661883
```

```r
cohens_dav(data = x3_ph1, x = DP, group = Reward_Category)
```

```
## # A tibble: 2 x 4
##   Reward_Category count  mean    sd
##   <fct>           <int> <dbl> <dbl>
## 1 High Reward       170  1.94 0.728
## 2 Low Reward        170  1.91 0.650
## [1] "Effect size Cohen's d(av):"
## [1] 0.03863648
```

The effect of reward category on memory of items encoded in the pre-conditioning phase was not significant, both with corrected recognition t(169) = -.15, p = .88, $d_{av}$ = -.01, and d-prime t(169) = 0.60, p = .55, $d_{av}$ = .04.

```r
# Effect of reward category on memory in phase 2
# (conditioning)

# Corrected recognition (CR)
t.test(data = x3_ph2, CR ~ Reward_Category, paired = TRUE)
```

```
##
##  Paired t-test
##
## data:  CR by Reward_Category
## t = 1.6088, df = 169, p-value = 0.1095
## alternative hypothesis: true mean difference is not equal to 0
## 95 percent confidence interval:
##  -0.004351765  0.042682477
## sample estimates:
## mean difference
##      0.01916536
```

```r
cohens_dav(data = x3_ph2, x = CR, group = Reward_Category)
```

```
## # A tibble: 2 x 4
##   Reward_Category count  mean    sd
##   <fct>           <int> <dbl> <dbl>
## 1 High Reward       170 0.482 0.192
## 2 Low Reward        170 0.463 0.194
## [1] "Effect size Cohen's d(av):"
## [1] 0.09939281
```

```r
# d-prime (DP)
t.test(data = x3_ph2, DP ~ Reward_Category, paired = TRUE)
```

```
##
##  Paired t-test
##
## data:  DP by Reward_Category
## t = 1.7851, df = 169, p-value = 0.07603
## alternative hypothesis: true mean difference is not equal to 0
## 95 percent confidence interval:
##  -0.009487367  0.188733007
## sample estimates:
## mean difference
##      0.08962282
```

```
cohens_dav(data = x3_ph2, x = DP, group = Reward_Category)
```

```
## # A tibble: 2 x 4
##   Reward_Category count  mean    sd
##   <fct>           <int> <dbl> <dbl>
## 1 High Reward       170  2.15 0.715
## 2 Low Reward        170  2.06 0.753
## [1] "Effect size Cohen's d(av):"
## [1] 0.1221076
```

The effect of reward category on corrected recognition for items encoded in the conditioning phase was not significant: t(169) = 1.61, p = .11, $d_{av}$ = .10, although this was the encoding phase where participants learn the high/low reward associations with items from animal/object categories. The effect was in the expected direction, favoring high-reward category, but still not significant when considering d-prime measures t(169) = 1.79, p = .08, $d_{av}$ = 0.12.

## 1.2 Experiment 3 (High Certainty Memory)

Data from experiment 3 was re-analysed considering only high certainty memory responses. **Corrected recognition (CR) by phase and reward category**

```
# Summary table and graph
aggregate(CR ~ Reward_Category + Phase, data.x3.high, FUN = function(CR) c(mean = mean(CR),
    se = std.error(CR)))
```

```
##   Reward_Category            Phase    CR.mean      CR.se
## 1     High Reward Pre-conditioning 0.55364232 0.01716397
## 2      Low Reward Pre-conditioning 0.55129270 0.01679534
## 3     High Reward     Conditioning 0.59724411 0.01783643
## 4      Low Reward     Conditioning 0.58346862 0.01757964
```

```
x3.high.CR = plot_by_group(data = data.x3.high, yvar = "CR",
    ylim = c(0, 1.2), ylab = "Corrected Recognition", subtitle = "Experiment 3 (High Certainty)",
    tag = "1.2 A")
x3.high.CR
```

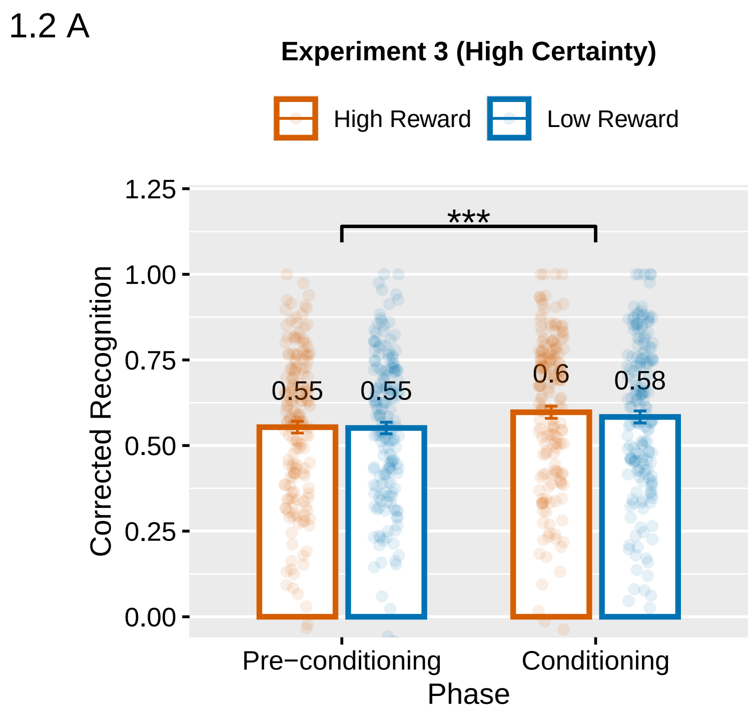


```
# Repeated measures two-factor ANOVA on corrected
# recognition (high certainty only)
anova_test(CR ~ Phase * Reward_Category + Error(UserID/(Phase *
    Reward_Category)), data = data.x3.high)
```

```
## ANOVA Table (type III tests)
##
##                   Effect DFn DFd      F        p p<.05      ges
## 1                  Phase   1 169 13.792 0.000277     * 0.007000
## 2        Reward_Category   1 169  0.443 0.507000       0.000319
## 3 Phase:Reward_Category   1 169  0.562 0.454000       0.000160
```

### d-prime (DP) by phase and reward category

```
# Summary table and graph
aggregate(DP ~ Reward_Category + Phase, data.x3.high, FUN = function(DP) c(mean = mean(DP),
    se = std.error(DP)))
```

```
##   Reward_Category           Phase    DP.mean      DP.se
## 1     High Reward Pre-conditioning 2.55059698 0.07398774
## 2      Low Reward Pre-conditioning 2.53041764 0.07316144
## 3     High Reward     Conditioning 2.73655271 0.08277085
## 4      Low Reward     Conditioning 2.71086189 0.07884899
```

```
x3.high.DP = plot_by_group(data = data.x3.high, yvar = "DP",
    ylim = c(0, 4.6), ylab = "d-prime", subtitle = "Experiment 3 (High Certainty)",
    tag = "1.2 B")
x3.high.DP
```

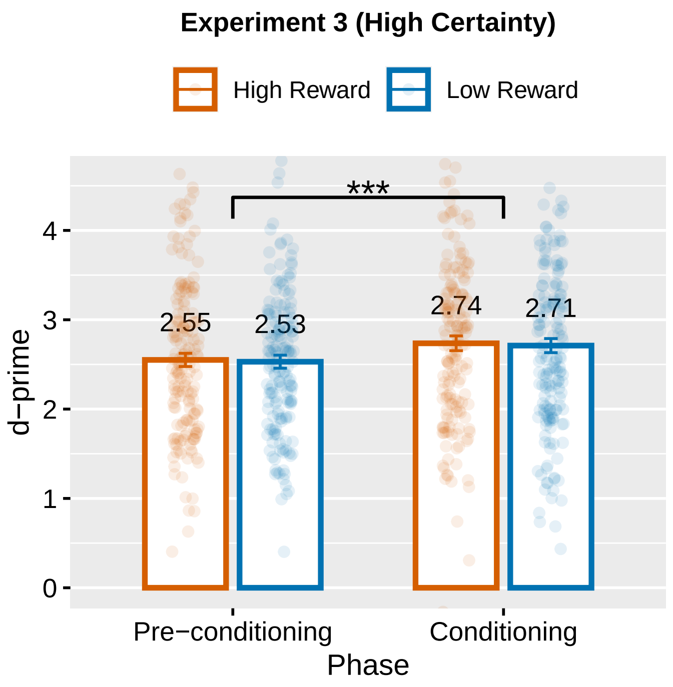


```
# Repeated measures two-factor ANOVA on d-prime scores
# (high certainty only)
anova_test(DP ~ Phase * Reward_Category + Error(UserID/(Phase *
    Reward_Category)), data = data.x3.high)
```

```
## ANOVA Table (type III tests)
##
##                   Effect DFn DFd      F        p p<.05      ges
## 1                  Phase   1 169 17.541 4.52e-05     * 8.00e-03
## 2        Reward_Category   1 169  0.179 6.73e-01       1.30e-04
## 3 Phase:Reward_Category   1 169  0.005 9.41e-01       1.88e-06
```

```
# Create subsets for each phase from data.x3 (high
# certainty)
x3_high_ph1 <- subset(data.x3.high, Phase == "Pre-conditioning")
x3_high_ph2 <- subset(data.x3.high, Phase == "Conditioning")
```

```
# Effect of reward category on high certainty memory in
# phase 1 (pre-conditioning)

# Corrected recognition (CR)
t.test(data = x3_high_ph1, CR ~ Reward_Category, paired = TRUE)
```

```
##
##  Paired t-test
##
## data:  CR by Reward_Category
```

```
## t = 0.16414, df = 169, p-value = 0.8698
## alternative hypothesis: true mean difference is not equal to 0
## 95 percent confidence interval:
##  -0.02590994  0.03060918
## sample estimates:
## mean difference
##     0.002349621
```

```
cohens_dav(data = x3_high_ph1, x = CR, group = Reward_Category)
```

```
## # A tibble: 2 x 4
##   Reward_Category count  mean    sd
##   <fct>           <int> <dbl> <dbl>
## 1 High Reward       170 0.554 0.224
## 2 Low Reward        170 0.551 0.219
## [1] "Effect size Cohen's d(av):"
## [1] 0.01061316
```

```
# d-prime (DP)
t.test(data = x3_high_ph1, DP ~ Reward_Category, paired = TRUE)
```

```
##
##  Paired t-test
##
## data:  DP by Reward_Category
## t = 0.32018, df = 169, p-value = 0.7492
## alternative hypothesis: true mean difference is not equal to 0
## 95 percent confidence interval:
##  -0.1042397  0.1445984
## sample estimates:
## mean difference
##      0.02017933
```

```
cohens_dav(data = x3_high_ph1, x = DP, group = Reward_Category)
```

```
## # A tibble: 2 x 4
##   Reward_Category count  mean    sd
##   <fct>           <int> <dbl> <dbl>
## 1 High Reward       170  2.55 0.965
## 2 Low Reward        170  2.53 0.954
## [1] "Effect size Cohen's d(av):"
## [1] 0.02103558
```

```
# Effect of reward category on high certainty memory in
# phase 2 (conditioning)

# Corrected recognition (CR)
t.test(data = x3_high_ph2, CR ~ Reward_Category, paired = TRUE)
```

```
##
##  Paired t-test
```

```
##
## data:  CR by Reward_Category
## t = 0.96254, df = 169, p-value = 0.3372
## alternative hypothesis: true mean difference is not equal to 0
## 95 percent confidence interval:
##  -0.01447712  0.04202810
## sample estimates:
## mean difference
##      0.01377549
```

```
cohens_dav(data = x3_high_ph2, x = CR, group = Reward_Category)
```

```
## # A tibble: 2 x 4
##   Reward_Category count  mean    sd
##   <fct>           <int> <dbl> <dbl>
## 1 High Reward       170 0.597 0.233
## 2 Low Reward        170 0.583 0.229
## [1] "Effect size Cohen's d(av):"
## [1] 0.05966397
```

```
# d-prime (DP)
t.test(data = x3_high_ph2, DP ~ Reward_Category, paired = TRUE)
```

```
##
##  Paired t-test
##
## data:  DP by Reward_Category
## t = 0.3761, df = 169, p-value = 0.7073
## alternative hypothesis: true mean difference is not equal to 0
## 95 percent confidence interval:
##  -0.1091571  0.1605388
## sample estimates:
## mean difference
##      0.02569082
```

```
cohens_dav(data = x3_high_ph2, x = DP, group = Reward_Category)
```

```
## # A tibble: 2 x 4
##   Reward_Category count  mean    sd
##   <fct>           <int> <dbl> <dbl>
## 1 High Reward       170  2.74  1.08
## 2 Low Reward        170  2.71  1.03
## [1] "Effect size Cohen's d(av):"
## [1] 0.02438309
```

When repeating the analysis with high certainty memory trials, the trend level effect of reward category on item memory in the conditioning phase observed in the analysis with all trails was no longer seen. This was true for both corrected recognition , $t(169) = 0.96$, $p = .34$, $d_{av} = .06$, and d-prime scores, $t(169) = 0.38$, $p = .71$, $d_{av} = .02$. From the ANOVA, the main effect of phase on memory enhancement remained significant, both on corrected recognition $F(1,169) = 13.79$, $p < .001$, $\eta^2 = .007$, and on d-prime scores, $F(1,169) = 17.54$, $p < .001$, $\eta^2 = .008$.

# 2. Complementary Bayesian t-tests

As complementary analysis to classical paired t-tests conducted above, we additionally used Bayesian analysis to confirm whether the data supports the null hypothesis of no effect of reward category on item memory from either encoding phases. We used Bayesian paired t-tests using ttestBF function in R, with the alternative hypothesis (H1) supporting a positive memory effect for high reward items compared to low reward items overall and from each phase, whereas the null hypothesis (H0) represents zero effect [Jarosz and Wiley, 2014, Rouder et al., 2009]

Bayes factors were calculated to test whether the null hypothesis H0 (true effect is equal to zero) holds against the one-sided alternative hypothesis H1 (effect is greater than zero). In the below analysis we used a Cauchy prior distribution with a default scale parameter of r = .707 and interpreted the Bayes factor ($BF_{10}$) as follows:

- $BF_{10} < 1/3$ : Substantial evidence for H0
- $1/3 < BF_{10} < 1$ : Anecdotal evidence for H0
- $1 < BF_{10} < 3$ : Anecdotal evidence for H1
- $BF_{10} > 3$ : Substantial evidence for H1

## 2.1 Experiment 3 (All Memory)

```r
# Effect of reward category on memory in phase 1
# (pre-conditioning) Corrected recognition (CR) Two-sided
# test
ttestBF(x = x3_ph1$CR[x3_ph1$Reward_Category == "High Reward"],
    y = x3_ph1$CR[x3_ph1$Reward_Category == "Low Reward"], paired = TRUE)
```

```
## Bayes factor analysis
## --------------
## [1] Alt., r=0.707 : 0.08650774 ±0.22%
##
## Against denominator:
##   Null, mu = 0
## ---
## Bayes factor type: BFoneSample, JZS
```

```r
# One-sided test
ttestBF(x = x3_ph1$CR[x3_ph1$Reward_Category == "High Reward"],
    y = x3_ph1$CR[x3_ph1$Reward_Category == "Low Reward"], nullInterval = c(-Inf,
        0), paired = TRUE)
```

```
## Bayes factor analysis
## --------------
## [1] Alt., r=0.707 -Inf<d<0    : 0.09670768 ±0%
## [2] Alt., r=0.707 !(-Inf<d<0) : 0.07630779 ±0.05%
##
## Against denominator:
##   Null, mu = 0
## ---
## Bayes factor type: BFoneSample, JZS
```

```r
# d-prime Two-sided test
ttestBF(x = x3_ph1$DP[x3_ph1$Reward_Category == "High Reward"],
    y = x3_ph1$DP[x3_ph1$Reward_Category == "Low Reward"], paired = TRUE)
```

```
## Bayes factor analysis
## --------------
## [1] Alt., r=0.707 : 0.1020266 ±0.19%
##
## Against denominator:
##   Null, mu = 0
## ---
## Bayes factor type: BFoneSample, JZS
```

```r
# One-sided test
ttestBF(x = x3_ph1$DP[x3_ph1$Reward_Category == "High Reward"],
    y = x3_ph1$DP[x3_ph1$Reward_Category == "Low Reward"], nullInterval = c(-Inf,
        0), paired = TRUE)
```

```
## Bayes factor analysis
## --------------
## [1] Alt., r=0.707 -Inf<d<0    : 0.05647724 ±0%
## [2] Alt., r=0.707 !(-Inf<d<0) : 0.1475759  ±0%
##
## Against denominator:
##   Null, mu = 0
## ---
## Bayes factor type: BFoneSample, JZS
```

In the pre-conditioning phase of experiment 3, Bayesian t-tests suggested that data is more probable under the null hypothesis (H0: no effect of reward category on memory) with $BF_{10} = 0.08$ for corrected recognition and $BF_{10} = 0.15$ for d-prime scores. These results are consistent with the findings from classical t-tests.

```r
# Effect of reward category on memory in phase 2
# (conditioning) Corrected recognition (CR) Two-sided test
ttestBF(x = x3_ph2$CR[x3_ph2$Reward_Category == "High Reward"],
    y = x3_ph2$CR[x3_ph2$Reward_Category == "Low Reward"], paired = TRUE)
```

```
## Bayes factor analysis
## --------------
## [1] Alt., r=0.707 : 0.3026351 ±0.07%
##
## Against denominator:
##   Null, mu = 0
## ---
## Bayes factor type: BFoneSample, JZS
```

```r
# One-sided test
ttestBF(x = x3_ph2$CR[x3_ph2$Reward_Category == "High Reward"],
    y = x3_ph2$CR[x3_ph2$Reward_Category == "Low Reward"], nullInterval = c(-Inf,
        0), paired = TRUE)
```

```
## Bayes factor analysis
## --------------
## [1] Alt., r=0.707 -Inf<d<0    : 0.03404228 ±0%
## [2] Alt., r=0.707 !(-Inf<d<0) : 0.5712279  ±0%
##
## Against denominator:
##   Null, mu = 0
## ---
## Bayes factor type: BFoneSample, JZS
```

```
# d-prime Two-sided test
ttestBF(x = x3_ph2$DP[x3_ph2$Reward_Category == "High Reward"],
    y = x3_ph2$DP[x3_ph2$Reward_Category == "Low Reward"], paired = TRUE)
```

```
## Bayes factor analysis
## --------------
## [1] Alt., r=0.707 : 0.4042228 ±0.05%
##
## Against denominator:
##   Null, mu = 0
## ---
## Bayes factor type: BFoneSample, JZS
```

```
# One-sided test
ttestBF(x = x3_ph2$DP[x3_ph2$Reward_Category == "High Reward"],
    y = x3_ph2$DP[x3_ph2$Reward_Category == "Low Reward"], nullInterval = c(-Inf,
        0), paired = TRUE)
```

```
## Bayes factor analysis
## --------------
## [1] Alt., r=0.707 -Inf<d<0    : 0.03172437 ±0%
## [2] Alt., r=0.707 !(-Inf<d<0) : 0.7767212  ±0%
##
## Against denominator:
##   Null, mu = 0
## ---
## Bayes factor type: BFoneSample, JZS
```

In the conditioning phase of experiment 3, Bayesian one-sided t-tests reveal that the data shows anecdotal evidence for the null hypothesis of no effect of reward category on corrected recognition $BF_{10} = 0.57$ for corrected recognition and $BF_{10} = 0.78$ for d-primes.

## 2.2 Experiment 3 (High Certainty Memory)

```
# Effect of reward category on high certainty memory in
# phase 1 (pre-conditioning) Corrected recognition (CR)
# Two-sided test
ttestBF(x = x3_high_ph1$CR[x3_high_ph1$Reward_Category == "High Reward"],
    y = x3_high_ph1$CR[x3_high_ph1$Reward_Category == "Low Reward"],
    paired = TRUE)
```

```
## Bayes factor analysis
## --------------
## [1] Alt., r=0.707 : 0.08669952 ±0.22%
##
## Against denominator:
##   Null, mu = 0
## ---
## Bayes factor type: BFoneSample, JZS
```

```r
# One-sided test
ttestBF(x = x3_high_ph1$CR[x3_high_ph1$Reward_Category == "High Reward"],
    y = x3_high_ph1$CR[x3_high_ph1$Reward_Category == "Low Reward"],
    nullInterval = c(-Inf, 0), paired = TRUE)
```

```
## Bayes factor analysis
## --------------
## [1] Alt., r=0.707 -Inf<d<0    : 0.07550612 ±0.04%
## [2] Alt., r=0.707 !(-Inf<d<0) : 0.09789293 ±0%
##
## Against denominator:
##   Null, mu = 0
## ---
## Bayes factor type: BFoneSample, JZS
```

```r
# d-prime Two-sided test
ttestBF(x = x3_high_ph1$DP[x3_high_ph1$Reward_Category == "High Reward"],
    y = x3_high_ph1$DP[x3_high_ph1$Reward_Category == "Low Reward"],
    paired = TRUE)
```

```
## Bayes factor analysis
## --------------
## [1] Alt., r=0.707 : 0.08998098 ±0.21%
##
## Against denominator:
##   Null, mu = 0
## ---
## Bayes factor type: BFoneSample, JZS
```

```r
# One-sided test
ttestBF(x = x3_high_ph1$DP[x3_high_ph1$Reward_Category == "High Reward"],
    y = x3_high_ph1$DP[x3_high_ph1$Reward_Category == "Low Reward"],
    nullInterval = c(-Inf, 0), paired = TRUE)
```

```
## Bayes factor analysis
## --------------
## [1] Alt., r=0.707 -Inf<d<0    : 0.06759792 ±0.01%
## [2] Alt., r=0.707 !(-Inf<d<0) : 0.1123641  ±0%
##
## Against denominator:
##   Null, mu = 0
## ---
## Bayes factor type: BFoneSample, JZS
```

When considering only high certainty memory, the one-sided Bayesian t-tests still favor the null hypothesis, $BF_{10} = .10$ for corrected recognition and $BF_{10} = .11$ for d-prime scores. This is consistent with results of the frequentist t-tests.

```r
# Effect of reward category on high certainty memory in
# phase 2 (conditioning) Corrected recognition (CR)
# Two-sided test
ttestBF(x = x3_high_ph2$CR[x3_high_ph2$Reward_Category == "High Reward"],
    y = x3_high_ph2$CR[x3_high_ph2$Reward_Category == "Low Reward"],
    paired = TRUE)
```

```
## Bayes factor analysis
## --------------
## [1] Alt., r=0.707 : 0.1347726 ±0.14%
##
## Against denominator:
##   Null, mu = 0
## ---
## Bayes factor type: BFoneSample, JZS
```

```r
# One-sided test
ttestBF(x = x3_high_ph2$CR[x3_high_ph2$Reward_Category == "High Reward"],
    y = x3_high_ph2$CR[x3_high_ph2$Reward_Category == "Low Reward"],
    nullInterval = c(-Inf, 0), paired = TRUE)
```

```
## Bayes factor analysis
## --------------
## [1] Alt., r=0.707 -Inf<d<0    : 0.04598149 ±0%
## [2] Alt., r=0.707 !(-Inf<d<0) : 0.2235638  ±0%
##
## Against denominator:
##   Null, mu = 0
## ---
## Bayes factor type: BFoneSample, JZS
```

```r
# d-prime Two-sided test
ttestBF(x = x3_high_ph2$DP[x3_high_ph2$Reward_Category == "High Reward"],
    y = x3_high_ph2$DP[x3_high_ph2$Reward_Category == "Low Reward"],
    paired = TRUE)
```

```
## Bayes factor analysis
## --------------
## [1] Alt., r=0.707 : 0.09171933 ±0.2%
##
## Against denominator:
##   Null, mu = 0
## ---
## Bayes factor type: BFoneSample, JZS
```

```r
# One-sided test
ttestBF(x = x3_high_ph2$DP[x3_high_ph2$Reward_Category == "High Reward"],
    y = x3_high_ph2$DP[x3_high_ph2$Reward_Category == "Low Reward"],
    nullInterval = c(-Inf, 0), paired = TRUE)
```

```
## Bayes factor analysis
## --------------
## [1] Alt., r=0.707 -Inf<d<0    : 0.0650875 ±0.01%
## [2] Alt., r=0.707 !(-Inf<d<0) : 0.1183512 ±0%
##
## Against denominator:
##   Null, mu = 0
## ---
## Bayes factor type: BFoneSample, JZS
```

With only high certainty memory for the conditioning phase of experiment 3, Bayes factors suggested substantial evidence in favor of the null hypothesis that there is no effect of reward category on memory, $BF_{10} = 0.22$ with corrected recognition and $BF_{10} = 0.12$ with d-prime scores, all Bayes factors $< 0.33$.

# 3. Summary Graphs & Tables

## 3.1 Memory Performance Graphs

```
x3.CR = plot_by_group(data = data.x3, yvar = "CR", ylim = c(0,
    1.2), ylab = "Corrected Recognition", xlab = NULL, subtitle = "All Memory",
    tag = "A")
x3.high.CR = plot_by_group(data = data.x3.high, yvar = "CR",
    ylim = c(0, 1.2), ylab = "Corrected Recognition", xlab = NULL,
    subtitle = "Higher Certainty Memory", tag = "B")
x3.DP = plot_by_group(data = data.x3, yvar = "DP", ylim = c(0,
    4.6), ylab = "d-prime", xlab = NULL, subtitle = "All Memory",
    tag = "C")
x3.high.DP = plot_by_group(data = data.x3.high, yvar = "DP",
    ylim = c(0, 4.6), ylab = "d-prime", xlab = NULL, subtitle = "Higher Certainty Memory",
    tag = "D")

summary.x3 <- ggarrange(x3.CR, x3.high.CR, x3.DP, x3.high.DP,
    ncol = 2, nrow = 2, common.legend = TRUE, legend = "top")
ggsave(file = "summary.x3.svg", plot = summary.x3, width = 8,
    height = 6.5)
ggsave(file = "summary.x3.jpg", plot = summary.x3, width = 8,
    height = 6.5)
```

## 3.2 Memory Performance by Certainty

Create tables to see how memory responses vary by certainty, coded: 0 = definitely old; 12 = likely old; 24 = maybe old; 48 = maybe new; 60 = likely new, 72 = definitely new.

```
data.cert.x3 <- read.csv("adaptiveMemoryReplication/Exp3_CleanData/Supp/x3_Certainty.csv")
ph1_hr <- subset(data.cert.x3, Phase == "1" & Reward_Category ==
    "1") %>%
    group_by(Certainty) %>%
    summarize(mean_size = mean(Proportion, na.rm = TRUE))
ph1_lr <- subset(data.cert.x3, Phase == "1" & Reward_Category ==
    "-1") %>%
```

```
    group_by(Certainty) %>%
    summarize(mean_size = mean(Proportion, na.rm = TRUE))
ph2_hr <- subset(data.cert.x3, Phase == "2" & Reward_Category ==
    "1") %>%
    group_by(Certainty) %>%
    summarize(mean_size = mean(Proportion, na.rm = TRUE))
ph2_lr <- subset(data.cert.x3, Phase == "2" & Reward_Category ==
    "-1") %>%
    group_by(Certainty) %>%
    summarize(mean_size = mean(Proportion, na.rm = TRUE))
new_hr <- subset(data.cert.x3, Phase == "New" & Reward_Category ==
    "1") %>%
    group_by(Certainty) %>%
    summarize(mean_size = mean(Proportion, na.rm = TRUE))
new_lr <- subset(data.cert.x3, Phase == "New" & Reward_Category ==
    "-1") %>%
    group_by(Certainty) %>%
    summarize(mean_size = mean(Proportion, na.rm = TRUE))
```

# 4. Supplementary

## 4.1 Performance on Matching Task

As part of control analyses, performance on the matching tasks were summarised and analysed for any biases between treatment groups. We first tested whether matching accuracy is above chance in each phase of encoding to ascertain participant's attention during encoding. Secondly, we tested whether there were significant differences in matching performance between items from different stimuli categories (animal vs. object) and reward categories (high vs. low).

```
# Matching accuracy above chance in each phase
aggregate(MA ~ Phase, data.x3, FUN = function(MA) c(mean = mean(MA),
    se = std.error(MA)))
```

```
##             Phase     MA.mean       MA.se
## 1 Pre-conditioning 0.871372549 0.005457128
## 2     Conditioning 0.935098039 0.003590152
```

```
# Phase 1 (pre-conditioning)
t.test(data = x3_ph1, mu = 0.5, MA ~ Category, alternative = "two.sided")
```

```
##
##  Welch Two Sample t-test
##
## data:  MA by Category
## t = -45.852, df = 337.14, p-value < 2.2e-16
## alternative hypothesis: true difference in means between group Animal and group Object is not equal t
## 95 percent confidence interval:
##  -0.02267645  0.02032351
## sample estimates:
## mean in group Animal mean in group Object
##            0.8707843            0.8719608
```

```r
# Phase 2 (conditioning)
t.test(data = x3_ph2, mu = 0.5, MA ~ Category, alternative = "two.sided")
```

```
##
##  Welch Two Sample t-test
##
## data:  MA by Category
## t = -71.021, df = 337.82, p-value < 2.2e-16
## alternative hypothesis: true difference in means between group Animal and group Object is not equal t
## 95 percent confidence interval:
##  -0.023520489  0.004696959
## sample estimates:
## mean in group Animal mean in group Object
##            0.9303922            0.9398039
```

Two sample t-tests showed that matching accuracy was well over chance level (0.5) in both phases, P values < .001, and thus suggested that participants were paying attention in both encoding phases.

Next we conducted paired t-tests and ANOVA to test whether matching accuracy in each phase varied by stimuli category (animal vs. objects), and whether matching accuracy for items from the conditioning phase varied with reward category (high vs. low reward).

```r
# Matching accuracy by categories (animal vs. objects)
aggregate(MA ~ Category + Phase, data.x3, FUN = function(MA) c(mean = mean(MA),
    se = std.error(MA)))
```

```
##   Category            Phase     MA.mean       MA.se
## 1   Animal Pre-conditioning 0.870784314 0.007530747
## 2   Object Pre-conditioning 0.871960784 0.007921941
## 3   Animal     Conditioning 0.930392157 0.005012559
## 4   Object     Conditioning 0.939803922 0.005130445
```

```r
# Phase 1 (pre-conditioning)
t.test(data = x3_ph1, MA ~ Category, paired = TRUE)
```

```
##
##  Paired t-test
##
## data:  MA by Category
## t = -0.18752, df = 169, p-value = 0.8515
## alternative hypothesis: true mean difference is not equal to 0
## 95 percent confidence interval:
##  -0.01356184  0.01120890
## sample estimates:
## mean difference
##    -0.001176471
```

```r
cohens_dav(data = x3_ph1, x = MA, group = Category)
```

```
## # A tibble: 2 x 4
##   Category count  mean     sd
```

```
##   <chr>    <int> <dbl>  <dbl>
## 1 Animal     170 0.871 0.0982
## 2 Object     170 0.872 0.103
## [1] "Effect size Cohen's d(av):"
## [1] -0.01167838
```

```
# Phase 2 (conditioning)
t.test(data = x3_ph2, MA ~ Category, paired = TRUE)
```

```
##
##  Paired t-test
##
## data:  MA by Category
## t = -1.9899, df = 169, p-value = 0.04822
## alternative hypothesis: true mean difference is not equal to 0
## 95 percent confidence interval:
##  -1.874886e-02 -7.466519e-05
## sample estimates:
## mean difference
##    -0.009411765
```

```
cohens_dav(data = x3_ph2, x = MA, group = Category)
```

```
## # A tibble: 2 x 4
##   Category count  mean     sd
##   <chr>    <int> <dbl>  <dbl>
## 1 Animal     170 0.930 0.0654
## 2 Object     170 0.940 0.0669
## [1] "Effect size Cohen's d(av):"
## [1] -0.1423344
```

```
# Repeated measures ANOVA on memory by phase and category
anova_test(MA ~ Phase * Category + Error(UserID/(Phase * Category)),
    data = data.x3)
```

```
## ANOVA Table (type III tests)
##
##           Effect DFn DFd       F        p p<.05      ges
## 1          Phase   1 169 107.456 8.47e-20     * 0.123000
## 2       Category   1 169   2.001 1.59e-01       0.000969
## 3 Phase:Category   1 169   1.006 3.17e-01       0.000587
```

We find that in the conditioning phase (phase 2) of experiment 3, at trend level, matching accuracy is higher for stimuli in the 'Object' category, t(169) = -1.99, p = .05, $d_{av}$ = -.14. The ANOVA also showed a non-significant main effect of category, F(1,169) = 2.00, p = .16, $\eta^2$ = .001. Since this effect is not strongly significant, and the study design being counter-balanced when allocating Object/Animal as the highly rewarded category, this effect should not have interfered with memory effects.

Furthermore, we check how matching accuracy and reaction time in the conditioning phase varies by reward category (high vs. low).

```r
# Matching accuracy by reward category (high vs. low) Phase
# 2 (conditioning)
t.test(data = x3_ph2, MA ~ Reward_Category, paired = TRUE)
```

```
##
##  Paired t-test
##
## data:  MA by Reward_Category
## t = 0.24592, df = 169, p-value = 0.806
## alternative hypothesis: true mean difference is not equal to 0
## 95 percent confidence interval:
##  -0.00826769  0.01062063
## sample estimates:
## mean difference
##     0.001176471
```

```r
cohens_dav(data = x3_ph2, x = MA, group = Reward_Category)
```

```
## # A tibble: 2 x 4
##   Reward_Category count  mean     sd
##   <fct>           <int> <dbl>  <dbl>
## 1 High Reward       170 0.936 0.0662
## 2 Low Reward        170 0.935 0.0664
## [1] "Effect size Cohen's d(av):"
## [1] 0.01774617
```

Matching accuracy did not significantly differ between item reward categories (high vs. low), thus suggesting that equal attention was paid to all items, regardless of reward category, during encoding.

```r
# Repeated measures ANOVA on matching accuracy by phase and
# reward category
anova_test(MA ~ Phase * Reward_Category + Error(UserID/(Phase *
    Reward_Category)), data = data.x3)
```

```
## ANOVA Table (type III tests)
##
##                  Effect DFn DFd       F        p p<.05      ges
## 1                 Phase   1 169 107.456 8.47e-20     * 1.23e-01
## 2       Reward_Category   1 169   0.003 9.59e-01       1.33e-06
## 3 Phase:Reward_Category   1 169   0.057 8.12e-01       3.32e-05
```

```r
# Repeated measures ANOVA on reaction time by phase and
# reward category
anova_test(RT ~ Phase * Reward_Category + Error(UserID/(Phase *
    Reward_Category)), data = data.x3)
```

```
## ANOVA Table (type III tests)
##
##                  Effect DFn DFd       F        p p<.05      ges
## 1                 Phase   1 169 123.575 6.77e-22     * 1.06e-01
## 2       Reward_Category   1 169   0.105 7.46e-01       1.85e-05
## 3 Phase:Reward_Category   1 169   1.108 2.94e-01       1.93e-04
```

```r
# Graph: matching accuracy by phase and category
x3.MA = plot_by_group_cat(data = data.x3, yvar = "MA", ylim = c(0,
    1.2), ylab = "Matching Accuracy", subtitle = "Experiment 3",
    lab.vjust = 2.5)
ggsave(file = "x3.MA.svg", plot = x3.MA, width = 10, height = 10,
    units = "cm")
x3.MA
```

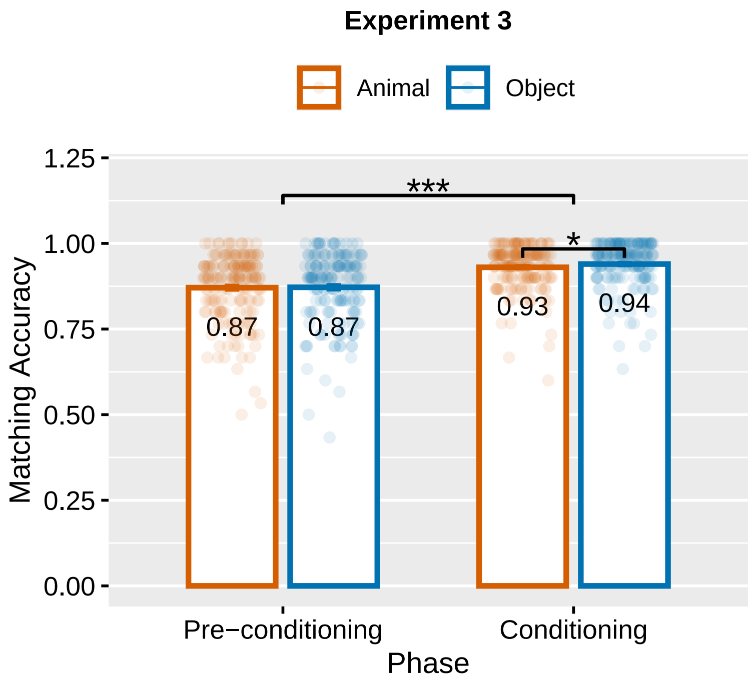


```r
# Graph: matching accuracy by phase and reward category
x3.MA = plot_by_group(data = data.x3, yvar = "MA", ylim = c(0,
    1.2), ylab = "Matching Accuracy", subtitle = "Experiment 3",
    lab.vjust = 2.5)
ggsave(file = "x3.MA.svg", plot = x3.MA, width = 10, height = 10,
    units = "cm")
x3.MA
```

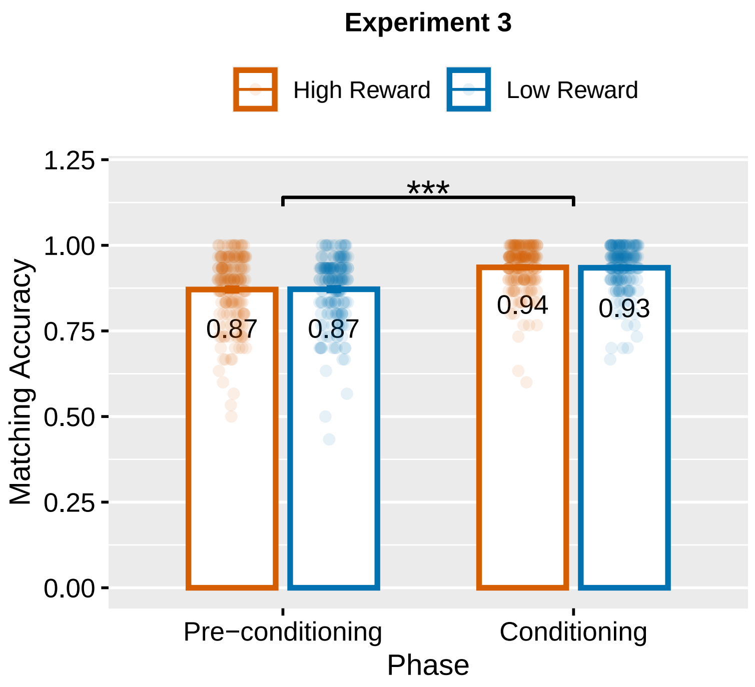


```
# Graph: matching reaction time by phase and reward
# category
x3.RT = plot_by_group(data = data.x3, yvar = "RT", ylim = c(0,
    600), ylab = "Reaction Time (ms)", subtitle = "Experiment 3",
    lab.sf = 0, lab.vjust = 2.5)
ggsave(file = "x3.RT.svg", plot = x3.RT, width = 10, height = 10,
    units = "cm")
x3.RT
```

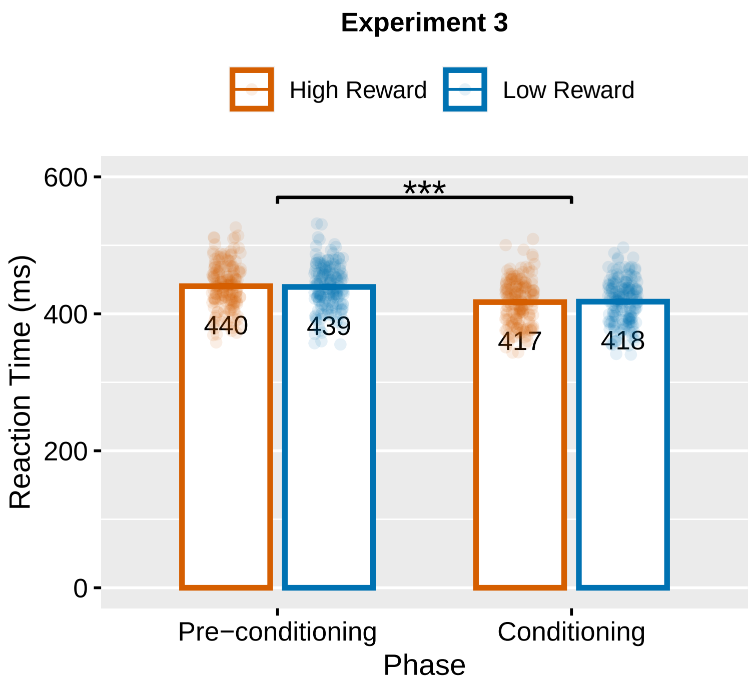


## 4.2 Exclusion Based on Surprisal

One way in which our study differed from the original study is that participants who were not surprised by the memory test were excluded from all analysis. Here we show that this does not significantly change the pattern of our main results by repeating the analysis after excluding participants who were not surprised. After exclusions, experiment 3 had N=104.

The ANOVAs revealed significant effects and trends of encoding phase, and a weak interaction effect between the encoding phase and reward category that the item belonged to. Furthermore, as found in the analysis prior to exclusions, t-tests revealed a trend level effect of reward category in the conditioning phase when considering all memory trials in experiment 3, $0.05 < p < 0.1$, which did not hold when considering high certainty memory trials. There were no further effects of reward category on memory, with both corrected recognition and d-primes, for items encoded in the pre-conditioning phase of the experiment.

```
# Participants to exclude as they anticipated the memory
# test
data.x3.exclude <- read.csv("adaptiveMemoryReplication/Exp3_CleanData/Supp/x3_Surprisal_Exclude.csv")
x3.exclude <- subset(data.x3.exclude, Exclude.Surprise == 1,
    UserID)

# Data subset (not surprised)
data.x3.ns <- data.x3[!data.x3$UserID %in% x3.exclude$UserID,
    ]
data.x3.high.ns <- data.x3.high[!data.x3.high$UserID %in% x3.exclude$UserID,
    ]

# Change phase labels for readability
data.x3.ns$Phase[data.x3.ns$Phase == "Ph1"] <- "Pre-conditioning"
```

```r
data.x3.ns$Phase[data.x3.ns$Phase == "Ph2"] <- "Conditioning"
data.x3.high.ns$Phase[data.x3.high.ns$Phase == "Ph1"] <- "Pre-conditioning"
data.x3.high.ns$Phase[data.x3.high.ns$Phase == "Ph2"] <- "Conditioning"
# Reorder variables
data.x3.ns$Reward_Category <- factor(data.x3.ns$Reward_Category,
    levels = c("High Reward", "Low Reward"))
data.x3.ns$Phase <- factor(data.x3.ns$Phase, levels = c("Pre-conditioning",
    "Conditioning"))
data.x3.high.ns$Reward_Category <- factor(data.x3.high.ns$Reward_Category,
    levels = c("High Reward", "Low Reward"))
data.x3.high.ns$Phase <- factor(data.x3.high.ns$Phase, levels = c("Pre-conditioning",
    "Conditioning"))
```

### Experiment 3 (All Memory)

```r
# Repeated measures two-factor ANOVA on corrected
# recognition (all memory) Corrected recognition (CR)
anova_test(CR ~ Phase * Reward_Category + Error(UserID/(Phase *
    Reward_Category)), data = data.x3.ns)
```

```
## ANOVA Table (type III tests)
##
##                  Effect DFn DFd      F       p p<.05      ges
## 1                 Phase   1 103 16.172 0.00011     * 0.014000
## 2       Reward_Category   1 103  0.471 0.49400       0.000527
## 3 Phase:Reward_Category   1 103  3.590 0.06100       0.001000
```

```r
# d-prime (DP)
anova_test(DP ~ Phase * Reward_Category + Error(UserID/(Phase *
    Reward_Category)), data = data.x3.ns)
```

```
## ANOVA Table (type III tests)
##
##                  Effect DFn DFd      F        p p<.05   ges
## 1                 Phase   1 103 17.238 6.81e-05     * 0.014
## 2       Reward_Category   1 103  1.016 3.16e-01       0.001
## 3 Phase:Reward_Category   1 103  4.614 3.40e-02     * 0.002
```

```r
# Create subsets for each phase from data.x3
x3_ns_ph1 <- subset(data.x3.ns, Phase == "Pre-conditioning")
x3_ns_ph2 <- subset(data.x3.ns, Phase == "Conditioning")
```

```r
# Effect of reward category on high certainty memory in
# phase 1 (pre-conditioning) Corrected recognition (CR)
t.test(data = x3_ns_ph1, CR ~ Reward_Category, paired = TRUE)
```

```
##
##  Paired t-test
##
```

```
## data:  CR by Reward_Category
## t = -0.36092, df = 103, p-value = 0.7189
## alternative hypothesis: true mean difference is not equal to 0
## 95 percent confidence interval:
##  -0.03275681  0.02267013
## sample estimates:
## mean difference
##    -0.005043342
```

```
cohens_dav(data = x3_ns_ph1, x = CR, group = Reward_Category)
```

```
## # A tibble: 2 x 4
##   Reward_Category count  mean    sd
##   <fct>           <int> <dbl> <dbl>
## 1 High Reward       104 0.412 0.198
## 2 Low Reward        104 0.418 0.178
## [1] "Effect size Cohen's d(av):"
## [1] -0.02688121
```

```
# d-prime (DP)
t.test(data = x3_ns_ph1, DP ~ Reward_Category, paired = TRUE)
```

```
##
##  Paired t-test
##
## data:  DP by Reward_Category
## t = -0.13495, df = 103, p-value = 0.8929
## alternative hypothesis: true mean difference is not equal to 0
## 95 percent confidence interval:
##  -0.10977641  0.09578903
## sample estimates:
## mean difference
##    -0.006993691
```

```
cohens_dav(data = x3_ns_ph1, x = DP, group = Reward_Category)
```

```
## # A tibble: 2 x 4
##   Reward_Category count  mean    sd
##   <fct>           <int> <dbl> <dbl>
## 1 High Reward       104  1.88 0.741
## 2 Low Reward        104  1.89 0.604
## [1] "Effect size Cohen's d(av):"
## [1] -0.01040274
```

```
# Effect of reward category on high certainty memory in
# phase 2 (conditioning) Corrected recognition (CR)
t.test(data = x3_ns_ph2, CR ~ Reward_Category, paired = TRUE)
```

```
##
##  Paired t-test
##
```

```
## data:  CR by Reward_Category
## t = 1.4748, df = 103, p-value = 0.1433
## alternative hypothesis: true mean difference is not equal to 0
## 95 percent confidence interval:
##  -0.007741596  0.052653913
## sample estimates:
## mean difference
##      0.02245616
```

```
cohens_dav(data = x3_ns_ph2, x = CR, group = Reward_Category)
```

```
## # A tibble: 2 x 4
##   Reward_Category count  mean    sd
##   <fct>           <int> <dbl> <dbl>
## 1 High Reward       104 0.471 0.199
## 2 Low Reward        104 0.448 0.187
## [1] "Effect size Cohen's d(av):"
## [1] 0.1163175
```

```
# d-prime (DP)
t.test(data = x3_ns_ph2, DP ~ Reward_Category, paired = TRUE)
```

```
##
##  Paired t-test
##
## data:  DP by Reward_Category
## t = 1.7788, df = 103, p-value = 0.07823
## alternative hypothesis: true mean difference is not equal to 0
## 95 percent confidence interval:
##  -0.01225203  0.22537743
## sample estimates:
## mean difference
##       0.1065627
```

```
cohens_dav(data = x3_ns_ph2, x = DP, group = Reward_Category)
```

```
## # A tibble: 2 x 4
##   Reward_Category count  mean    sd
##   <fct>           <int> <dbl> <dbl>
## 1 High Reward       104  2.10 0.748
## 2 Low Reward        104  1.99 0.665
## [1] "Effect size Cohen's d(av):"
## [1] 0.1507386
```

### Experiment 3 (High Certainty Memory)

```
# Repeated measures two-factor ANOVA on corrected
# recognition (high certainty only) Corrected recognition
# (CR)
anova_test(CR ~ Phase * Reward_Category + Error(UserID/(Phase *
    Reward_Category)), data = data.x3.high.ns)
```

```
## ANOVA Table (type III tests)
##
##                   Effect DFn DFd     F     p p<.05      ges
## 1                  Phase   1 103 6.219 0.014     * 0.005000
## 2        Reward_Category   1 103 0.976 0.326       0.001000
## 3 Phase:Reward_Category   1 103 0.673 0.414       0.000336
```

```
# d-prime (DP)
anova_test(DP ~ Phase * Reward_Category + Error(UserID/(Phase *
    Reward_Category)), data = data.x3.high.ns)
```

```
## ANOVA Table (type III tests)
##
##                   Effect DFn DFd     F     p p<.05      ges
## 1                  Phase   1 103 7.456 0.007     * 0.006000
## 2        Reward_Category   1 103 0.312 0.577       0.000314
## 3 Phase:Reward_Category   1 103 0.265 0.608       0.000150
```

```
# Create subsets for each phase from data.x3
x3_high_ns_ph1 <- subset(data.x3.high.ns, Phase == "Pre-conditioning")
x3_high_ns_ph2 <- subset(data.x3.high.ns, Phase == "Conditioning")
```

```
# Effect of reward category on high certainty memory in
# phase 1 (pre-conditioning) Corrected recognition (CR)
t.test(data = x3_high_ns_ph1, CR ~ Reward_Category, paired = TRUE)
```

```
##
##  Paired t-test
##
## data:  CR by Reward_Category
## t = 0.38243, df = 103, p-value = 0.7029
## alternative hypothesis: true mean difference is not equal to 0
## 95 percent confidence interval:
##  -0.02758951  0.04077162
## sample estimates:
## mean difference
##     0.006591053
```

```
cohens_dav(data = x3_high_ns_ph1, x = CR, group = Reward_Category)
```

```
## # A tibble: 2 x 4
##   Reward_Category count  mean    sd
##   <fct>           <int> <dbl> <dbl>
## 1 High Reward       104 0.548 0.227
## 2 Low Reward        104 0.541 0.220
## [1] "Effect size Cohen's d(av):"
## [1] 0.02948757
```

```
# d-prime (DP)
t.test(data = x3_high_ns_ph1, DP ~ Reward_Category, paired = TRUE)
```

```
##
##  Paired t-test
##
## data:  DP by Reward_Category
## t = 0.15254, df = 103, p-value = 0.8791
## alternative hypothesis: true mean difference is not equal to 0
## 95 percent confidence interval:
##  -0.1280695  0.1494111
## sample estimates:
## mean difference
##      0.01067084
```

```
cohens_dav(data = x3_high_ns_ph1, x = DP, group = Reward_Category)
```

```
## # A tibble: 2 x 4
##   Reward_Category count  mean    sd
##   <fct>           <int> <dbl> <dbl>
## 1 High Reward       104  2.51 0.957
## 2 Low Reward        104  2.50 0.883
## [1] "Effect size Cohen's d(av):"
## [1] 0.01159796
```

```
# Effect of reward category on high certainty memory in
# phase 2 (conditioning) Corrected recognition (CR)
t.test(data = x3_high_ns_ph2, CR ~ Reward_Category, paired = TRUE)
```

```
##
##  Paired t-test
##
## data:  CR by Reward_Category
## t = 1.2165, df = 103, p-value = 0.2266
## alternative hypothesis: true mean difference is not equal to 0
## 95 percent confidence interval:
##  -0.01465142  0.06114605
## sample estimates:
## mean difference
##      0.02324731
```

```
cohens_dav(data = x3_high_ns_ph2, x = CR, group = Reward_Category)
```

```
## # A tibble: 2 x 4
##   Reward_Category count  mean    sd
##   <fct>           <int> <dbl> <dbl>
## 1 High Reward       104 0.589 0.234
## 2 Low Reward        104 0.566 0.231
## [1] "Effect size Cohen's d(av):"
## [1] 0.0999259
```

```
# d-prime (DP)
t.test(data = x3_high_ns_ph2, DP ~ Reward_Category, paired = TRUE)
```

```
##
##  Paired t-test
##
## data:  DP by Reward_Category
## t = 0.69683, df = 103, p-value = 0.4875
## alternative hypothesis: true mean difference is not equal to 0
## 95 percent confidence interval:
##  -0.1076066  0.2241815
## sample estimates:
## mean difference
##      0.05828747
```

```
cohens_dav(data = x3_high_ns_ph2, x = DP, group = Reward_Category)
```

```
## # A tibble: 2 x 4
##   Reward_Category count  mean    sd
##   <fct>           <int> <dbl> <dbl>
## 1 High Reward       104  2.69  1.06
## 2 Low Reward        104  2.63  1.00
## [1] "Effect size Cohen's d(av):"
## [1] 0.0564759
```

### Summary Graphs

```
x3.CR.ns = plot_by_group(data = data.x3.ns, yvar = "CR", ylim = c(0,
    1.2), ylab = "Corrected Recognition", xlab = NULL, subtitle = "All Memory",
    tag = "A")
x3.high.CR.ns = plot_by_group(data = data.x3.high.ns, yvar = "CR",
    ylim = c(0, 1.2), ylab = "Corrected Recognition", xlab = NULL,
    subtitle = "High Certainty Memory", tag = "B")
x3.DP.ns = plot_by_group(data = data.x3.ns, yvar = "DP", ylim = c(0,
    4.6), ylab = "d-prime", xlab = NULL, subtitle = "All Memory",
    tag = "C")
x3.high.DP.ns = plot_by_group(data = data.x3.high.ns, yvar = "DP",
    ylim = c(0, 4.6), ylab = "d-prime", xlab = NULL, subtitle = "High Certainty Memory",
    tag = "D")

summary.x3.ns <- ggarrange(x3.CR.ns, x3.high.CR.ns, x3.DP.ns,
    x3.high.DP.ns, ncol = 2, nrow = 2, common.legend = TRUE,
    legend = "top")
# summary.x3 <- annotate_figure(summary.x3, top =
# text_grob('Experiment 3', face = 'bold', size = 12))
ggsave(file = "summary.x3.ns.svg", plot = summary.x3.ns, width = 8,
    height = 6.5)
ggsave(file = "summary.x3.ns.jpg", plot = summary.x3.ns, width = 8,
    height = 6.5)
```

## 4.3 Comparison of Response Biases

Response bias, calculated as per signal detection theory, was calculated for trials in each phase and reward category. We used paired t-tests to check if there were significant differences between response biases for

items in high vs. low reward categories in either encoding phases which could have influenced our results. The analysis below did not reveal any such effects.

### Experiment 3 (All Memory)

```r
# Effect of reward category on response bias in phase 1
# (pre-conditioning)
t.test(data = x3_ph1, RB ~ Reward_Category, paired = TRUE)
```

```
##
##  Paired t-test
##
## data:  RB by Reward_Category
## t = 0.020269, df = 169, p-value = 0.9839
## alternative hypothesis: true mean difference is not equal to 0
## 95 percent confidence interval:
##  -0.05993920  0.06118281
## sample estimates:
## mean difference
##    0.0006218036
```

```r
cohens_dav(data = x3_ph1, x = RB, group = Reward_Category)
```

```
## # A tibble: 2 x 4
##   Reward_Category count  mean    sd
##   <fct>           <int> <dbl> <dbl>
## 1 High Reward       170 0.215 0.422
## 2 Low Reward        170 0.215 0.389
## [1] "Effect size Cohen's d(av):"
## [1] 0.001534527
```

```r
# Effect of reward category on response bias in phase 2
# (conditioning)
t.test(data = x3_ph2, RB ~ Reward_Category, paired = TRUE)
```

```
##
##  Paired t-test
##
## data:  RB by Reward_Category
## t = -0.81479, df = 169, p-value = 0.4163
## alternative hypothesis: true mean difference is not equal to 0
## 95 percent confidence interval:
##  -0.08832931  0.03671761
## sample estimates:
## mean difference
##     -0.02580585
```

```r
cohens_dav(data = x3_ph2, x = RB, group = Reward_Category)
```

```
## # A tibble: 2 x 4
##   Reward_Category count  mean    sd
##   <fct>           <int> <dbl> <dbl>
## 1 High Reward       170 0.120 0.430
## 2 Low Reward        170 0.145 0.402
## [1] "Effect size Cohen's d(av):"
## [1] -0.06203619
```

### Experiment 3 (High Certainty Memory)

```
# Effect of reward category on response bias in phase 1
# (pre-conditioning)
t.test(data = x3_high_ph1, RB ~ Reward_Category, paired = TRUE)
```

```
##
##  Paired t-test
##
## data:  RB by Reward_Category
## t = -0.87698, df = 169, p-value = 0.3817
## alternative hypothesis: true mean difference is not equal to 0
## 95 percent confidence interval:
##  -0.11999645  0.04617563
## sample estimates:
## mean difference
##     -0.03691041
```

```
cohens_dav(data = x3_high_ph1, x = RB, group = Reward_Category)
```

```
## # A tibble: 2 x 4
##   Reward_Category count  mean    sd
##   <fct>           <int> <dbl> <dbl>
## 1 High Reward       170 0.123 0.564
## 2 Low Reward        170 0.160 0.554
## [1] "Effect size Cohen's d(av):"
## [1] -0.0659973
```

```
# Effect of reward category on response bias in phase 2
# (conditioning)
t.test(data = x3_high_ph2, RB ~ Reward_Category, paired = TRUE)
```

```
##
##  Paired t-test
##
## data:  RB by Reward_Category
## t = -1.2049, df = 169, p-value = 0.2299
## alternative hypothesis: true mean difference is not equal to 0
## 95 percent confidence interval:
##  -0.12824949  0.03102977
## sample estimates:
## mean difference
##     -0.04860986
```

```r
cohens_dav(data = x3_high_ph2, x = RB, group = Reward_Category)
```

```
## # A tibble: 2 x 4
##   Reward_Category count   mean    sd
##   <fct>           <int>  <dbl> <dbl>
## 1 High Reward       170 0.0326 0.543
## 2 Low Reward        170 0.0812 0.563
```

# Experiment 3 Linear Model

```
# Load necessary packages
library(dplyr)
library(tidyverse)
library(rstatix)
library(svglite)
library(rlang)
library(plotrix)
library(gridExtra)
library(tinytex)
library(formatR)
library(knitr)
library(lme4)
```

**This section contains linear mixed model analyses and results for Experiment 3.**

## Data loading

```
# Load Experiment 3 data
data.x3 <- read.csv("adaptiveMemoryReplication/Exp3_CleanData/Main/x3_Regression.csv")  # all trial dat

# Filter to create dataset with only high certainty memory
# trials
data.high.x3 <- subset(data.x3, Certainty == 0 | Certainty ==
    12 | Certainty == 60 | Certainty == 72)

# Insert Say_Old column based on memory responses Trials
# where participants were too slow are omitted (taken as
# NA)
data.x3 <- data.x3 %>%
    mutate(Say_Old = NA, Say_Old = ifelse(Certainty == 0, 1,
        Say_Old), Say_Old = ifelse(Certainty == 12, 1, Say_Old),
        Say_Old = ifelse(Certainty == 24, 1, Say_Old), Say_Old = ifelse(Certainty ==
            48, 0, Say_Old), Say_Old = ifelse(Certainty == 60,
            0, Say_Old), Say_Old = ifelse(Certainty == 72, 0,
            Say_Old))

data.high.x3 <- data.high.x3 %>%
    mutate(Say_Old = NA, Say_Old = ifelse(Certainty == 0, 1,
        Say_Old), Say_Old = ifelse(Certainty == 12, 1, Say_Old),
        Say_Old = ifelse(Certainty == 60, 0, Say_Old), Say_Old = ifelse(Certainty ==
            72, 0, Say_Old))
```

```
data.x3 <- data.x3[!is.na(data.x3$Say_Old), ]
data.high.x3 <- data.high.x3[!is.na(data.high.x3$Say_Old), ]
```

## Prepare data for regression

```
# Prepare coded and factored data for regression analysis
data.x3 <- data.x3 %>%
    mutate(Phase = replace(Phase, Phase == "Rec_Memory", 0),
        Reward_Category = replace(Reward_Category, Reward_Category ==
            -1, 0))

data.high.x3 <- data.high.x3 %>%
    mutate(Phase = replace(Phase, Phase == "Rec_Memory", 0),
        Reward_Category = replace(Reward_Category, Reward_Category ==
            -1, 0))
```

## Data format

**Datasets:** Trial by trial summary of performance on the matching and memory tasks for all participants.

**Data variables:**

1. UserID: unique user identification
2. Rew_Subgroup: allocation of stimuli category to high reward ("Reward_Animals", "Reward_Objects")
3. Category: stimuli category ("Animal", "Object")
4. Reward_Category: stimuli reward category ("1":High Reward, "0":Low Reward)
5. Phase: phase in which stimuli was encoded ("0":New Items, 1":Pre-conditioning, "2":Conditioning)
6. Memory_RT: memory trial reaction time in ms
7. Memory_Correct: memory trial ("1" correct, "0" wrong)
8. Match_RT: matching trial reaction time in ms
9. Match_Correct: matching trial ("1":correct, "0":wrong)
10. Stim: word describing the stimuli image

Further unused variables: 11. Sex 12. Age 13. Stim_Type: ("old_img", "new_img") 14. Certainty: memory trial certainty response ("0":definitely old, "12":likely old, "24":maybe old, "48":maybe new, "60":likley new, "72":definitely new)

## 1. Main Analysis (LM Model)

As another complementary analysis of the effects of reward category on recognition memory performance across phases, we estimated generalized linear mixed-effect models (GLMMs) with a logit-link function using the lme4 R package (Bates et al., 2015). The dependent variable (Say_Old) was participants' categorical response to the memory test collapsed across response certainty with responding old (Say_Old = 1) or responding new (Say_Old = 0). We included main effects of reward category, with high reward category (Reward_Category = 1) and low reward category (Reward_Category = 0) and encoding phase for which we used dummy coding. New items (Phase = 0) were taken as the reference category for the other two phases

(Phase = 1, Phase = 2). In terms of random effects, we first ran models with random intercepts for each participant (UserID) and stimuli item (Stim). Note that adding random slopes for each predictor did not result in model convergence, thus we omit this from our models and only retain random intercepts.

Confidence intervals were calculated using the confint function with bootstrapping method. Instead of relying on Wald's method obtained from the summary() function, we have used bootMer function calculate bootstrapped parametric p-values. For each fixed effect, we calculated the proportion of estimates > 0 (when beta is negative) or < 0 (when beta is positive) and output a p-value based on this.

```
# Set number of iterations for bootstrapping
Nsim = 100
```

## 1.1 Experiment 3 (All Memory)

```
glm1.1 <- glmer(Say_Old ~ 1 + Phase * Reward_Category + (1 |
    UserID) + (1 | Stim), family = binomial(link = "logit"),
    data = data.x3, glmerControl(optimizer = "bobyqa"))
summary(glm1.1)
```

```
## Generalized linear mixed model fit by maximum likelihood (Laplace
##   Approximation) [glmerMod]
##  Family: binomial  ( logit )
## Formula: Say_Old ~ 1 + Phase * Reward_Category + (1 | UserID) + (1 | Stim)
##    Data: data.x3
## Control: glmerControl(optimizer = "bobyqa")
##
##      AIC      BIC   logLik deviance df.resid
##  43275.8  43344.6 -21629.9  43259.8    40434
##
## Scaled residuals:
##     Min      1Q  Median      3Q     Max
## -5.4921 -0.6008 -0.2792  0.6657  6.7468
##
## Random effects:
##  Groups Name        Variance Std.Dev.
##  Stim   (Intercept) 0.4180   0.6465
##  UserID (Intercept) 0.3112   0.5578
## Number of obs: 40442, groups:  Stim, 240; UserID, 170
##
## Fixed effects:
##                        Estimate Std. Error z value Pr(>|z|)
## (Intercept)            -1.48227    0.07767 -19.083   <2e-16 ***
## Phase1                  2.16492    0.09316  23.239   <2e-16 ***
## Phase2                  2.37570    0.09353  25.401   <2e-16 ***
## Reward_Category         0.02022    0.03617   0.559   0.5762
## Phase1:Reward_Category -0.01878    0.05698  -0.330   0.7418
## Phase2:Reward_Category  0.09860    0.05829   1.691   0.0908 .
## ---
## Signif. codes:  0 '***' 0.001 '**' 0.01 '*' 0.05 '.' 0.1 ' ' 1
##
## Correlation of Fixed Effects:
##               (Intr) Phase1 Phase2 Rwrd_C P1:R_C
```

```
## Phase1      -0.582
## Phase2      -0.580  0.885
## Rewrd_Ctgry -0.234  0.195  0.195
## Phs1:Rwrd_C  0.149 -0.307 -0.123 -0.635
## Phs2:Rwrd_C  0.145 -0.120 -0.308 -0.621  0.393
```

```
# confint.1.1 <- confint.merMod(glm1.1, method = 'boot',
# nsim = Nsim, parallel = 'multicore',ncpus = 4) pvals.1.1
# <- bootMer(glm1.1, FUN = fixef, nsim = Nsim, parallel =
# 'multicore', ncpus = 4) # saveRDS(confint.1.1,
# 'x3_confs/confint.1.1.rds') saveRDS(pvals.1.1,
# 'x3_confs/pvals.1.1.rds')
```

```
# load previously run results
confint.1.1 <- readRDS(file = "x3_confs/confint.1.1.rds")
confint.1.1
```

```
##                              2.5 %     97.5 %
## .sig01                  0.57353567  0.7053134
## .sig02                  0.49557542  0.6240707
## (Intercept)            -1.62932471 -1.3305619
## Phase1                  1.97472767  2.3279839
## Phase2                  2.17039637  2.5249676
## Reward_Category        -0.04750715  0.1020317
## Phase1:Reward_Category -0.11920351  0.1108569
## Phase2:Reward_Category -0.01397678  0.2183069
```

```
pvals.1.1 <- readRDS(file = "x3_confs/pvals.1.1.rds")
pvals.1.1
```

```
##
## PARAMETRIC BOOTSTRAP
##
##
## Call:
## bootMer(x = glm1.1, FUN = fixef, nsim = Nsim, parallel = "multicore",
##     ncpus = 4)
##
##
## Bootstrap Statistics :
##        original        bias    std. error
## t1* -1.48226038 -0.009885785  0.07539133
## t2*  2.16490931  0.011603256  0.09241308
## t3*  2.37569568  0.008605226  0.09650935
## t4*  0.02021834 -0.004604331  0.03043500
## t5* -0.01877694  0.005961305  0.04949747
## t6*  0.09859654  0.004396885  0.04317947
```

```
pvals.1.1.list <- mean(pvals.1.1$t[, 1] > 0) * 2
pvals.1.1.list[2] <- mean(pvals.1.1$t[, 2] < 0) * 2
pvals.1.1.list[3] <- mean(pvals.1.1$t[, 3] < 0) * 2
pvals.1.1.list[4] <- mean(pvals.1.1$t[, 4] < 0) * 2
```

```r
pvals.1.1.list[5] <- mean(pvals.1.1$t[, 5] > 0) * 2
pvals.1.1.list[6] <- mean(pvals.1.1$t[, 6] > 0) * 2

# label output
pvals.1.1.out <- as.list(pvals.1.1.list)
names(pvals.1.1.out) <- row.names(as.data.frame(summary(glm1.1)$coefficients))
pvals.1.1.out
```

```
## $`(Intercept)`
## [1] 0
##
## $Phase1
## [1] 0
##
## $Phase2
## [1] 0
##
## $Reward_Category
## [1] 0.64
##
## $`Phase1:Reward_Category`
## [1] 0.82
##
## $`Phase2:Reward_Category`
## [1] 2
```

Firstly, the GLMMM analysis on Say_Old responses can be used to analyse participants overall performance on the memory task. The 'Intercept' term which is negative, $\beta$ = -1.48, 95% CI [-1.63, -1.33], p < .001, represents the log odds of answering 'old' to a new item. Whereas, the 'Phase 1' and 'Phase 2' predictor estimates are positive, $\beta$ = 2.16, 95% CI [1.97, 2.33], p < .001, and $\beta$ = 2.37, 95% CI [2.17, 2.52], p < .001, respectively, showing that participants have successfully remembered previously seen items. The model shows no significant effects on response bias for items in the high reward category.

There was no significant interaction between reward category and the pre-conditioning phase. However, there was a trend level interaction with the conditioning phase, $\beta$ = 0.01, 95% CI [-0.01, 0.22], p = .09, since the confidence interval includes zero (possibility of no effect), we cannot be confident about this effect being significant.

## 1.2 Experiment 3 (High Certainty Memory)

```r
glm1.2 <- glmer(Say_Old ~ 1 + Phase * Reward_Category + (1 |
    UserID) + (1 | Stim), family = binomial(link = "logit"),
    data = data.high.x3, glmerControl(optimizer = "bobyqa"))
summary(glm1.2)
```

```
## Generalized linear mixed model fit by maximum likelihood (Laplace
##   Approximation) [glmerMod]
##  Family: binomial  ( logit )
## Formula: Say_Old ~ 1 + Phase * Reward_Category + (1 | UserID) + (1 | Stim)
##    Data: data.high.x3
## Control: glmerControl(optimizer = "bobyqa")
```

```
##
##      AIC      BIC   logLik deviance df.resid
##  23067.4  23132.7 -11525.7  23051.4    26072
##
## Scaled residuals:
##      Min       1Q   Median       3Q      Max
## -13.5547  -0.4171  -0.1283   0.4969   9.6124
##
## Random effects:
##  Groups Name        Variance Std.Dev.
##  Stim   (Intercept) 0.7323   0.8558
##  UserID (Intercept) 0.6821   0.8259
## Number of obs: 26080, groups:  Stim, 240; UserID, 170
##
## Fixed effects:
##                         Estimate Std. Error z value Pr(>|z|)
## (Intercept)             -1.83326    0.10857 -16.886   <2e-16 ***
## Phase1                   3.06467    0.12559  24.401   <2e-16 ***
## Phase2                   3.31063    0.12623  26.227   <2e-16 ***
## Reward_Category          0.04545    0.05362   0.848    0.397
## Phase1:Reward_Category   0.00054    0.08071   0.007    0.995
## Phase2:Reward_Category   0.07809    0.08230   0.949    0.343
## ---
## Signif. codes:  0 '***' 0.001 '**' 0.01 '*' 0.05 '.' 0.1 ' ' 1
##
## Correlation of Fixed Effects:
##             (Intr) Phase1 Phase2 Rwrd_C P1:R_C
## Phase1      -0.570
## Phase2      -0.568  0.882
## Rewrd_Ctgry -0.251  0.218  0.217
## Phs1:Rwrd_C  0.166 -0.322 -0.143 -0.663
## Phs2:Rwrd_C  0.163 -0.141 -0.325 -0.651  0.432
```

```
# confint.1.2 <- confint.merMod(glm1.2, method = 'boot',
# nsim = Nsim, parallel = 'multicore',ncpus = 4) pvals.1.2
# <- bootMer(glm1.2, FUN = fixef, nsim = Nsim, parallel =
# 'multicore', ncpus = 4) # saveRDS(confint.1.2,
# 'x3_confs/confint.1.2.rds') saveRDS(pvals.1.2,
# 'x3_confs/pvals.1.2.rds')
```

```
# load previously run results
confint.1.2 <- readRDS(file = "x3_confs/confint.1.2.rds")
confint.1.2
```

```
##                              2.5 %     97.5 %
## .sig01                  0.75271462  0.9552424
## .sig02                  0.70809006  0.9218106
## (Intercept)            -2.05428587 -1.5953969
## Phase1                  2.80491228  3.3719732
## Phase2                  3.03272538  3.6418026
## Reward_Category        -0.06933965  0.1466981
## Phase1:Reward_Category -0.14606515  0.1603142
## Phase2:Reward_Category -0.08575401  0.2383384
```

```r
pvals.1.2 <- readRDS(file = "x3_confs/pvals.1.2.rds")
pvals.1.2
```

```
##
## PARAMETRIC BOOTSTRAP
##
##
## Call:
## bootMer(x = glm1.2, FUN = fixef, nsim = Nsim, parallel = "multicore",
##     ncpus = 4)
##
##
## Bootstrap Statistics :
##          original        bias    std. error
## t1* -1.8332528723 -3.293966e-03  0.10468621
## t2*  3.0646641555  1.467289e-02  0.11878040
## t3*  3.3106182282  2.033728e-02  0.13189386
## t4*  0.0454420109  9.633102e-04  0.04915033
## t5*  0.0005447306  9.497839e-05  0.08334939
## t6*  0.0781012838 -8.282108e-03  0.07806830
```

```r
pvals.1.2.list <- mean(pvals.1.2$t[, 1] > 0) * 2
pvals.1.2.list[2] <- mean(pvals.1.2$t[, 2] < 0) * 2
pvals.1.2.list[3] <- mean(pvals.1.2$t[, 3] < 0) * 2
pvals.1.2.list[4] <- mean(pvals.1.2$t[, 4] < 0) * 2
pvals.1.2.list[5] <- mean(pvals.1.2$t[, 5] > 0) * 2
pvals.1.2.list[6] <- mean(pvals.1.2$t[, 6] > 0) * 2

# label output
pvals.1.2.out <- as.list(pvals.1.2.list)
names(pvals.1.2.out) <- row.names(as.data.frame(summary(glm1.2)$coefficients))
pvals.1.2.out
```

```
## $`(Intercept)`
## [1] 0
##
## $Phase1
## [1] 0
##
## $Phase2
## [1] 0
##
## $Reward_Category
## [1] 0.38
##
## $`Phase1:Reward_Category`
## [1] 0.92
##
## $`Phase2:Reward_Category`
## [1] 1.7
```

When considering only the trials in which participants provided high certainty responses during the memory test, the overall pattern of results does not change compared to all trials analysis shown above. The trend

level interaction between reward category and conditioning phase (Phase 2) was weaker than seen in the analysis with all trials, $\beta = 0.08$, 95% CI [-0.09, 0.24], p = .34 and includes zero in the confidence interval again. The model shows no significant effects on response bias for items in the high reward category.

# 2. Supplementary Analysis (Exclusion Based on Surprisal)

```r
# Participants to exclude as they anticipated the memory
# test
data.x3.exclude <- read.csv("adaptiveMemoryReplication/Exp3_CleanData/Supp/x3_Surprisal_Exclude.csv")
x3.exclude <- subset(data.x3.exclude, Exclude.Surprise == 1,
    UserID)

# Data subset (not surprised)
data.x3.ns <- data.x3[!data.x3$UserID %in% x3.exclude$UserID,
    ]
data.x3.high.ns <- data.high.x3[!data.high.x3$UserID %in% x3.exclude$UserID,
    ]
```

## 2.1 Experiment 3 (All Memory)

```r
glm2.1 <- glmer(Say_Old ~ 1 + Phase * Reward_Category + (1 |
    UserID) + (1 | Stim), family = binomial(link = "logit"),
    data = data.x3.ns, glmerControl(optimizer = "bobyqa"))
summary(glm2.1)
```

```
## Generalized linear mixed model fit by maximum likelihood (Laplace
##   Approximation) [glmerMod]
##  Family: binomial  ( logit )
## Formula: Say_Old ~ 1 + Phase * Reward_Category + (1 | UserID) + (1 | Stim)
##    Data: data.x3.ns
## Control: glmerControl(optimizer = "bobyqa")
##
##      AIC      BIC   logLik deviance df.resid
##  26778.3  26843.3 -13381.2  26762.3    24739
##
## Scaled residuals:
##     Min      1Q  Median      3Q     Max
## -5.5881 -0.6154 -0.2785  0.6699  6.6505
##
## Random effects:
##  Groups Name        Variance Std.Dev.
##  Stim   (Intercept) 0.4376   0.6615
##  UserID (Intercept) 0.3409   0.5838
## Number of obs: 24747, groups:  Stim, 240; UserID, 104
##
## Fixed effects:
##                          Estimate Std. Error z value Pr(>|z|)
## (Intercept)              -1.47314    0.08999 -16.370   <2e-16 ***
## Phase1                    2.13940    0.10056  21.276   <2e-16 ***
```

```
## Phase2                  2.30374    0.10093  22.826   <2e-16 ***
## Reward_Category         0.09627    0.04568   2.108   0.0351 *
## Phase1:Reward_Category -0.05787    0.07275  -0.795   0.4263
## Phase2:Reward_Category  0.11359    0.07426   1.530   0.1261
## ---
## Signif. codes:  0 '***' 0.001 '**' 0.01 '*' 0.05 '.' 0.1 ' ' 1
##
## Correlation of Fixed Effects:
##             (Intr) Phase1 Phase2 Rwrd_C P1:R_C
## Phase1      -0.534
## Phase2      -0.533  0.839
## Rewrd_Ctgry -0.261  0.234  0.233
## Phs1:Rwrd_C  0.164 -0.365 -0.146 -0.628
## Phs2:Rwrd_C  0.160 -0.142 -0.363 -0.615  0.385
```

## 2.2 Experiment 3 (High Certainty Memory)

```
glm2.2 <- glmer(Say_Old ~ 1 + Phase * Reward_Category + (1 |
    UserID) + (1 | Stim), family = binomial(link = "logit"),
    data = data.x3.high.ns, glmerControl(optimizer = "bobyqa"))
summary(glm2.2)
```

```
## Generalized linear mixed model fit by maximum likelihood (Laplace
##   Approximation) [glmerMod]
##  Family: binomial  ( logit )
## Formula: Say_Old ~ 1 + Phase * Reward_Category + (1 | UserID) + (1 | Stim)
##    Data: data.x3.high.ns
## Control: glmerControl(optimizer = "bobyqa")
##
##      AIC      BIC   logLik deviance df.resid
##  14379.0  14440.4  -7181.5  14363.0    15923
##
## Scaled residuals:
##     Min      1Q  Median      3Q     Max
## -8.2565 -0.4317 -0.1186  0.5020  8.0258
##
## Random effects:
##  Groups Name        Variance Std.Dev.
##  Stim   (Intercept) 0.7586   0.8710
##  UserID (Intercept) 0.6851   0.8277
## Number of obs: 15931, groups:  Stim, 240; UserID, 104
##
## Fixed effects:
##                        Estimate Std. Error z value Pr(>|z|)
## (Intercept)            -1.80069    0.12484 -14.424   <2e-16 ***
## Phase1                  2.98369    0.13579  21.973   <2e-16 ***
## Phase2                  3.16794    0.13625  23.251   <2e-16 ***
## Reward_Category         0.11447    0.06729   1.701   0.0889 .
## Phase1:Reward_Category  0.02512    0.10297   0.244   0.8073
## Phase2:Reward_Category  0.15387    0.10453   1.472   0.1410
## ---
## Signif. codes:  0 '***' 0.001 '**' 0.01 '*' 0.05 '.' 0.1 ' ' 1
```

```
##
## Correlation of Fixed Effects:
##             (Intr) Phase1 Phase2 Rwrd_C P1:R_C
## Phase1      -0.531
## Phase2      -0.530  0.837
## Rewrd_Ctgry -0.281  0.259  0.258
## Phs1:Rwrd_C  0.182 -0.381 -0.169 -0.652
## Phs2:Rwrd_C  0.179 -0.166 -0.379 -0.643  0.422
```

When considering only participants who were surprised by the memory test, we again see the same pattern of results as in the full analysis, with the only difference being the response bias which emerges for items in the high reward category. This is signified by the coefficient of 'Reward Category'. In other words, this is the effect of reward category on the log odds of Say_Old response. The positive and significant coefficient means that participants were more likely to respond 'old' to new items in the high reward category in general (a more liberal response bias) which would have an influence on memory effects.
